# Cognitive decline in older adults with type 2 diabetes: Unraveling site-specific glycoproteomic alterations

**DOI:** 10.1371/journal.pone.0318916

**Published:** 2025-05-08

**Authors:** Yishai Levin, Nili Tickotsky, David Morgenstern, Hila Wolf-Levy, Barak Markus, Itzik Cooper, Anat Reiner-Benaim, Jaime Uribarri, Ron Unger, Aron S. Buchman, Michal Schnaider Beeri

**Affiliations:** 1 The de Botton Institute for Protein Profiling, Nancy and Stephen Grand Israel National Center for Personalized Medicine, Weizmann Institute of Science, Rehovot, Israel; 2 The Joseph Sagol Neuroscience Center, Sheba Medical Center, Ramat Gan, Israel; 3 The Mantoux Bioinformatics Institute of the Nancy and Stephen Grand Israel National Center for Personalized Medicine, Weizmann Institute of Science, Rehovot, Israel; 4 School of Psychology, Reichman University, Herzliya, Israel; 5 School of Medicine, Tel-Aviv University, Tel-Aviv, Israel; 6 Department of Epidemiology, Biostatistics and Community Health Sciences, School of Public Health, Faculty of Health Sciences, Ben Gurion University of the Negev, Be’er-Sheva, Israel; 7 Department of Medicine, The Icahn School of Medicine at Mount Sinai, New York, New York, United States of America; 8 The Goodman faculty of life sciences, Bar Ilan University, Ramat Gan, Israel.; 9 Rush Alzheimer’s Disease Center, Rush University Medical Center, Chicago, Illinois, United States of America; 10 Department of Psychiatry, The Icahn School of Medicine at Mount Sinai, New York, New York, United States of America; 11 The Herbert and Jackeline Krieger Klein Alzheimer’s Research Center, Brain Health Institute, Rutgers University, New Brunswick, New Jersey, United States of America; Augusta University, TAIWAN

## Abstract

Type 2 diabetes (T2D) is consistently related to an increased risk of cognitive decline and dementia. However, the molecular underpinnings of this association remain poorly understood. In this study, we applied a novel mass spectrometry-based glycoproteomic methodology to profile serum glycoproteins in older adults with T2D, aiming to identify glycopeptiforms associated with cognitive impairment. Our method allowed comprehensive profiling of N glycosylation in addition to the unique ability to profile glycation events on specific amino acid sites. Serum samples from initially cognitively normal older adults with T2D were collected, with participants classified as cognitive decliners (who developed impairment) and non-decliners (who maintained normal cognition over time). We identified significant differences in the abundance of glycopeptiforms between these groups, noting that certain glycopeptiforms exhibited unique changes over time in decliners. We identified 13 glycopeptiforms that exhibited significant differences between the groups both at baseline and in their rates of change over time. Pathway analysis indicated that glycation events were linked to metabolic pathways while glycosylation to immune-related pathways, aligning with established links between these processes and cognitive decline. This study offers new insights into glycoproteoform alterations in older adults with T2D experiencing cognitive decline. It highlights the potential of specific glycopeptiforms as biomarkers for early cognitive impairment in T2D. Further validation in larger cohorts will enhance our understanding of glycosylation and glycation in T2D and potentially lead to the discovery of novel treatment targets for T2D-related cognitive decline. Raw data and search are available via ProteomeXchange with identifier PXD050780.

## Introduction

Type 2 diabetes (T2D) is a chronic metabolic disorder consistently associated with an elevated risk for cognitive decline and dementia [1,2]. While numerous mechanisms for the association of T2D with cognitive decline have been explored, encompassing chronic inflammation, vascular damage, oxidative stress, and advanced glycation end products [3], the precise molecular processes underlying or linking these two conditions remain elusive. This knowledge gap impedes the ability to detect the earliest stages of cognitive decline in older adults with T2D [4,5], consequently hindering the advancement of preventive interventions.

While the protein’s amino acid sequence is coded in the genome, posttranslational modifications (PTMs) commonly alter protein function by changing their physical and chemical properties. The most common PTM is the addition of glycans [6] resulting in glycoproteoforms, which have crucial roles in almost all cellular processes, including cell signaling, immune recognition, and cell-cell interaction [7,8]. Glycan structures are highly diverse, and they can be altered by changes in physiological conditions of the organism and cells [8,9]. Consequently, glycan heterogeneity may contribute and provide a sensitive indicator for age-related cellular changes and diverse diseases of aging, including diabetes and dementia [7,10].

Evidence regarding the role of glycosylation in T2D and its complications has advanced significantly in recent years [11,12]. T2D is known to be associated with systemic inflammation and increased secretion of pro-inflammatory cytokines [13–15] which in turn, modulate glycosyltransferase expression [16,17], leading to altered protein glycosylation patterns. Furthermore, glycosylation has been implicated in glucose-stimulated insulin secretion by modulating expression of glucose transporters [18]. However, a scarcity of comprehensive serum glycoproteomic data exists concerning T2D and late-life cognitive impairment. A recent targeted glycoproteomics study on individuals with T2D, showed altered N-glycosylation on 18 plasma proteins whose expression levels changed in individuals who had cognitive decline [19]. Technical challenges in measuring glycoproteoforms have impeded studies of the glycoproteome and thus their link to T2D and cognitive decline.

Glycoproteoforms are created by two main types of sugar-adding reactions, glycosylation and glycation [20]: Glycosylation is a highly regulated enzymatic reaction that is part of normal protein biosynthesis, and creates complex glycan structures. It is the most abundant PTM encountered in nature [9], and the mammalian glycome repertoire contains thousands of glycan structures. Glycosylation can occur at different amino acid residues in the protein sequence. The most common and widely studied forms are N-linked and O-linked glycosylation. O-linked glycans are linked to the hydroxyl group on Ser or Thr residues. N-linked glycans are attached to the amide group of Asn residues in a sequence motif of Asn-X-Ser/Thr (where X can be any amino acid except proline) [21]. Glycation, on the other hand, is a non-enzymatic, non-regulated reaction associated with diseases or pathological changes related to hyperglycemia in old age, primarily T2D [22,23]. In its initial form, a simple sugar, hexose, is attached to lysine on a protein. Later it develops to become advanced glycation end-products (AGEs), which are a complex and heterogeneous group of compounds that have been implicated in diabetes related complications [24] and in cognitive decline and Alzheimer’s disease [25,26].

Bottom-up mass spectrometry-based glycoproteomics aims to identify both the amino acid sequence and the attached glycan composition. However, it can only determine the sugar subunits of the glycans and not their overall structure. In contrast, other approaches involve cleaving off the glycans and analyzing them separately from the protein, a method known as glycomics, which enables the identification of glycan structures by comparison to standards [27].

The two main challenges in glycoproteomics are the enrichment of glycopeptides and data processing. Numerous strategies for glycopeptide enrichment have been devised, however, in most instances, these methods exhibit either suboptimal enrichment efficiency or a bias towards specific subsets of glycopeptides, thereby introducing a significant distortion in profiling efforts. The prevailing techniques, namely hydrophilic interaction liquid chromatography (HILIC) and the utilization of multiple lectins, are commonly employed. HILIC, while popular due to its simplicity, suffers from low enrichment efficiency, targeting predominantly highly hydrophilic peptides and displaying bias against hydrophobic glycopeptides and peptides with small glycans. On the other hand, lectin affinity offers a focused approach for enriching particular glycan subclasses, and the use of multiple lectins widens the spectrum, albeit without achieving unbiased enrichment.

The use of boronic acid emerges as a promising approach for glycopeptide enrichment. Boronic acids enable reversible, pH-dependent covalent binding to cis-diols present in sugars, holding the promise of unbiased enrichment across the entire glycopeptide population in the proteome. Numerous method papers describe innovative chemistries and approaches employing boronic acids for glycopeptide enrichment. While some experiments have achieved extensive glycoproteomic coverage, they necessitated prefractionation to attain such results [28]. We recently introduced a novel boronic acid-based protocol, significantly enhancing identification and quantification of glycopeptides while enabling detection of glycated glycopeptides in the same experiment [29].

The second challenge in glycoproteomics is data processing. The requirement for identifying both the peptide backbone and the composition of the glycan from the same fragmentation spectrum presents a major challenge. This is further complicated by the fact that typical glycan databases include dozens of glycans. So unlike other posttranslational modifications, where each modification is represented by one option of a modification mass, in glycoproteomics, it includes up to dozens of different glycan masses. This represents a very large search space, particularly for O-linked glycans which can be found on any serine or threonine. For N-linked glycans, the search space is much smaller due to a restrictive amino acid sequence motif of N-X-S/T. In the human proteome, for example, there are 30,742 theoretical N-glycosylation sites [30], which is a modest increase to a three million peptide search space of typical tryptic cleavage (based on the human SwissProt database, 7–30 amino acid peptides, up to 2 miscleavages). There are a number of software tools available for processing mass spectrometry based glycoproteomics data. One of the leading software packages for this type of analysis is Byonic, which is among the most successful in comparative studies [31]. However there seems to be a lack of consensus as to how to filter glycopeptide identification reported by Byonic, as people use different scoring cutoffs [32–34].

To address these challenges, we developed an LC-MS/MS–based methodology that can identify proteins modified by both glycation and glycosylation and the locations where glycans link to proteins [29]. In this ‘bottom-up’ approach, proteins are digested with trypsin, followed by glycopeptide enrichment, LC-MS/MS and data processing. We also added a novel step of searching against a target glycan database and a decoy glycan database. This allows us to report the false discovery rate of both peptide sequence and glycan composition.

Here, we applied this novel methodology to profile the serum glycoproteome of older adults with T2D. The goal was to test the hypothesis that serum glycopeptiforms, a peptide with a specific glycan composition at a specific site on a protein, representing glycoproteoforms, are associated with late-life cognitive impairment in T2D. We were able to analyze protein N-linked glycosylation, with the added benefit of extensive characterization of the serum protein glycation, a major driver of T2D complications [35,36] including dementia [25,26]. We compared serum expression levels of glycopeptiforms at in older adults with T2D who later developed cognitive impairment, and controls who maintained normal cognition over time.

## Methods

### Participants

Participants were from the ongoing Israel Diabetes and Cognitive decline (IDCD) study [37], a community-based longitudinal cohort study of the chronic conditions of aging in older adults with T2D that began in 2010. To date the IDCD study has recruited 1211 eligible participants, all from the Maccabi Health Services (MHS), the second largest HMO in Israel, whose exquisitely detailed electronic medical records include diagnoses, blood work, and medications since 1998. The study was approved by all three IRB committees involved with the IDCD study (Ichan School of Medicine at Mount Sinai, NY, Sheba Medical Center, Israel and Maccabi Heath Services, Israel, approval 5555-18). All participants signed informed consent.

Criteria for enrolment into the IDCD study were: (1) having T2D; (2) ≥  65 years of age, (3) being free of major neurological (e.g., Parkinson’s disease, stroke), psychiatric (e.g., schizophrenia) or other diseases (e.g., alcohol or drug abuse) that might affect cognition; (4) having an informant; (5) fluency in Hebrew; (6) living in the area of Tel-Aviv; (7) normal cognition at study entry.

### Determination of cognitive “decliners” versus “non-decliners” status

All IDCD study participants undergo cognitive assessment about 18-months apart. To ensure accurate classification of cognitive status, we included participants with three cognitive assessments. This allowed us to confirm true cognitive decline. However, the glycoproteomic analyses were performed only on blood samples from the **first** and **third** cognitive assessments. However, the glycoproteomic analyses were performed only on blood samples from the first and third cognitive assessments. A multidisciplinary diagnostic consensus conference, composed of neurologists, geriatric psychiatrists (at least one of the expertise), and neuropsychologists, determine the cognitive status at each visit based on all available clinical data as well as the Clinical Dementia Rating scale (CDR) [38]. Through an interview with the participant and an informant, the CDR assesses the severity of cognitive and functional impairment in 6 domains: memory, orientation, judgment and problem-solving, community affairs, home and hobbies, and personal care. A CDR = 0 represents normal cognition, CDR = 0.5 reflects questionable dementia, or CDR = 1–3 represents mild, moderate, or severe dementia. The assessment date range is 2/26/2007–3/12/2020.

In the IDCD study, cognitive impairment is an exclusion criterion, so all participants of this study began with normal cognitive function (CDR = 0). For the purpose of this study, two groups were randomly selected from IDCD participants: The first (N = 8) was comprised of individuals who initially had normal cognition (CDR = 0) but later developed cognitive impairment (CDR > 0.5) in at least the first and second follow-ups, forming the **‘cognitive decliners’** group. The second group (N = 14) maintained normal cognition throughout the IDCD study, with a CDR = 0 at all three time points, and are referred to as **‘non-decliners’**. The two groups were matched on baseline age, education, HbA1c and duration of type 2 diabetes. The goal for matching the two groups on baseline HbA1c is to ensure that the effect of glycopeptiforms on cognition is independent of glycemic control.

### Collection of blood samples and clinical characteristics

Blood samples were obtained in the morning after a 10-h fast. Blood was drawn into serum separator tubes, centrifuged, aliquoted into 0.1 ml vials, and stored at -80 C. Information on demographic (age, gender and years of education) was collected at baseline of the IDCD. HbA1c, systolic and diastolic blood pressure (measured sitting), body mass index (measured as weight in kg divided by height square in meters), creatinine and cholesterol levels were calculated as the average of all lab results available in MHS for each participant until the IDCD study baseline [37], see **S1 File**. The first HbA1c data point is on 12/31/2000 and the last from 7/6/2020.

### Glycoproteomics analysis

We refer to the specific glycan composition on a specific amino acid site of a protein as a “glycopeptiform”. We conducted MS1-intensity-based, label-free, quantitative glycoproteomic analysis on sera obtained from study participants for a total of 44 samples (22 baselines and their respective 22 last available followup), for 8 ‘decliners’ and 14 ‘non-decliners’, which were processed and analyzed in a randomized order in one batch.

The glycoproteomic method allows for measurement of glycopeptiforms, facilitating both identification of the glycan composition and the site on the tryptic peptide. As in all ‘bottom -up’ approaches, the peptide is used to identify the protein. In addition to glycosylated peptides, our enrichment method also has the unique feature of enriching for glycated peptides (Hex modification on Lys).

**Fig 1:**
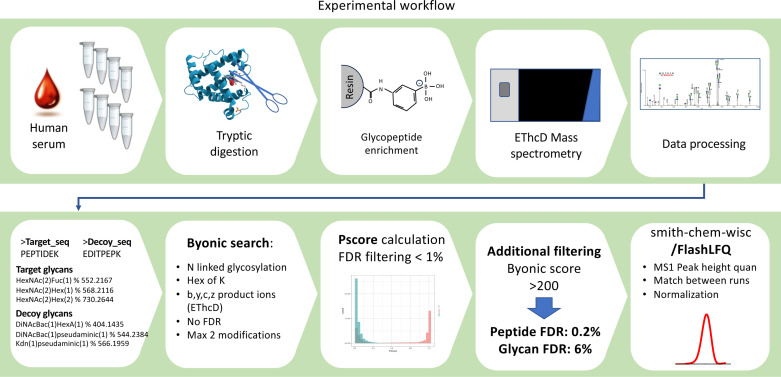
Description of the pipeline used to identify glycopeptiforms associated with cognitive impairment, as detailed in Morgenstern *et al* [29].

Serum samples were digested, then enriched using a boronic acid derivative, and analyzed on a Fusion Lumos mass spectrometer in EThcD mode. Data was processed using Byonic, including both peptide sequence decoys and glycan decoys, for generating glycopeptiform identifications and FlashLFQ software for extracting the glycopeptiform quantitative information as detailed in Morgenstern *et al* [29] and **Fig 1**. The approach facilitates identification and relative MS1 based quantification of early glycation events as well as N linked glycosylation. Furthermore, we applied the target-decoy method for both peptide sequence identification and for identifying the glycan composition.

### Sample preparation

Samples were prepared using the S-trap method in a 96 well plate format as described in Elinger *et al* [39]. It should be noted that we [40] and others [41,42] have shown that the age of the serum does not affect the ability of trypsin to digest the samples. For all samples, the total protein concentration was measured using a BCA assay. One hundred micrograms of each sample were used for downstream preparation. Dithiothreitol (DTT) was prepared fresh in 50 mM ammonium bicarbonate and added to a final concentration of 5 mM. Samples were then incubated at 56 °C for 1 h. Iodoacetamide was prepared fresh in 50 mM ammonium bicarbonate and added to a final concentration of 10 mM. Samples were incubated in the dark for 45 min. Phosphoric acid was added to the samples to a final concentration of 1%. The samples were mixed with 350 μL of 90% MeOH +  10% 50 mM ammonium bicarbonate and then transferred to the S-trap plate (Protifi, USA), centrifuged for 1 min at 4000g, washed three times with 400 μL of 90% MeOH +  10% 50 mM ammonium bicarbonate, and then centrifuged at 4000g for 1 min. Four microliters of 0.5 μg/μL trypsin in 125 μL in ammonium bicarbonate (50:1 protein amount:trypsin) was added to the samples. Samples were incubated at 37 °C overnight. The next day, peptides were eluted using 80 μL of 50 mM ammonium bicarbonate, which was added to the S-trap cartridge and centrifuged at 4000g for 1 min into new tubes, and the peptides were then collected. Then, a second digestion was performed using 4 μL of 0.5 μg/μL trypsin in 50 mM ammonium bicarbonate, which was added to the eluted samples and incubated at 37 °C for 4 h. Two more elations from the S-trap cartridge were performed: one was carried out with 80 μL of 0.2% formic acid, which was added to the S-trap cartridge and spun down at 4000g for 1 min. The second was done using 80 μL of 50% acetonitrile +  0.2% formic acid, which was added to the cartridge and spun down at 4000g for 1 min. The three elutions were mixed and dried using a vacuum centrifuge (Centrivac, LabConco).

### Glycopeptide enrichment

Resulting peptides from each sample were enriched by using polyethyleneimine (PEI)-benzoboroxole beads prepared in-house as detailed in Morgenstern *et al* [29]. Twenty microliters of derivatized beads (per sample) were spun down to remove storage buffers and washed with the loading buffer twice. Samples were dissolved in 50 μL of loading buffer (50% MeCN, 50 mM carbonate pH 10.5, 1 M KCl) and added to the beads. Beads were incubated in rotation at RT for 30 min, loaded on empty TopTips (10 μL) and spun in a centrifuge at 376g for 30 s to remove the solution. Beads were washed twice with the loading buffer and twice with the washing buffer (50% MeCN, 50 mM carbonate pH 10.5). Twenty microliters of 5% formic acid/50%ACN was added to the samples, incubated for 10 min at RT to elute the bound glycopeptides. The samples were spun for 60 s. The above volume of elution buffer was added again and eluted immediately.

### Mass spectrometry

Samples were reconstituted in 15 µ L 3% Acetonitrile/0.1% formic acid. 1.5 µ L were loaded on a Symmetry trap column (C18, 180µm * 20mm, 5µm, 100A, Waters inc.) followed by separation using the HSS T3 analytical column (C18, 75µm * 250mm, 1.8µm, 100A, Waters inc.), mounted on a nanoAcquity running at a flow of 0.35 µ L/min. Mobile phase consisted of A: H_2_O+0.1% formic acid, B: ACN + 0.1% formic acid. Peptides were separated using a gradient of 4–25% B in 125min, followed by 25–40% in 30 minutes, 40% to 90% in 5 min, maintained at 90% for 7min and back to initial conditions. The column was connected to the mass spectrometer via a FlexIon electrospray ionization source (Thermo) and a 10 µm tip emitter (Fossil, Spain). Data was acquired with the Orbitrap Fusion Lumos (Thermo Fisher scientific) running 3sec top-speed data dependent acquisition (DDA) method, EThcD fragmentation mode. MS1 scans were performed at 120,000 resolution (at 200 m/z), in 400-1800m/z range. Most abundant ions at charge states 2–8, and at minimum 5e4 intensity were chosen for fragmentation. Precursors were isolated in the quadrupole using 1m/z isolation window. MS2 fragmentation was performed using EThcD with calibrated charge-dependent parameters and supplemental activation of 15 NCE. MS/MS was acquired at 15,000 resolution (at 200m/z), using a first mass of 120 m/z with standard automatic gain control (AGC) and maximum injection time of 120ms.

The samples were run in a random order and a HeLa digest (Pierce, Thermo) was run every 8 samples as part of Quality Control during the run. Chromatograms of all 44 runs are provided in S2 File.

### Data processing and analysis

Raw data were processed by Byonic software [29] version 5.1.1. Data were searched against a plasma proteome previously published [43] and, with a common contaminant library appended for a total of 4,578 sequences (See S3 File). Data was also searched against a glycan database, containing 141 human plasma glycans [44–46] and 141 non-human glycans which served as decoys (S4 File).

All searches used specific trypsin cleavage with up to 2 missed cleavages. Fragmentation was set to EThcD. Mass tolerances were set to 10ppm for MS1 and 20ppm for MS2. N-glycopeptide search was enabled (based on the sequence motif N-X-S/T). The following modifications were allowed: fixed carbamidomethylation on C, variable oxidation on M (common1), hex on K (common 1), protein N-terminal acetylation (rare 1), for up to two common modifications and one rare modifications. Each sample was searched without filtering for false discovery rate (FDR).

Since the process of identifying the peptide sequence and the glycan composition from the same MS/MS spectrum is a complex process and since there is no consensus in the literature how to filter the Byonic glycopeptide output, we took a conservative approach for calculating the FDR and a hard score cutoff. FDR for peptide identification was controlled by the following procedure: Byonic provides 3 quality scores for each pepide-spectrum-match: Score, Log-P, and Delta-Mod. A combined P-score was defined by regressing the Decoy state of all peptides on Log-P and Delta-Mod scores. A lower-bound threshold for P-score was chosen such that the Decoy-FDR is less than 1%. S5 File shows the P-score distributions for the target and decoy peptide spectrum matches (PSMs). The remaining peptides were further filtered on Score > 200. The resulting effective FDR was 0.2% at the PSM level. A master PSM table is provided as S6 File. The filtered PSM list was used as input to the FlashLFQ software [47] for generating the MS1 based intensities, using default settings, including match-between-runs and normalization.

The raw mass spectrometry data, Byonic search output files, a concatenated file of all search results and the formatted file for FlashLFQ have all been deposited to the ProteomeXchange Consortium via the PRIDE [48] partner repository with the dataset identifier PXD050780 and 10.6019/PXD050780

### Statistical analyses

We compared the decliners group, who developed cognitive decline over time, to the non-decliners group, who maintained normal cognition throughout all follow up assessments. Prior to the univariate statistical analysis, missing values (36% of the data) were imputed from a normal distribution of the log-transformed intensities by drawing from a shifted normal distribution representing the background noise of the spectra. The mean of the noise distribution was shifted from the original distribution by 1.8 standard deviation (STD) units, and the corresponding STD was multiplied by a factor of 0.3 from the original STD, as commonly used [49]. Finally, glycopeptiforms with at least 70% valid values in at least one group were kept for statistical analysis.

To find glycopeptiforms associated with cognitive impairment, we regressed the log intensities against group (decliners versus non-decliners) using a linear regression model. We adjusted for HbA1c levels because the difference between the groups in HbA1c was borderline significant and we aimed to ensure that our results are not due to a difference in glycemic control. Results without adjustment for HbA1c were very similar so we chose to report them with the HbA1c adjustment. For the analysis of differences between the groups at time point 1, we utilized the lm() function from the R stats package to perform the regression analysis. P values were calculated with a Student’s T Test against the null hypotheses that the coefficient is equal to zero. The effect-size (+/-2 fold change) and p-values (set at < 0.05) corresponding to the coefficients of the decliner/non-decliner variable, were set a-priori to identify significant glycopeptiforms. These cutoffs were chosen to be somewhat conservative compared to previous longitudinal plasma proteomics studies [19].

We also utilized linear mixed effects models to analyze the changes in expression between time points 1 and 2, focusing on the differences between decliners and non-decliners over time. The model was implemented using the R package lmerTest. The fixed effects included group (decliner and non-decliner), visit time, baseline age, and mean HbA1c scores. Subjects were treated as a random effect to account for the repeated measures from the two visits. The difference in the rate of change across time between cases and controls was assessed through the interaction term between visit time and group. In S7 File, which summarizes the statistical results, the effect size indicates the difference in the rate of change from time point 1 to time point 2 between decliners and non-decliners. The p-value reflects the significance of this difference.

All statistical analyses were performed in R (version 4.2.2). Biological function enrichment analysis was done using Reactome (www.reactome.org) [50], we chose an enrichment Q value cut off of < 1e-3.

## Results

### Description of participants

In this study, we performed glycoproteomic profiling of 44 serum samples collected from 22 older adults with T2D, who were initially cognitively normal. As described in the Methods, these participants were randomly selected from the IDCD study and divided into two groups: eight individuals who experienced cognitive decline and fourteen who maintained normal cognition over time. As shown in **Table 1**, cognitive decliners and non-decliners did not differ significantly in any demographic or clinical characteristic at baseline.

**Table 1:** Demographic and clinical characteristics of cognitive decliners versus non-decliners (Mean [SD]).

Covariates	Cognitive decliners [Mean (SD)]	Cognitive non-decliners [Mean (SD)]	Student’s T Test or χ² [p-value]
**CDR at time point 1**	0	0	Not applicable
**CDR at time point 2**	0.75 (0.27)	0	Not applicable
**Age at baseline (yrs)**	75.58 (2.90)	73.87 (2.18)	0.132
**Female**	1	4	0.200
**Male**	7	10	
**Education (yrs)**	12.13 (2.90)	13.29 (3.43)	0.430
**Mean BMI (kg/m2)**	26.92 (3.40)	27.29 (2.78)	0.782
**Mean systolic blood pressure (mmHg)**	132.81 (8.65)	132.18 (6.01)	0.841
**Mean diastolic blood pressure (mmHg)**	72.05 (3.86)	71.70 (3.99)	0.844
**Mean total cholesterol (mmol/L)**	172.42 (22.19)	161.71 (22.99)	0.300
**Mean serum creatinine (µmol/L)**	1.12 (0.34)	1.07 (0.33)	0.731
**Mean HbA1c (mmol/mol)**	6.95 (0.64)	6.52 (0.46)	0.084
**Duration of diabetes (years)**	10.39 (0.79)	10.66 (0.70)	0.417

### Identification of glycopeptiforms across the entire dataset

Our analysis started with a global inspection of the data. As noted in the Methods section, identification criteria included passing the identification FDR cutoff and replication criteria, which required a minimum of 70% intensity values in at least one of the study groups. In total, we identified and quantified 2,750 glycopeptiforms, at time point 1 of which 1,264 (46%) were glycated peptides. In the ‘rate of change’ analysis 2,963 glycopeptiforms were identified and quantified, 1,300 (44%) of which were from glycated peptides, all of which are listed in **S7 File**.

We compared the glycopeptiform intensity distributions in each sample, before and after normalization to check for outlier samples. S8 file shows there are no such outliers. Next, we evaluated the data globally using principal component analysis (PCA). When coloring the samples based on time point 1 and time point 2, a clear pattern emerged of a separation between the time points (see S9 File). To address this observation, we included the time points as a covariate in the linear mixed model, as noted in the Methods section.

**Fig 2:**
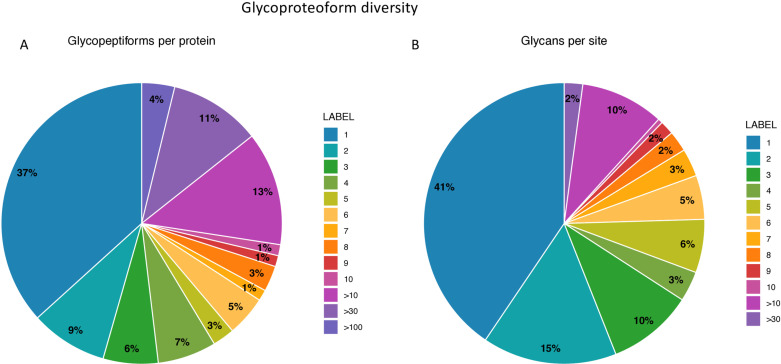
Pie charts showing the proportion of proteins having multiple glycopeptiforms (A) and the proportion of glycopeptiforms having multiple glycans on the same proteinsite (B).

We found many proteins with more than one unique glycopeptiform (namely, a unique sequence and glycan form). Moreover, we found sites on various proteins that were modified by multiple different glycan forms. To quantify this observation, we generated pie charts showing the proportion of proteins having multiple glycopeptiforms (**Fig 2A**) and the proportion of glycopeptiforms having multiple forms for the same protein site (**Fig 2B**). Over 60% of the proteins detected had two or more glycopeptiforms, a third of the proteins had over six glycopeptiforms. Furthermore, 59% of the proteins had at least two glycopeptiforms covering the same protein site.

### Identification of glycopeptiforms associated with cognitive decline

**Fig 3:**
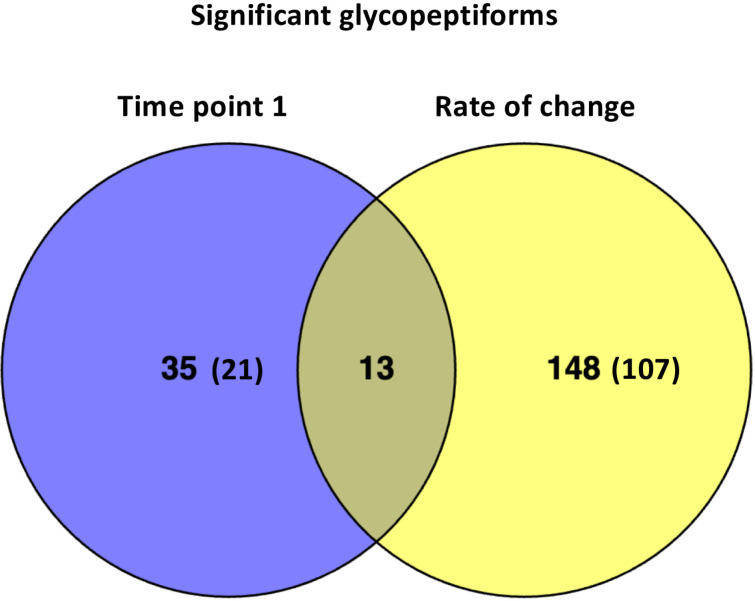
Venn diagram describing the number of glycopetiforms significantly related with baseline cognitive status and with rate of cognitive decline over time. The number is brackets indicates the number of glycated glycopeptiforms.

When comparing the cognitive decliners to the non-decliners groups, we found 48 glycopeptiforms to be significantly different between the groups at time point 1 and 161 showed significant differences over time between the two groups. **Fig 3** shows a Venn diagram describing the number of glycopetiforms significantly related with baseline (time point 1) cognitive status and with rate of cognitive decline over time Annotated MS/MS spectra for the significant glycopeptiforms are provided in S10 File. To test whether patient age affected the associations between glycopeptiforms and cognitive status, we repeated the analyses adjusting for age. Results remained essentially unchanged (data not shown).

Notably, the overall proportion of glycated glycopeptiforms, 44%, is not maintained in those that are significantly associated with cognitive decline. We found a higher proportion of differentially expressed glycated glycopeptiforms (60%) both in time point 1 and in the rate of change of glycated glycopeptiform expression over time (72%) (see S7 File, which can be filtered based on Hex on K).”

**Fig 4:**
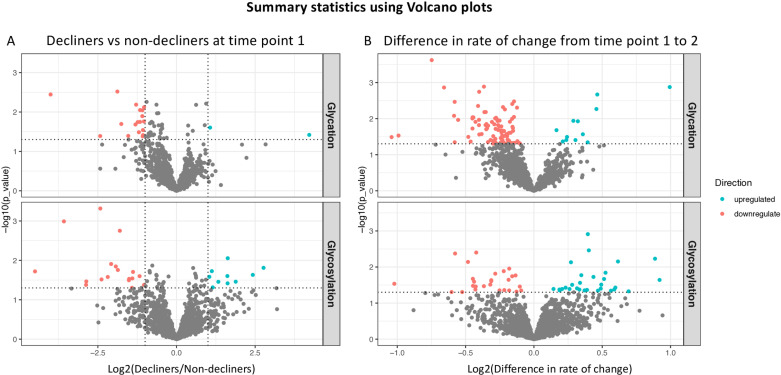
Volcano plots comparing the relative intensities of glycopeptiforms measured in serum samples from cognitive decliners versus non-decliners at time point 1 (A); and (B) the difference in rate of change between decliners and non-decliners from time point 1 to 2. Blue: glycopeptiforms that were significantly up-regulated in decliners. Red: glycopeptiforms that were significantly down-regulated in decliners. Criteria for significance for the comparison at time point 1: p-value <  0.05 and + /-2 fold change; for the rate of change: p < 0.05.

In order to visualize the expression patterns of glycated and glycosylated peptides separately, we generated volcano plots for time point 1 (T1) and the difference between the groups in changes of the glycopeptiform expression levels between T1 and time point 2 (T2) (**Fig 4**).

**Fig 5:**
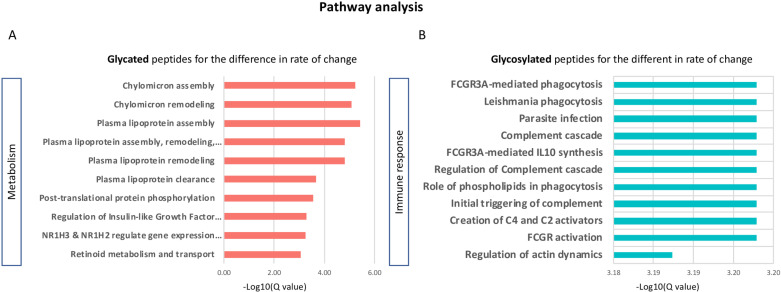
Pathway analysis based on the protein accessions from the significant glycopeptiforms separately for glycation and glycosylation. Only the results from the difference in rate of change yielded significant results. The analysis was done using Reactome and significance was set to q < 1e-3. A: downregulated, glycated glycopeptiforms; B: up-regulated glycosylated glycopeptiforms.

Next, we attempted to find biological insights based on these results. Since there are no knowledgebases that can be queried for glycoproteomics, we used Reactome [50], a knowledgebase of gene and protein expression. We submitted lists of protein accessions from the significant glycopeptiforms, separately for time point 1 and for the difference in rate of change and for the up or down regulated ones, separately for glycation and glycosylation. The results are summarized in **Fig 5**. Only the data from the difference in rate of change yielded significant results (q < 0.001). Also, significant results were only found for down-regulated glycated glycopeptiforms and for up-regulated glycosylated glycopeptiforms. Glycation was involved in metabolic pathways (5A) including lipoprotein assembly, remodeling and clearance as well as regulation of insulin like growth factor. There were no significant results from the up-regulated glycated glycopeptiforms. The glycosylated glycopeptiforms were mainly involved in immune response (5B).

### Serum amyloid component P

**Fig 6:**
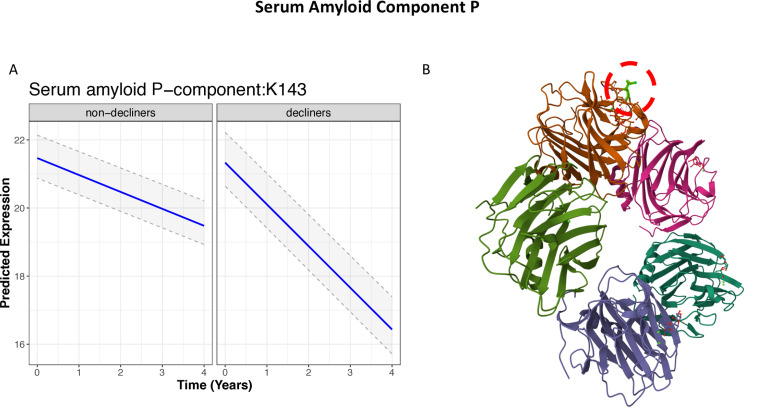
A) Expression of the glycated K143 peptide from serum amyloid component P (SAMP) and B) 3D structure of SAMP (https://www.rcsb.org/3d-sequence/1GYK?asymId=B). Highlighted in dashed red circle is the location of K143, which bares the glycation.

The most significant finding in the ‘rate of change’ analysis, comparing the two groups, was a glycated glycopeptiform from serum amyloid component P (SAMP), at K143 (p < 001). This glycopeptiform did not differ between the two groups at baseline but was strongly downregulated over time in the decliners (see **Fig 6A**). We note this protein particularly due to its relevance to Alzheimer’s disease (see the Discussion). Looking at the 3D structure of SAMP (https://www.rcsb.org/3d-sequence/1GYK?asymId=B), the location of K143 is on the outer region of this protein, which makes sense since it is a modification caused by prolonged exposure to sugar (**Fig 6B**).

### Potential biomarkers for cognitive decline

In addition to describing longitudinal changes of glycopeptiform expression in relation to cognitive decline, we also explored potential biomarkers that could serve as indicators of cognitive decline in its earliest stages, before symptoms become evident. Given that both study groups exhibited normal cognitive function at the beginning of the study, discrepancies in expression of a glycopeptiform at time point 1 may potentially serve as an early indicator of incipient cognitive decline. However, for a true biomarker to accurately reflect progression of the disease, it must change concurrently with progression of disease symptoms. Hence, we focused on molecular changes that can both be detected early and which continued to change over time, as represented by the rate of change between time point 1 and 2. **Table 2** presents the 13 glycopeptiforms that overlapped between the differences at time point 1 and the rate of change comparison (see **Fig 2**).

**Table 2:** Significant glycosylated and glycated glycopeptiforms which were differentially expressed both at time point 1 and over time (time point 1 to 2).

Protein accession	Name	Protein site	Glyco type	Glycans	Rate of change Log2(Effect size) (decl./stab.)	Rate of change p value	Time point 1 Fold change (decl./stab.)	Time point 1 p value
P02787	Transferin	K212	Glycation	Hex	0.3	0.011	-2.0	0.007
P00738	Haptoglobin	K141	Glycation	Hex	-2.1	0.033	18.4	0.038
P00738	Haptoglobin	K72	Glycation	Hex	2.0	0.001	-16.0	0.004
P0C0L5	Complement C4-A	K92	Glycation	Hex	1.2	0.039	-2.4	0.007
P02768	Albumin	K499	Glycation	Hex	1.2	0.012	-2.1	0.013
P19827	inter-alpha-trypsin inhibitor	N285	Glycosylation	HexNAc(5)Hex(6)NeuAc(3)	1.3	0.027	-2.9	0.032
P01857	IgG	N180	Glycosylation	HexNAc(4)Hex(5)Fuc(1)	1.5	0.042	-4.6	0.026
P01876	IgA	N144	Glycosylation	HexNAc(3)Hex(4)	-1.3	0.004	3.1	0.025
P01871	IgM	N209	Glycosylation	HexNAc(5)Hex(5)NeuAc(2)	1.9	0.023	-12.0	0.001
Q96PD5	N-acetylmuramoyl-L-alanine amidase	N367	Glycosylation	HexNAc(4)Hex(5)NeuAc(2)	1.5	0.007	-5.4	<0.001
P01019	Angiotensinogen	N38	Glycosylation	HexNAc(4)Hex(4)NeuAc(1)	1.3	0.001	-3.5	0.002
P00734	Prothrombin	N416	Glycosylation	HexNAc(4)Hex(5)Fuc(1)NeuAc(2)	1.1	0.042	-2.8	0.030

To visualize the differential expression of the significant glycopeptiforms, we included box plots showing the expression at time point 1 and the difference in rate of change for decliners compared to non-decliners (S11 File). Eight glycopeptiforms showed lower expression at time point 1 for the non-decliners and decreased over time, while the decliners remained constant or increased. These included glycosylation and glycation on inter alpha trypsin inhibitor, transferrin, albumin, IgM, IgG, prothrombin, angiotensinogen and N-acetylmurancyl L-alanine amidase.

**Fig 7:**
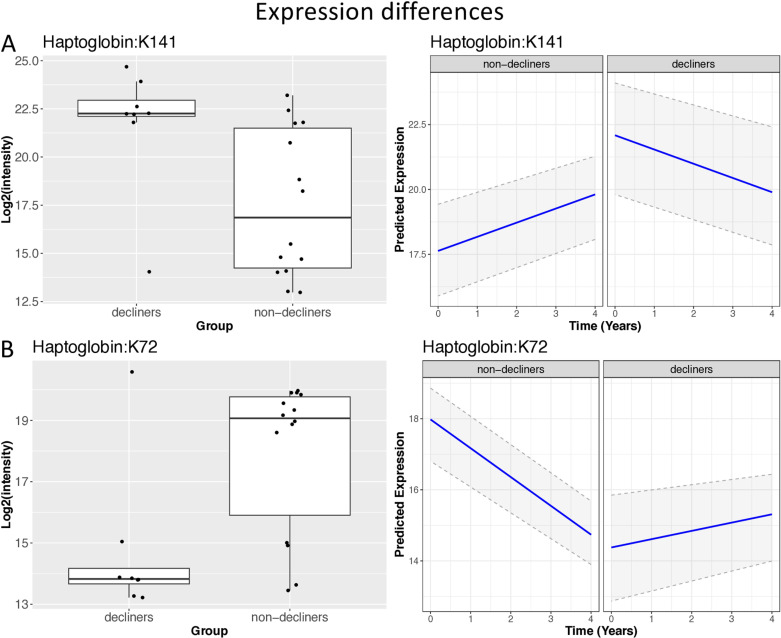
Box plots and plots of predicted values versus time, with prediction confidence intervals based on the glycopeptiform intensities for the two significant glycopeptiforms of haptoglobin, that were significantly altered both at time point 1 and in the rate of change when comparing cognitive decliners versus non-decliners. The regression plot shows the predicted values of an average person with regard to age and HbA1c. We used median values from our sample for these predictors and a confidence interval of 95%. Predicted values and their confidence intervals were obtained from the linear mixed effects model.

Conversely, three glycopeptiforms presented the opposite patters. One protein, haptoglobin, stood out, with two significant glycopeptiforms, showing opposite patterns of expression (**Fig 7**). K141 (glycation) on haptoglobin was higher in decliners at time point 1, and decreased over time, while non-decliners had lower expression levels at time point 1 and then its expression increased over time. The pattern of expression for K72 was the opposite (**Fig 7**).

### Role of glycopeptiforms beyond protein expression level

Focusing on the thirteen glycopeptiforms listed in **Table 2**, we examined all the other glycopeptiforms from each of the proteins in the table, and generated bar graphs showing the differential expression of all glycopeptiforms from the respective protein (S12 File). In all cases, there was no systematic pattern of glycopeptiform changes, suggesting that the significant changes are primarily unrelated to variations in overall protein expression.

## Discussion

Our study utilized a novel glycoproteomic pipeline [29] to analyze site-specific glycosylation, as well as glycation, in 44 serum samples from 22 initially cognitively normal individuals with T2D, eight of whom declined over time and developed either mild cognitive impairment or frank dementia. We focused on glycopeptiforms, which refer to specific glycan compositions on specific amino acid sites of proteins. The novelty of the approach includes our glycopeptide enrichment method, a target-decoy approach for both peptide sequence and glycans and the combined Pscore based on the Byonic LogProb and delta-mod scores for calculation of the false discovery rate. The rationale behind this was our observation that the recommended filtering criteria of LogProb < 2 was not stringent enough.

This workflow enabled identification and quantification glycosylated glycopeptiforms associated with cognitive impairment. Furthermore, our unique enrichment method [29] enabled characterization of serum protein glycation—a major driver of T2D pathologies [51,52], to gain a more comprehensive understanding of the molecular alterations associated with cognitive decline in older adults with T2D. Indeed, our glycoproteomic analysis revealed quantitative differences in glycopeptiforms between participants whose cognition declined over time and those whose cognition remained normal. At baseline, all participants had normal cognition. However, already in this “asymptomatic” phase, 35 glycopeptiforms were uniquely different in decliners compared to non-decliners. After an average of 52 months, 148 glycopeptiforms had a significantly different pattern of change over time between the groupssuggesting that they may reflect progression of pathological processes contributing to cognitive decline.

While 44% of all glycopeptiforms were glycated, a higher percentage was associated with cognitive decline. Specifically, 60% of glycated glycopeptiforms showed differential expression between the groups at time point 1, and 72% showed different patterns of change in expression over time in the two groups This suggests that, in the presence of cognitive impairment, there is a marked prevalence of glycation processes in the glycoproteoform landscape.

To gain further insights into the potential roles of glycation and glycosylation in cognition, pathway analysis was conducted. Among the glycopeptiforms significantly associated with cognition, downregulation of glycation was associated primarily with metabolism. Conversely, upregulation of glycosylation was associated mainly with immune response. These findings are in line with a wealth of evidence highlighting the involvement of metabolic derangements and the immune systems in cognitive decline and dementia, especially in the context of T2D [53–56]. Moreover, these results underscore the importance of investigating glycation and glycosylation separately when studying cognitive decline, as their roles and contributions may vary throughout the progression of the disease. It also highlights the need for more research to understand the underlying mechanisms driving these distinct associations and how they evolve over time. Replication in larger samples and further studies examining the specific molecular pathways involved in glycosylation and glycation and their interactions with cognitive trajectories will be essential to unravel the complexities and potential therapeutic implications of our findings.

The damaging effects of advanced glycation end-products (AGE) has been recognized for several decades [57], including on the brain and especially in T2D [25,58–60]. The method employed in this study focused on identification of “early glycation products” rather than more complex protein modifications recognized in downstream AGEs, due to technical limitations. Nevertheless, our findings show that even early glycation was associated with various metabolic pathways, reflecting its potential role as a contributing factor to metabolic derangement and subsequent deleterious health outcomes, including cognitive decline.

Over 60% of the proteins detected, were found based on two or more glycopeptiforms, with some proteins having more than 10 variations. These different glycopeptiforms, on the same protein, exhibited distinct associations with cognitive decline. Additionally, our study demonstrated that over 50% of the glycopeptiforms had two or more forms at the same site of a specific protein. This is exemplified by Inter-alpha-trypsin inhibitor heavy chain H1 (P19827). It was detected with 24 glycopeptiforms at time point 1, 15 of whom were glycosylated. These glycans were on two known glycosylation sites: N285 and N588. Each of these sites had multiple glycoforms: 11 and 4 respectively. However, among all these glycosylated peptides, only one was significant when comparing ‘decliners’ versus ‘non-decliners’ at time point 1. The depth and diversity of glycosylation and glycation we detected in serum proteins, underscores that operationalizing a ‘protein’ as one type of molecule is inaccurate, since in fact it has numerous forms, hence the need to relate to proteoforms or glycopeptiforms. Our results suggest that different glyco-proteoforms possess distinct biological functions, yet to be revealed [61,62].

The strongest difference in rate of change over time between the two groups was for the glycation site K143 on the serum amyloid component P (SAMP). The fact that the location of the glycation is on the outer protein envelope is logical because it is caused by long exposure to sugar in the blood stream. The K143 expression over time in the decliners was significantly faster than in non-decliners. This protein is of particular interest since higher neocortical SAMP levels is significantly associated with dementia at death, independent of neuropathological severity [63]. Furthermore, lower cerebrospinal fluid SAMP levels in mild cognitive impairment (MCI) patients were associated with a higher risk of progression to Alzheimer’s Disease [64]. Our findings strengthen this previous observation and may contribute to its associations with cognitive impairment and AD.

A biomarker is a characteristic that can be objectively measured and used as an indicator of disease processes, including prediction, diagnosis and monitoring of disease. For a biomarker to be reliable, it must mirror the disease’s progression, evolving in tandem with the development of disease symptoms. We therefore focused our analyses on the 13 glycoproteoforms exhibiting distinct patterns between decliners and non-decliners, in their expression at baseline as well as over time. These glycoproteoforms may serve as “non-causal” objective indicators of disease progression or as causal biomarkers, underlying biological changes preceding and accompanying the manifestation and progression of overt cognitive symptoms.

Consistent with our results, a study of Japanese older adults supports a glycopeptiform signature characteristic of older adults with T2D with incipient cognitive impairment. In this study [19], they measured approximately 500 N-glycopeptides, derived from 18 proteins. In those who had cognitive impairment, N-glycopeptides with sialylated tri- or tetra-antennary glycans on alpha-2-macroglobulin, clusterin, serum paraoxonase/arylesterase 1, and haptoglobin were less abundant, whereas 3-sialylated tri-antennary N-glycopeptiforms on serotransferrin were more abundant. In contrast to our glycoproteome-wide study, this was a targeted glycoproteome study which focused on 18 proteins, selected based on differential spots on a 2-dimensional gel electrophoresis glycoproteome-wide study [19]. Since the study used HILIC for glycopeptide enrichment, they were not able to measure glycation.

Haptoglobin is a plasma protein that binds to hemoglobin with high affinity and is generated primarily in the liver but also in the brain [65]. The heme iron in hemoglobin is a potent oxidant, and the binding of haptoglobin to hemoglobin decreases the ability of hemoglobin-derived iron from carrying out oxidative reactions [66,67]. In our study, two glycated haptoglobin glycopeptiforms were significantly different in decliners compared to non-decliners both at time point 1 and over time. Interestingly, the two peptides changed in opposite directions, i.e., one was upregulated over time, while the other was downregulated. This is yet another piece of evidence as to the complex proteoform diversity that is often overlooked when discussing protein expression. Our group has shown that the haptoglobin genotype is related to cognitive decline [68] and to hippocampal volume [69] in older adults with T2D. However, the levels of expression of the haptoglobin protein were not associated with cognitive decline [68], suggesting that glycosylation and glycation of the haptoglobin protein, rather than the protein expression itself, may serve as the functional link. Furthermore, our prior findings relating haptoglobin to cognitive decline focused on Jewish [70] and African American [68] participants; along with the results on Japanese older adults [19], the association of haptoglobin glycosylation with T2D-related cognitive decline may generalize across races and ethnicities.

Our study design, focusing exclusively on individuals with T2D, limits our ability to definitively conclude whether these changes are unique to T2D-related cognitive decline or represent a broader signature of inflammation/ metabolic derangements in cognitive decline. However, it is important to note that glycation and glycosylation processes are intimately linked to the pathophysiology of T2D, playing significant roles in its onset, progression, and complications and their severity [71–76]. The alterations we observed may therefore reflect T2D-specific pathways of cognitive decline, even if they share some features with other conditions. Future studies that include non-T2D control groups are necessary to differentiate T2D-specific glycoproteomic signatures. Furthermore, integrating glycoproteomic data with other biomarkers of inflammation and metabolic dysfunction will help better understand the specificity of these changes to T2D-related cognitive decline.

Limitations of the study primarily relate to the relatively small sample size and follow-up period. Hence, we view this study as exploratory, requiring replication in larger samples. It should be noted that none of the glycopeptiforms withstood FDR adjustment for multiple comparisons. Yet, despite the small size, and using a conservative 2-fold change cutoff for statistical significance, large changes in glycosylation and glycation patterns were observed. Another limitation of our study is the narrow range of CDR scores (0–1) which precluded the use of linear regression with CDR as a continuous variable. Future larger scale studies with a broader range of cognitive functioning will enable modeling of cognitive trajectories and provide a more detailed understanding of the subtle changes in cognition as a function of glycosylation and glycation in T2D. Finally, our glycoproteomics method can only detect glycan composition but not its structure so we cannot distinguish between different structural isomers of glycans with the same composition.

The study has several strengths, including the accurate diagnosis of T2D and of the clinical characteristics of the sample, which were directly measured avoiding reliance on self-reported medical data. To the best of our knowledge, this is the first study to investigate longitudinal changes of cognition with concomitant changes in glycopeptiform expression, in an unbiased manner. The longitudinal design of the study lends credibility to its findings and opens a window for the identification of potential early biomarkers for T2D-related cognitive decline. Furthermore, a multi-disciplinary clinical consensus conference was employed to confirm significant cognitive impairment in participants whose cognition deteriorated over time. Importantly, the two groups were similar in sociodemographic and clinical characteristics at baseline, further suggesting that the associations we found between specific glycopeptiforms and cognitive decline were not confounded by these factors. Our glycoproteomics method has several strengths, notably the crucial ability to identify both glycation and glycosylation forms with the high enrichment efficiency, which leads to high coverage of serum glycopeptiforms. Additionally, the inclusion of a decoy list of glycans and the calculation of the Pscore allowed us to control and verify the true false discovery rate. To ensure robustness, only glycopeptiforms meeting conservative replication criteria were included in the analyses. Taken together we presented a robust workflow for glycoproteomics. While HbA1c levels did not differ significantly between decliners and non-decliners in our study, this does not diminish the established link between T2D and cognitive impairment. By matching groups on HbA1c, our study design allowed for the investigation of specific glycopeptiforms as potential mechanisms underlying cognitive decline in T2D. This approach suggests that other T2D-related factors, particularly glycation and glycosylation processes, may affect cognitive outcomes independently of glycemic control.

In conclusion, our study provides evidence that site-specific glycoproteomic modifications are linked to cognitive decline in older adults with T2D, representing an advancement in understanding of the molecular mechanisms underlying cognitive decline in this high-risk population. The identification and quantification of specific glycopeptiforms associated with cognitive decline not only enhance our knowledge of the role of metabolic dysregulation in cognitive impairment but also highlight the potential of these glycopeptiforms as novel biomarkers for early detection and monitoring. Furthermore, by identifying specific glycoproteomic modifications, our research opens new avenues for targeted therapeutic interventions, paving the way for personalized medicine approaches that align with preventive health strategies. Future research should focus on validating these findings through large-scale longitudinal studies, exploring the functional consequences of glycosylation changes, and ultimately developing interventions aimed at these pathways. While initial in scope, our work contributes to the broader goal of improving cognitive health in T2D patients and reducing the burden of dementia in this vulnerable population.

## Supporting information

S1 File A table of the samples included in the study. It includes the demographics information and link to the raw data file names.
(XLSX)

S2 File LC-MS chromatograms of the 44 samples. (PDF)

S3 File Protein sequence database used to search the data. (TXT)

S4 File Glycan database, include the decoy gycans. (TXT)

S5 File A plot showing the target and decoy PSMs based on the Pscore. (PDF)

S6 File The filtered, formatted list of PSMs for input into FlashLFQ. (TXT)

S7 File The final data matrix, including statistics, split into T1, T2 and the ratio of T2/T1. (XLSX)

S8 File A plot showing the intensity values, before and after normalization. (PDF)

S9 File Principal component analysis. (PDF)

S10 File Annotated MS/MS spectra for all the statistically significant glycopeptiforms. (PDF)

S11 File Box plots and prediction plots for the 13 significant glycopeptiforms. (PDF)

S12 File Bar graphs of all glycopeptiforms from the significant proteins. (PDF)
