# Supplementary figures and images for: Cognitive decline in older adults with type 2 diabetes: Unraveling site-specific glycoproteomic alterations
Source: PLoS One. 2025 May 8;20(5):e0318916. doi: 10.1371/journal.pone.0318916 (PMC12061096; doi:10.1371/journal.pone.0318916)

RT: 0.00 - 178.00

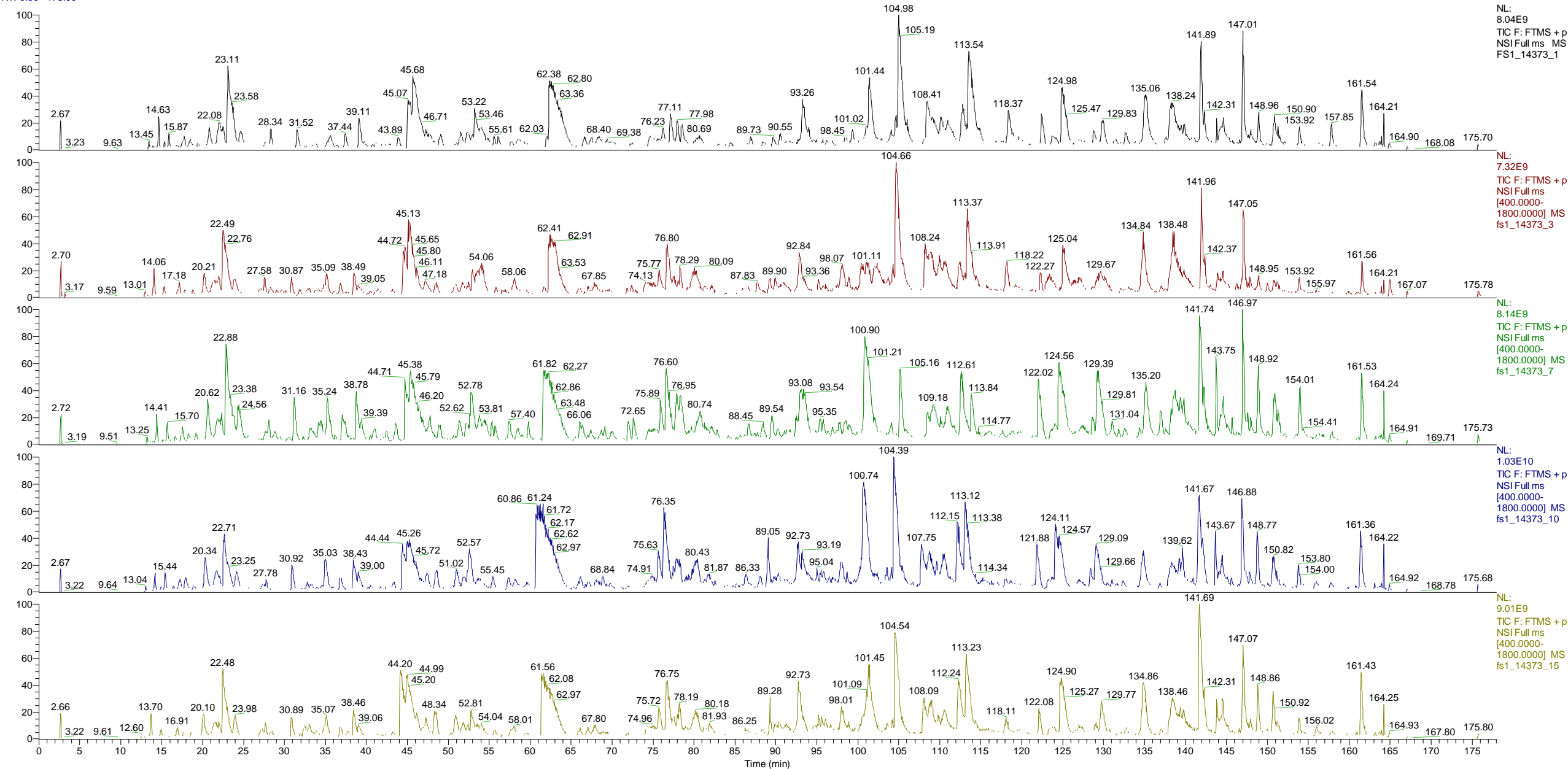

RT: 0.00 - 178.00

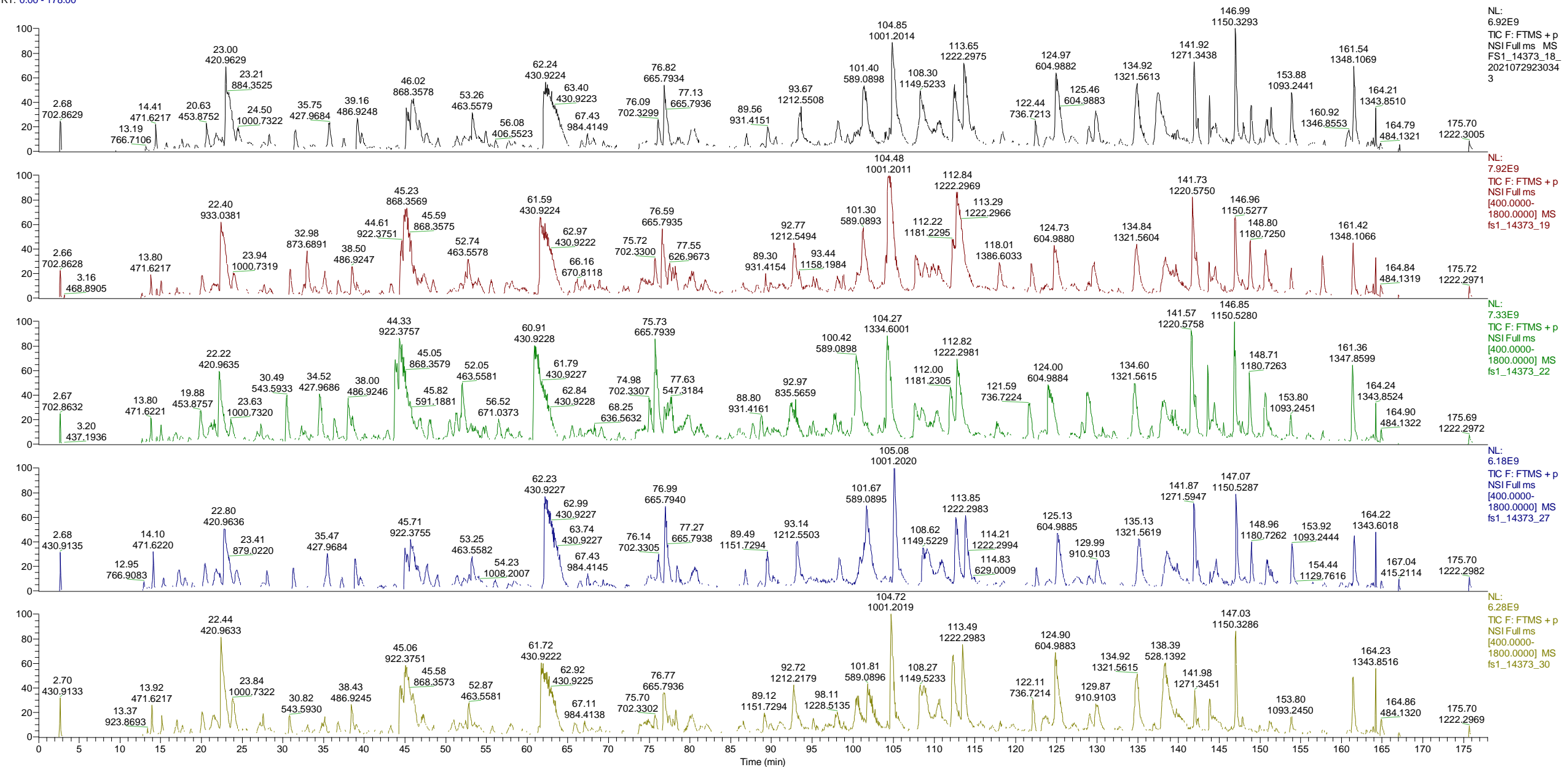

RT: 0.00 - 178.00

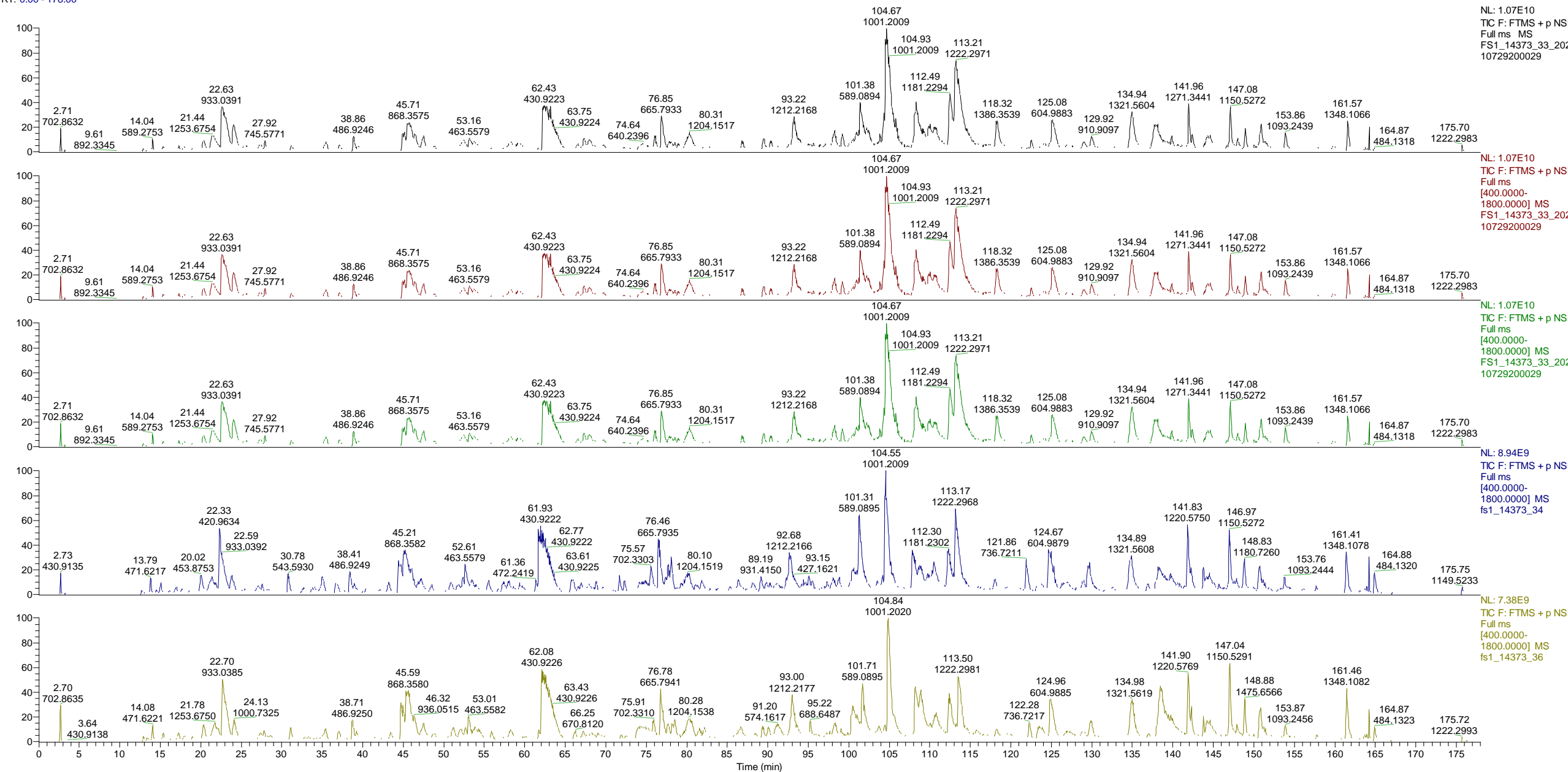

RT: 0.00 - 178.00

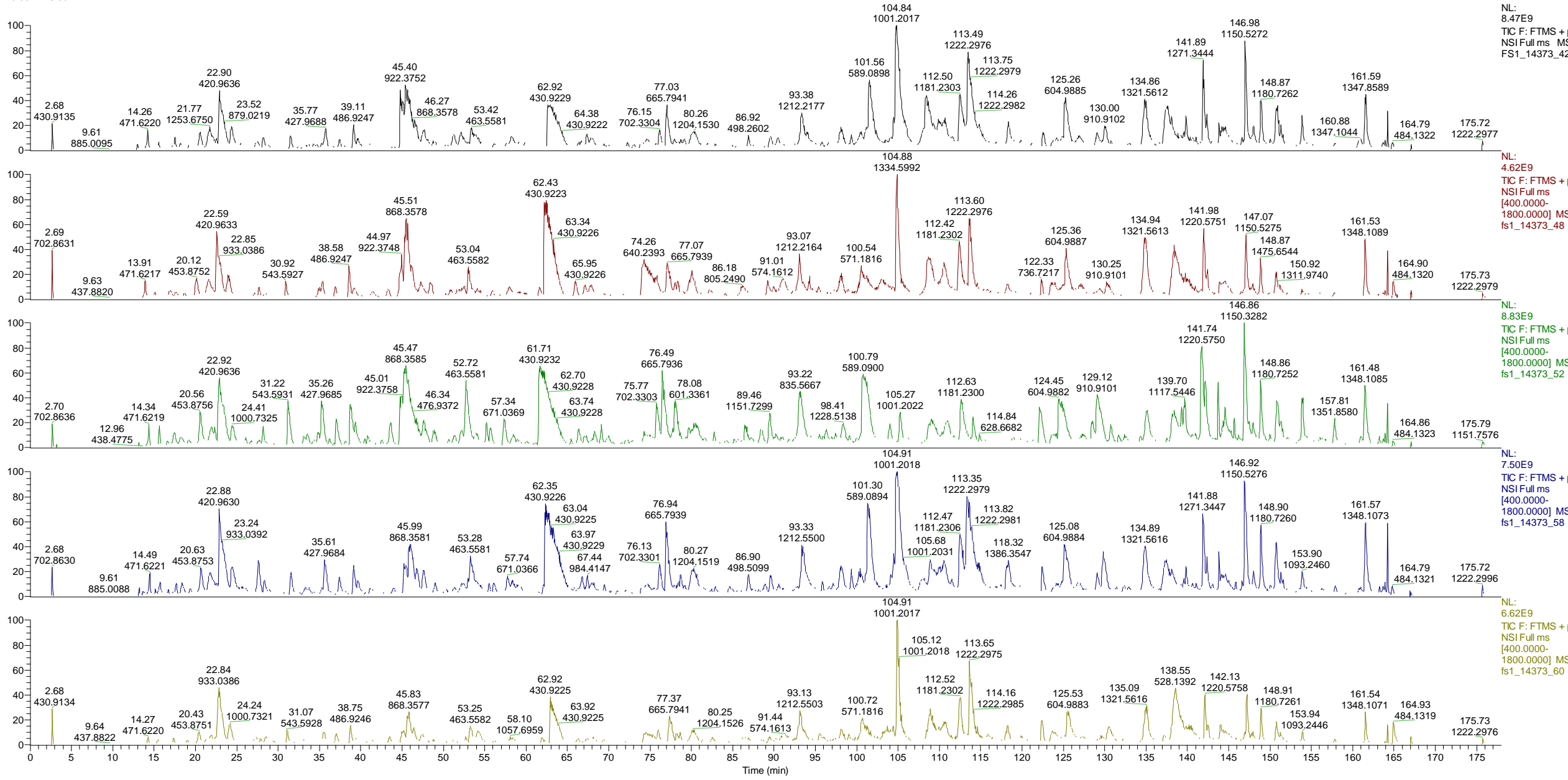

RT: 0.00 - 178.00

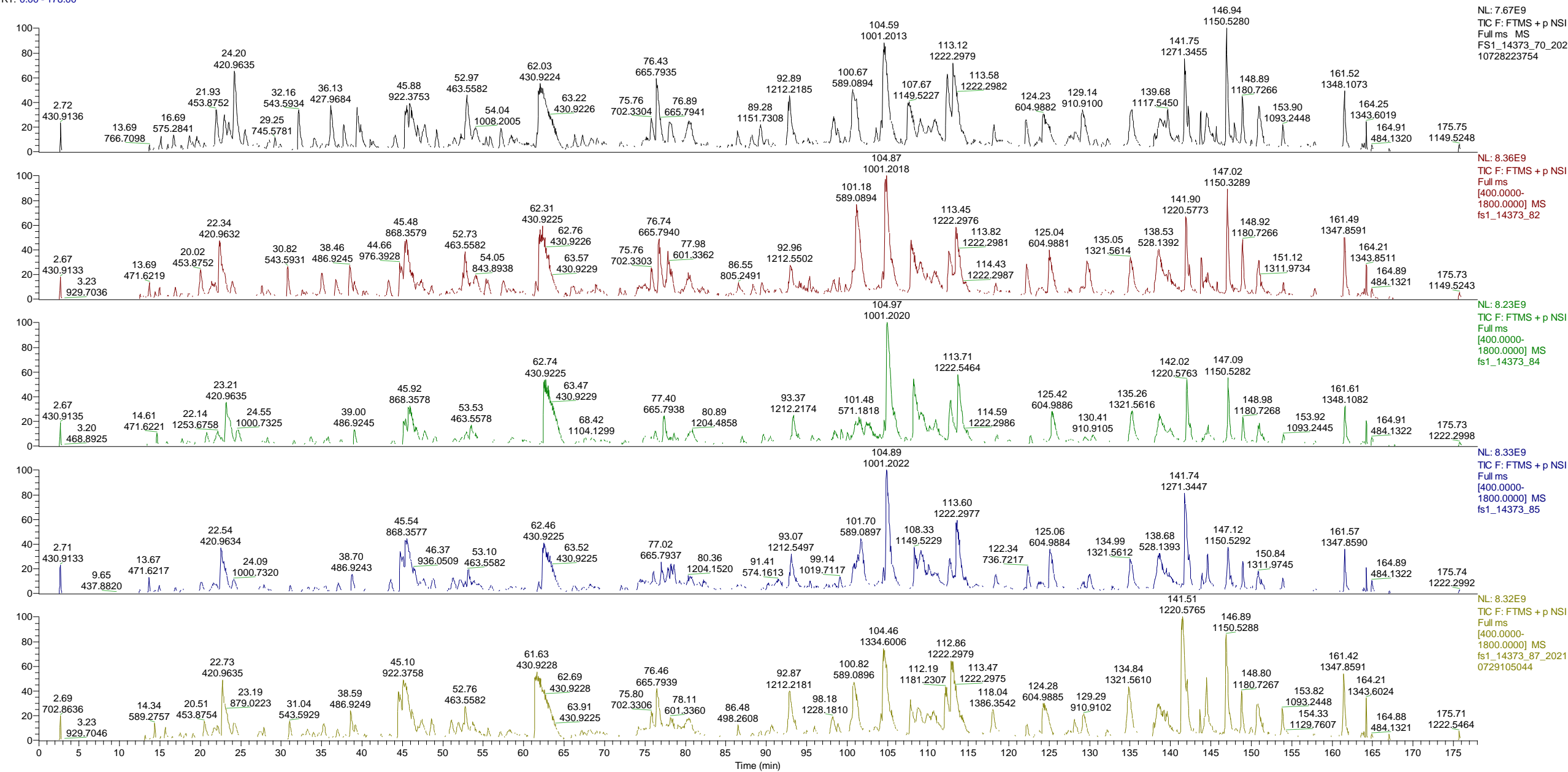

RT: 0.00 - 178.00

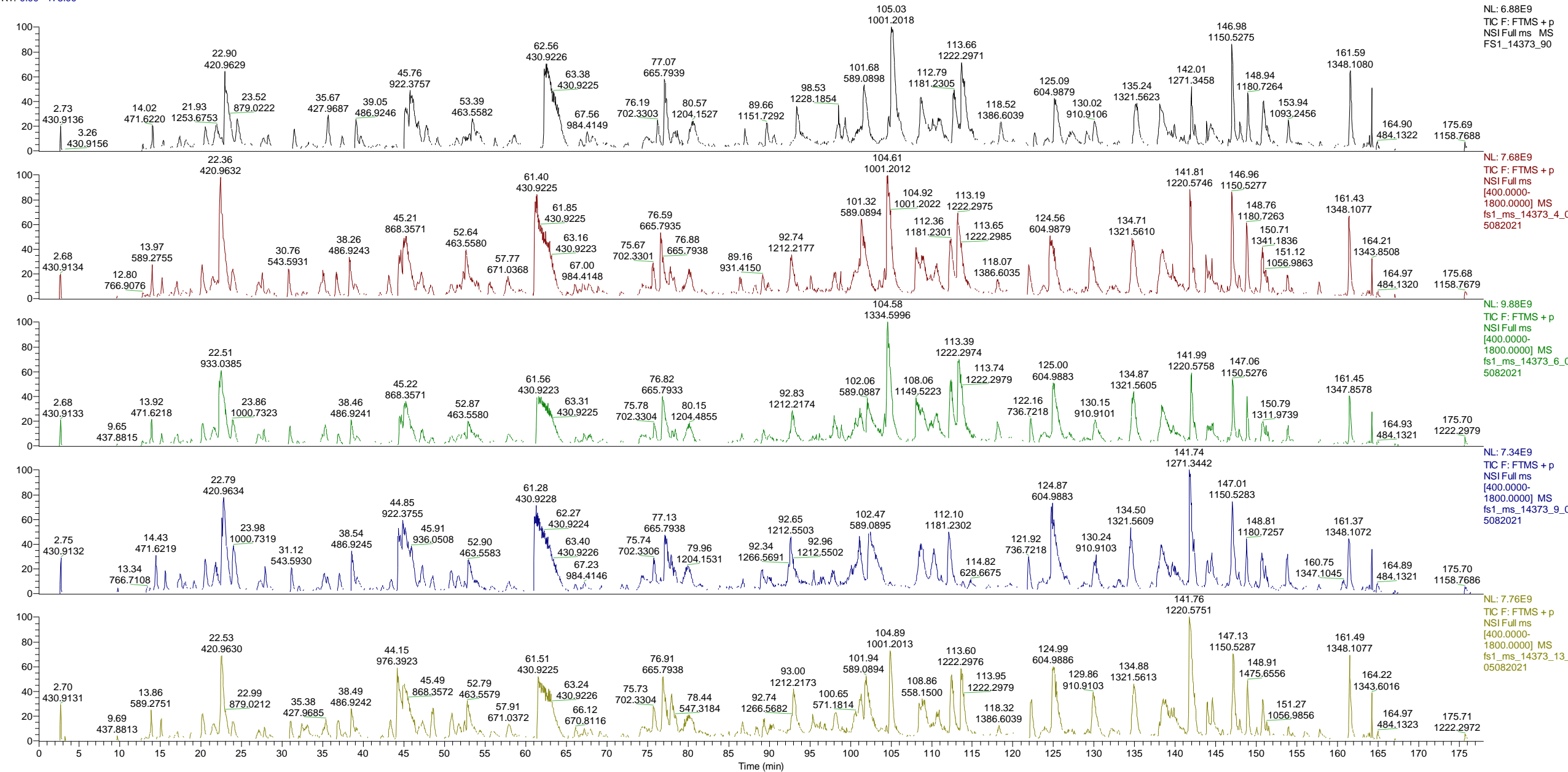

RT: 0.00 - 178.00

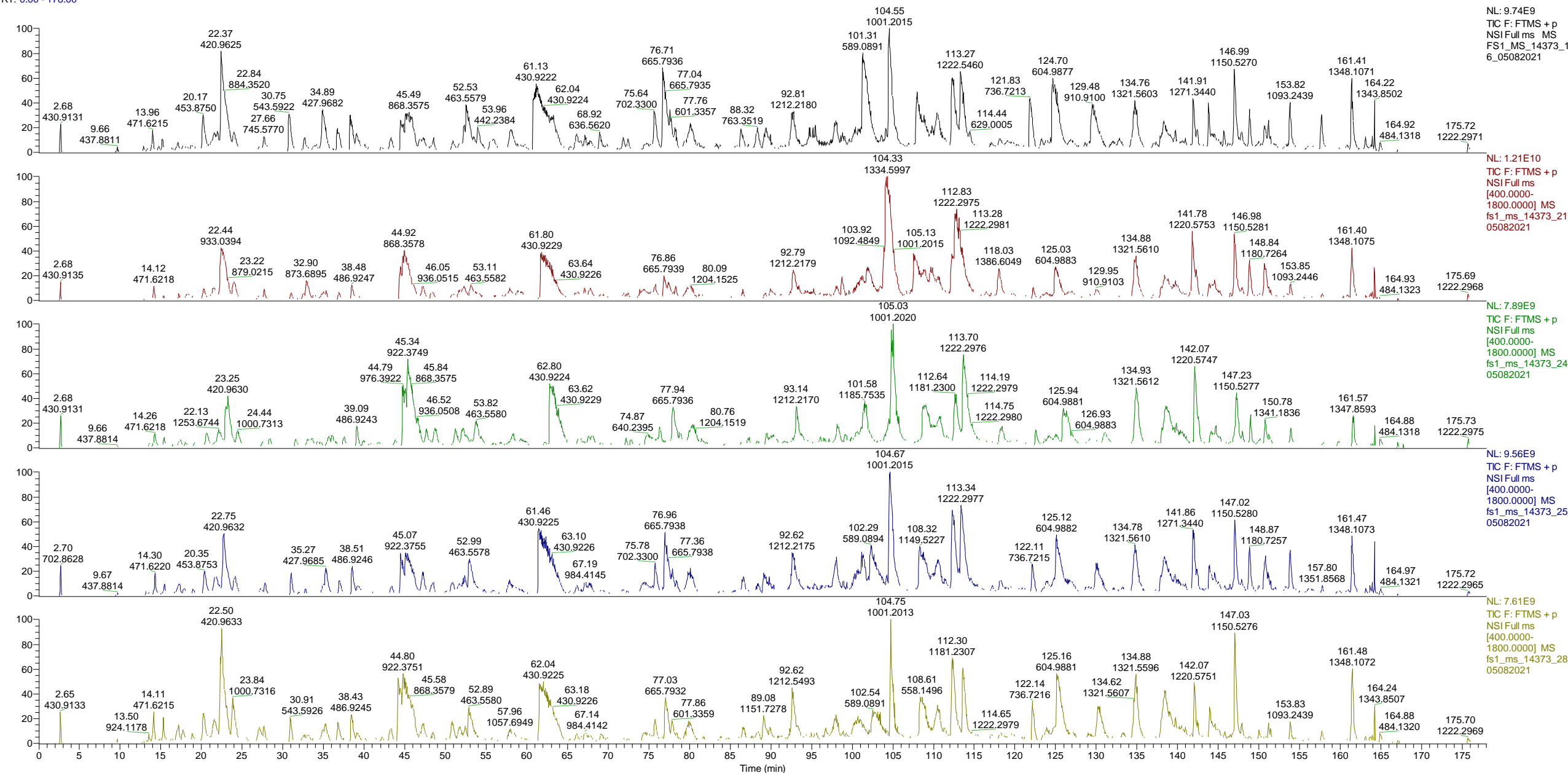

RT: 0.00 - 178.00

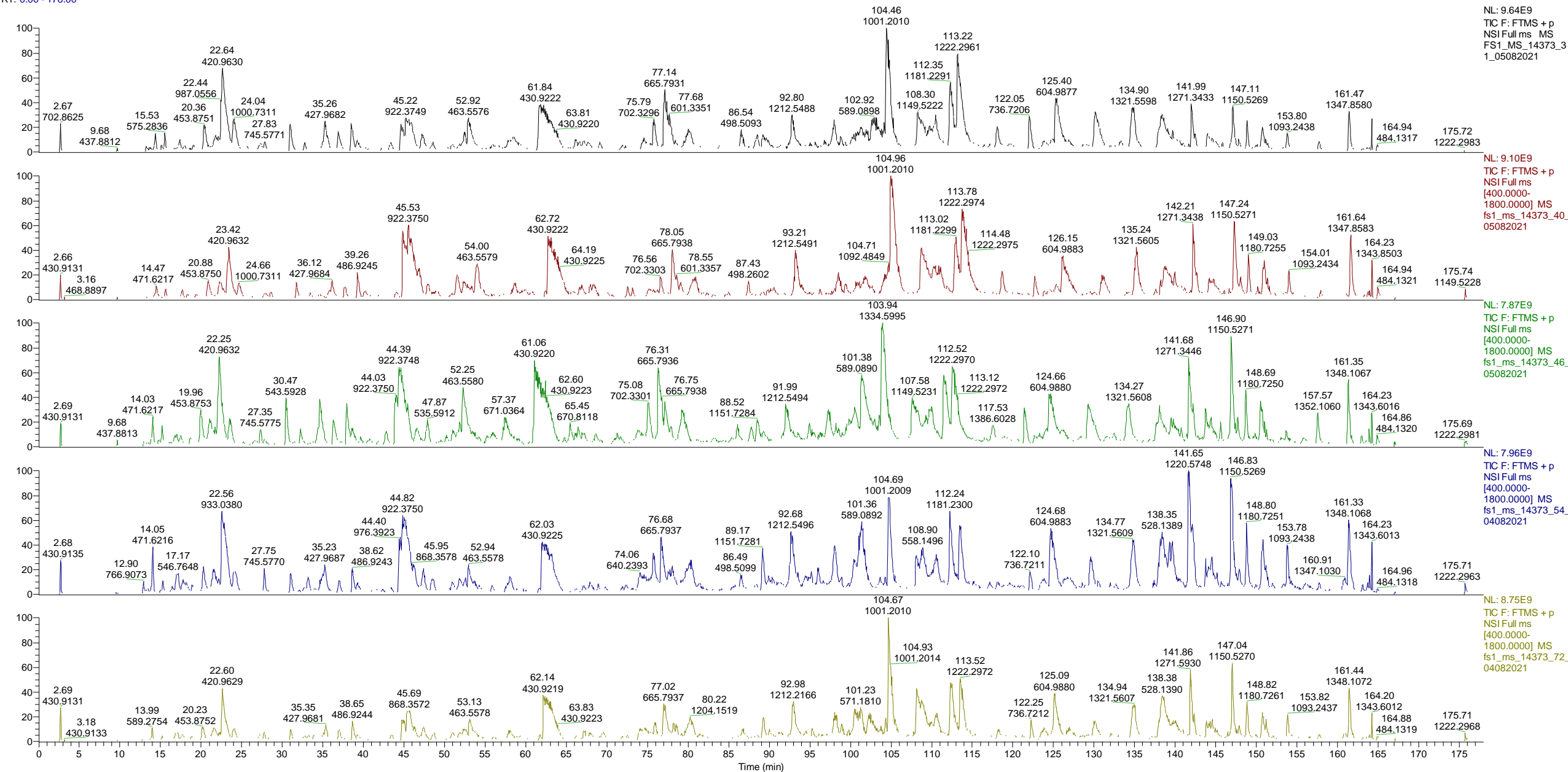

RT: 0.00 - 178.00

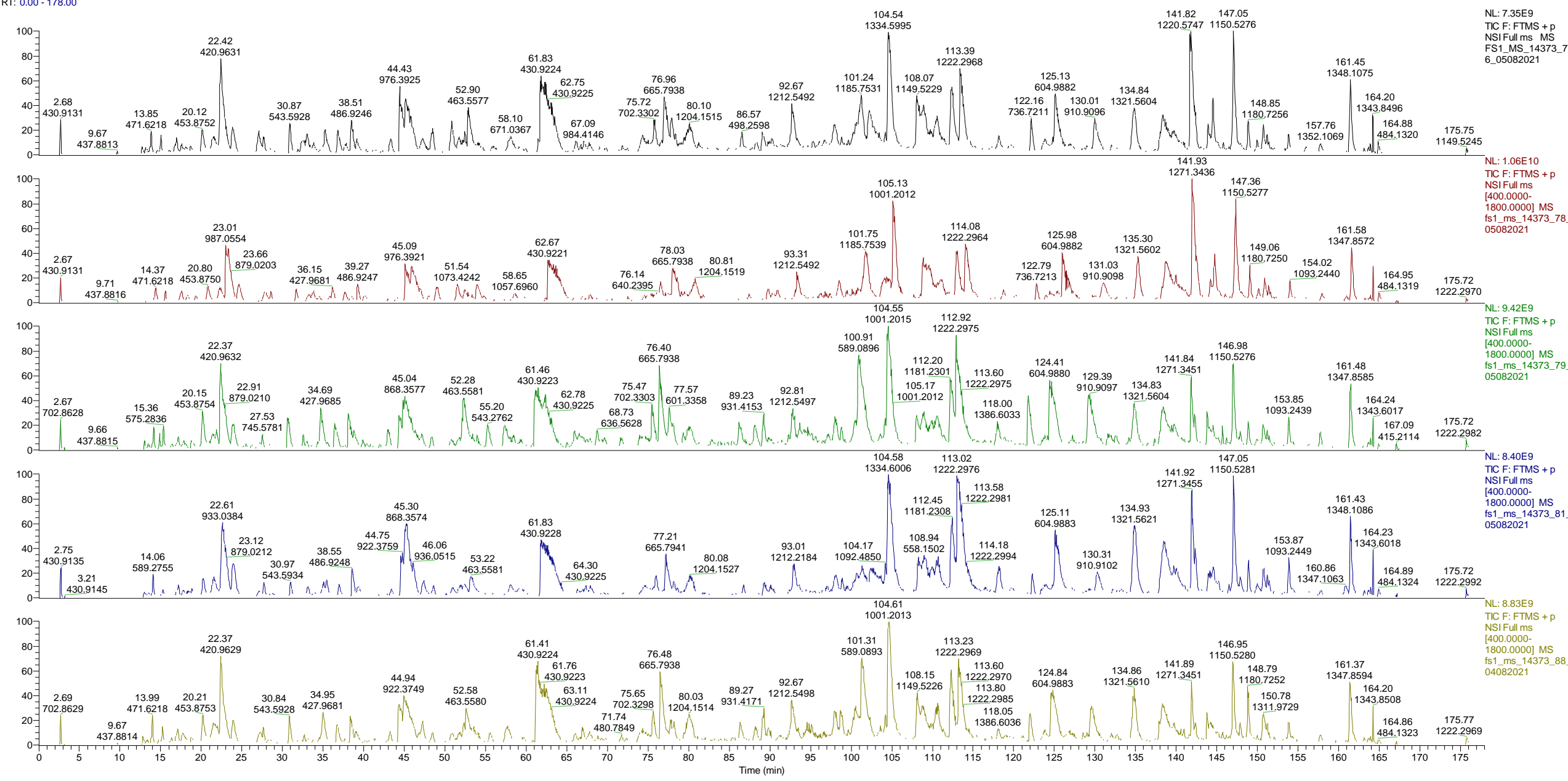

Supplement: S2 File — (PDF) [file pone.0318916.s002.pdf]

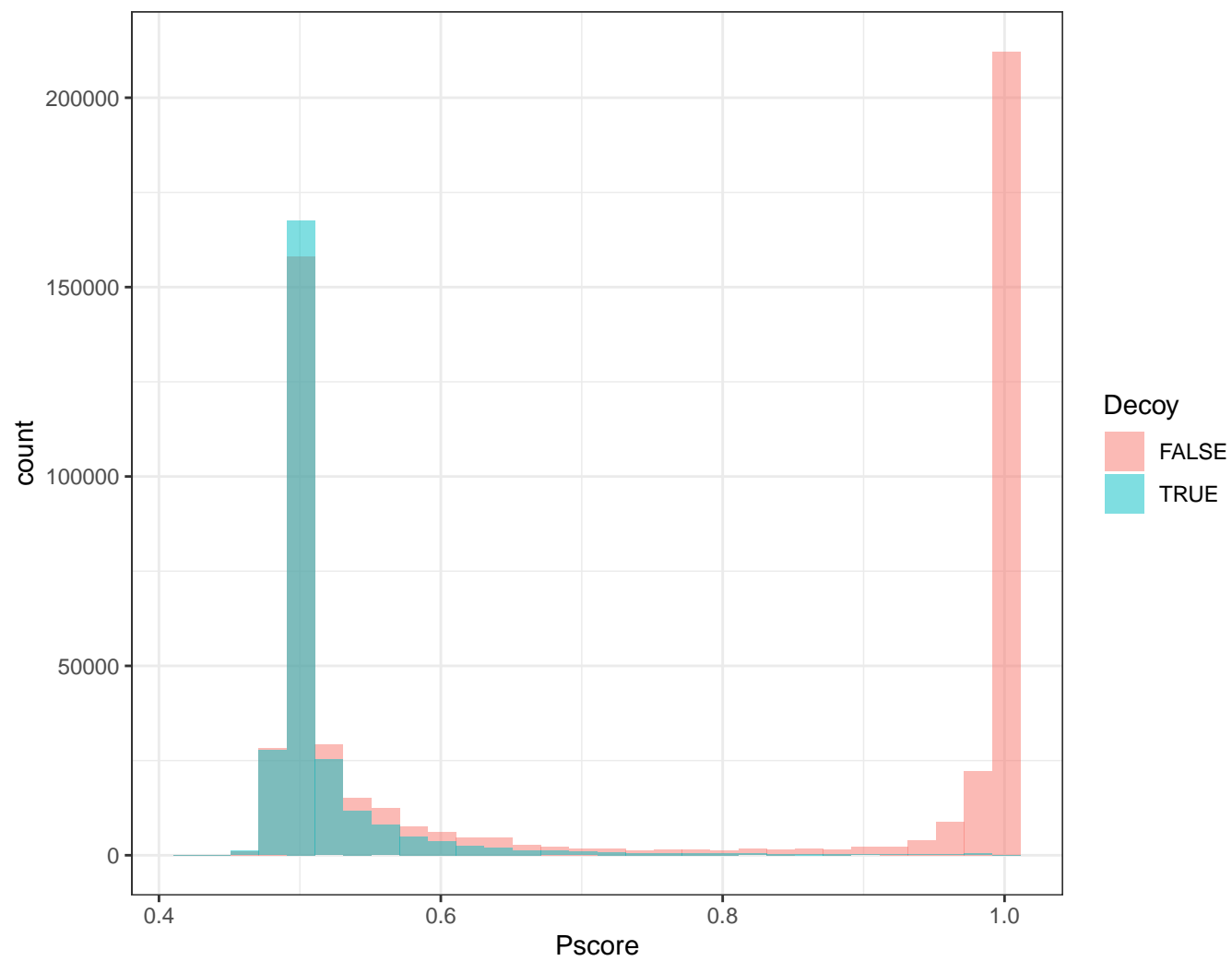

Supplement: S5 File — (PDF) [file pone.0318916.s005.pdf]

# Distribution of $\log_2(I)$ between LFQ sessions

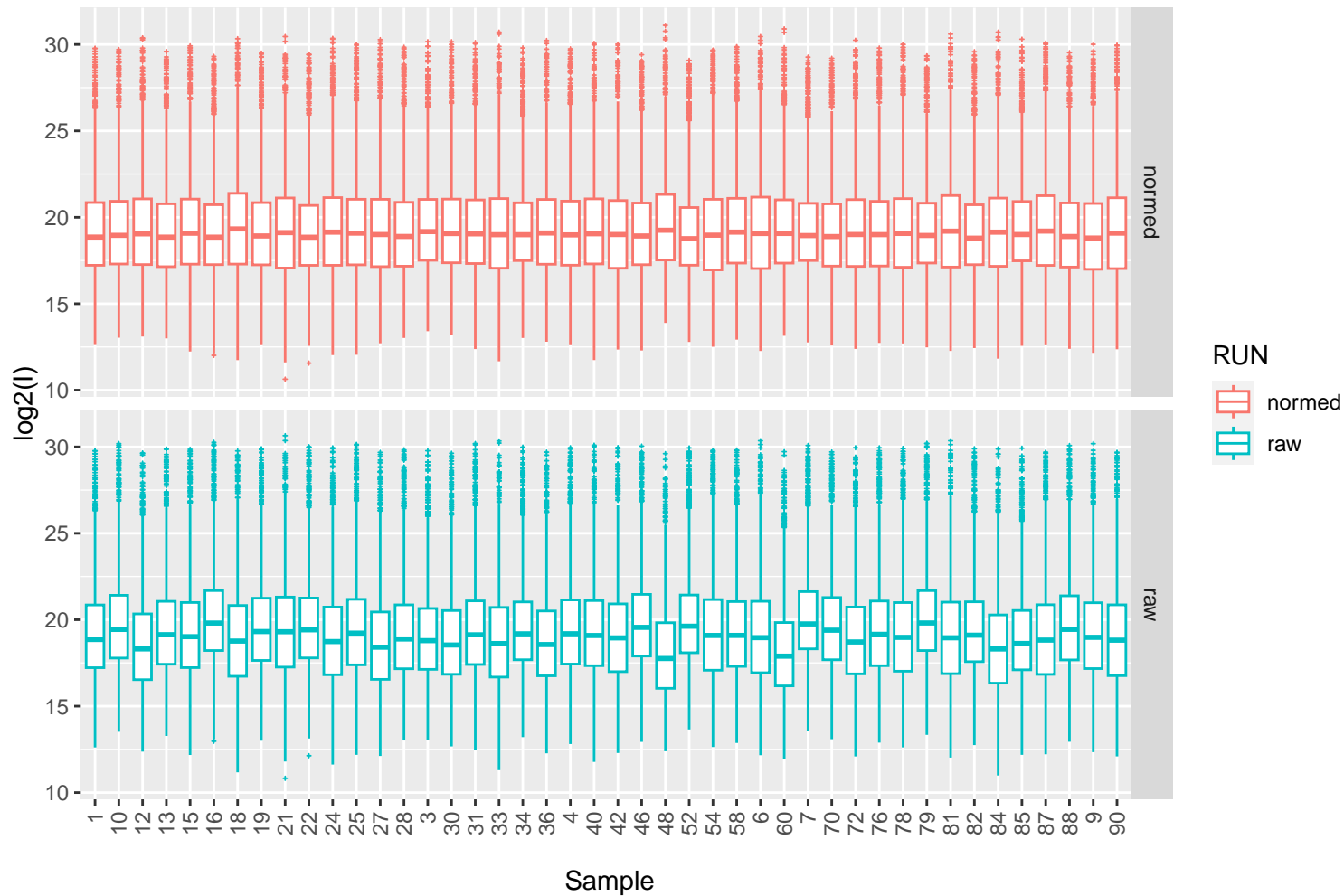

Supplement: S8 File — (PDF) [file pone.0318916.s008.pdf]

PC2 (12%)

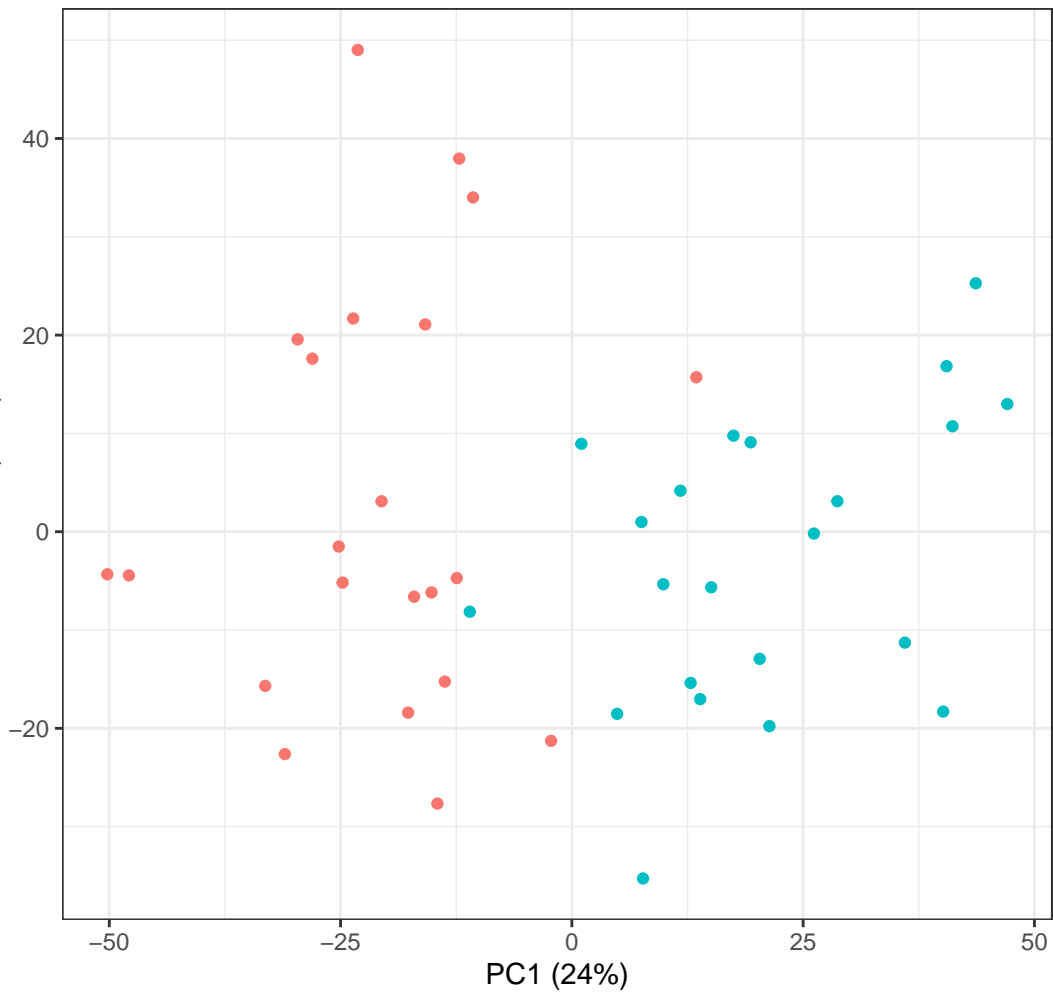

Supplement: S9 File — (PDF) [file pone.0318916.s009.pdf]
