# Supplementary material for: Cognitive decline in older adults with type 2 diabetes: Unraveling site-specific glycoproteomic alterations
Source: PLoS One. 2025 May 8;20(5):e0318916. doi: 10.1371/journal.pone.0318916 (PMC12061096; doi:10.1371/journal.pone.0318916)
Supplement: S10 File — (PDF) [file pone.0318916.s010.pdf]

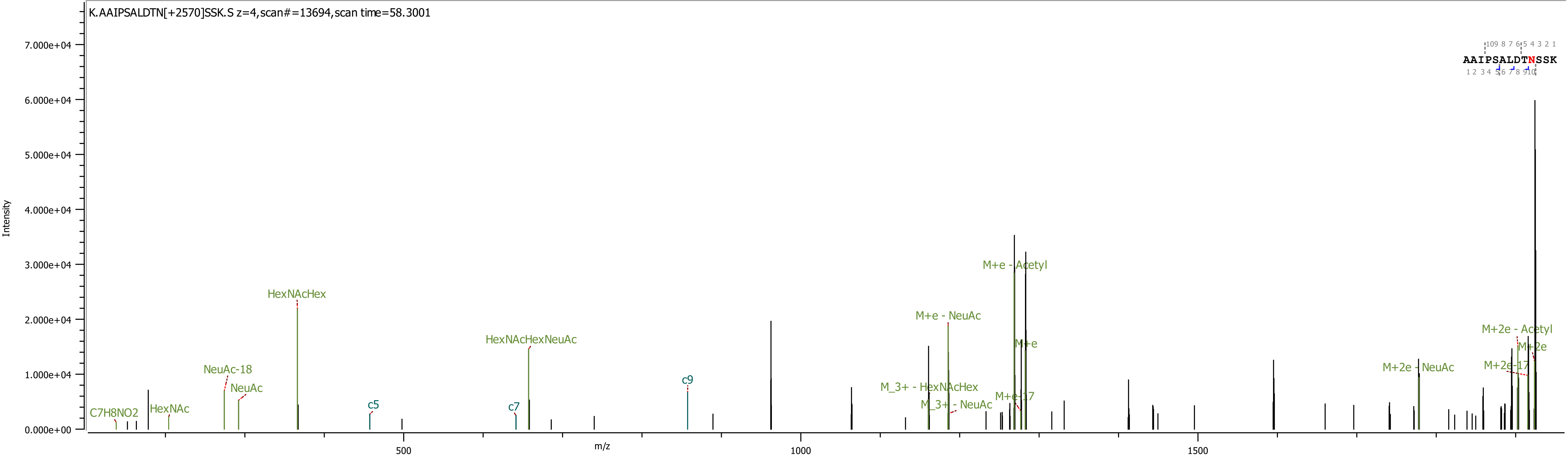

K.AAIPSALDTN[+2570]SSK.S z=4,scan#=13694,scan time=58.3001
Intensity
m/z
AAIPSALDTNSSK
C7H8NO2
HexNAc
NeuAc-18
NeuAc
HexNAcHex
c5
c7
HexNAcHexNeuAc
c9
M_3+ - HexNAcHex
M_3+ - NeuAc
M+e - NeuAc
M+e - Acetyl
M+e
M+e-17
M+2e - NeuAc
M+2e - Acetyl
M+2e
M+2e-17

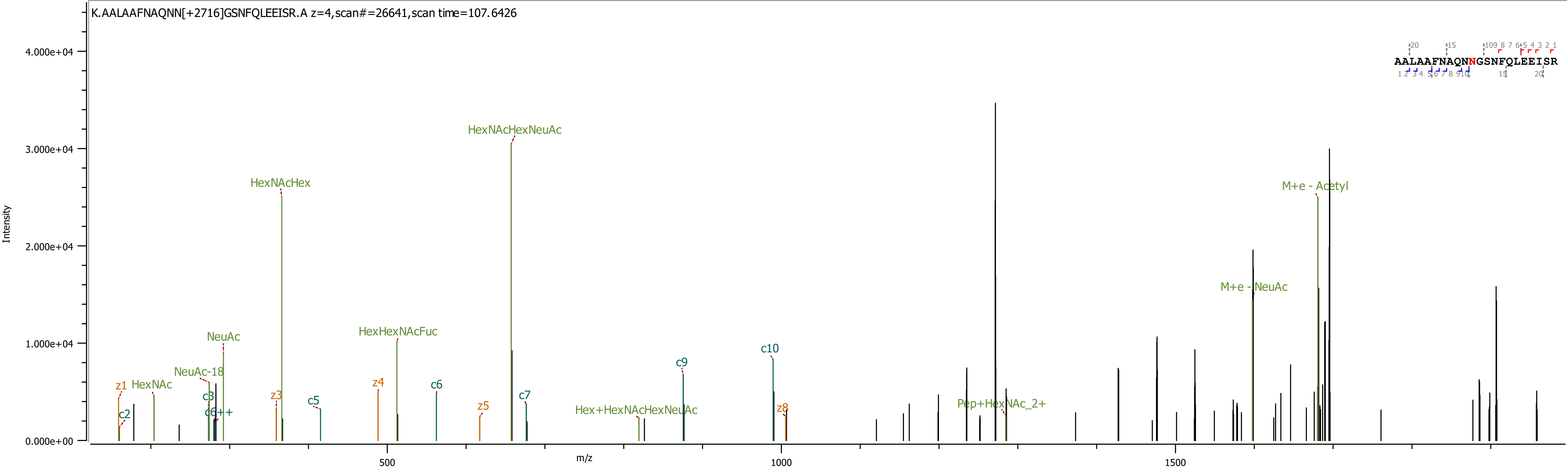

K.AALAAFNAQNN[+2716]GSNFQLEEISR.A z=4,scan#=26641,scan time=107.6426
AALAAFNAQNNGSNFQLEEISR
Intensity
m/z
4.000e+04
3.000e+04
2.000e+04
1.000e+04
0.000e+00
500
1000
1500
z1
c2
HexNAc
NeuAc-18
c3
c6++
NeuAc
HexNAcHex
z3
c5
z4
HexHexNAcFuc
c6
z5
HexNAcHexNeuAc
c7
Hex+HexNAcHexNeuAc
c9
c10
z8
Pep+HexNAc_2+
M+e - NeuAc
M+e - Acetyl

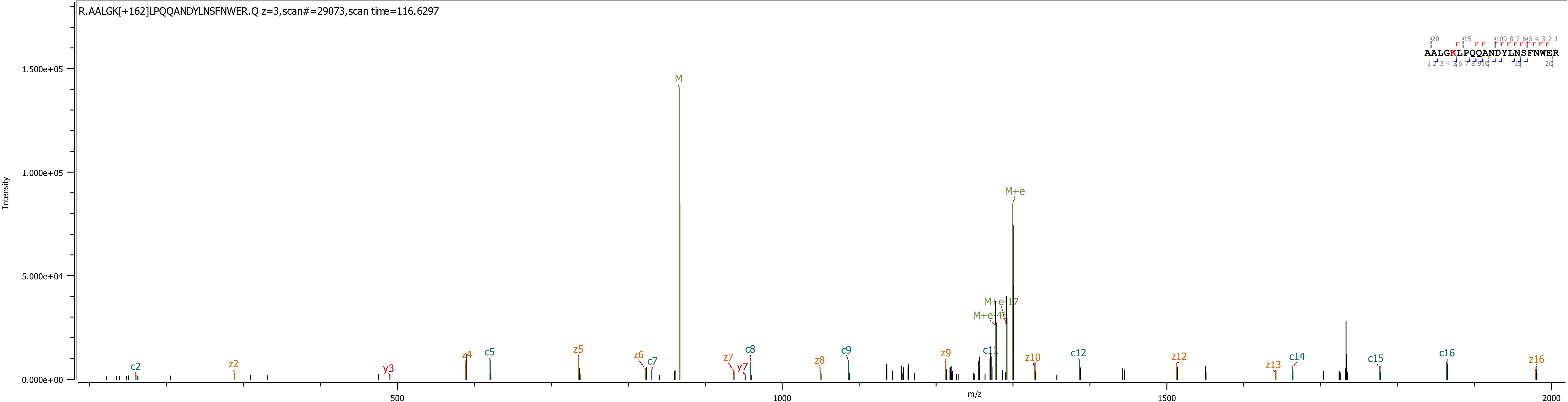

R.AALGK[+162]LPQQANDYLNSFNWER.Q z=3,scan#=29073,scan time=116.6297
AALGKLPQQANDYLNSFNWER
Intensity
m/z
1.500e+05
1.000e+05
5.000e+04
0.000e+00
500
1000
1500
2000
M
M+e
M+e-17
M+e-45
c2
z2
y3
z4
c5
z5
z6
c7
z7
y7
c8
z8
c9
z9
c11
z10
c12
z12
z13
c14
c15
c16
z16

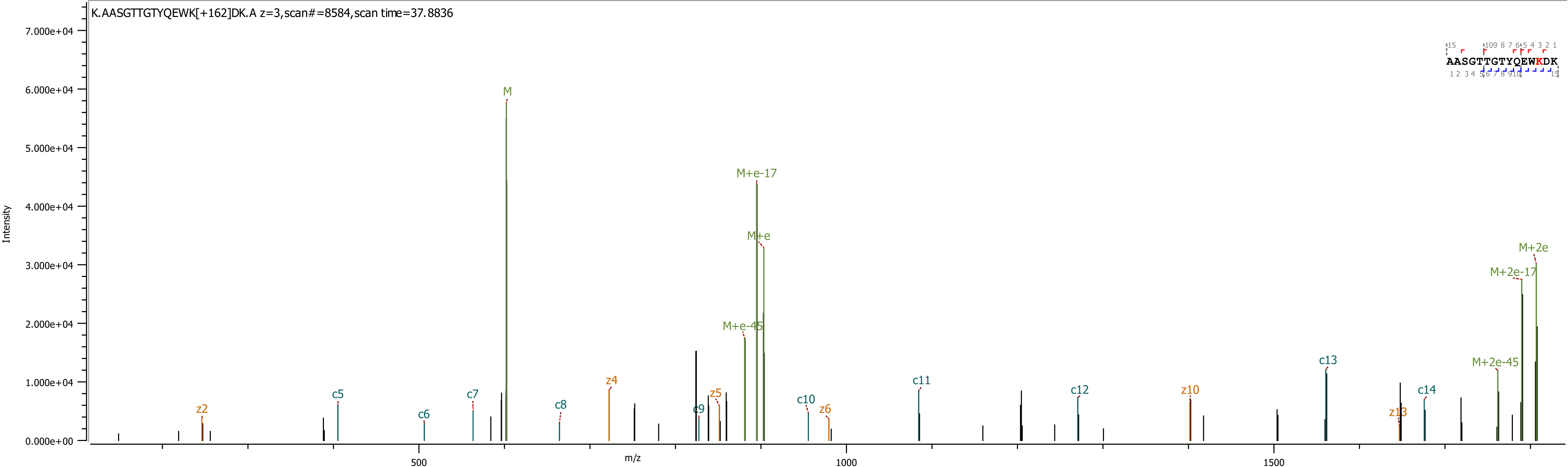

K.AASGTTGTYQEWK[+162]DK.A z=3,scan#=8584,scan time=37.8836
Intensity
m/z
7.000e+04
6.000e+04
5.000e+04
4.000e+04
3.000e+04
2.000e+04
1.000e+04
0.000e+00
500
1000
1500
M
M+e-17
M+e
M+e-45
M+2e
M+2e-17
M+2e-45
z2
c5
c6
c7
c8
z4
c9
z5
c10
z6
c11
c12
z10
c13
z13
c14
AASGTTGTYQEWKDK

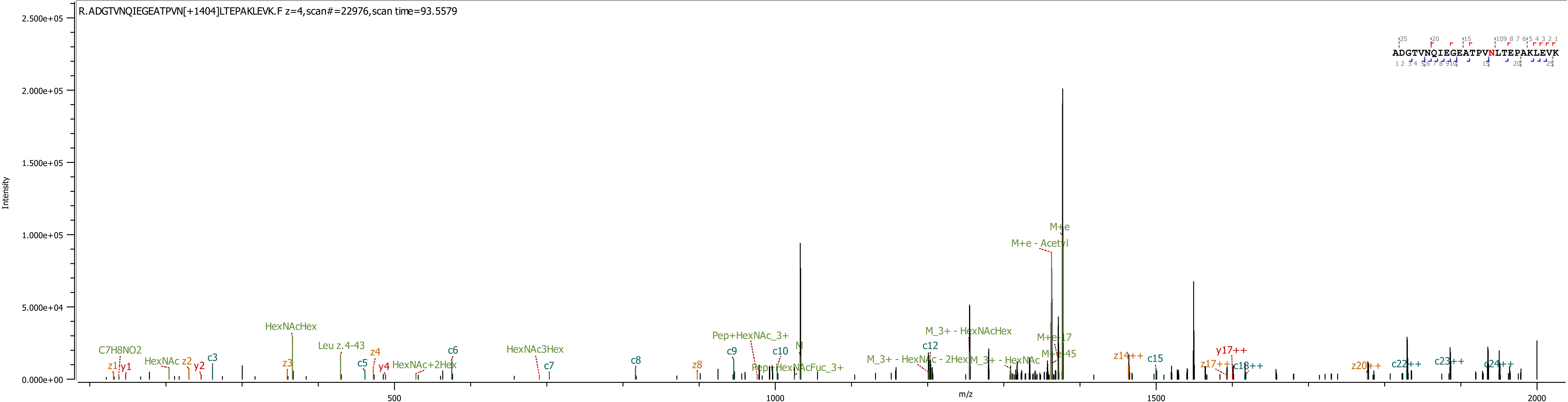
R.ADGTVNQIEGEATPVN[+1404]LTEPAKLEVK.F z=4,scan#=22976,scan time=93.5579
ADGTVNQIEGEATPVNLTEPAKLEVK
Intensity
2.500e+05
2.000e+05
1.500e+05
1.000e+05
5.000e+04
0.000e+00
m/z
500
1000
1500
2000
C7H8NO2
z1
y1
HexNAc
z2
y2
c3
HexNAcHex
z3
Leu z.4-43
c5
z4
y4
HexNAc+2Hex
c6
HexNAc3Hex
c7
c8
z8
c9
Pep+HexNAc_3+
c10
M
Pep+HexNAcFuc_3+
c12
M_3+ - HexNAcHex
M_3+ - HexNAc - 2Hex
M_3+ - HexNAc
M+e
M+e - Acetyl
M+e-17
M+e-45
z14++
c15
y17++
z17++
c18++
z20++
c22++
c23++
c24++

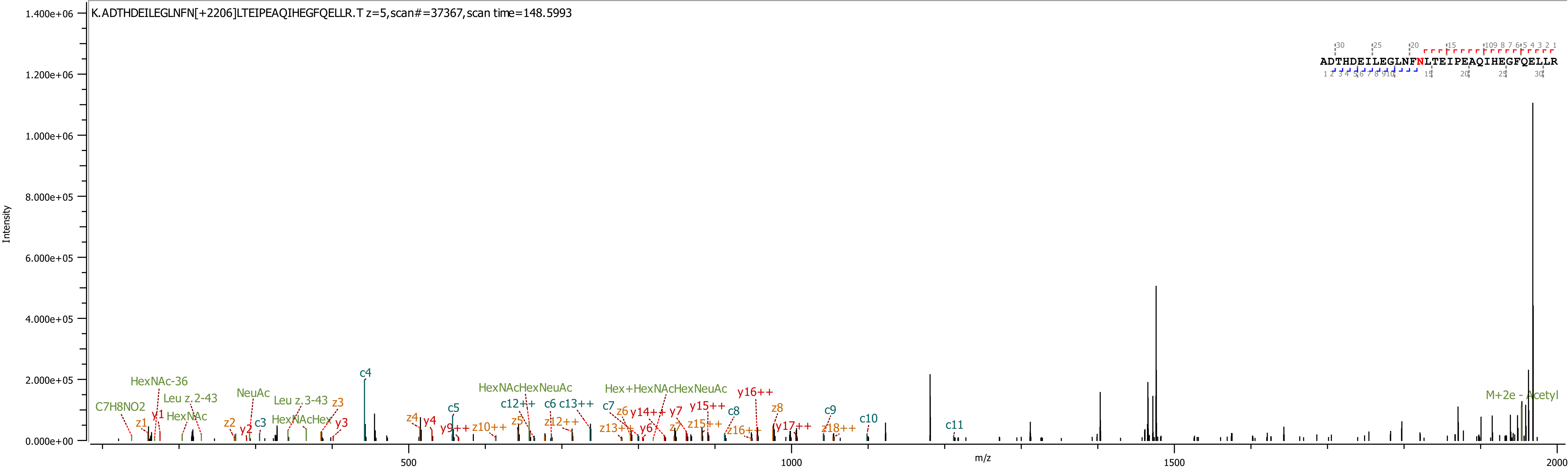

K.ADTHDEILEGLNFN[+2206]LTEIPEAQIHEGFQELLR.T z=5,scan#=37367,scan time=148.5993
ADTHDEILEGLNFNLTEIPEAQIHEGFQELLR
Intensity
m/z
1.400e+06
1.200e+06
1.000e+06
8.000e+05
6.000e+05
4.000e+05
2.000e+05
0.000e+00
500
1000
1500
2000
C7H8NO2
HexNAc-36
Leu z.2-43
HexNAc
NeuAc
Leu z.3-43
HexNAcHex
c4
c5
c12++
c6
c13++
HexNAcHexNeuAc
Hex+HexNAcHexNeuAc
c7
y16++
c8
c9
c10
c11
M+2e - Acetyl

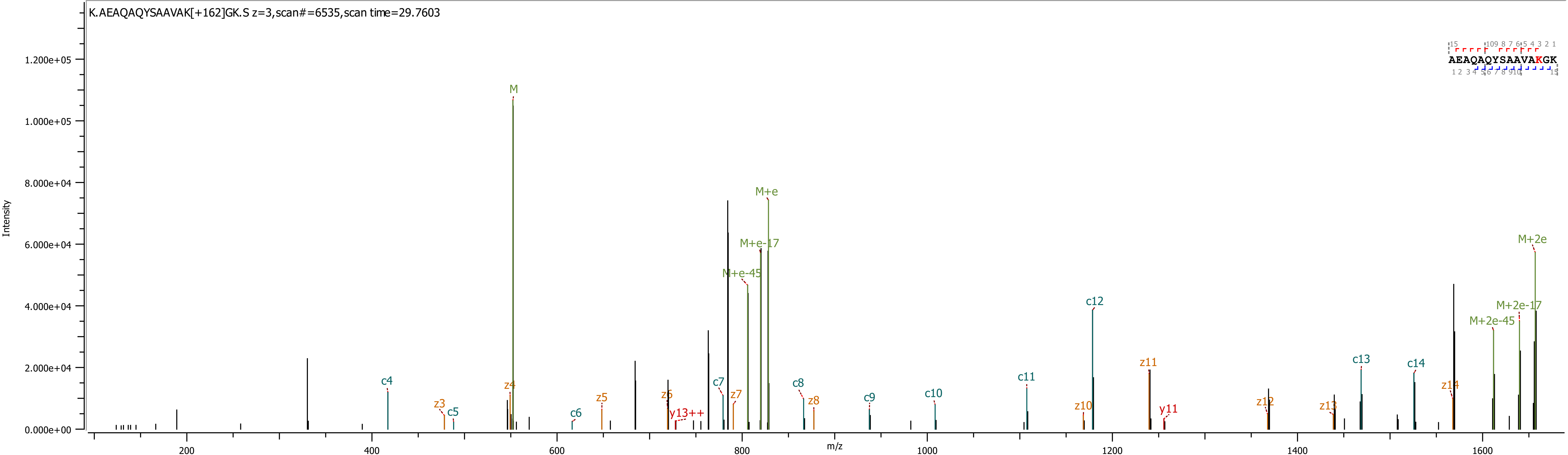

K.AEAQAQYSAAVAK[+162]GK.S z=3,scan#=6535,scan time=29.7603
Intensity
m/z
AEAQAQYSAAVAKGK
M
M+e
M+e-17
M+e-45
M+2e
M+2e-17
M+2e-45
c4
c5
c6
c7
c8
c9
c10
c11
c12
c13
c14
z3
z4
z5
z6
z7
z8
z10
z11
z12
z13
z14
y11
y13++

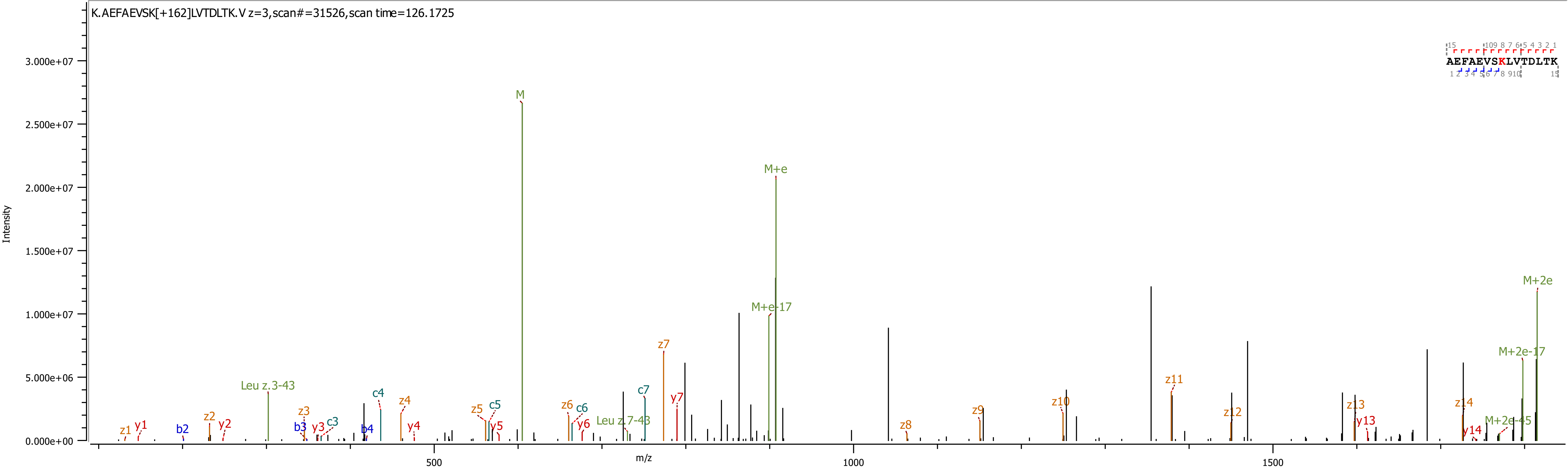

K.AEFAEVSK[+162]LVTDLTK.V z=3,scan#=31526,scan time=126.1725
Intensity
m/z
AEFAEVSKLVTDLTK
M
M+e
M+e-17
M+2e
M+2e-17
M+2e-45
Leu z.3-43
Leu z.7-43

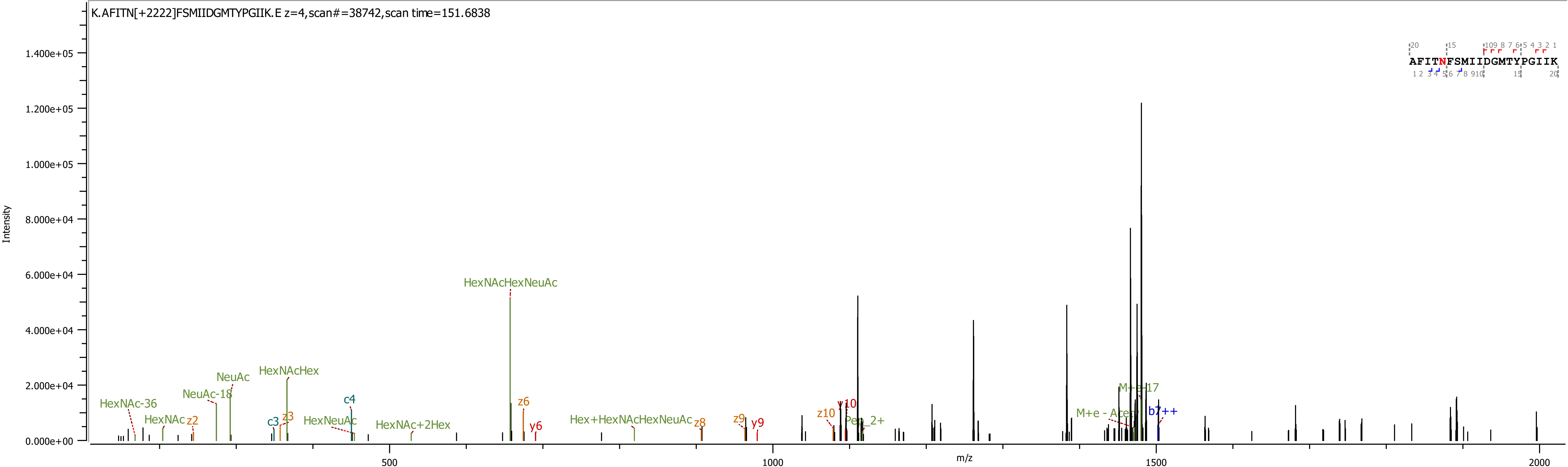
K.AFITN[+2222]FSMIIDGMTYPGIIK.E z=4,scan#=38742,scan time=151.6838
Intensity
m/z
AFITNFSMIIDGMTYPGIIK
HexNAcHexNeuAc
HexNAcHex
NeuAc
NeuAc-18
HexNAc-36
HexNAc
HexNeuAc
HexNAc+2Hex
Hex+HexNAcHexNeuAc
M+e-17
M+e - Acet
Pep_2+
b7++
c3
c4
z2
z3
z6
z8
z9
z10
y6
y9
y10

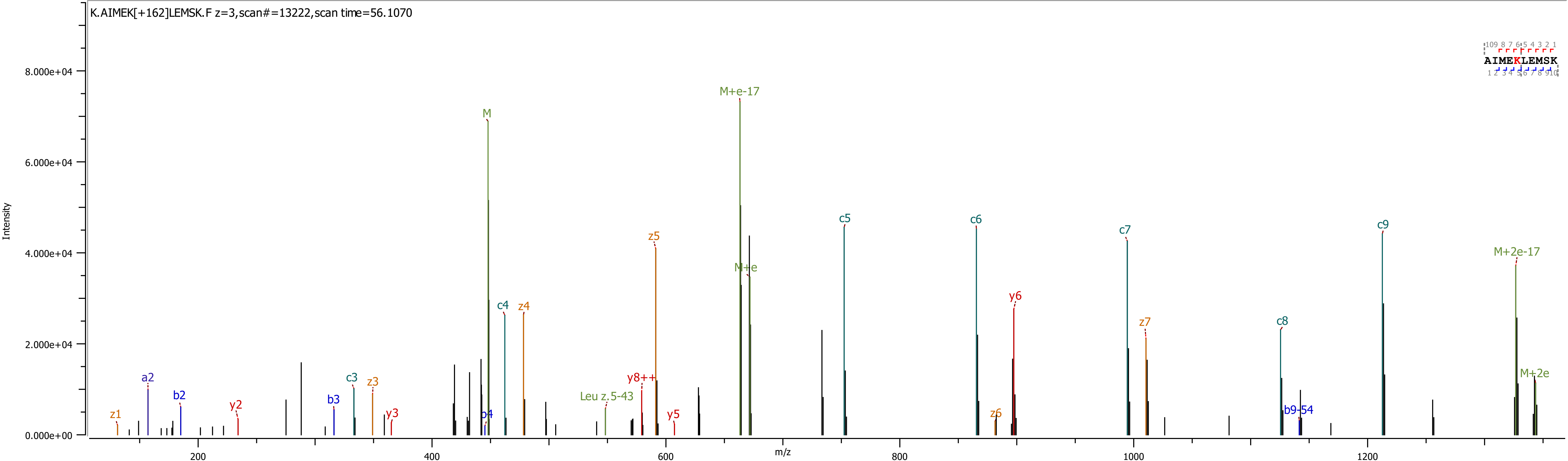
K.AIMEK[+162]LEMSK.F z=3,scan#=13222,scan time=56.1070
Intensity
m/z
8.000e+04
6.000e+04
4.000e+04
2.000e+04
0.000e+00
200
400
600
800
1000
1200
AIMEKLEMSK
M+e-17
M
M+e
z5
c5
c6
c7
c9
M+2e-17
M+2e
c4
z4
y6
z7
c8
b9-54
z6
y8++
Leu z.5-43
y5
y3
z3
c3
b3
b4
a2
b2
y2
z1

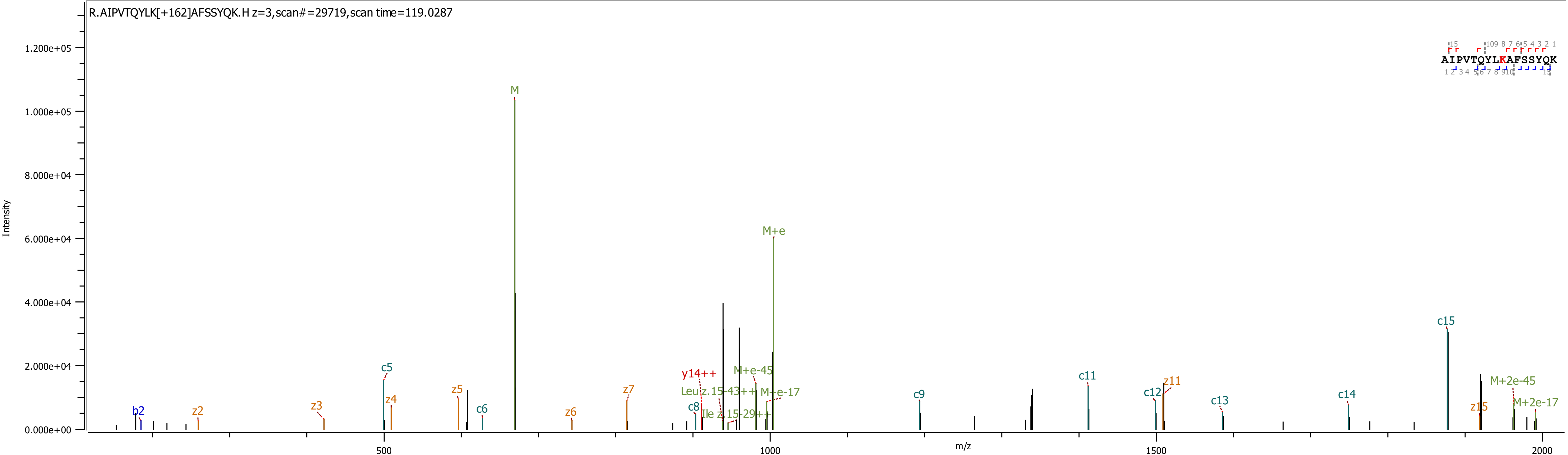

R.AIPVTQYLK[+162]AFSSYQK.H z=3,scan#=29719,scan time=119.0287
Intensity
m/z
1.200e+05
1.000e+05
8.000e+04
6.000e+04
4.000e+04
2.000e+04
0.000e+00
500
1000
1500
2000
AIPVTQYLKAFSSYQK
M
M+e
c15
c5
c11
c9
c12
z11
c13
c14
M+2e-45
M+2e-17
z15
b2
z2
z3
z4
z5
c6
z6
z7
c8
y14++
M+e-45
M+e-17
Leu z.15-43++
Ile z.15-29++

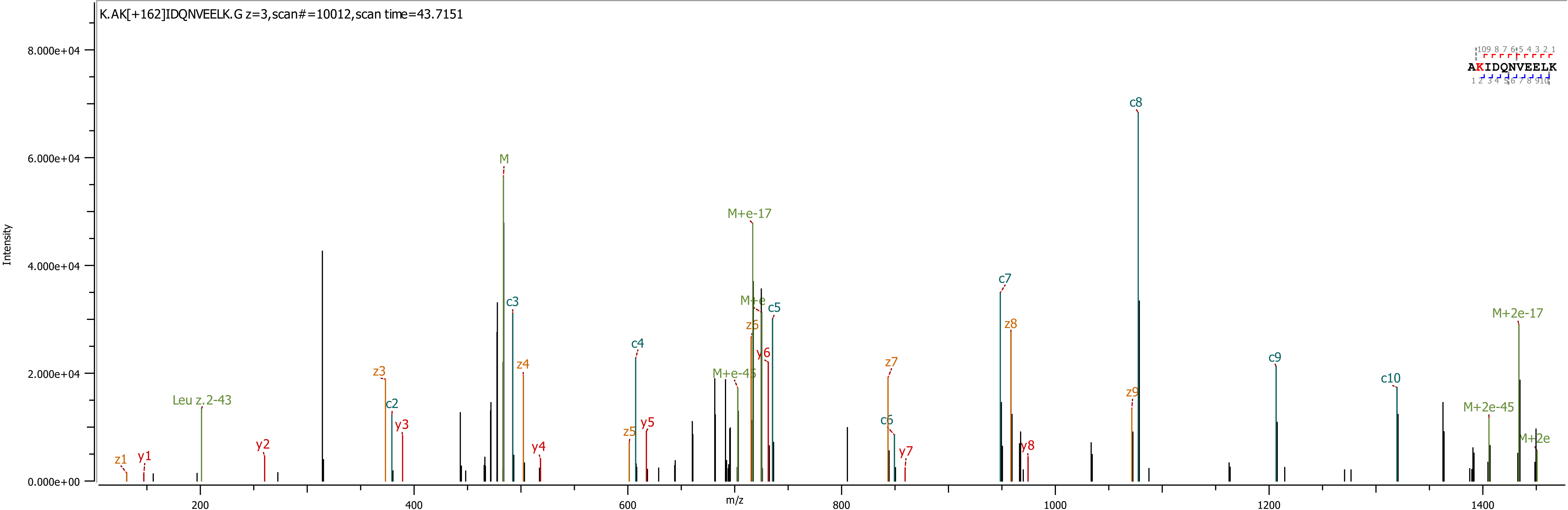

K.AK[+162]IDQNVEELK.G z=3,scan#=10012,scan time=43.7151
AKIDQNVEELK
Intensity
m/z
M
c8
M+e-17
c7
c3
M+e
c5
z6
z8
y6
c4
z4
z3
M+e-45
z7
c9
c10
M+2e-17
z9
Leu z.2-43
c2
y3
y5
c6
M+2e-45
z5
y4
y7
y8
y2
y1
z1
M+2e
8.000e+04
6.000e+04
4.000e+04
2.000e+04
0.000e+00
200
400
600
800
1000
1200
1400

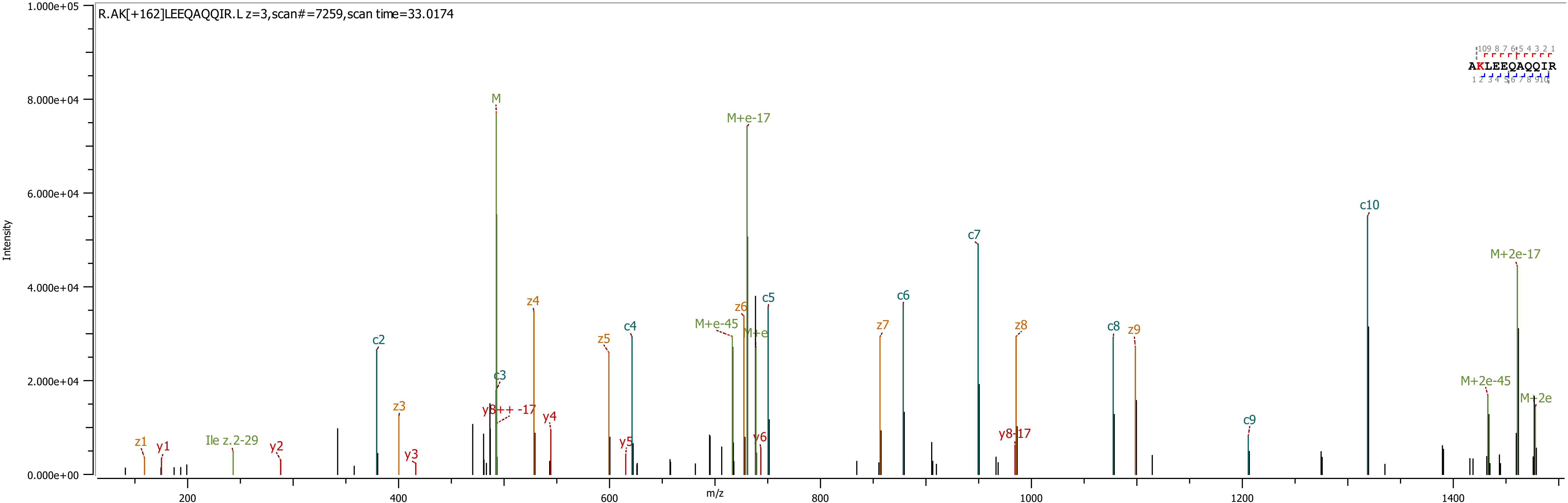
R.AK[+162]LEEQAQQIR.L z=3,scan#=7259,scan time=33.0174
AKLEEQAQQIR
Intensity
m/z
M
M+e-17
c10
c7
M+2e-17
c6
c5
z4
z6
M+e-45
M+e
c4
z5
c2
z7
z8
c8
z9
c3
z3
y8++ -17
y4
M+2e-45
M+2e
c9
y8-17
y6
y5
z1
y1
Ile z.2-29
y2
y3

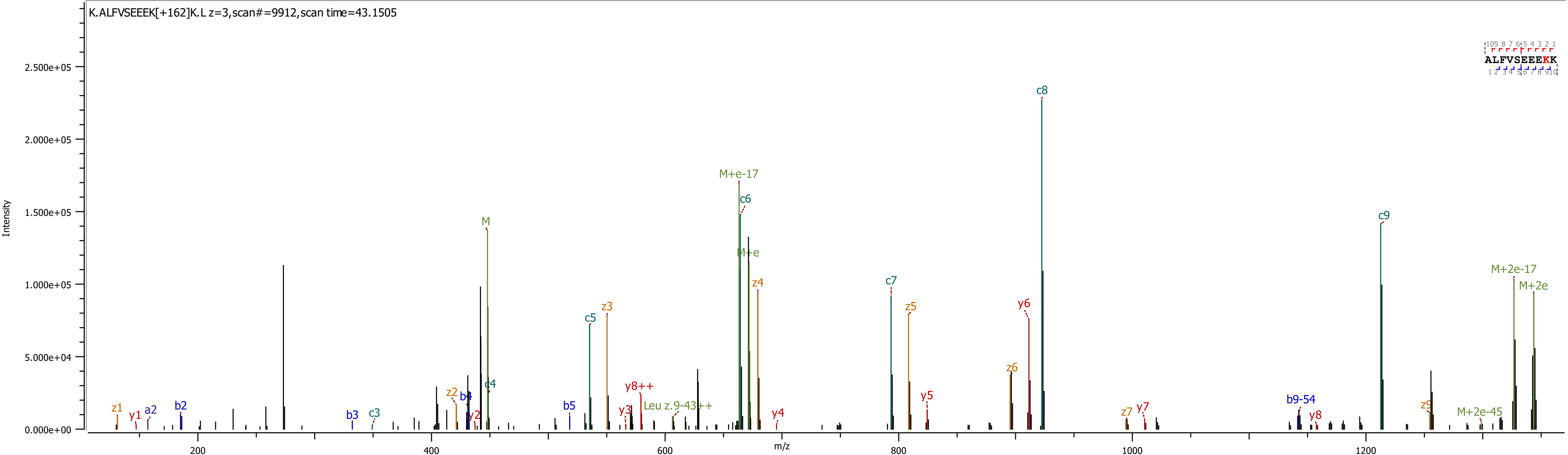
K.ALFVSEEEK[+162]K.L z=3,scan#=9912,scan time=43.1505
Intensity
m/z
ALFVSEEEKK
c8
M+e-17
c6
M
M+e
z4
c7
z5
y6
c9
M+2e-17
M+2e
c5
z3
z6
y8++
Leu z.9-43++
c4
z2
b4
y2
b5
y3
y4
y5
z7
y7
b9-54
y8
z9
M+2e-45
z1
y1
a2
b2
b3
c3

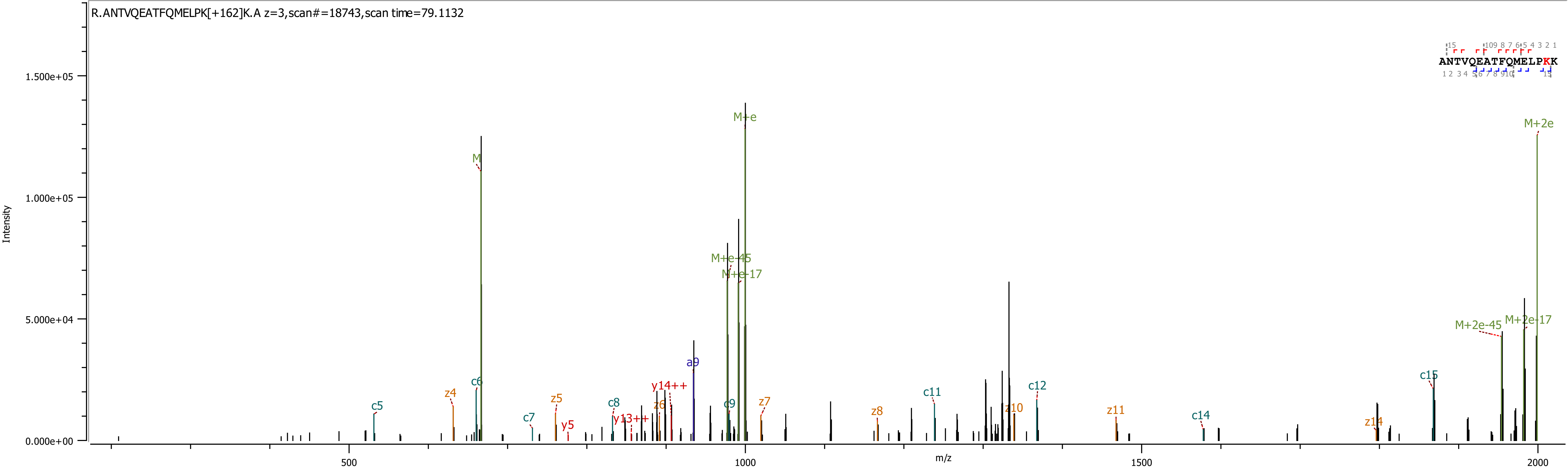
R.ANTVQEATFQMELPK[+162]K.A z=3,scan#=18743,scan time=79.1132
ANTVQEATFQMELPKK
Intensity
m/z
M
M+e
M+2e
M+e-45
M+e-17
M+2e-45
M+2e-17
c5
c6
c7
c8
c9
c11
c12
c14
c15
z4
z5
z6
z7
z8
z10
z11
z14
y5
y13++
y14++
a9
1.500e+05
1.000e+05
5.000e+04
0.000e+00
500
1000
1500
2000

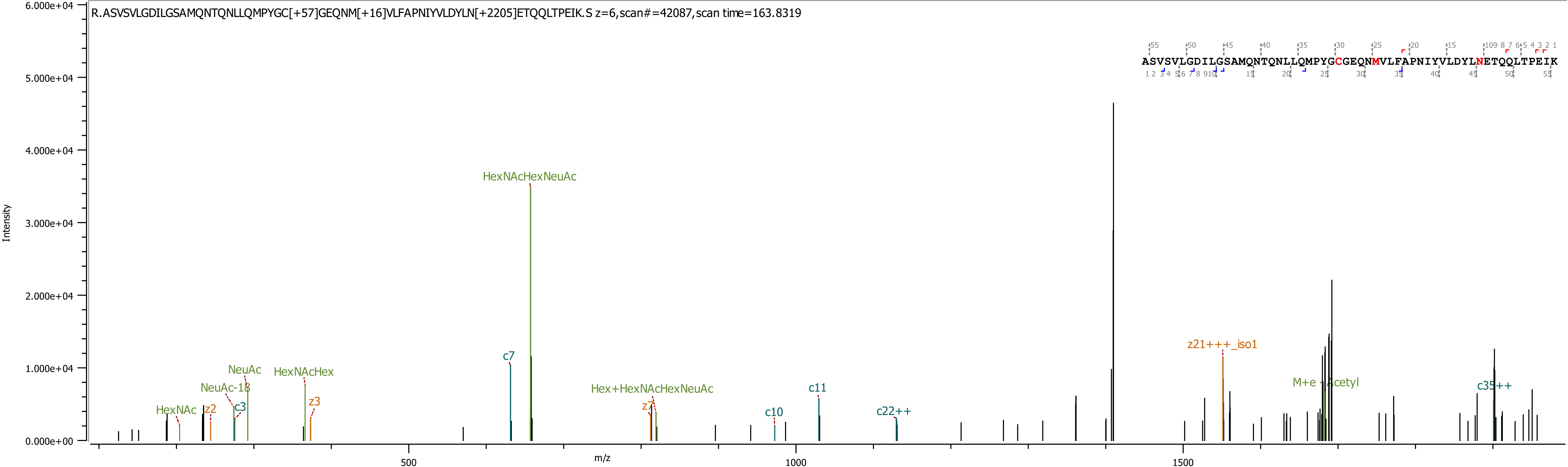

R.ASVSVLGDILGSAMQNTQNLLQMPYGC[+57]GEQNM[+16]VLFAPNIYVLDYLN[+2205]ETQQLTPEIK.S z=6,scan#=42087,scan time=163.8319
ASVSVLGDILGSAMQNTQNLLQMPYGCGEQNMVLFAPNIYVLDYLNETQQLTPEIK
Intensity
m/z
HexNAc
z2
NeuAc-18
NeuAc
c3
HexNAcHex
z3
c7
HexNAcHexNeuAc
Hex+HexNAcHexNeuAc
z7
c10
c11
c22++
z21+++_iso1
M+e - Acetyl
c35++

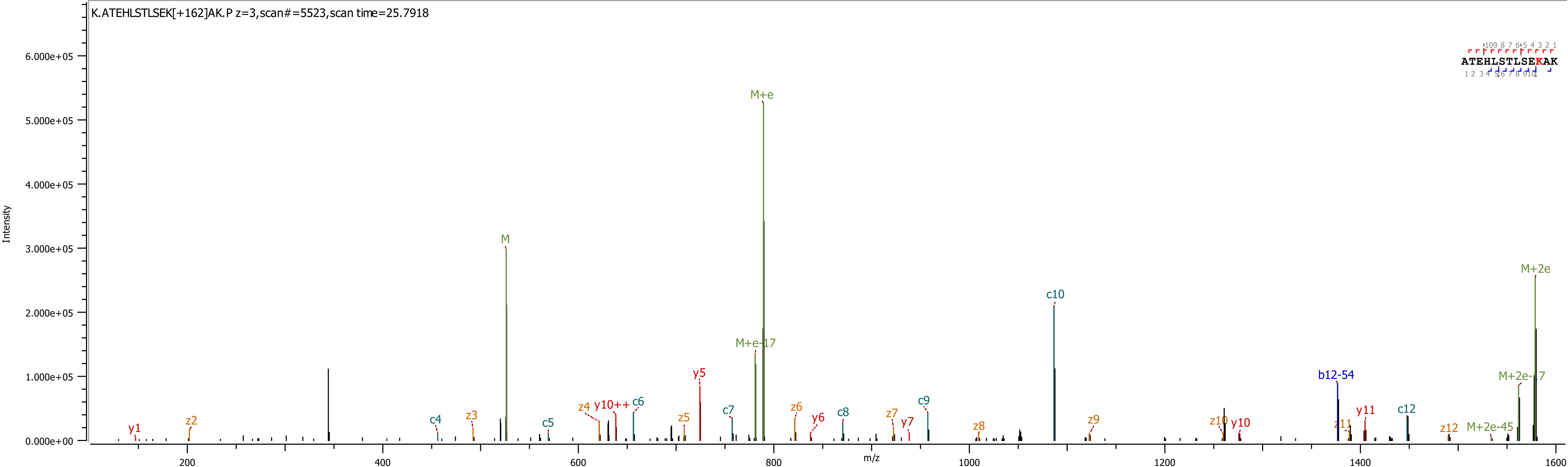
K.ATEHLSTLSEK[+162]AK.P z=3,scan#=5523,scan time=25.7918
Intensity
m/z
ATEHLSTLSEKAK
M+e
M
M+e-17
M+2e
M+2e-17
M+2e-45
c10
b12-54
y1
z2
c4
z3
c5
z4
y10++
c6
z5
y5
c7
z6
y6
c8
z7
y7
c9
z8
z9
z10
y10
z11
y11
c12
z12
0.000e+00
1.000e+05
2.000e+05
3.000e+05
4.000e+05
5.000e+05
6.000e+05
200
400
600
800
1000
1200
1400
1600

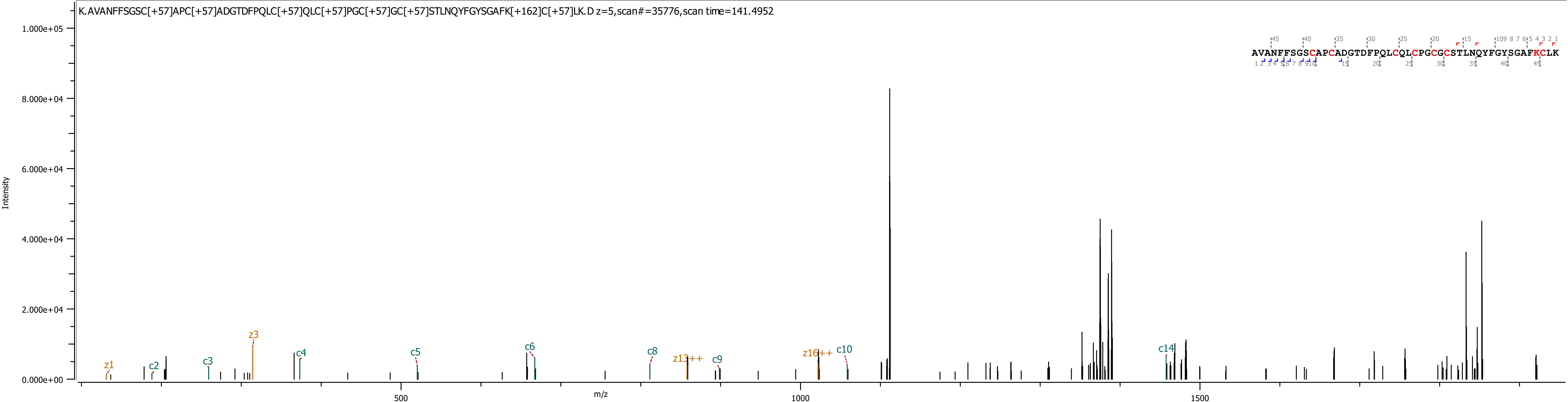

K.AVANFFSGSC[+57]APC[+57]ADGTDFPQLC[+57]QLC[+57]PGC[+57]GC[+57]STLNQYFGYSGAFK[+162]C[+57]LK.D z=5,scan#=35776,scan time=141.4952
AVANFFSGSCAPCADGTDFPQLCQLCPGCGCSTLNQYFGYSGAFKCLK
Intensity
m/z
1.000e+05
8.000e+04
6.000e+04
4.000e+04
2.000e+04
0.000e+00
500
1000
1500
z1
c2
c3
z3
c4
c5
c6
c8
z13++
c9
z16++
c10
c14

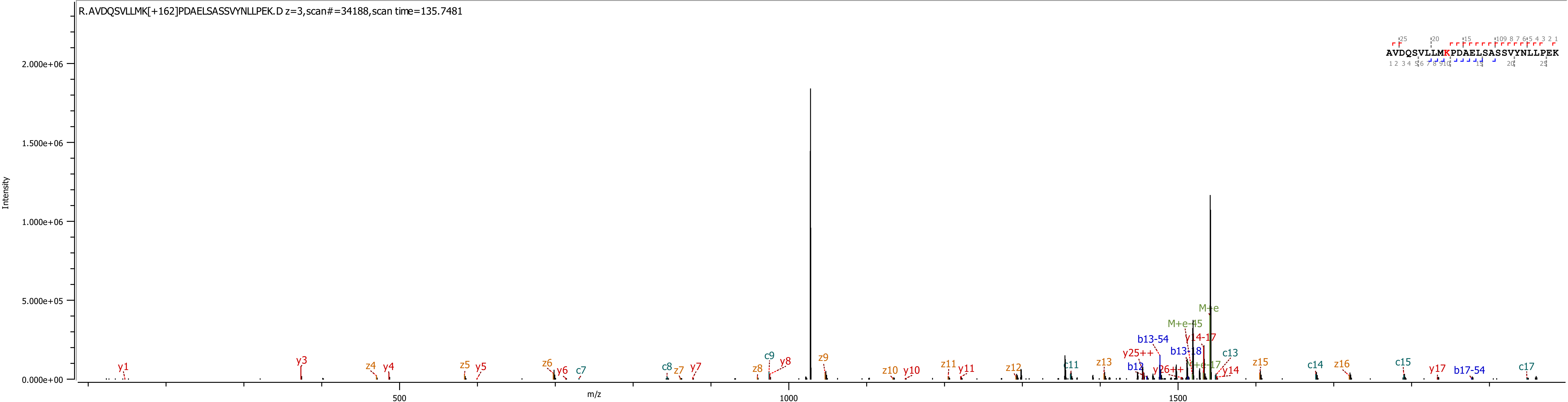

R.AVDQSVLLMK[+162]PDAELSASSVYNLLPEK.D z=3,scan#=34188,scan time=135.7481
AVDQSVLLMKPDAELSASSVYNLLPEK
Intensity
m/z
2.000e+06
1.500e+06
1.000e+06
5.000e+05
0.000e+00
500
1000
1500
y1
y3
z4
y4
z5
y5
z6
y6
c7
c8
z7
y7
z8
c9
y8
z9
z10
y10
z11
y11
z12
c11
z13
b12
y25++
b13-54
y26++
b13-18
M+e-45
y14-17
M+e
c13
y14
z15
c14
z16
c15
y17
b17-54
c17

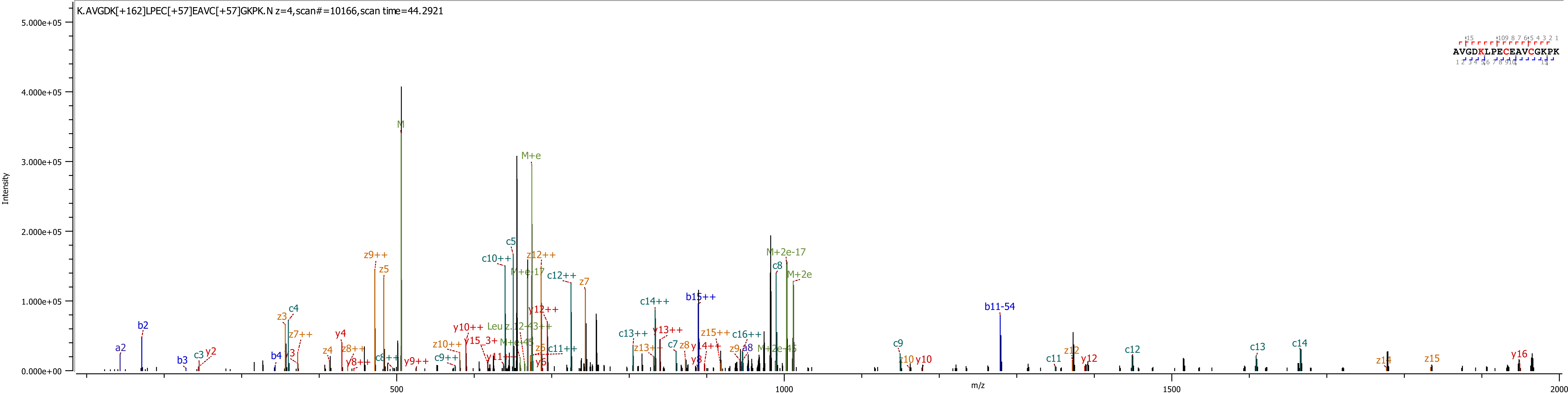
K.AVGDK[+162]LPEC[+57]EAVC[+57]GKPK.N z=4,scan#=10166,scan time=44.2921
AVGDKLPECEAVCGKPK
Intensity
m/z
0.000e+00
1.000e+05
2.000e+05
3.000e+05
4.000e+05
5.000e+05
500
1000
1500
2000
M
M+e
M+2e-17
M+2e
b11-54
b15++
c14++
c10++
c5
z12++
z9++
z5
c12++
z7
y12++
y10++
y15_3+
z10++
c9++
y9++
c8++
y8++
z8++
y4
z4
z7++
z3
c4
y3
b4
c3
y2
b3
b2
a2
c13++
z13++
y13++
c7
z8
y14++
y8
z15++
c16++
z9
a8
c9
z10
y10
c11
z12
y12
c12
c13
c14
z14
z15
y16

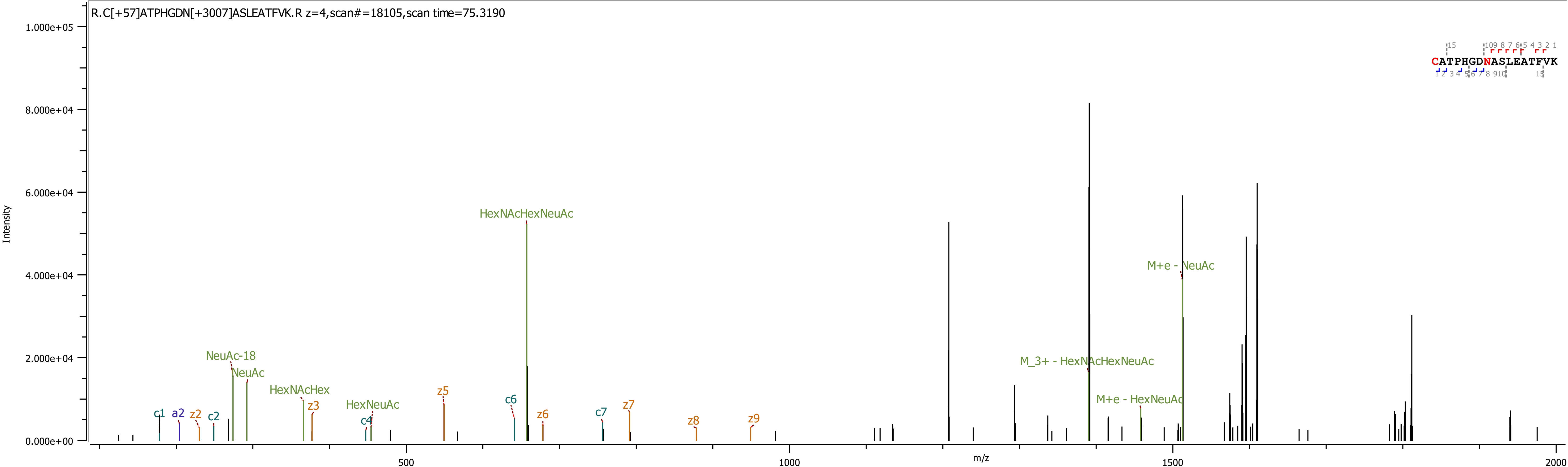

R.C[+57]ATPHGDN[+3007]ASLEATFVK.R z=4,scan#=18105,scan time=75.3190
CATPHGDNASLEATFVK
Intensity
m/z
HexNAcHexNeuAc
NeuAc-18
NeuAc
HexNAcHex
HexNeuAc
M+e - NeuAc
M_3+ - HexNAcHexNeuAc
M+e - HexNeuAc
c1
a2
z2
c2
z3
c4
z5
c6
z6
c7
z7
z8
z9

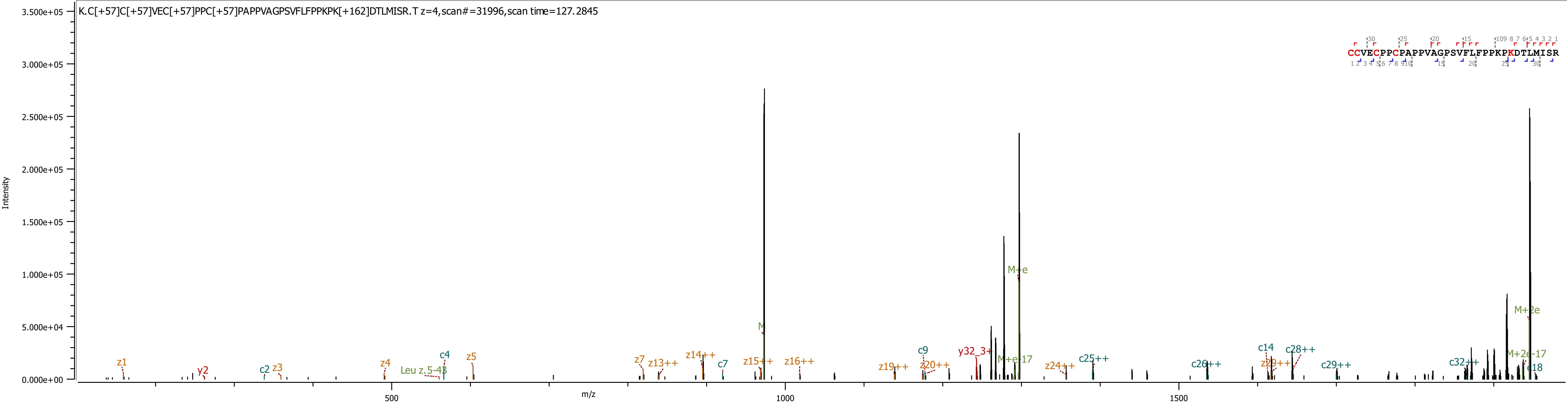
K.C[+57]C[+57]VEC[+57]PPC[+57]PAPPVAGPSVFLFPPKPK[+162]DTLMISR.T z=4,scan#=31996,scan time=127.2845
Intensity
m/z
CCVECPPCPAPPVAGPSVFLFPPKPKDTLMISR
M+e
M+2e
M+e-17
M+2e-17
y32_3+
Leu z.5-43

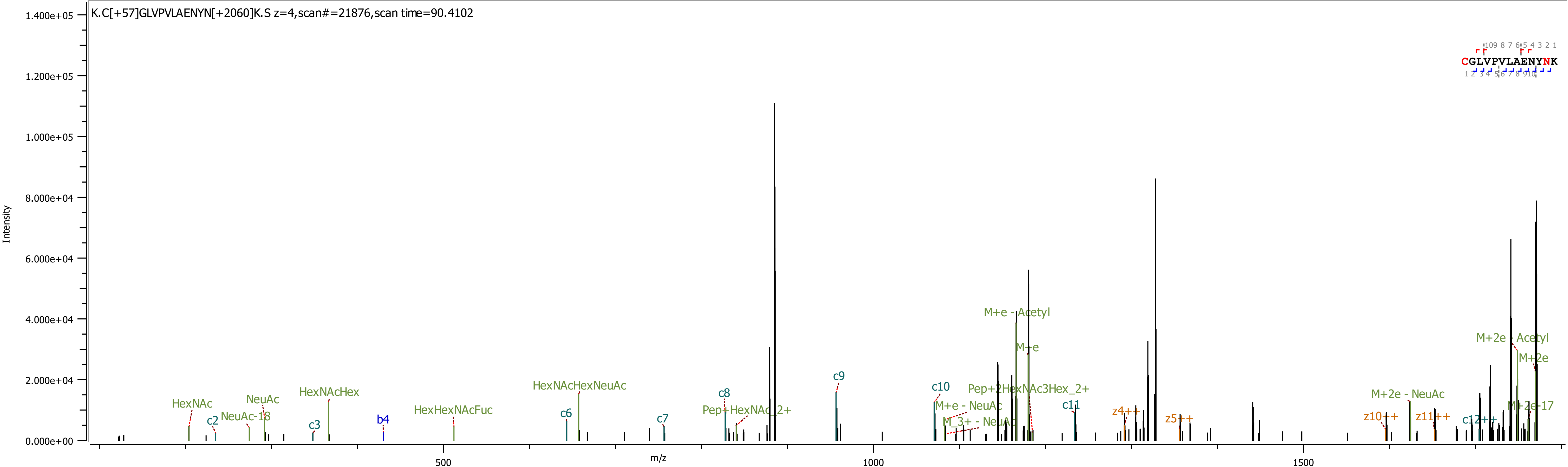
K.C[+57]GLVPVLAENYN[+2060]K.S z=4,scan#=21876,scan time=90.4102
Intensity
m/z
1.400e+05
1.200e+05
1.000e+05
8.000e+04
6.000e+04
4.000e+04
2.000e+04
0.000e+00
500
1000
1500
CGLVPVLAENYNK
HexNAc
c2
NeuAc-18
NeuAc
c3
HexNAcHex
b4
HexHexNAcFuc
c6
HexNAcHexNeuAc
c7
c8
Pep+HexNAc_2+
c9
c10
M+e - NeuAc
M_3+ - NeuAc
Pep+2HexNAc3Hex_2+
M+e - Acetyl
M+e
c11
z4++
z5++
z10++
z11++
M+2e - NeuAc
c12++
M+2e - Acetyl
M+2e
M+2e-17

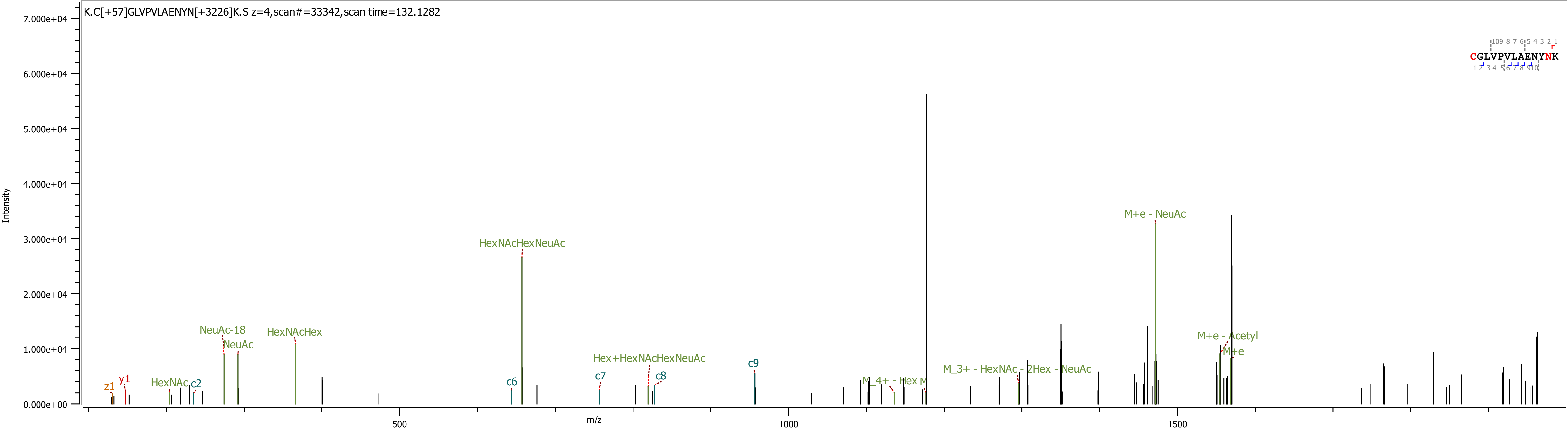
K.C[+57]GLVPVLAENYN[+3226]K.S z=4,scan#=33342,scan time=132.1282
Intensity
m/z
7.000e+04
6.000e+04
5.000e+04
4.000e+04
3.000e+04
2.000e+04
1.000e+04
0.000e+00
500
1000
1500
CGLVPVLAENYNK
z1
y1
HexNAc
c2
NeuAc-18
NeuAc
HexNAcHex
HexNAcHexNeuAc
c6
c7
Hex+HexNAcHexNeuAc
c8
c9
M_4+ - Hex M
M_3+ - HexNAc - 2Hex - NeuAc
M+e - NeuAc
M+e - Acetyl
M+e

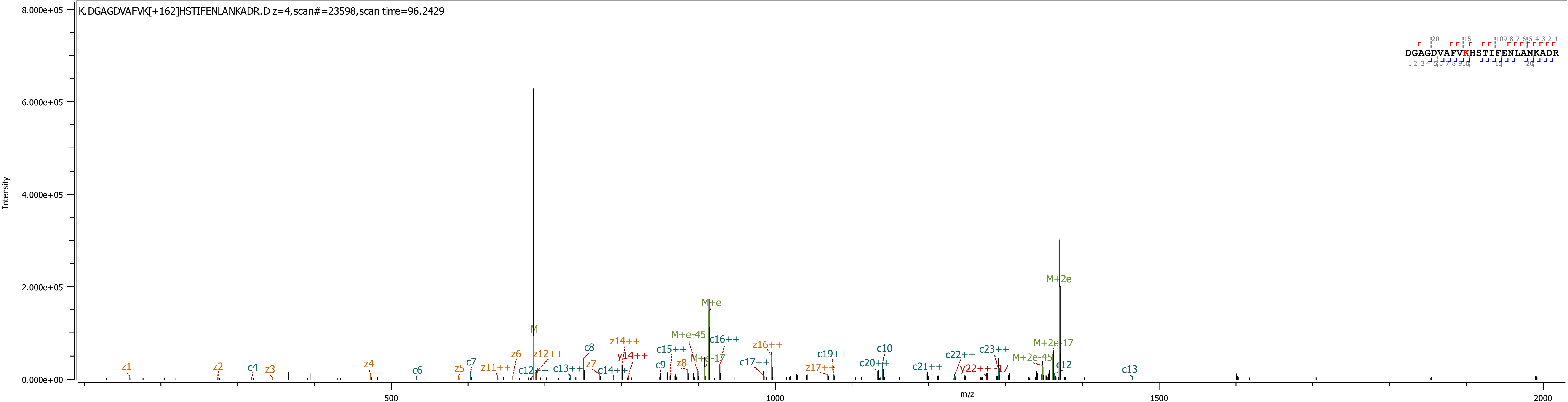
K.DGAGDVAFVK[+162]HSTIFENLANKADR.D z=4,scan#=23598,scan time=96.2429
DGAGDVAFVKHSTIFENLANKADR
Intensity
m/z
8.000e+05
6.000e+05
4.000e+05
2.000e+05
0.000e+00
500
1000
1500
2000
z1
z2
c4
z3
z4
c6
z5
c7
z11++
z6
M
c12++
z12++
c13++
c8
z7
c14++
z14++
y14++
c9
c15++
M+e-45
z8
M+e-17
M+e
c16++
c17++
z16++
z17++
c19++
c20++
c10
c21++
c22++
y22++-17
c23++
M+2e-45
M+2e-17
M+2e
c12
c13

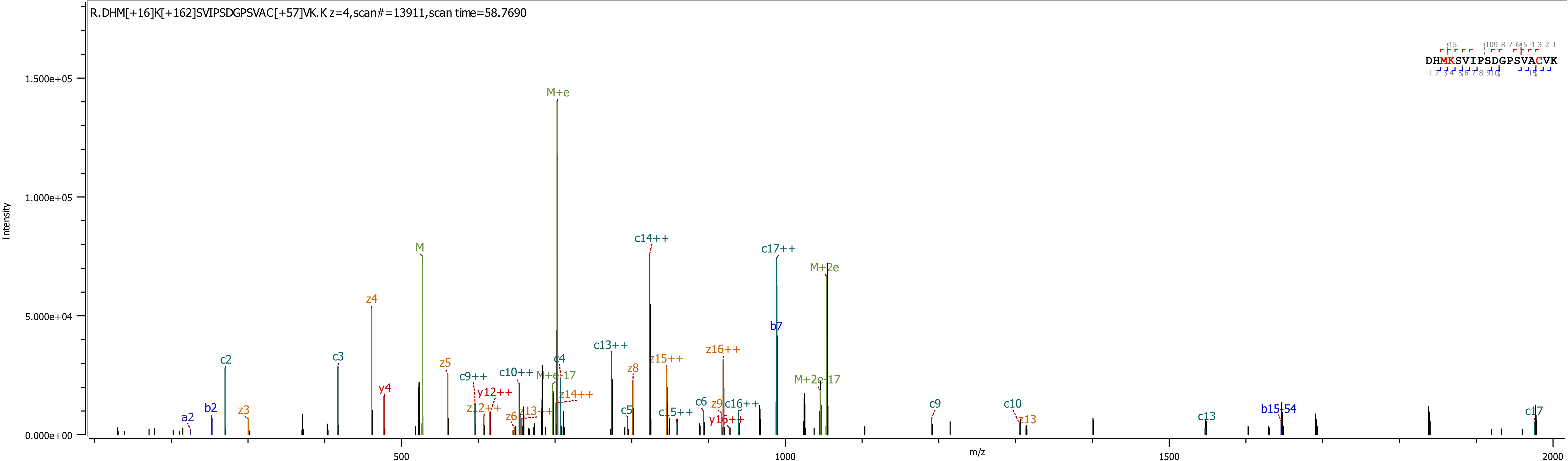
R.DHM[+16]K[+162]SVIPSDGPSVAC[+57]VK.K z=4,scan#=13911,scan time=58.7690
DHMKSVIPSDGPSVACVK
Intensity
m/z
1.500e+05
1.000e+05
5.000e+04
0.000e+00
500
1000
1500
2000
M+e
M
c14++
c17++
M+2e
z4
b7
c13++
z16++
z15++
c2
c3
z5
z8
c4
c9++
c10++
M+e-17
M+2e-17
y12++
y4
z14++
z12++
z13++
z6
c5
c15++
c6
z9
c16++
y16++
z3
b2
a2
c9
c10
z13
c13
b15-54
c17

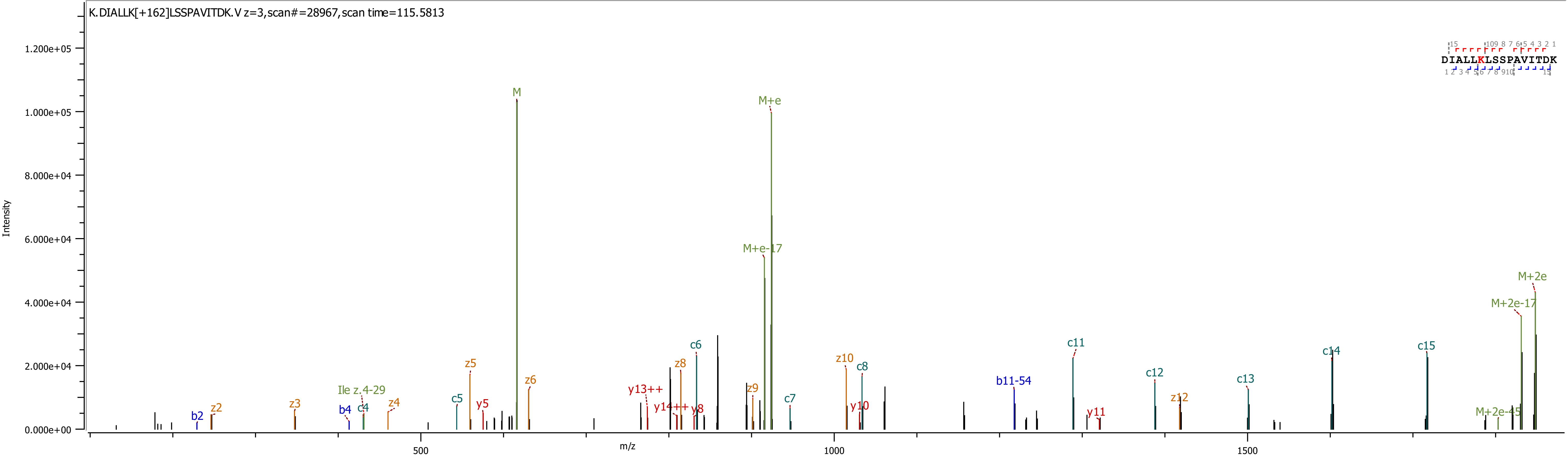
K.DIALLK[+162]LSSPAVITDK.V z=3,scan#=28967,scan time=115.5813
Intensity
m/z
DIALLKLSSPAVITDK
M
M+e
M+e-17
M+2e
M+2e-17
M+2e-45
Ile z.4-29
b2
z2
z3
b4
c4
z4
c5
z5
y5
z6
y13++
y14++
y8
z8
c6
z9
c7
z10
y10
c8
b11-54
c11
y11
c12
z12
c13
c14
c15

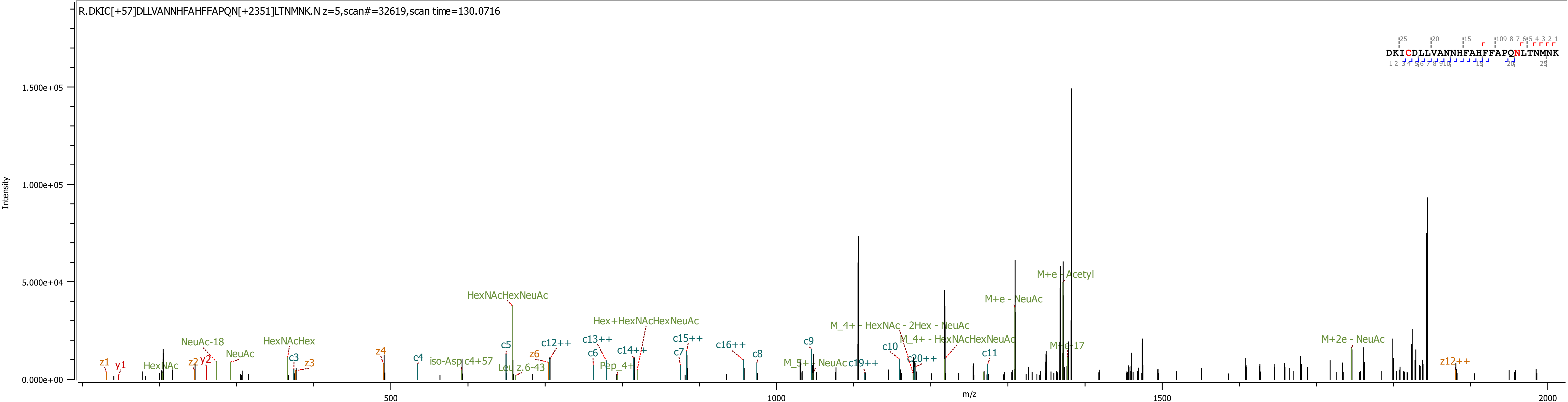
R.DKIC[+57]DLLVANNHFAHFFAPQN[+2351]LTNMNK.N z=5,scan#=32619,scan time=130.0716
DKICDLLVANNHFAHFFAPQNLTNMNK
Intensity
m/z
1.500e+05
1.000e+05
5.000e+04
0.000e+00
500
1000
1500
2000
z1
y1
HexNAc
z2
y2
NeuAc-18
NeuAc
HexNAcHex
c3
z3
z4
c4
iso-Asp c4+57
c5
HexNAcHexNeuAc
Leu z.6-43
z6
c12++
c13++
c6
Pep_4+
c14++
Hex+HexNAcHexNeuAc
c7
c15++
c16++
c8
c9
M_5+ - NeuAc
c19++
c10
M_4+ - HexNAc - 2Hex - NeuAc
M_4+ - HexNAcHexNeuAc
c20++
c11
M+e - NeuAc
M+e - Acetyl
M+e-17
M+2e - NeuAc
z12++

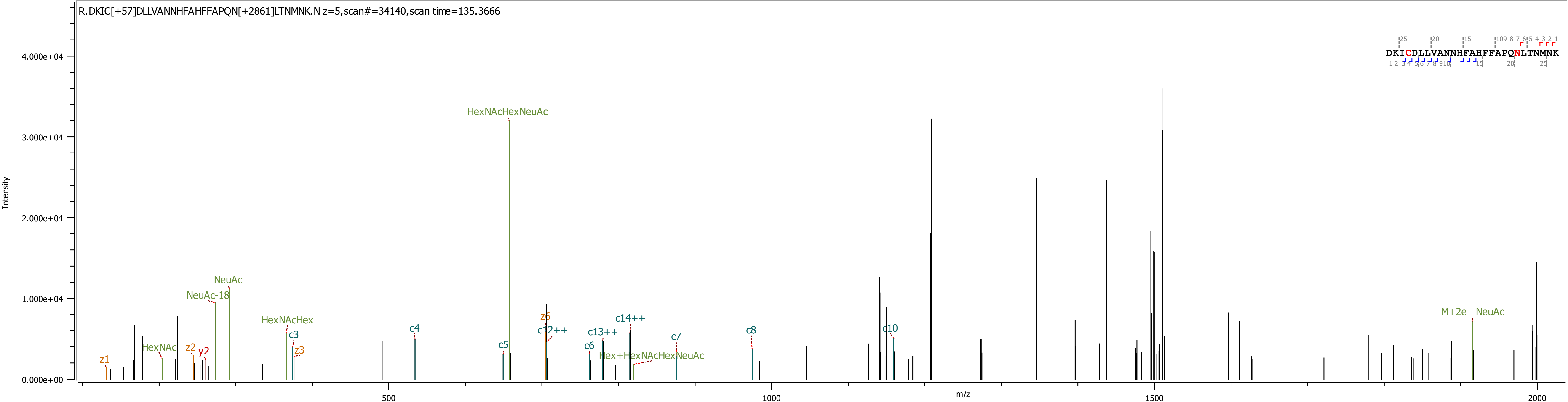
R.DKIC[+57]DLLVANNHFAHFFAPQN[+2861]LTNMNK.N z=5,scan#=34140,scan time=135.3666
DKICDLLVANNHFAHFFAPQNLTNMNK
Intensity
4.000e+04
3.000e+04
2.000e+04
1.000e+04
0.000e+00
HexNAcHexNeuAc
NeuAc
NeuAc-18
HexNAcHex
HexNAc
z1
z2
y2
c3
z3
c4
c5
z6
c12++
c6
c13++
c14++
Hex+HexNAcHexNeuAc
c7
c8
c10
M+2e - NeuAc
500
1000
1500
2000
m/z

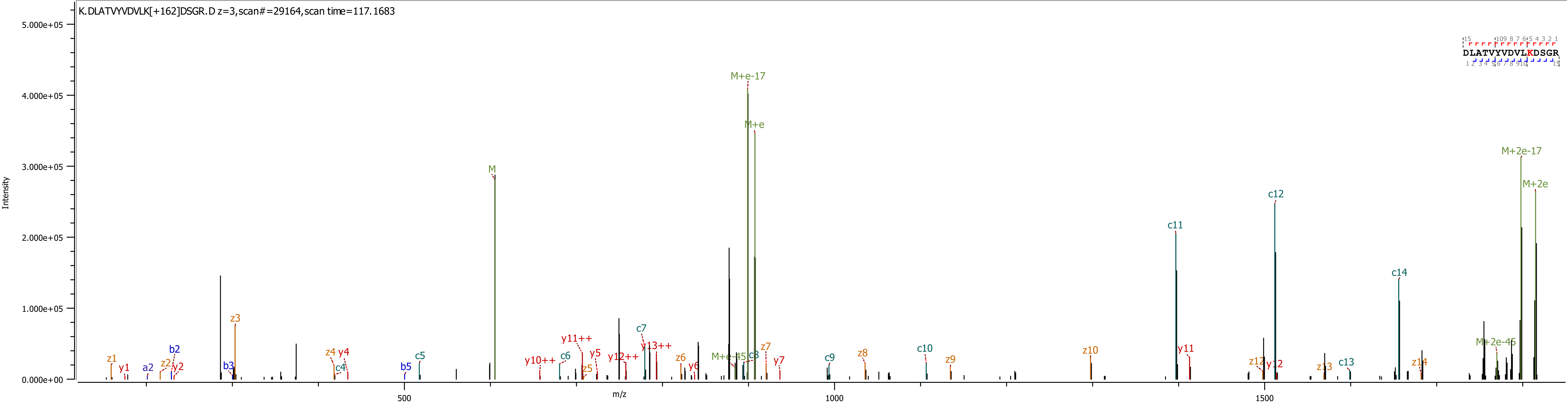
K.DLATVYVDVLK[+162]DSGR.D z=3,scan#=29164,scan time=117.1683
DLATVYVDVLKDSGR
Intensity
m/z
M+e-17
M+e
M
M+2e-17
M+2e
c11
c12
c14
z3
b2
z1
y1
a2
z2
y2
b3
z4
y4
c4
b5
c5
y11++
y10++
c6
y5
z5
y12++
c7
y13++
z6
y6
M+e-45
c8
z7
y7
c9
z8
c10
z9
z10
y11
z12
y12
z13
c13
z14
M+2e-45
500
1000
1500
0.000e+00
1.000e+05
2.000e+05
3.000e+05
4.000e+05
5.000e+05

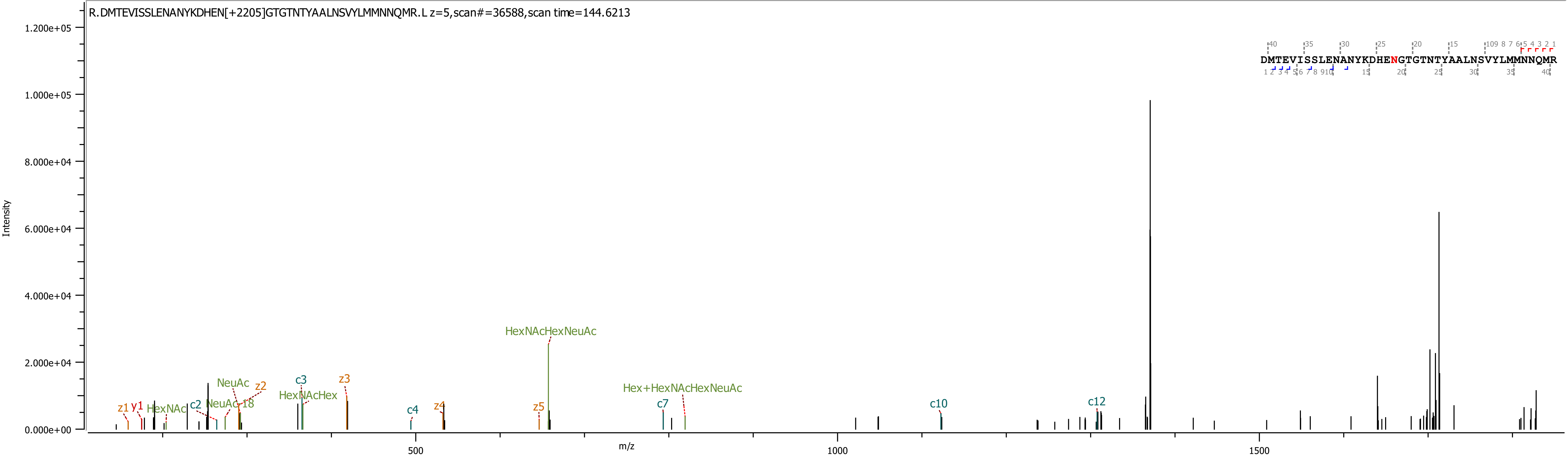
R.DMTEVISSLENANYKDHEN[+2205]GTGTNTYAALNSVYLMMNNQMR.L z=5,scan#=36588,scan time=144.6213
DMTEVISSLENANYKDHENGTGTNTYAALNSVYLMMNNQMR
Intensity
m/z
1.200e+05
1.000e+05
8.000e+04
6.000e+04
4.000e+04
2.000e+04
0.000e+00
500
1000
1500
z1
y1
HexNAc
c2
NeuAc
NeuAc-18
z2
c3
HexNAcHex
z3
c4
z4
z5
HexNAcHexNeuAc
c7
Hex+HexNAcHexNeuAc
c10
c12

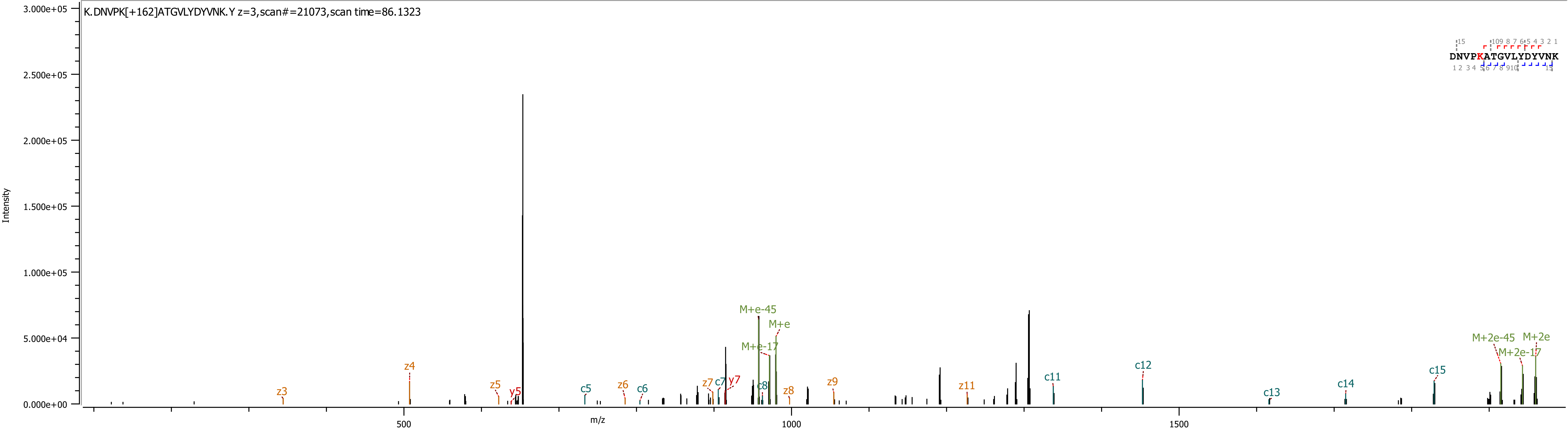

K.DNVPK[+162]ATGVLYDYVNK.Y z=3,scan#=21073,scan time=86.1323
Intensity
m/z
DNVPKATGVLYDYVNK
M+e-45
M+e
M+e-17
M+2e-45
M+2e
M+2e-17
z3
z4
z5
y5
c5
z6
c6
z7
c7
y7
c8
z8
z9
z11
c11
c12
c13
c14
c15

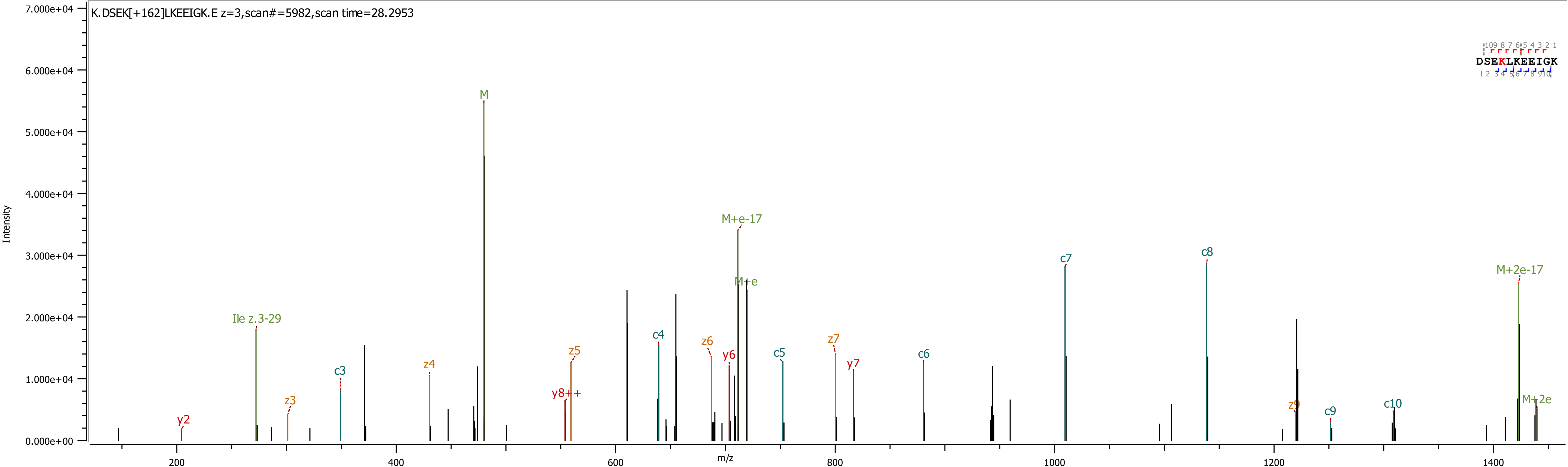
K.DSEK[+162]LKEEIGK.E z=3,scan#=5982,scan time=28.2953
Intensity
m/z
DSEKLKEEIGK
M
M+e-17
M+e
M+2e-17
M+2e
Ile z.3-29
y2
z3
c3
z4
y8++
z5
c4
z6
y6
c5
z7
y7
c6
c7
c8
z9
c9
c10

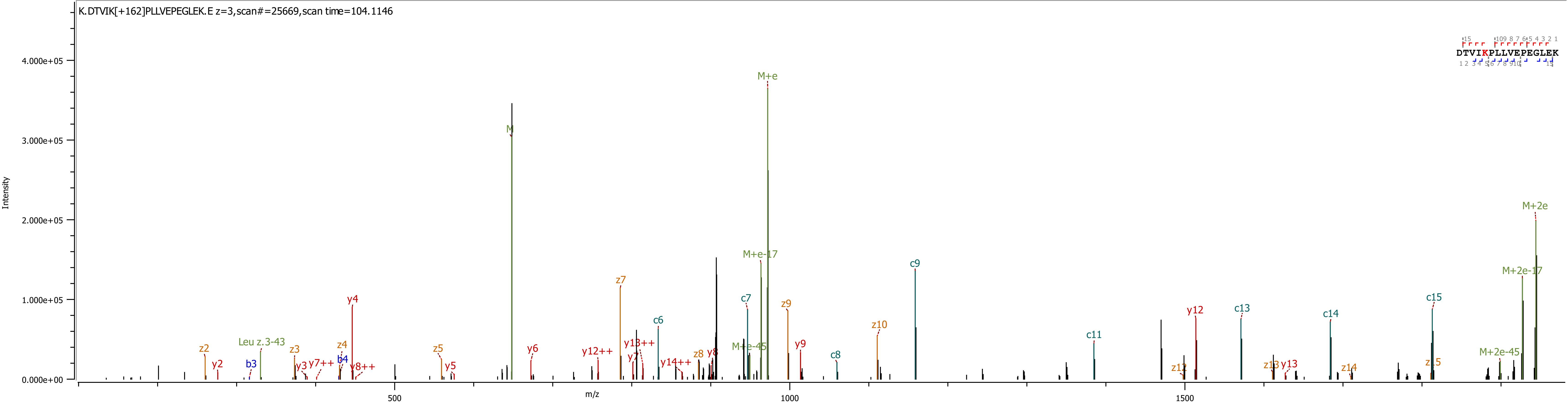
K.DTVIK[+162]PLLVEPEGLEK.E z=3,scan#=25669,scan time=104.1146
DTVIKPLLVEPEGLEK
Intensity
m/z
M+e
M
M+2e
M+2e-17
M+e-17
c9
c15
c13
c14
y12
c11
z7
y4
z9
c7
c6
z10
y13++
y12++
y6
z2
Leu z.3-43
z3
z4
z5
y2
b3
b4
y3
y7++
y8++
y5
y7
y14++
z8
y8
M+e-45
y9
c8
z12
z13
y13
z14
z15
M+2e-45

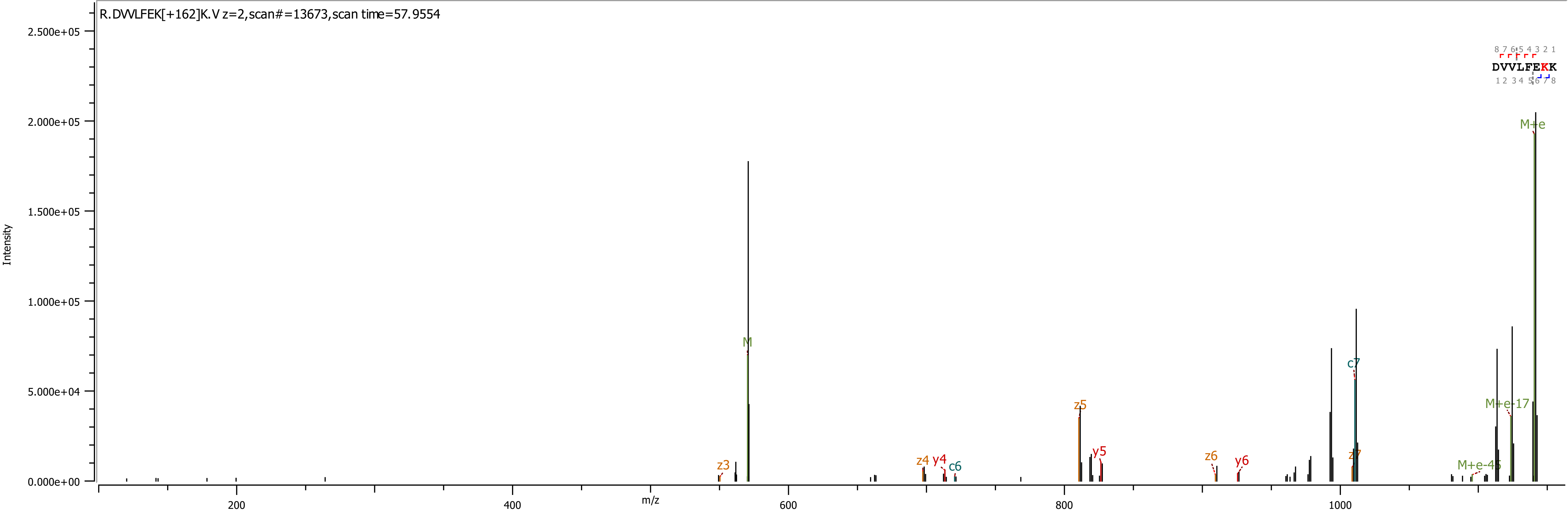

R.DVVLFEK[+162]K.V z=2,scan#=13673,scan time=57.9554
Intensity
2.500e+05
2.000e+05
1.500e+05
1.000e+05
5.000e+04
0.000e+00
200
400
600
800
1000
m/z
DVVLFEKK
M
M+e
M+e-17
M+e-45
z3
z4
z5
z6
z7
y4
y5
y6
c6
c7

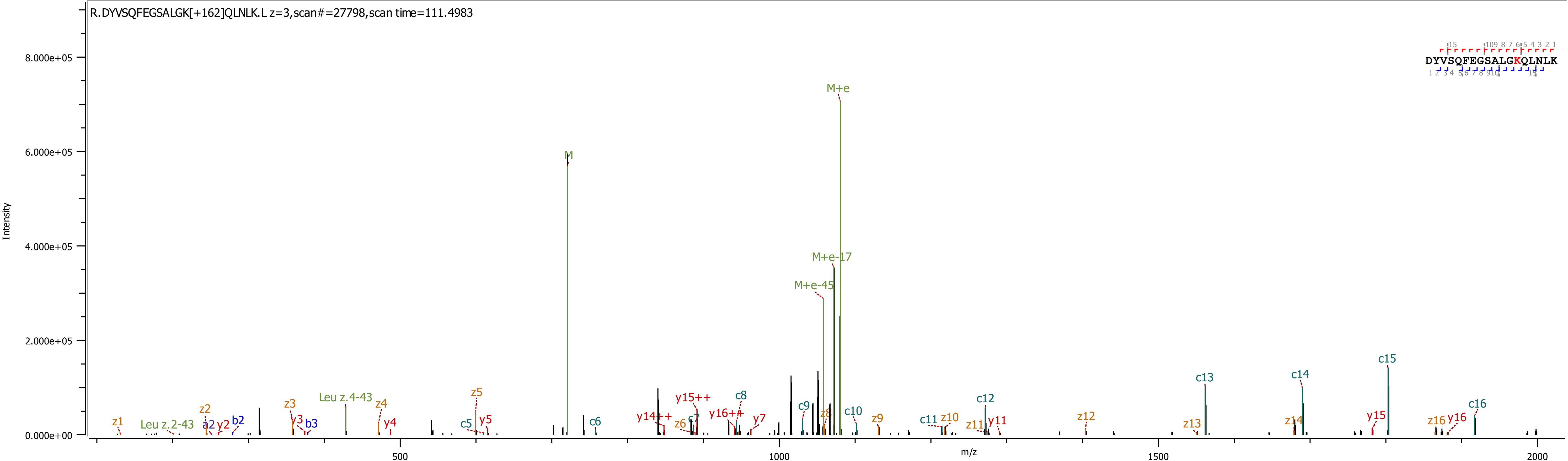
R.DYVSQFEGSALGK[+162]QLNLK.L z=3,scan#=27798,scan time=111.4983
Intensity
m/z
8.000e+05
6.000e+05
4.000e+05
2.000e+05
0.000e+00
500
1000
1500
2000
DYVSQFEGSALGKQLNLK
M
M+e
M+e-17
M+e-45
Leu z.2-43
Leu z.4-43
z1
z2
z3
z4
z5
z6
z8
z9
z10
z11
z12
z13
z14
z16
a2
b2
b3
y2
y3
y4
y5
y7
y11
y14++
y15++
y16++
y15
y16
c5
c6
c7
c8
c9
c10
c11
c12
c13
c14
c15
c16

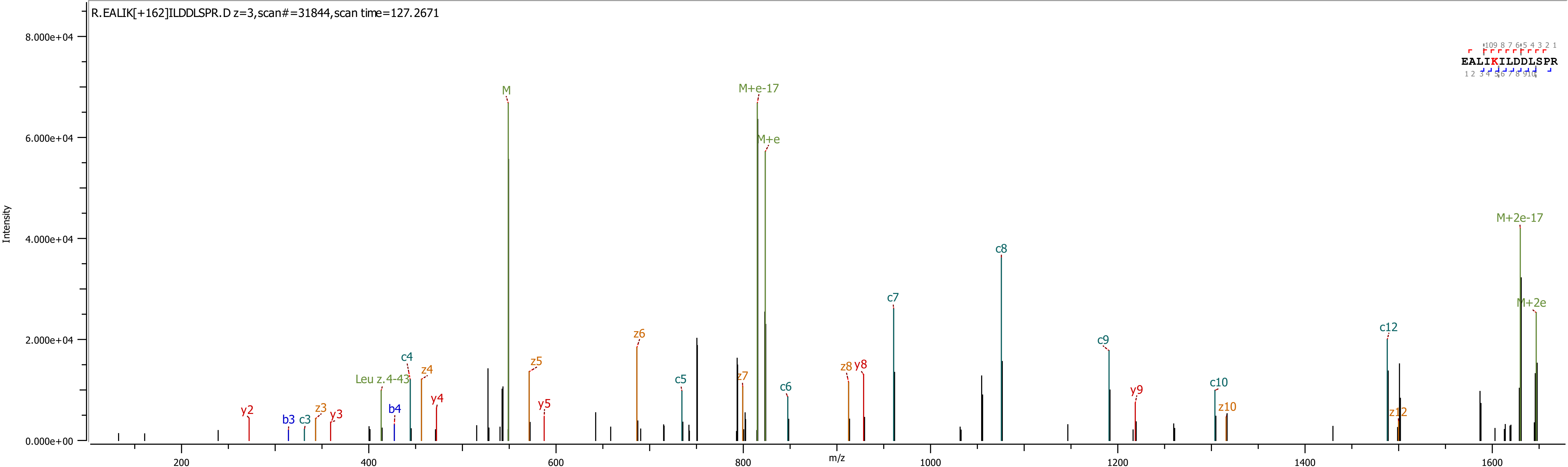
R.EALIK[+162]ILDDLSPR.D z=3,scan#=31844,scan time=127.2671
EALIKILDDLSPR
Intensity
m/z
M
M+e-17
M+e
M+2e-17
M+2e
Leu z.4-43
y2
b3
c3
z3
y3
b4
c4
z4
y4
z5
y5
z6
c5
z7
c6
z8
y8
c7
c8
c9
y9
c10
z10
c12
z12

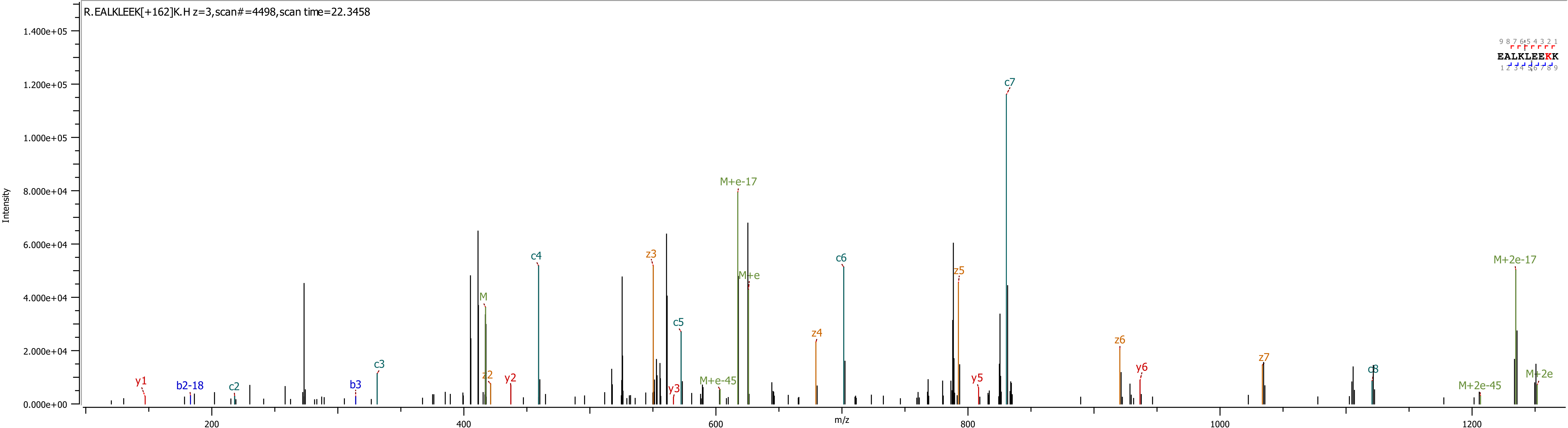
R.EALKLEEK[+162]K.H z=3,scan#=4498,scan time=22.3458
EALKLEEKK
Intensity
m/z
1.400e+05
1.200e+05
1.000e+05
8.000e+04
6.000e+04
4.000e+04
2.000e+04
0.000e+00
200
400
600
800
1000
1200
y1
b2-18
c2
b3
c3
M
z2
y2
c4
z3
y3
c5
M+e-45
M+e-17
M+e
z4
c6
z5
y5
c7
z6
y6
z7
c8
M+2e-45
M+2e-17
M+2e

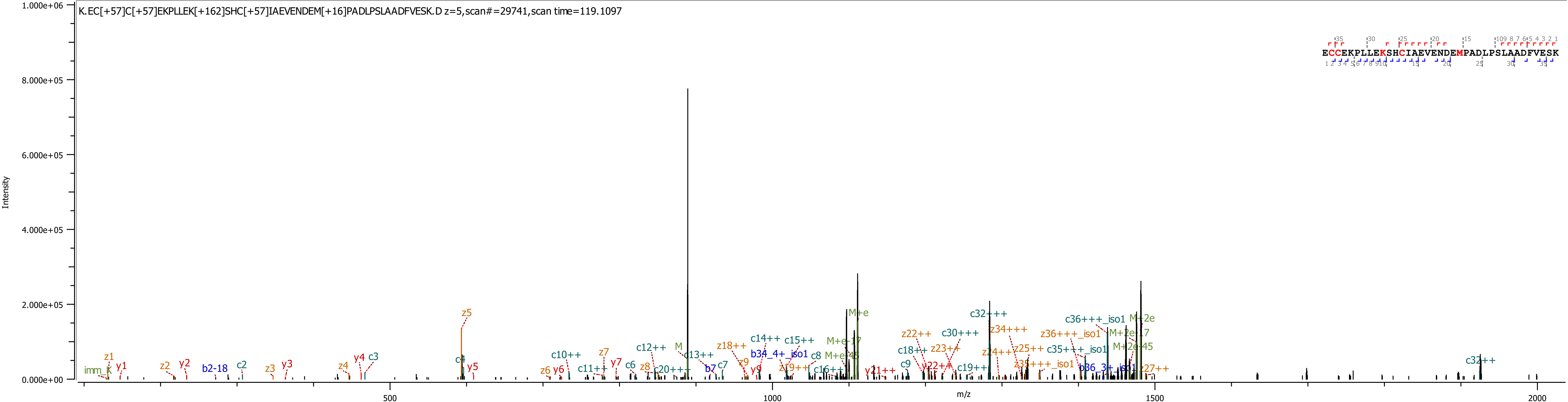
K.EC[+57]C[+57]EKPLLEK[+162]SHC[+57]IAEVENDEM[+16]PADLPSLAADFVESK.D z=5,scan#=29741,scan time=119.1097
ECCEKPLLEKSHCIAEVENDEMPADLPSLAADFVESK
Intensity
m/z
1.000e+06
8.000e+05
6.000e+05
4.000e+05
2.000e+05
0.000e+00
500
1000
1500
2000

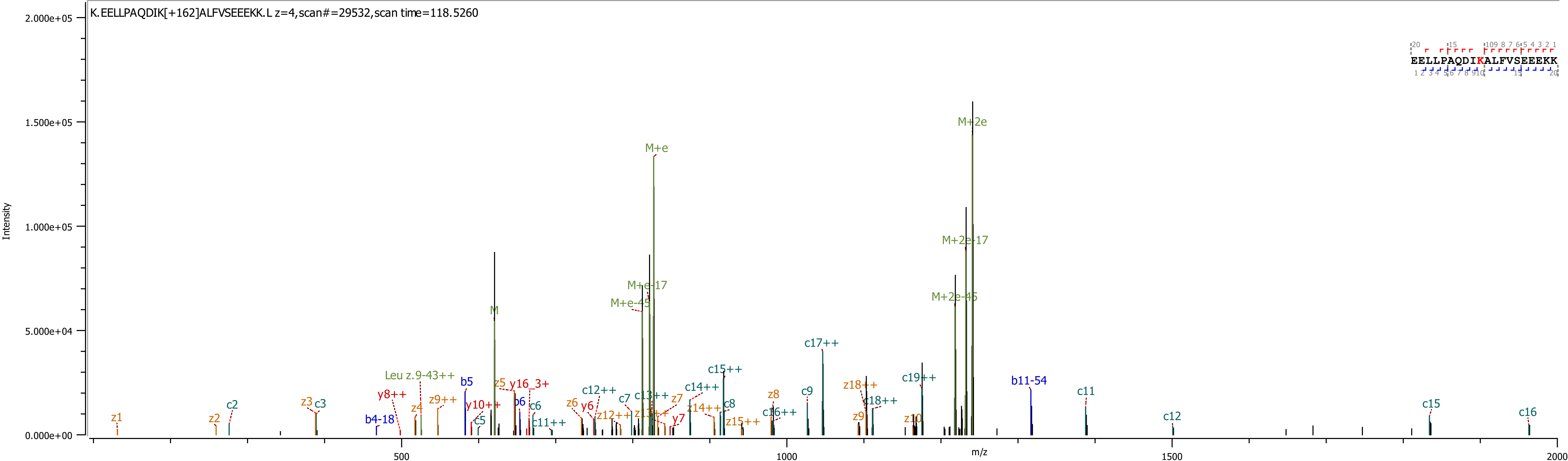
K.EELLPAQDIK[+162]ALFVSEEEKK.L z=4,scan#=29532,scan time=118.5260
EELLPAQDIKALFVSEEEKK
Intensity
m/z
2.000e+05
1.500e+05
1.000e+05
5.000e+04
0.000e+00
500
1000
1500
2000
M+2e
M+e
M+2e-17
M+e-17
M+e-45
M+2e-45
M
Leu z.9-43++
c17++
c15++
c19++
b11-54
c11
c12
c15
c16
z1
z2
c2
z3
c3
y8++
b4-18
z4
z9++
b5
y10++
c5
z5
y16_3+
b6
c6
c11++
z6
y6
c12++
z12++
c7
c13++
z13++
z7
y7
c14++
z14++
c8
z15++
z8
c16++
c9
z18++
z9
c18++
z10

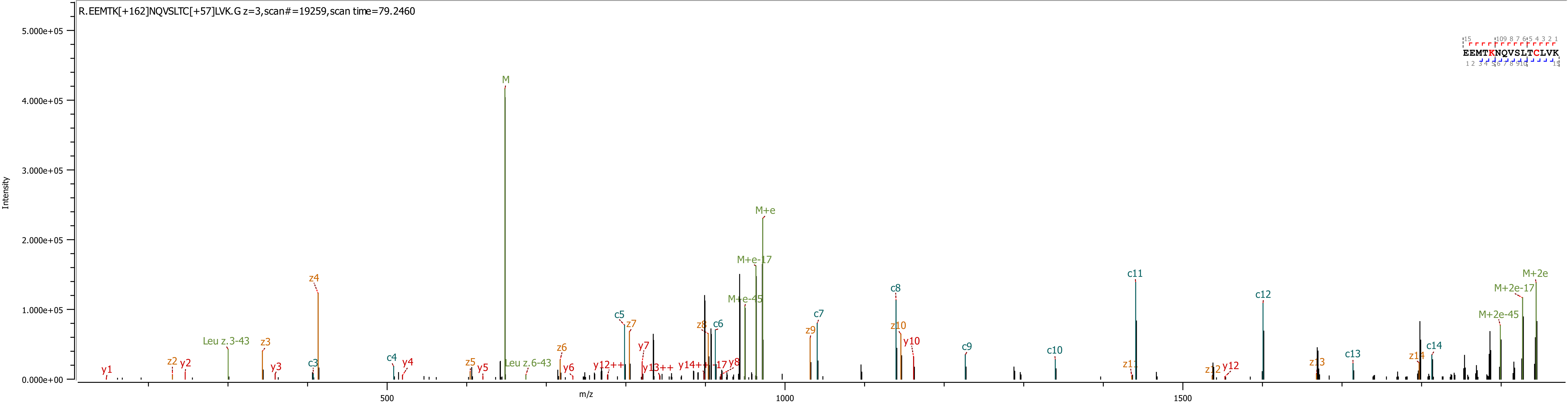
R.EEMTK[+162]NQVSLTC[+57]LVK.G z=3,scan#=19259,scan time=79.2460
Intensity
m/z
EEMTKNQVSLTCLVK
M
M+e
M+e-17
M+e-45
M+2e
M+2e-17
M+2e-45
Leu z.3-43
Leu z.6-43
y1
y2
y3
y4
y5
y6
y7
y8
y10
y12
y12++
y13++
y14++
z2
z3
z4
z5
z6
z7
z8
z9
z10
z11
z12
z13
z14
c3
c4
c5
c6
c7
c8
c9
c10
c11
c12
c13
c14
0.000e+00
1.000e+05
2.000e+05
3.000e+05
4.000e+05
5.000e+05
500
1000
1500

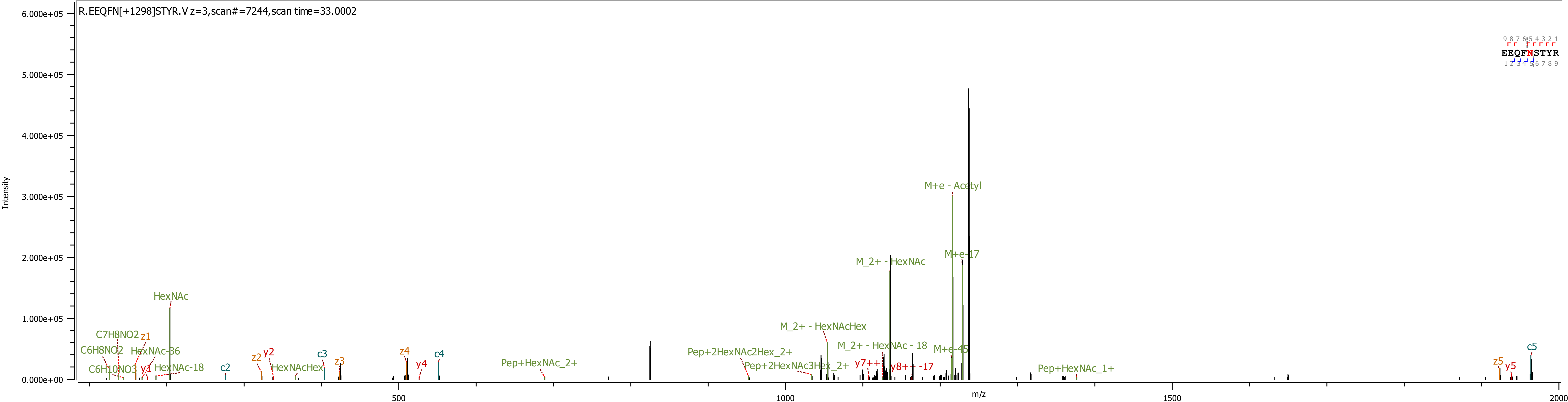

R.EEQFN[+1298]STYR.V z=3,scan#=7244,scan time=33.0002
EEQFNSTYR
Intensity
m/z
6.000e+05
5.000e+05
4.000e+05
3.000e+05
2.000e+05
1.000e+05
0.000e+00
500
1000
1500
2000
M+e - Acetyl
M_2+ - HexNAc
M+e-17
HexNAc
M_2+ - HexNAcHex
M_2+ - HexNAc - 18
M+e-45
C7H8NO2
C6H8NO2
C6H10NO3
z1
y1
HexNAc-36
HexNAc-18
c2
z2
y2
HexNAcHex
c3
z3
z4
y4
c4
Pep+HexNAc_2+
Pep+2HexNAc2Hex_2+
Pep+2HexNAc3Hex_2+
y7++
y8++ -17
Pep+HexNAc_1+
z5
y5
c5

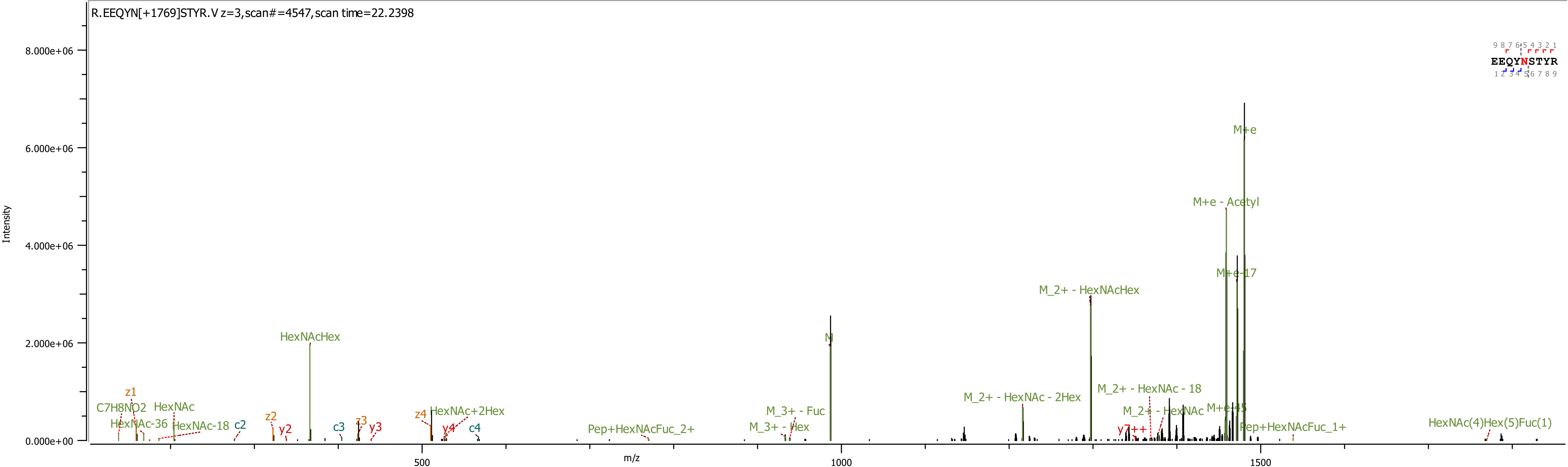

R.EEQYN[+1769]STYR.V z=3,scan#=4547,scan time=22.2398
Intensity
m/z
8.000e+06
6.000e+06
4.000e+06
2.000e+06
0.000e+00
500
1000
1500
EEQYNSTYR
M+e
M+e - Acetyl
M+e-17
M_2+ - HexNAcHex
M
HexNAcHex
M_2+ - HexNAc - 18
M_2+ - HexNAc - 2Hex
M_2+ - HexNAc
M+e-45
M_3+ - Fuc
M_3+ - Hex
Pep+HexNAcFuc_2+
Pep+HexNAcFuc_1+
HexNAc(4)Hex(5)Fuc(1)
y7++
z1
C7H8NO2
HexNAc
HexNAc-36
HexNAc-18
c2
z2
y2
c3
z3
y3
z4
HexNAc+2Hex
y4
c4

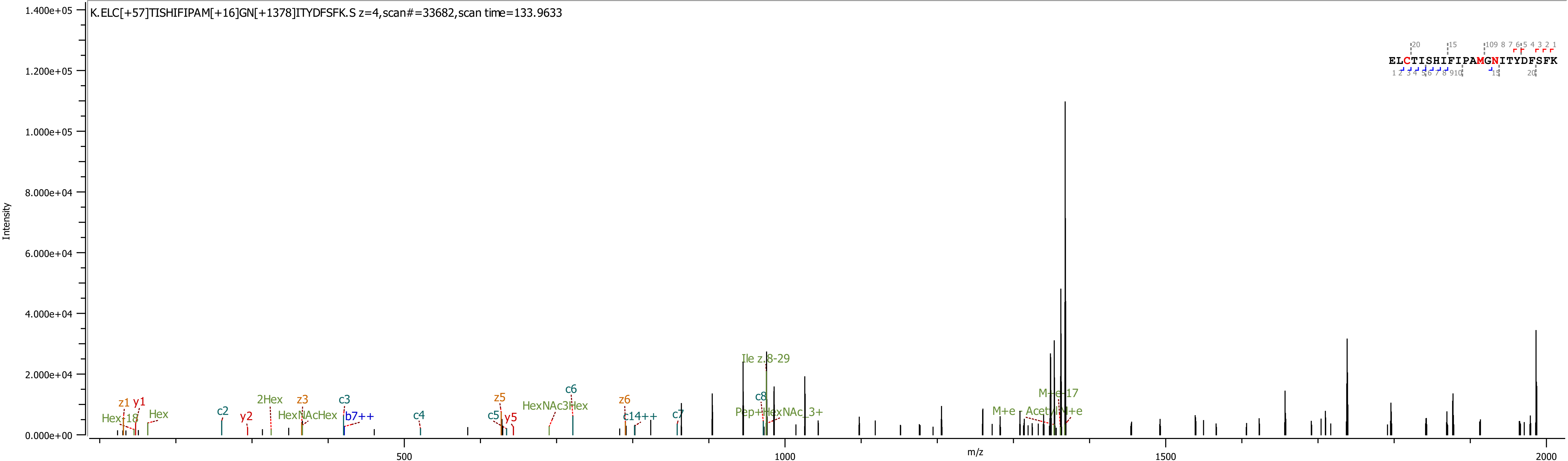
K.ELC[+57]TISHIFIPAM[+16]GN[+1378]ITYDFSFK.S z=4,scan#=33682,scan time=133.9633
ELCTISHIFIPAMGNITYDFSFK
Intensity
m/z
Hex-18
z1
y1
Hex
c2
y2
2Hex
z3
HexNAcHex
c3
b7++
c4
c5
z5
y5
HexNAc3Hex
c6
z6
c14++
c7
Ile z.8-29
c8
Pep+HexNAc_3+
M+e
M+e-17
Acetyl M+e

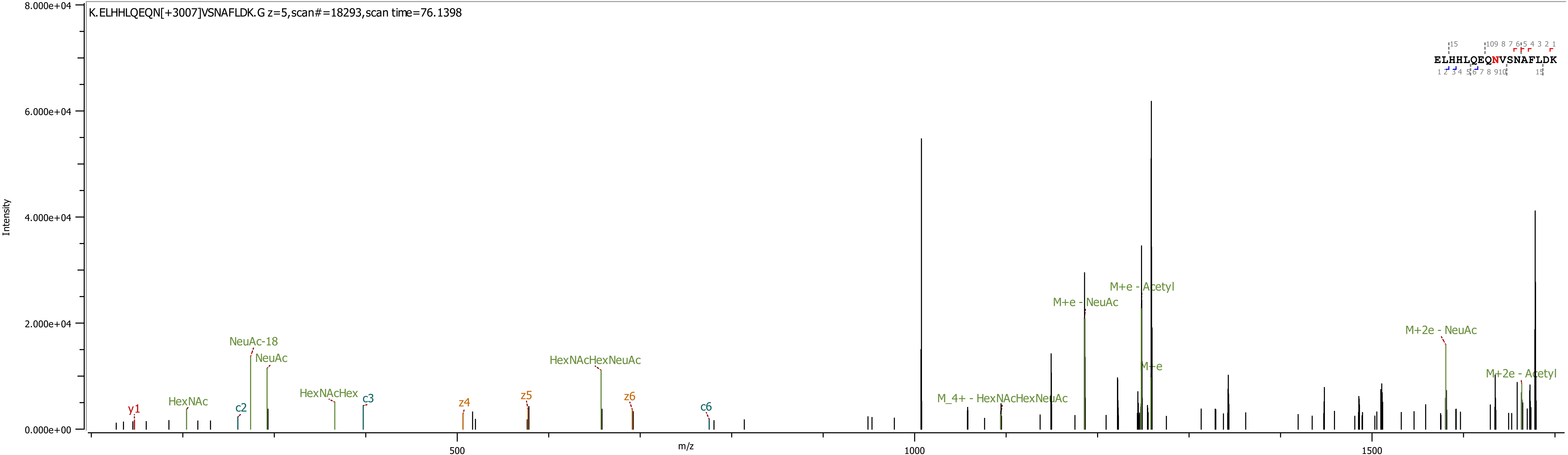
K.ELHHLQEQN[+3007]VSNAFLDK.G z=5,scan#=18293,scan time=76.1398
Intensity
m/z
8.000e+04
6.000e+04
4.000e+04
2.000e+04
0.000e+00
500
1000
1500
ELHHLQEQNVSNAFLDK
y1
HexNAc
c2
NeuAc-18
NeuAc
HexNAcHex
c3
z4
z5
HexNAcHexNeuAc
z6
c6
M_4+ - HexNAcHexNeuAc
M+e - NeuAc
M+e - Acetyl
M+e
M+2e - NeuAc
M+2e - Acetyl

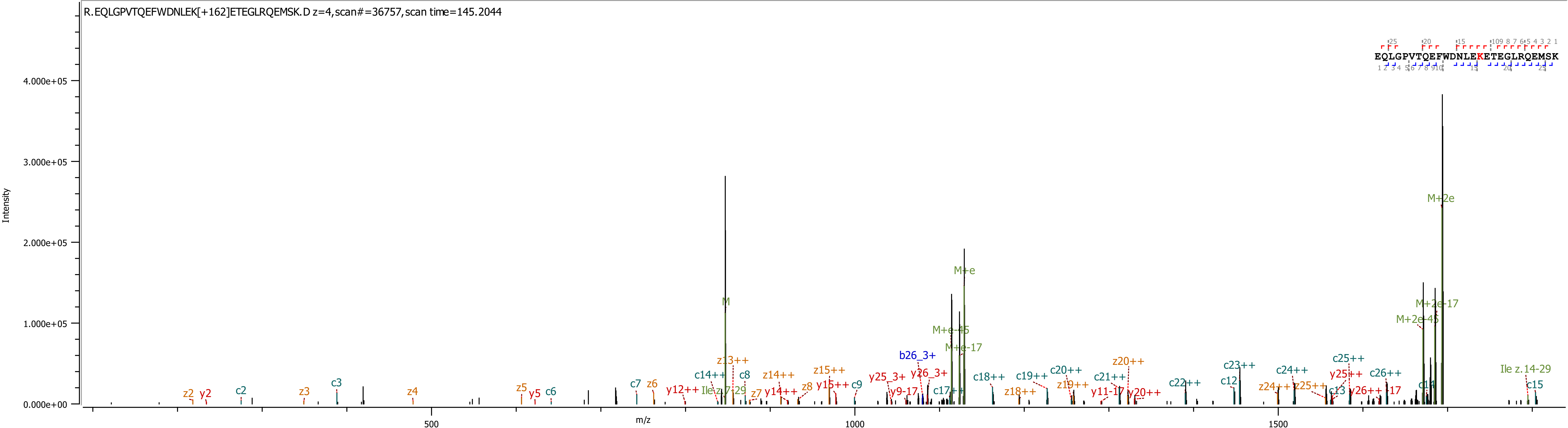

R.EQLGPVTQEFWDNLEK[+162]ETEGLRQEMSK.D z=4,scan#=36757,scan time=145.2044
EQLGPVTQEFWDNLEKETEGLRQEMSK
Intensity
m/z
M
M+e
M+2e
M+2e-17
M+2e-45
M+e-45
M+e-17
b26_3+

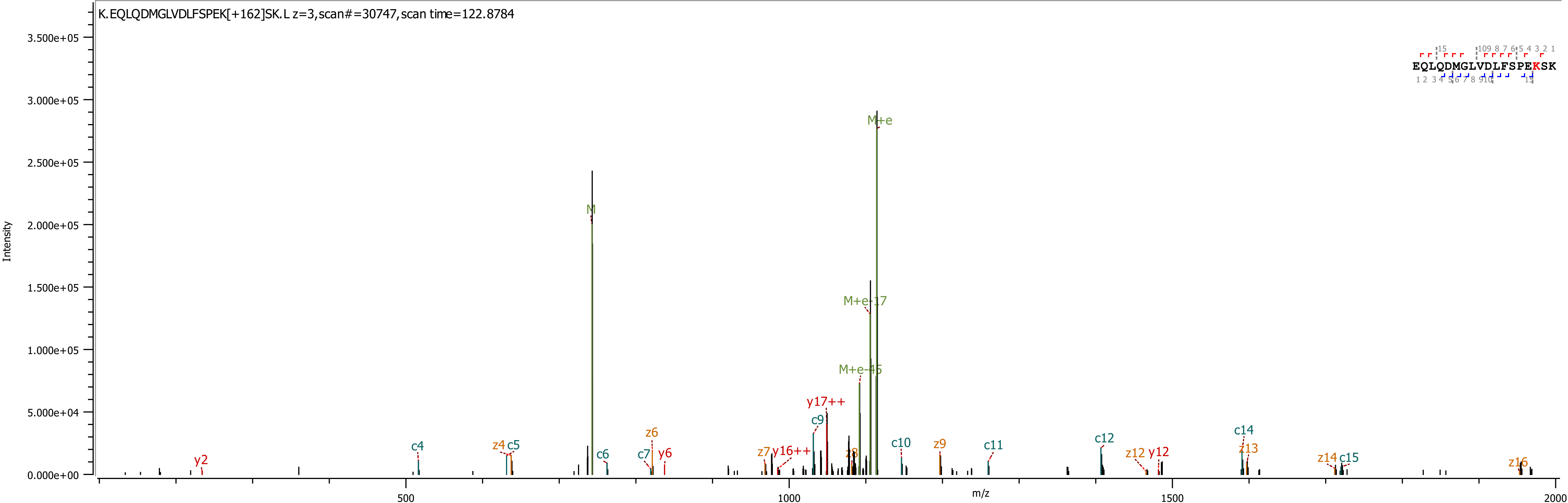
K.EQLQDMGLVDLFSPEK[+162]SK.L z=3,scan#=30747,scan time=122.8784
Intensity
m/z
EQLQDMGLVDLFSPEKSK
M+e
M
M+e-17
M+e-45
y17++
c9
y16++
y2
c4
z4
c5
c6
c7
z6
y6
z7
z8
c10
z9
c11
c12
z12
y12
c14
z13
z14
c15
z16

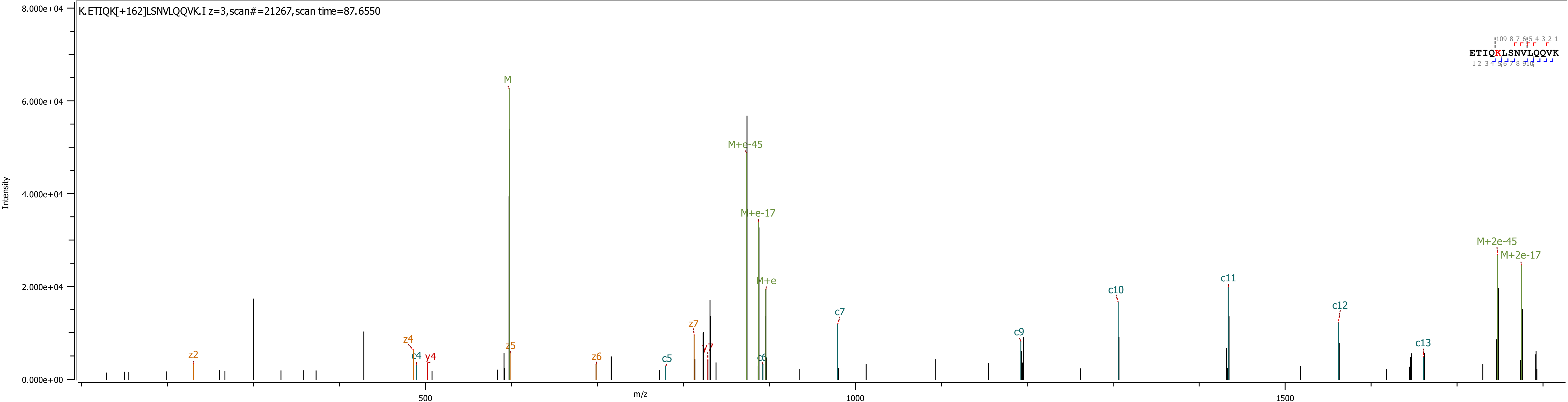

K.ETIQK[+162]LSNVLQQVK.I z=3,scan#=21267,scan time=87.6550
ETIQKLSNVLQQVK
Intensity
m/z
8.000e+04
6.000e+04
4.000e+04
2.000e+04
0.000e+00
500
1000
1500
M
M+e-45
M+e-17
M+e
M+2e-45
M+2e-17
z2
z4
z5
z6
z7
c4
c5
c6
c7
c9
c10
c11
c12
c13
y4
y7

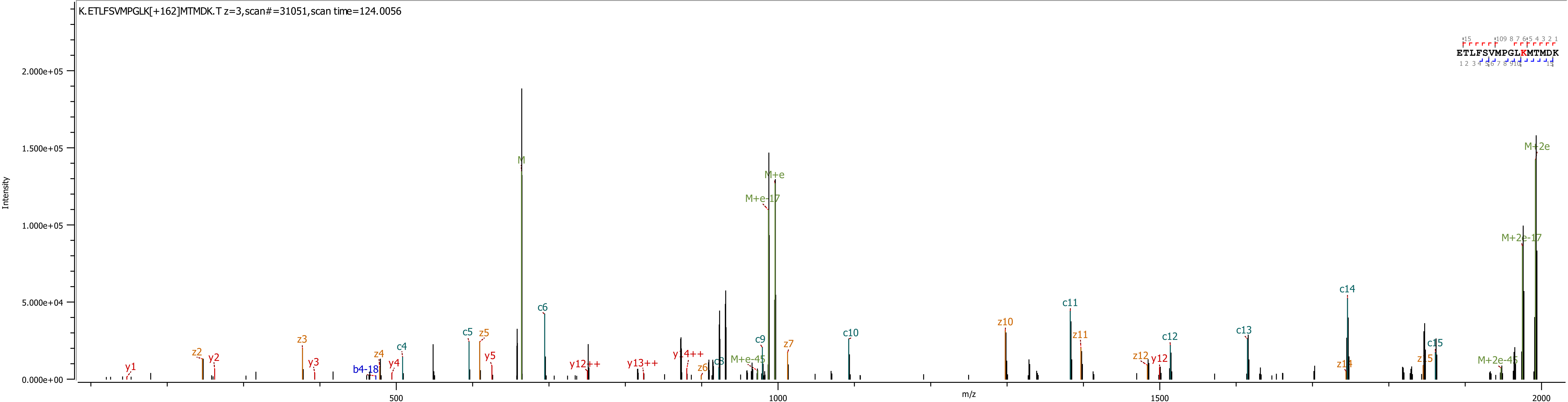
K.ETLFSVMPGLK[+162]MTMDK.T z=3,scan#=31051,scan time=124.0056
ETLFSVMPGLKMTMDK
Intensity
m/z
2.000e+05
1.500e+05
1.000e+05
5.000e+04
0.000e+00
500
1000
1500
2000
y1
z2
y2
z3
y3
b4-18
z4
y4
c4
c5
z5
y5
M
c6
y12++
y13++
y14++
z6
c8
M+e-45
M+e-17
c9
M+e
z7
c10
z10
c11
z11
z12
y12
c12
c13
c14
z14
z15
c15
M+2e-45
M+2e-17
M+2e

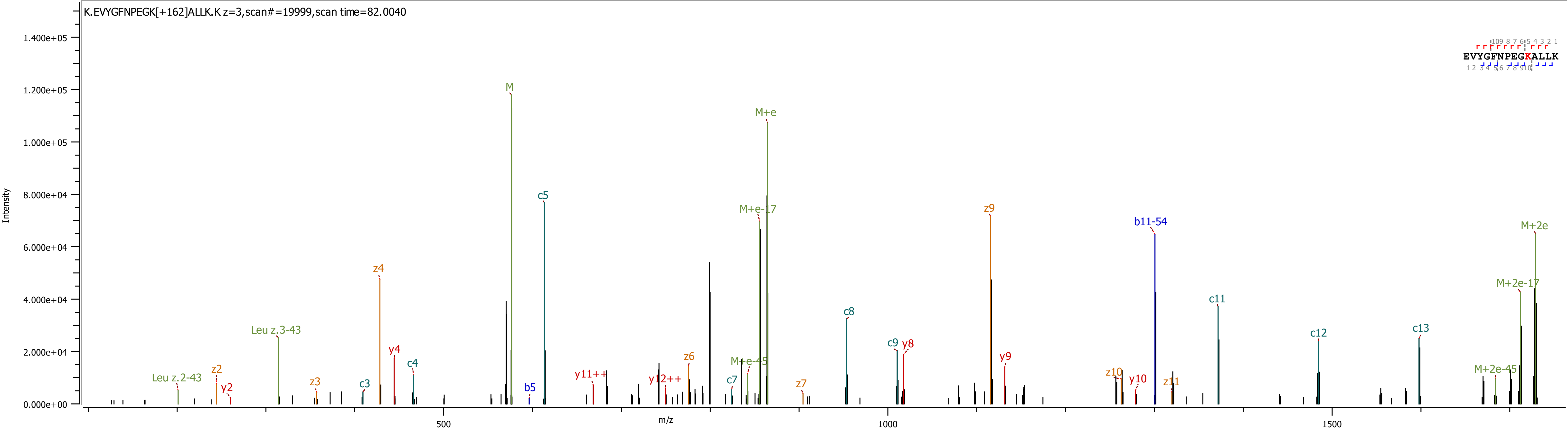
K.EVYGFNPEGK[+162]ALLK.K z=3,scan#=19999,scan time=82.0040
EVYGFNPEGKALLK
Intensity
m/z
0.000e+00
2.000e+04
4.000e+04
6.000e+04
8.000e+04
1.000e+05
1.200e+05
1.400e+05
500
1000
1500
M
M+e
M+e-17
M+e-45
M+2e
M+2e-17
M+2e-45
Leu z.2-43
Leu z.3-43
z2
z3
z4
z6
z7
z9
z10
z11
y2
y4
y8
y9
y10
y11++
y12++
c3
c4
c5
c7
c8
c9
c11
c12
c13
b5
b11-54

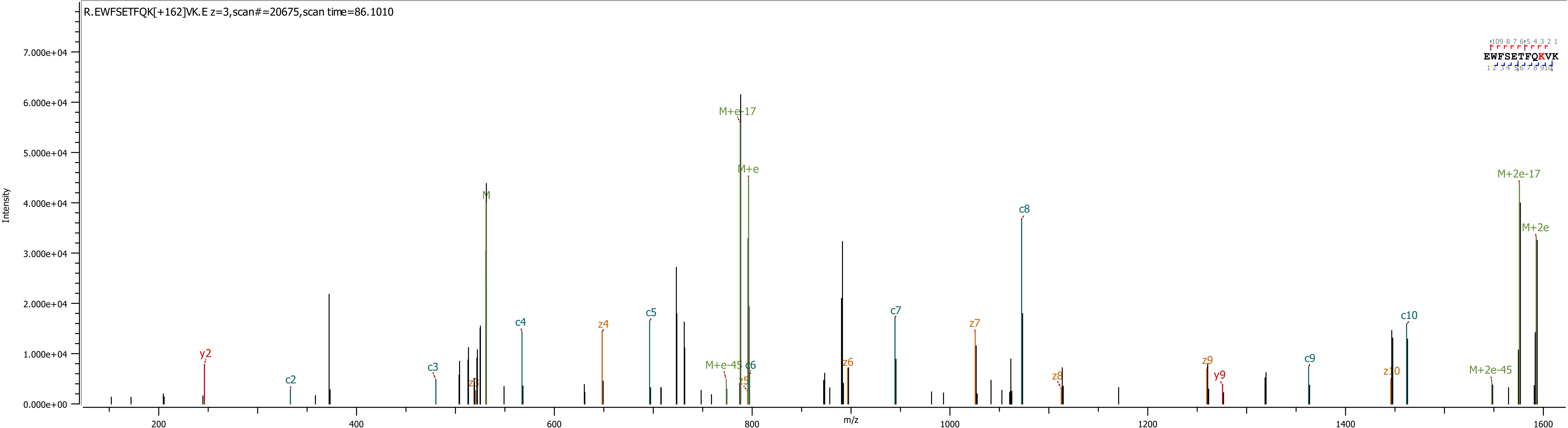
R.EWFSETFQK[+162]VK.E z=3,scan#=20675,scan time=86.1010
Intensity
7.000e+04
6.000e+04
5.000e+04
4.000e+04
3.000e+04
2.000e+04
1.000e+04
0.000e+00
m/z
200
400
600
800
1000
1200
1400
1600
EWFSETFQKVK
M+e-17
M+e
M
M+2e-17
M+2e
c8
c7
c10
c9
c5
c4
c3
c2
c6
z4
z7
z6
z9
z10
z8
z3
z5
y2
y9
M+e-45
M+2e-45

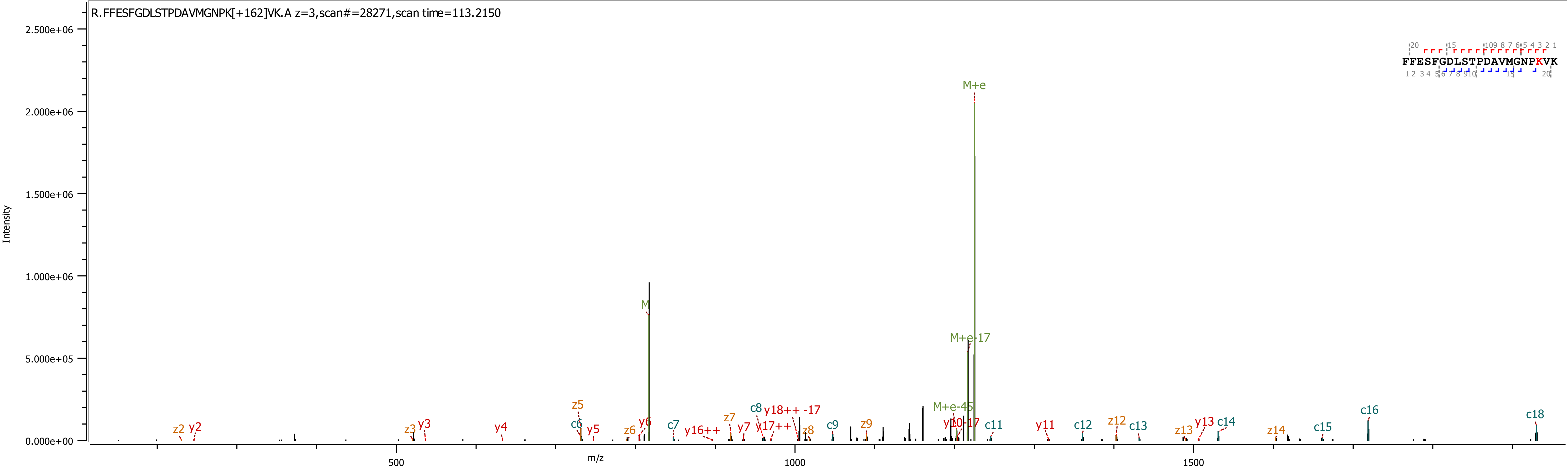
R.FFESFGDLSTPDAVMGNPK[+162]VK.A z=3,scan#=28271,scan time=113.2150
FFESFGDLSTPDAVMGNPKVK
Intensity
m/z
M+e
M
M+e-17
M+e-45
c6
c7
c8
c9
c11
c12
c13
c14
c15
c16
c18
z2
z3
z5
z6
z7
z8
z9
z12
z13
z14
y2
y3
y4
y5
y6
y7
y11
y13
y16++
y17++
y18++ -17
y10-17
500
1000
1500
0.000e+00
5.000e+05
1.000e+06
1.500e+06
2.000e+06
2.500e+06

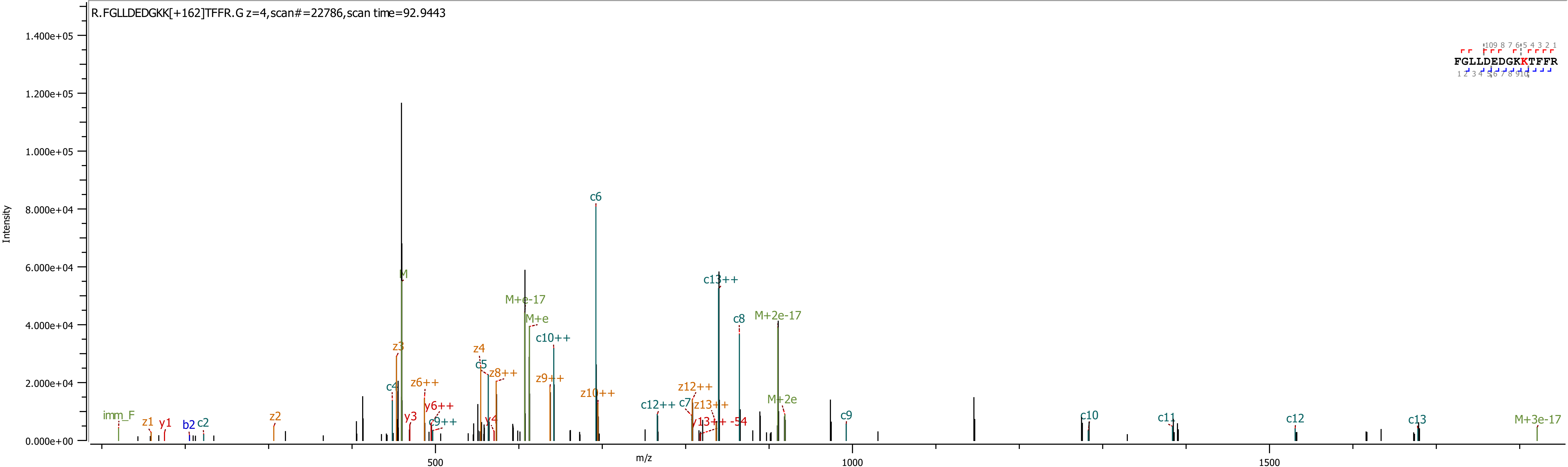
R.FGLLDEDGKK[+162]TFFR.G z=4,scan#=22786,scan time=92.9443
Intensity
m/z
1.400e+05
1.200e+05
1.000e+05
8.000e+04
6.000e+04
4.000e+04
2.000e+04
0.000e+00
500
1000
1500
FGLLDEDGKKTFFR
M
M+e-17
M+e
M+2e-17
M+2e
M+3e-17
imm_F
c6
c13++
c8
c10++
c4
c5
c7
c9
c9++
c10
c11
c12
c12++
c13
c2
b2
z1
z2
z3
z4
z6++
z8++
z9++
z10++
z12++
z13++
y1
y3
y4
y6++
y13++ -54

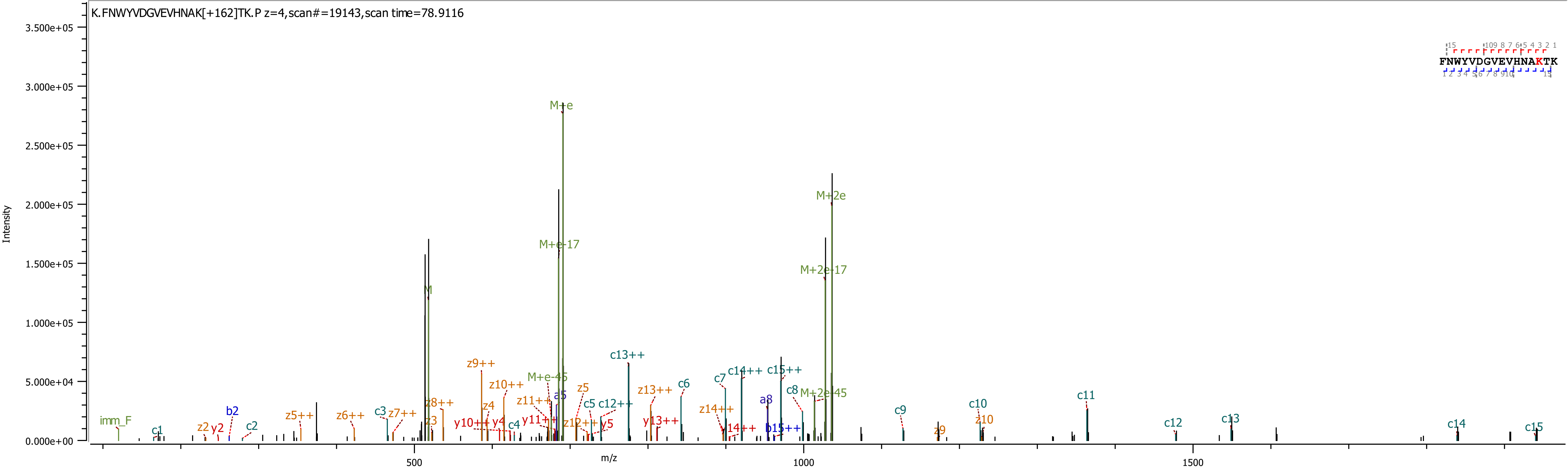
K.FNWYVDGVEVHNAK[+162]TK.P z=4,scan#=19143,scan time=78.9116
FNWYVDGVEVHNAKTK
Intensity
m/z
3.500e+05
3.000e+05
2.500e+05
2.000e+05
1.500e+05
1.000e+05
5.000e+04
0.000e+00
500
1000
1500
M+e
M+e-17
M+e-45
M
M+2e
M+2e-17
M+2e-45
imm_F
c1
c2
c3
c4
c5
c6
c7
c8
c9
c10
c11
c12
c13
c14
c15
c12++
c13++
c14++
c15++
b2
a5
a8
b15++
z2
z3
z4
z5
z9
z10
z5++
z6++
z7++
z8++
z9++
z10++
z11++
z12++
z13++
z14++
y2
y4
y5
y10++
y11++
y13++
y14++

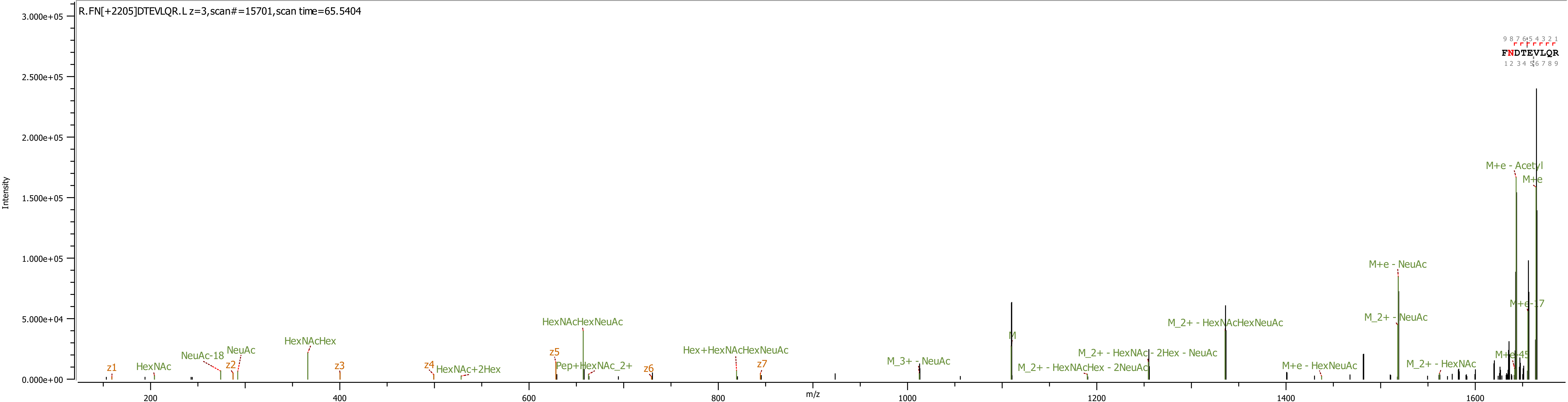
R.FN[+2205]DTEVLQR.L z=3,scan#=15701,scan time=65.5404
FNDTEVLQR
Intensity
m/z
z1
HexNAc
NeuAc-18
NeuAc
z2
HexNAcHex
z3
z4
HexNAc+2Hex
HexNAcHexNeuAc
z5
Pep+HexNAc_2+
z6
Hex+HexNAcHexNeuAc
z7
M_3+ - NeuAc
M
M_2+ - HexNAcHex - 2NeuAc
M_2+ - HexNAc - 2Hex - NeuAc
M_2+ - HexNAcHexNeuAc
M+e - HexNeuAc
M+e - NeuAc
M_2+ - NeuAc
M_2+ - HexNAc
M+e - Acetyl
M+e
M+e-17
M+e-45

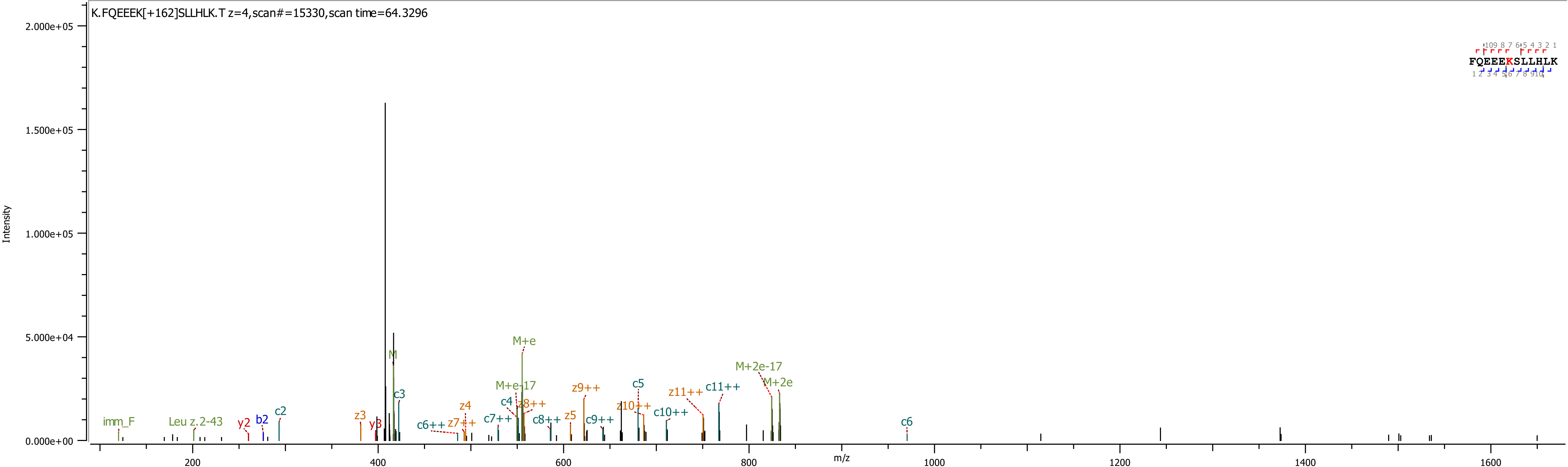

K.FQEEEK[+162]SLLHLK.T z=4,scan#=15330,scan time=64.3296
FQEEEKSLLHLK
Intensity
2.000e+05
1.500e+05
1.000e+05
5.000e+04
0.000e+00
m/z
200
400
600
800
1000
1200
1400
1600
imm_F
Leu z.2-43
y2
b2
c2
z3
y3
M
c3
c6++
z7++
z4
c7++
M+e-17
c4
M+e
z8++
c8++
z5
z9++
c9++
z10++
c5
c10++
z11++
c11++
M+2e-17
M+2e
c6

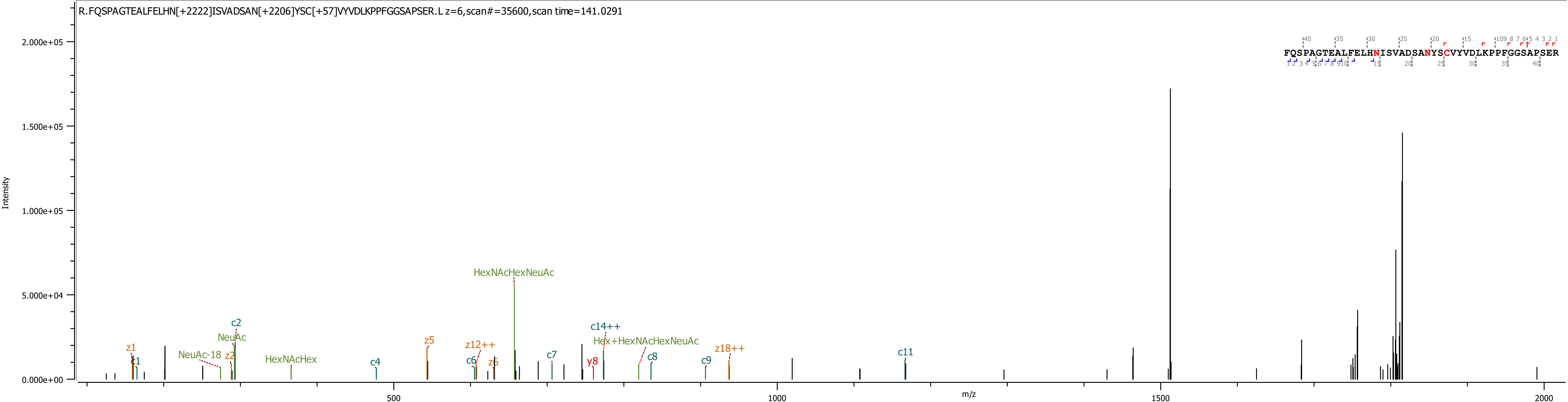

R.FQSPAGTEALFELHN[+2222]ISVADSAN[+2206]YSC[+57]VYVDLKPPFGGSAPSER.L z=6,scan#=35600,scan time=141.0291
FQSPAGTEALFELHNISVADSANYSCVYVDLKPPFGGSAPSER
Intensity
m/z
HexNAcHexNeuAc
c14++
Hex+HexNAcHexNeuAc
z18++
c11
c2
NeuAc
NeuAc-18
HexNAcHex
z1
c1
z2
c4
z5
z12++
c6
z6
c7
y8
c8
c9

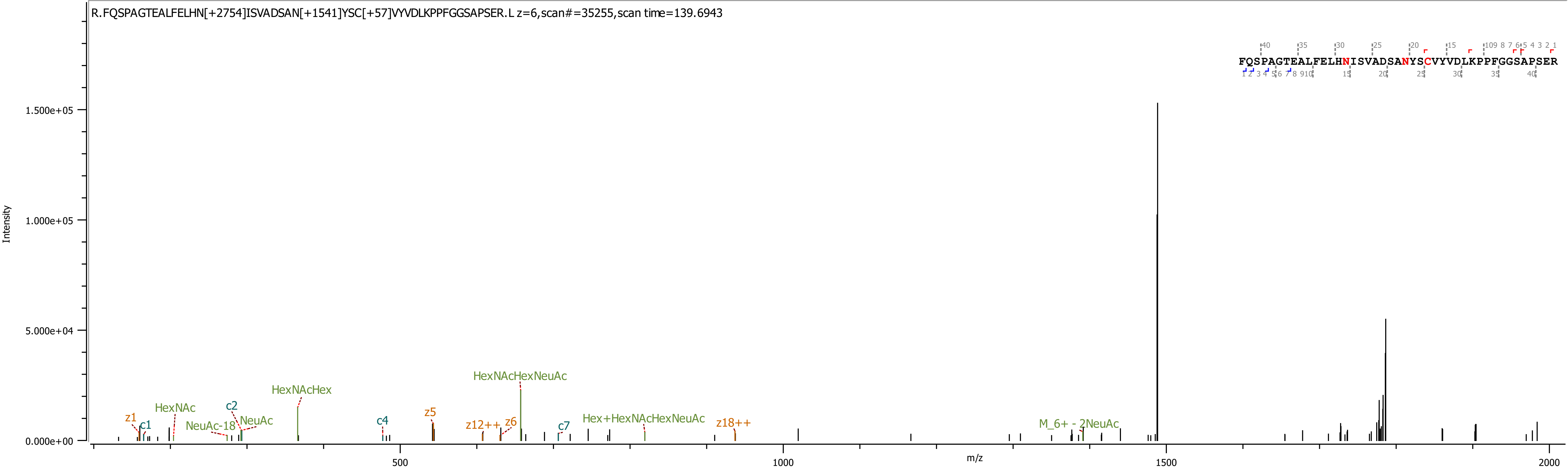

R.FQSPAGTEALFELHN[+2754]ISVADSAN[+1541]YSC[+57]VYVDLKPPFGGSAPSER.L z=6,scan#=35255,scan time=139.6943
FQSPAGTEALFELHNISVADSANYSCVYVDLKPPFGGSAPSER
Intensity
m/z
z1
c1
HexNAc
NeuAc-18
c2
NeuAc
HexNAcHex
c4
z5
z12++
z6
HexNAcHexNeuAc
c7
Hex+HexNAcHexNeuAc
z18++
M_6+ - 2NeuAc

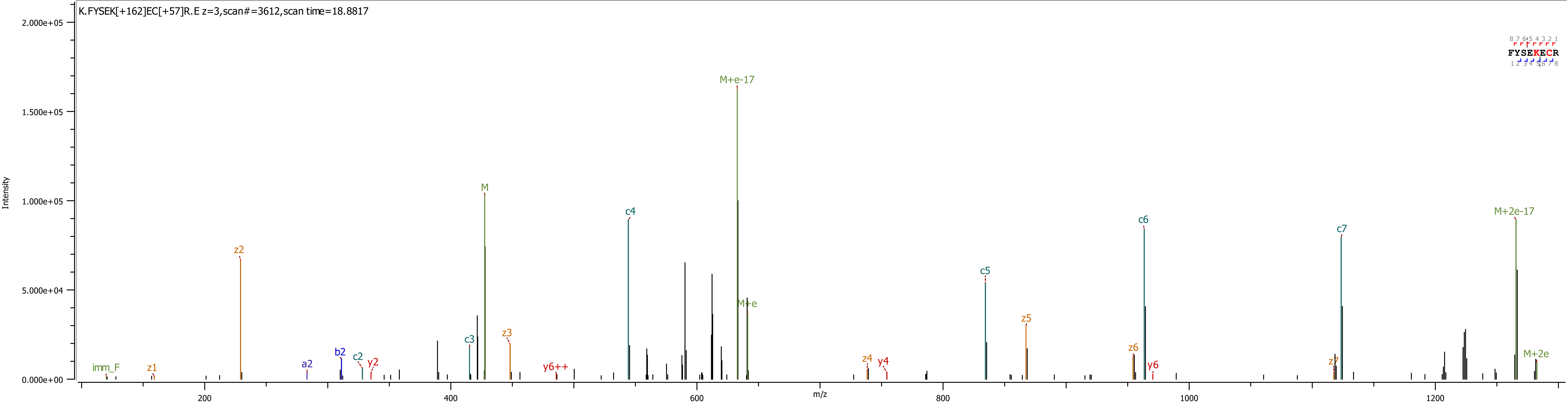
K.FYSEK[+162]EC[+57]R.E z=3,scan#=3612,scan time=18.8817
Intensity
m/z
FYSEKECR
imm_F
z1
z2
a2
b2
c2
y2
c3
M
z3
y6++
c4
M+e-17
M+e
z4
y4
c5
z5
z6
c6
y6
c7
z7
M+2e-17
M+2e
2.000e+05
1.500e+05
1.000e+05
5.000e+04
0.000e+00
200
400
600
800
1000
1200

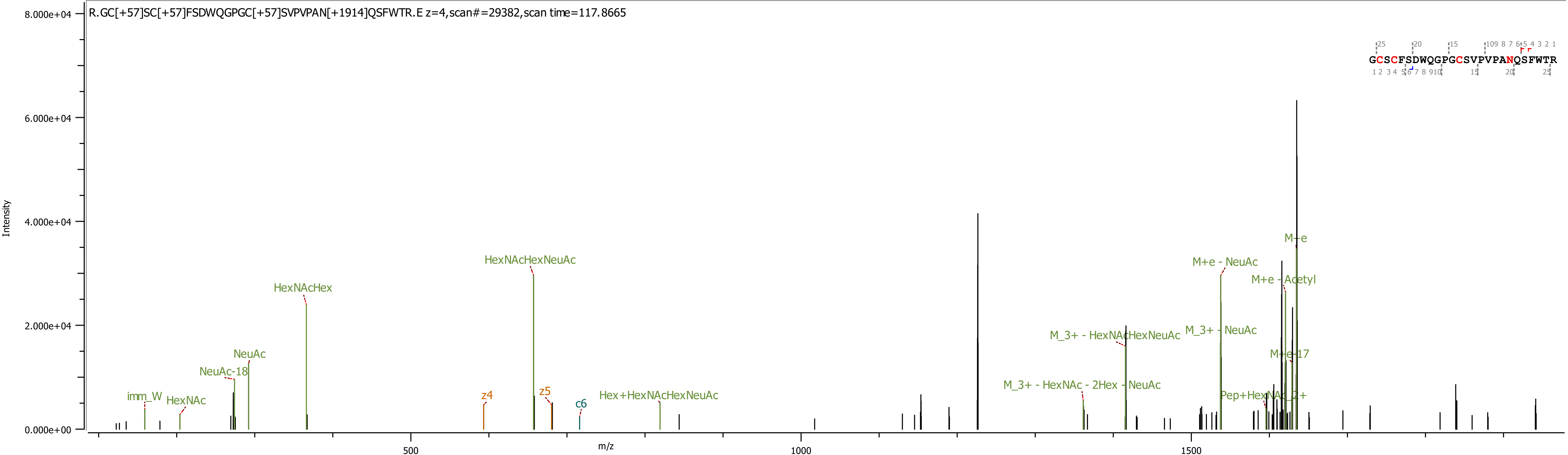
R.GC[+57]SC[+57]FSDWQGPGC[+57]SVPVPAN[+1914]QSFWTR.E z=4,scan#=29382,scan time=117.8665
GCSCFSDWQGPGCSVPVPANQSFWTR
Intensity
8.000e+04
6.000e+04
4.000e+04
2.000e+04
0.000e+00
m/z
500
1000
1500
imm_W
HexNAc
NeuAc-18
NeuAc
HexNAcHex
z4
HexNAcHexNeuAc
z5
c6
Hex+HexNAcHexNeuAc
M_3+ - HexNAc - 2Hex - NeuAc
M_3+ - HexNAcHexNeuAc
M_3+ - NeuAc
M+e - NeuAc
M+e - Acetyl
M+e
M+e-17
Pep+HexNAc_2+

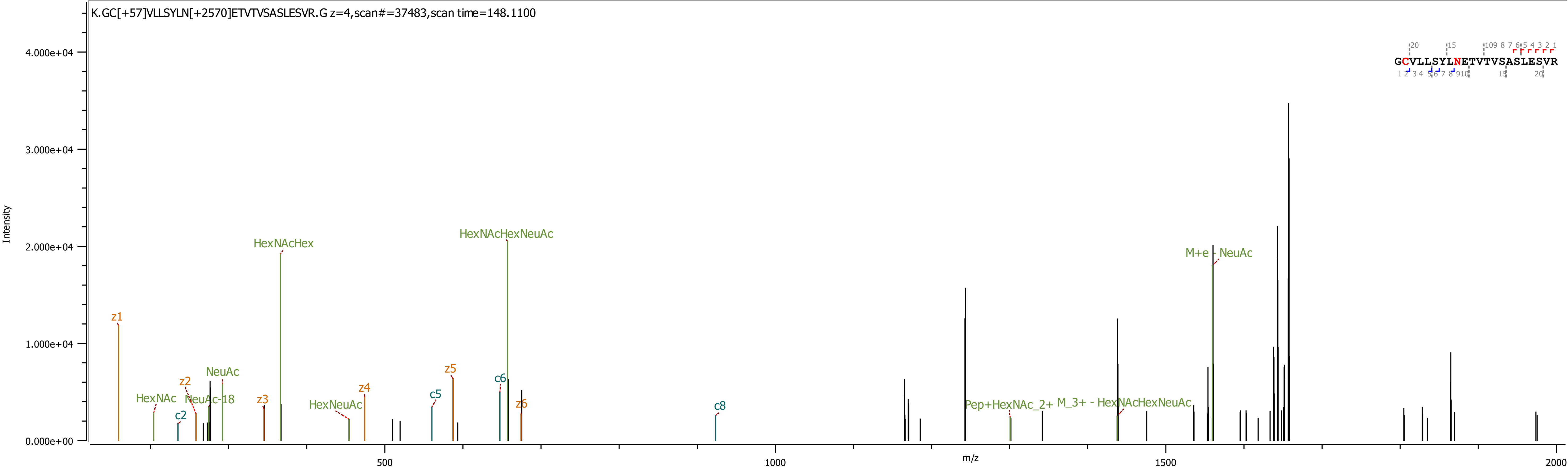
K.GC[+57]VLLSYLN[+2570]ETVTVSASLESVR.G z=4,scan#=37483,scan time=148.1100
GCVLLSYLNETVTVSASLESVR
Intensity
m/z
z1
HexNAc
c2
z2
NeuAc-18
NeuAc
z3
HexNAcHex
HexNeuAc
z4
c5
z5
c6
HexNAcHexNeuAc
z6
c8
Pep+HexNAc_2+
M_3+ - HexNAcHexNeuAc
M+e - NeuAc

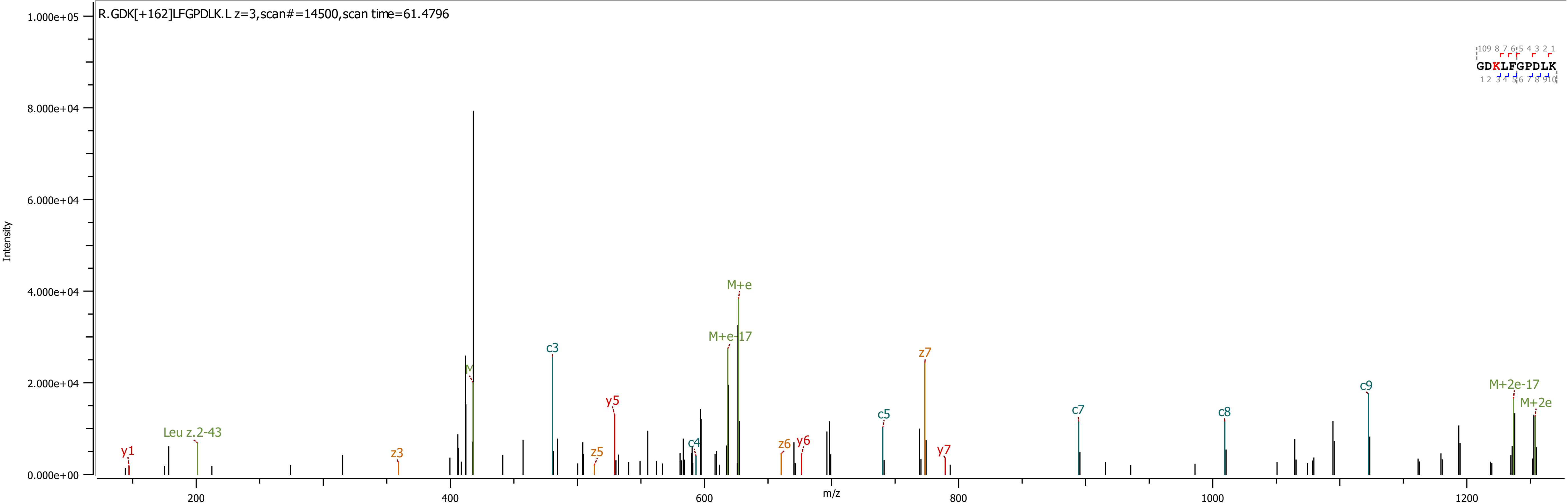

R.GDK[+162]LFGPDLK.L z=3,scan#=14500,scan time=61.4796
Intensity
m/z
GDKLFGPDLK
y1
Leu z.2-43
z3
c3
y5
z5
c4
M+e
M+e-17
z6
y6
c5
z7
y7
c7
c8
c9
M+2e-17
M+2e

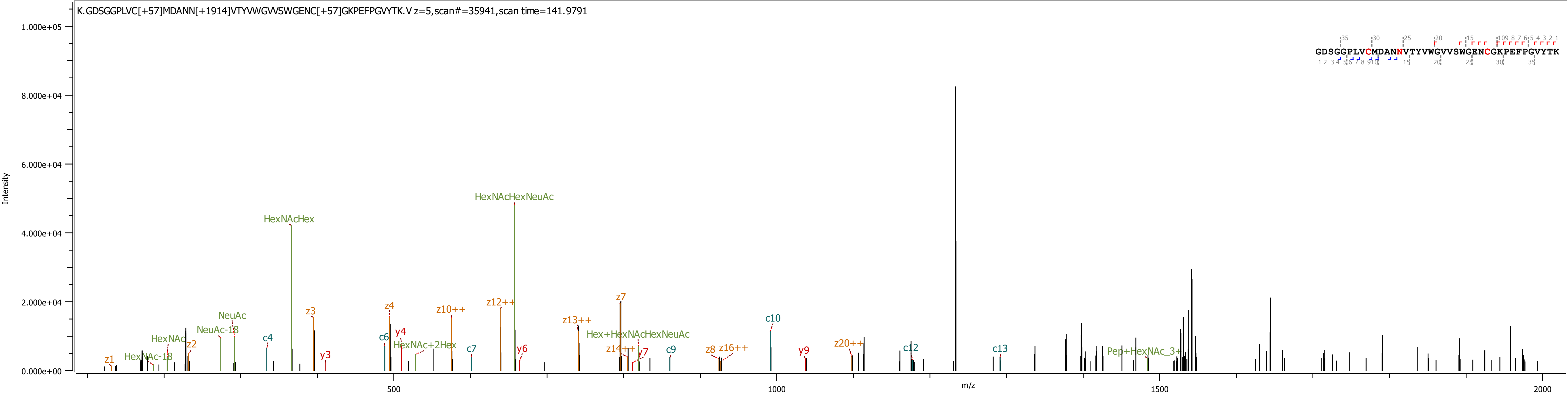
K.GDSGGPLVC[+57]MDANN[+1914]VTYVWGVVSWGENC[+57]GKPEFPGVYTK.V z=5,scan#=35941,scan time=141.9791
GDSGGPLVCMDANNVTYVWGVVSWGENCGKPEFPGVYTK
Intensity
m/z
HexNAcHexNeuAc
HexNAcHex
z7
z12++
z10++
z4
z3
c10
NeuAc
NeuAc-18
HexNAc
HexNAc-18
z13++
Hex+HexNAcHexNeuAc
c4
c6
y4
HexNAc+2Hex
z2
z1
y3
c7
y6
z14++
y7
c9
z8
z16++
y9
z20++
c12
c13
Pep+HexNAc_3+

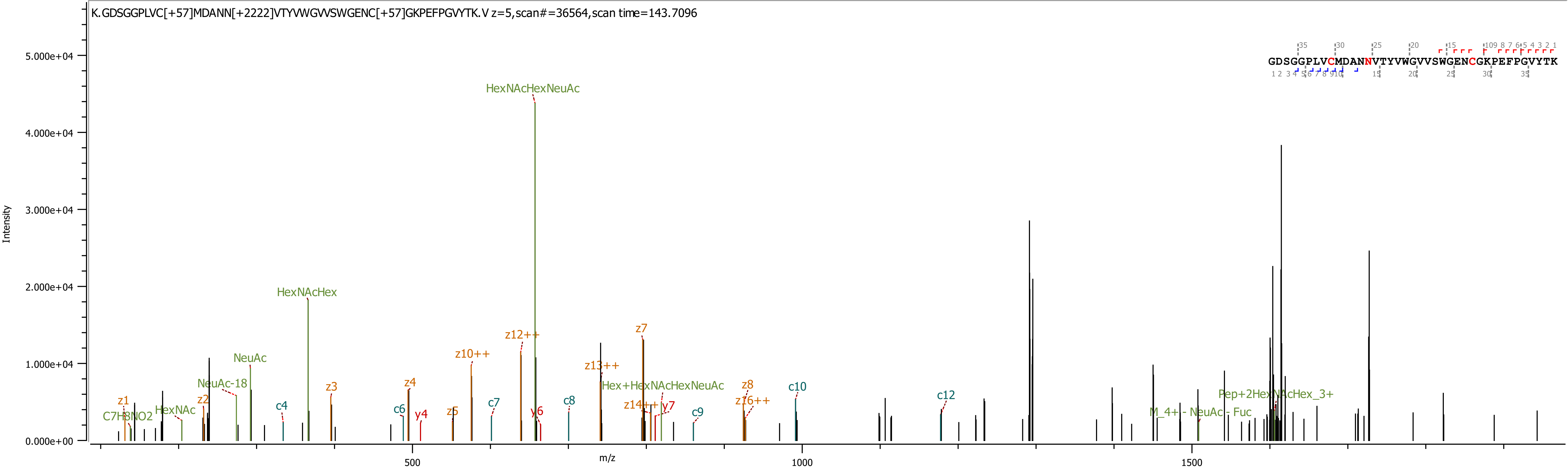
K.GDSGGPLVC[+57]MDANN[+2222]VTYVWGVVSWGENC[+57]GKPEFPGVYTK.V z=5,scan#=36564,scan time=143.7096
GDSGGPLVCMDANNVTYVWGVVSWGENCGKPEFPGVYTK
Intensity
m/z
0.000e+00
1.000e+04
2.000e+04
3.000e+04
4.000e+04
5.000e+04
500
1000
1500
HexNAcHexNeuAc
HexNAcHex
NeuAc
NeuAc-18
HexNAc
C7H8NO2
z1
z2
z3
z4
z5
z7
z8
z10++
z12++
z13++
z14++
z16++
c4
c6
c7
c8
c9
c10
c12
y4
y6
y7
Hex+HexNAcHexNeuAc
Pep+2HexNAcHex_3+
M_4+ - NeuAc - Fuc

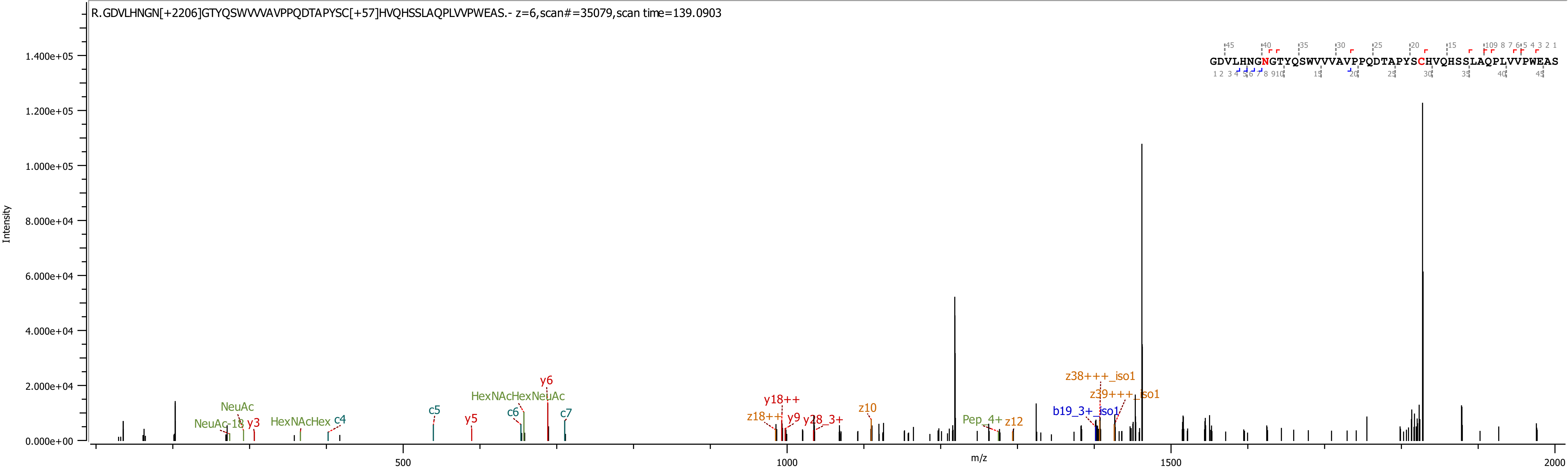

R.GDVLHNGN[+2206]GTYQSWVVVAVPPQDTAPYSC[+57]HVQHSSLAQPLVVPWEAS.- z=6,scan#=35079,scan time=139.0903
GDVLHNGNGTYQSWVVVAVPPQDTAPYSCHVQHSSLAQPLVVPWEAS
Intensity
m/z
1.400e+05
1.200e+05
1.000e+05
8.000e+04
6.000e+04
4.000e+04
2.000e+04
0.000e+00
500
1000
1500
2000
NeuAc
NeuAc-18
y3
HexNAcHex
c4
c5
y5
c6
HexNAcHexNeuAc
y6
c7
y18++
z18++
y9
y28_3+
z10
Pep_4+
z12
b19_3+_iso1
z38+++_iso1
z39+++_iso1

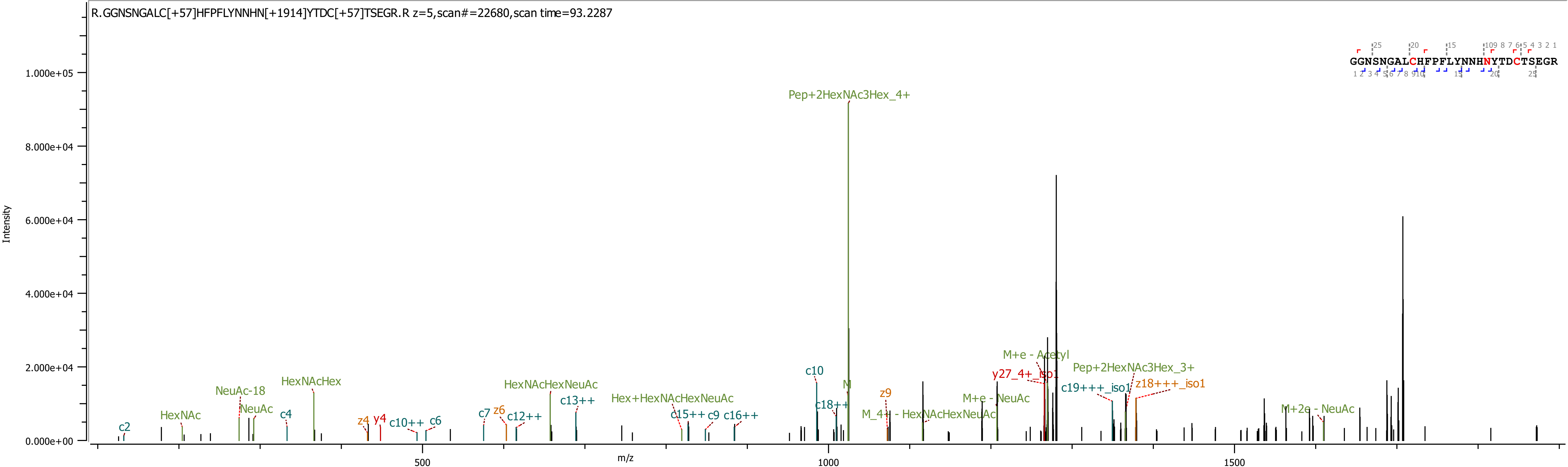
R.GGNSNGALC[+57]HFPFLYNNHN[+1914]YTDC[+57]TSEGR.R z=5,scan#=22680,scan time=93.2287
GGNSNGALCHFPFLYNNHNYTDCTSEGR
Intensity
m/z
0.000e+00
2.000e+04
4.000e+04
6.000e+04
8.000e+04
1.000e+05
500
1000
1500
Pep+2HexNAc3Hex_4+
c2
HexNAc
NeuAc-18
NeuAc
c4
HexNAcHex
z4
y4
c10++
c6
c7
z6
c12++
HexNAcHexNeuAc
c13++
Hex+HexNAcHexNeuAc
c15++
c9
c16++
c10
c18++
M
z9
M_4+ - HexNAcHexNeuAc
M+e - NeuAc
M+e - Acetyl
y27_4+_iso1
Pep+2HexNAc3Hex_3+
c19+++_iso1
z18+++_iso1
M+2e - NeuAc

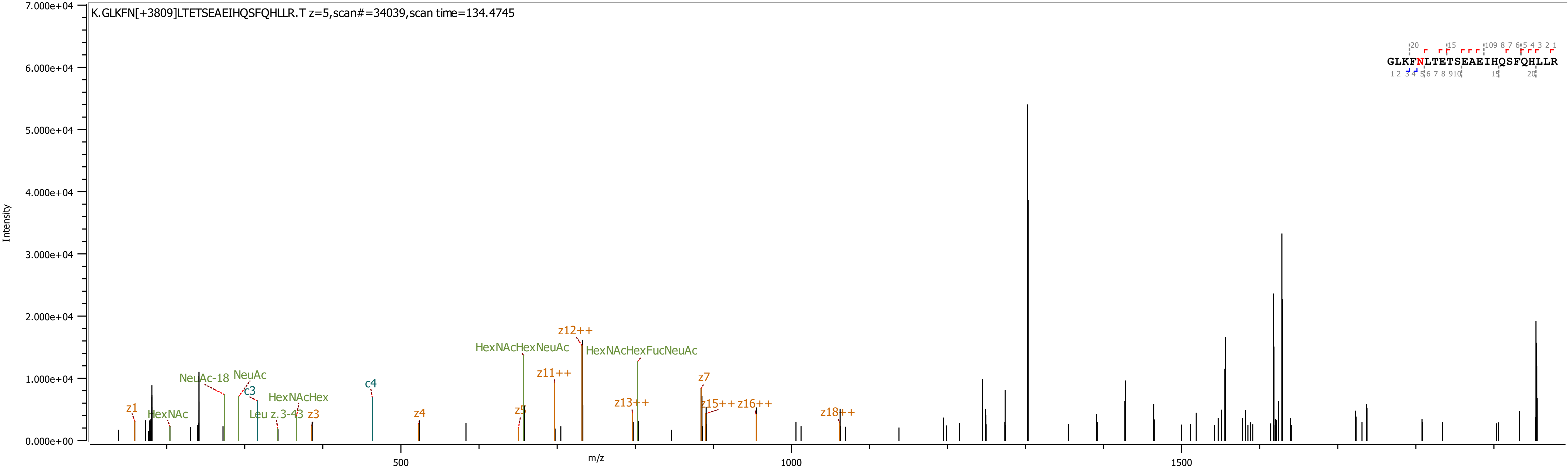
K.GLKFN[+3809]LTETSEAEIHQSFQHLLR.T z=5,scan#=34039,scan time=134.4745
GLKFNLTETSEAEIHQSFQHLLR
Intensity
7.000e+04
6.000e+04
5.000e+04
4.000e+04
3.000e+04
2.000e+04
1.000e+04
0.000e+00
m/z
500
1000
1500
z1
HexNAc
NeuAc-18
NeuAc
c3
Leu z.3-43
HexNAcHex
z3
c4
z4
z5
HexNAcHexNeuAc
z11++
z12++
HexNAcHexFucNeuAc
z13++
z7
z15++
z16++
z18++

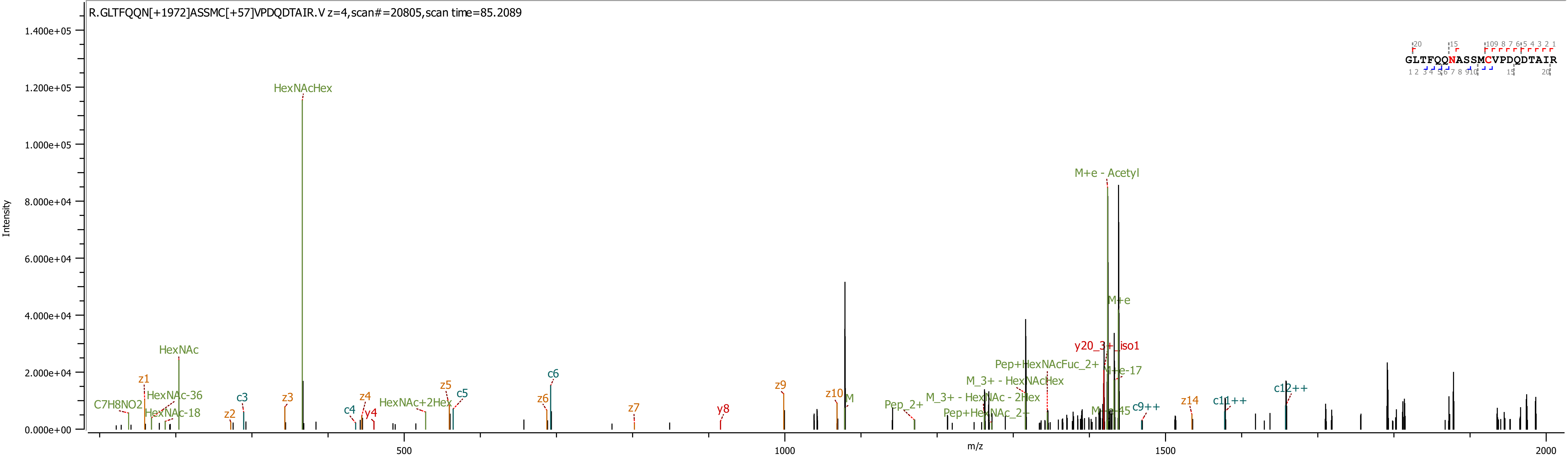
R.GLTFQQN[+1972]ASSMC[+57]VPDQDTAIR.V z=4,scan#=20805,scan time=85.2089
Intensity
1.400e+05
1.200e+05
1.000e+05
8.000e+04
6.000e+04
4.000e+04
2.000e+04
0.000e+00
m/z
500
1000
1500
2000
GLTFQQNASSMCVPDQDTAIR
HexNAcHex
M+e - Acetyl
M+e
HexNAc
C7H8NO2
z1
HexNAc-36
HexNAc-18
z2
c3
z3
c4
z4
y4
HexNAc+2Hex
z5
c5
z6
c6
z7
y8
z9
z10
M
Pep_2+
M_3+ - HexNAc - 2Hex
Pep+HexNAc_2+
M_3+ - HexNAcHex
Pep+HexNAcFuc_2+
y20_3+_iso1
M+e-17
M+e-45
c9++
z14
c11++
c12++

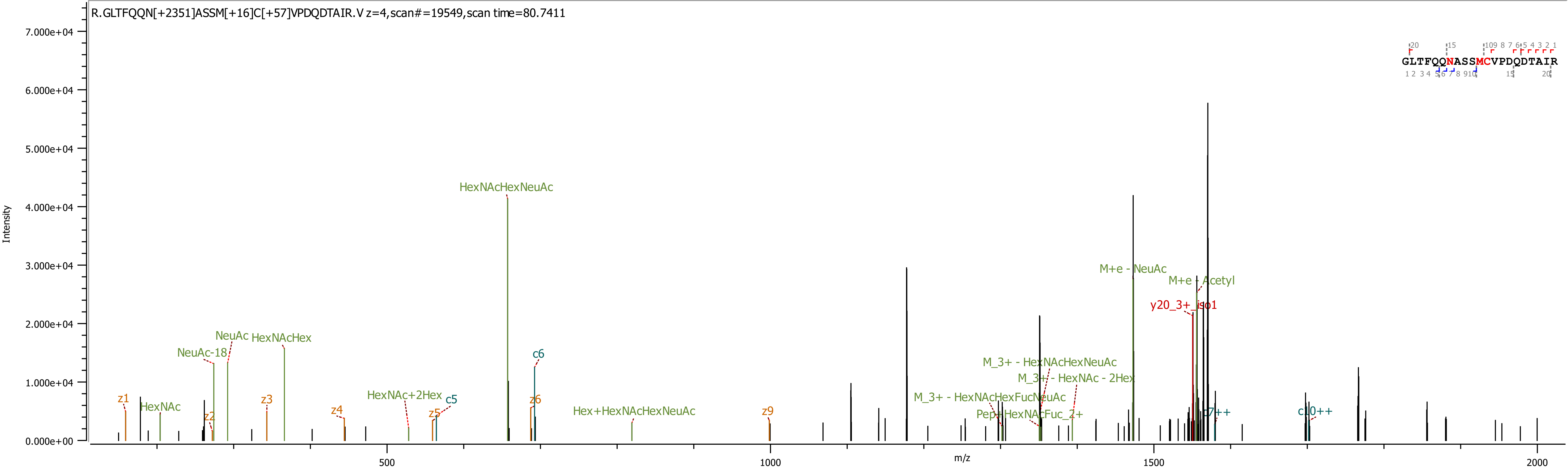
R.GLTFQQN[+2351]ASSM[+16]C[+57]VPDQDTAIR.V z=4,scan#=19549,scan time=80.7411
Intensity
m/z
HexNAcHexNeuAc
M+e - NeuAc
M+e - Acetyl
y20_3+_iso1
NeuAc
HexNAcHex
NeuAc-18
c6
M_3+ - HexNAcHexNeuAc
M_3+ - HexNAc - 2Hex
HexNAc+2Hex
c5
M_3+ - HexNAcHexFucNeuAc
z1
HexNAc
z3
z6
z2
z4
z5
Hex+HexNAcHexNeuAc
z9
Pep+HexNAcFuc_2+
c7++
c10++
GLTFQQNASSMCVPDQDTAIR

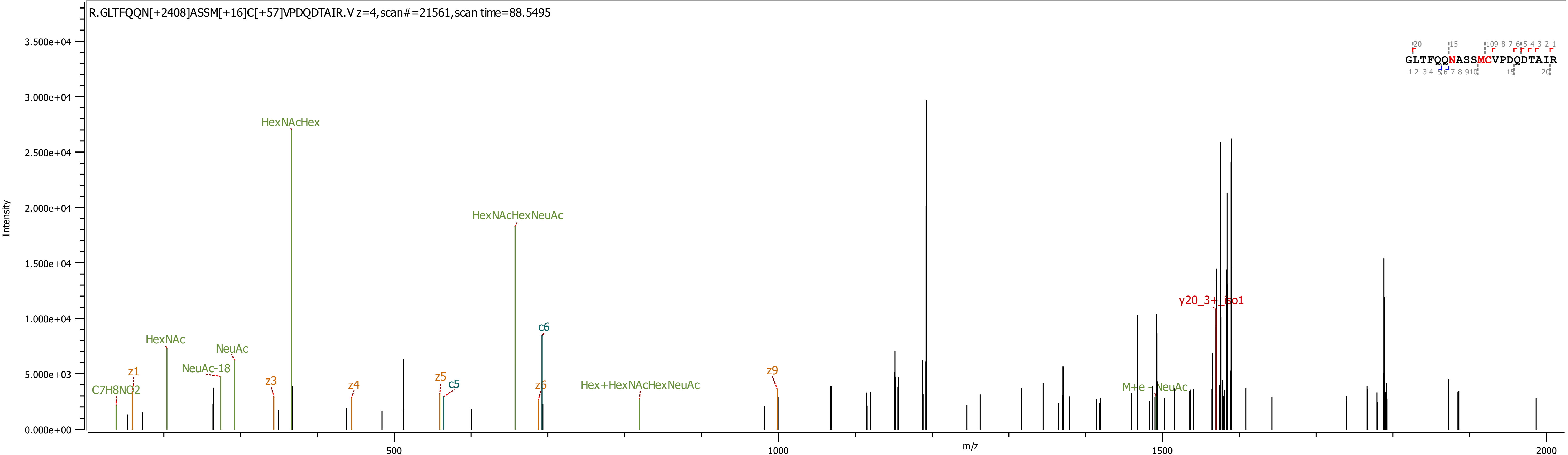

R.GLTFQQN[+2408]ASSM[+16]C[+57]VPDQDTAIR.V z=4,scan#=21561,scan time=88.5495
GLTFQQNASSMCVPDQDTAIR
Intensity
m/z
HexNAcHex
HexNAcHexNeuAc
HexNAc
NeuAc
NeuAc-18
C7H8NO2
z1
z3
z4
z5
c5
c6
z6
Hex+HexNAcHexNeuAc
z9
y20_3+_iso1
M+e - NeuAc

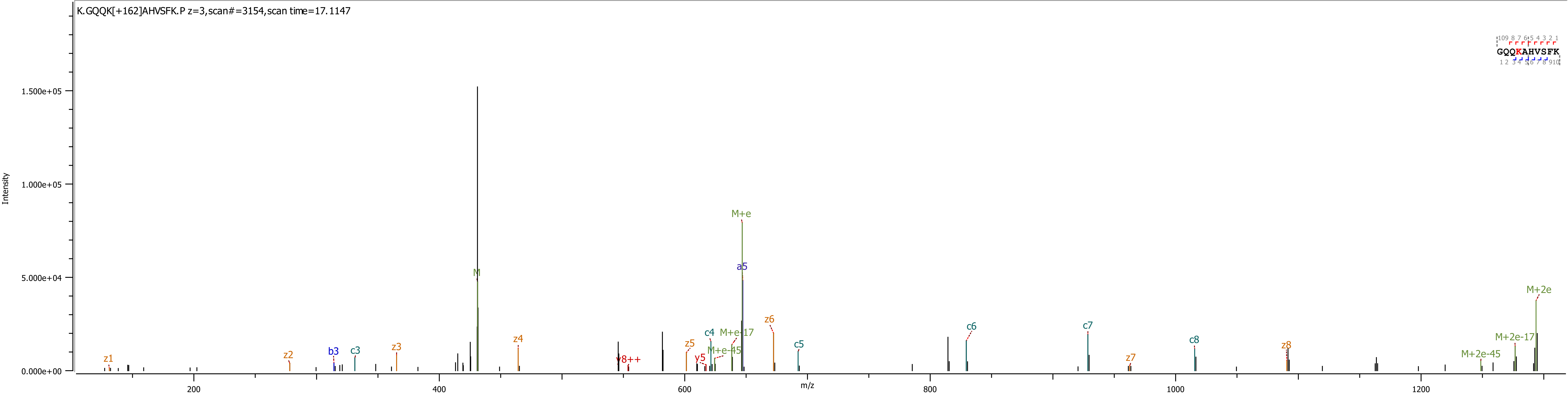
K.GQQK[+162]AHVSFK.P z=3,scan#=3154,scan time=17.1147
GQQKAHVSFK
Intensity
1.500e+05
1.000e+05
5.000e+04
0.000e+00
m/z
200
400
600
800
1000
1200
z1
z2
b3
c3
z3
M
z4
y8++
z5
y5
c4
M+e-45
M+e-17
M+e
a5
z6
c5
c6
c7
z7
c8
z8
M+2e-45
M+2e-17
M+2e

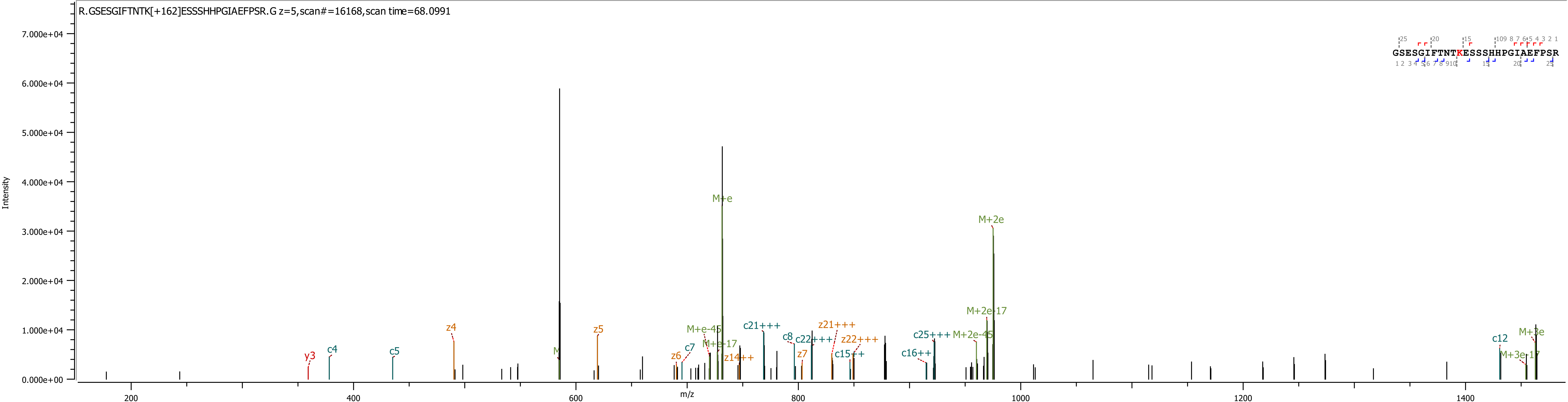

R.GSESGIFTNTK[+162]ESSSHHPGIAEFPSR.G z=5,scan#=16168,scan time=68.0991
Intensity
m/z
GSESGIFTNTKESSSHHPGIAEFPSR
M+e
M+2e
M+2e-17
M+2e-45
M+e-45
M+e-17
M+3e
M+3e-17
c21+++
c22+++
z21+++
z22+++
c25+++
c15++
c16++
z14++
y3
c4
c5
z4
z5
z6
z7
c7
c8
c12

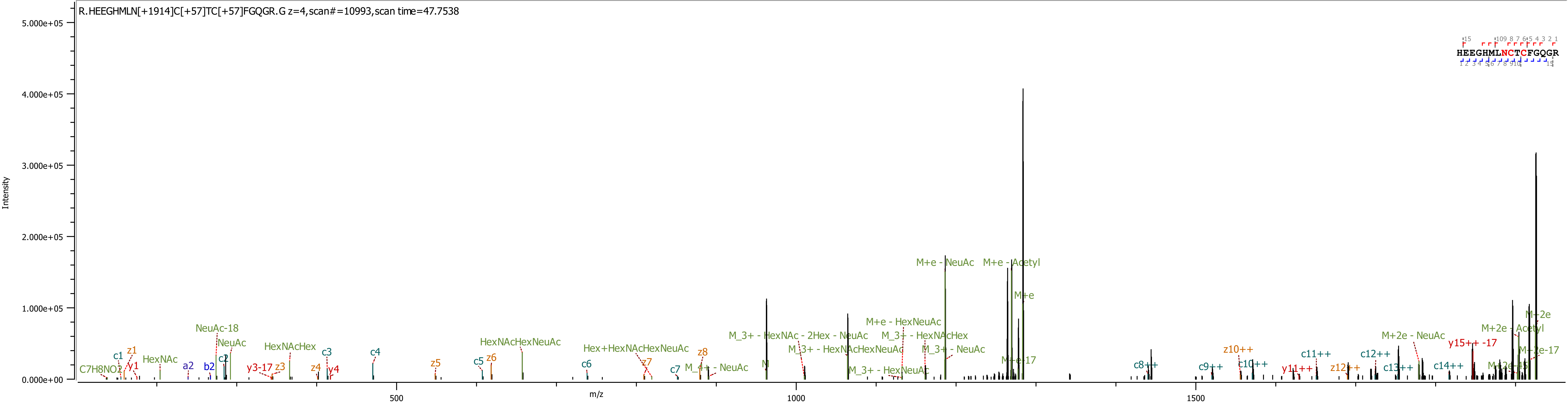
R.HEEGHMLN[+1914]C[+57]TC[+57]FGQGR.G z=4,scan#=10993,scan time=47.7538
HEEGHMLNCTCFGQGR
Intensity
m/z
5.000e+05
4.000e+05
3.000e+05
2.000e+05
1.000e+05
0.000e+00
500
1000
1500
C7H8NO2
c1
z1
y1
HexNAc
a2
b2
NeuAc-18
NeuAc
c2
y3-17
z3
HexNAcHex
z4
c3
y4
c4
z5
c5
z6
HexNAcHexNeuAc
c6
Hex+HexNAcHexNeuAc
z7
c7
z8
M_4+ - NeuAc
M
M_3+ - HexNAc - 2Hex - NeuAc
M_3+ - HexNAcHexNeuAc
M_3+ - HexNeuAc
M+e - HexNeuAc
M_3+ - HexNAcHex
M_3+ - NeuAc
M+e - NeuAc
M+e - Acetyl
M+e
M+e-17
c8++
c9++
z10++
c10++
y11++
c11++
z12++
c12++
c13++
M+2e - NeuAc
c14++
y15++ -17
M+2e - Acetyl
M+2e
M+2e-17

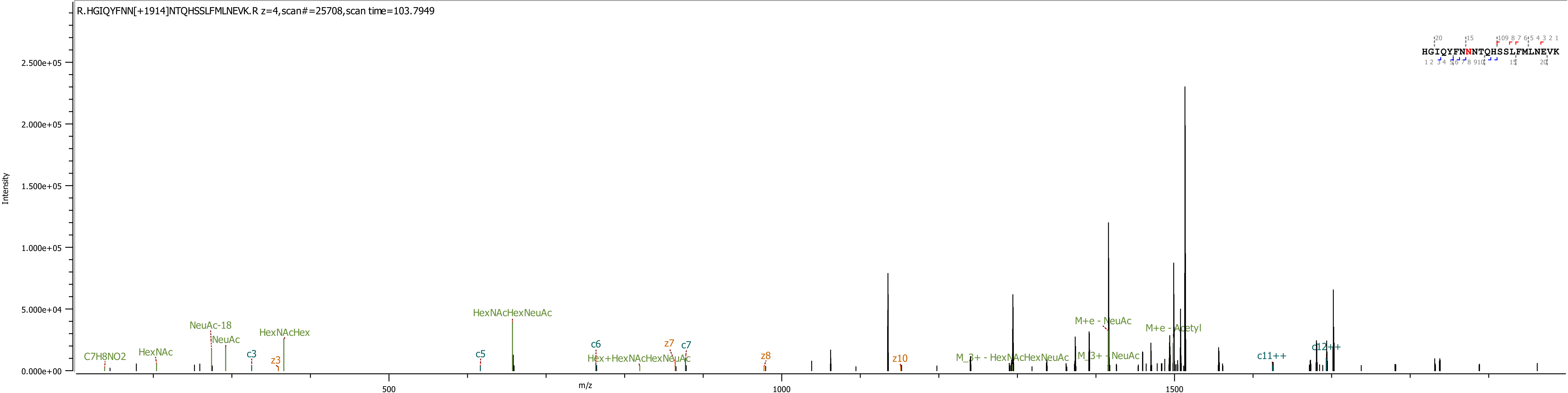
R.HGIQYFNN[+1914]NTQHSSLFMLNEVK.R z=4,scan#=25708,scan time=103.7949
HGIQYFNNNTQHSSLFMLNEVK
Intensity
m/z
C7H8NO2
HexNAc
NeuAc-18
NeuAc
c3
z3
HexNAcHex
c5
HexNAcHexNeuAc
c6
Hex+HexNAcHexNeuAc
z7
c7
z8
z10
M_3+ - HexNAcHexNeuAc
M+e - NeuAc
M_3+ - NeuAc
M+e - Acetyl
c11++
c12++

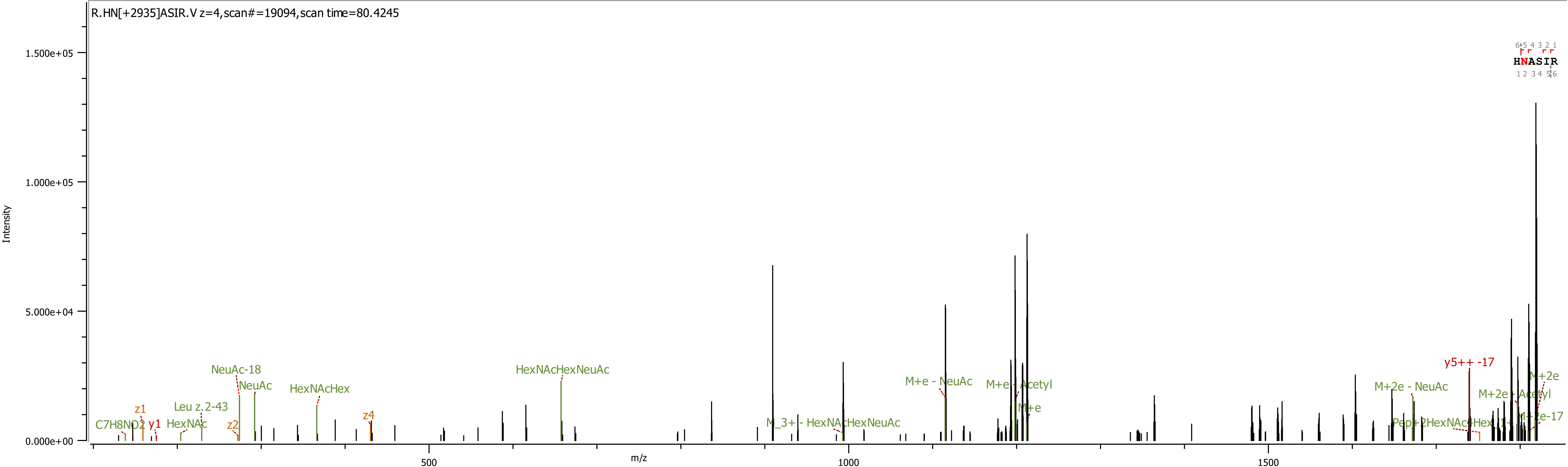

R.HN[+2935]ASIR.V z=4,scan#=19094,scan time=80.4245
Intensity
1.500e+05
1.000e+05
5.000e+04
0.000e+00
m/z
500
1000
1500
HNASIR
C7H8NO2
z1
y1
Leu z.2-43
HexNAc
z2
NeuAc-18
NeuAc
HexNAcHex
z4
HexNAcHexNeuAc
M_3+ - HexNAcHexNeuAc
M+e - NeuAc
M+e - Acetyl
M+e
M+2e - NeuAc
Pep+2HexNAc+Hex
y5++ -17
M+2e - Acetyl
M+2e
M+2e-17

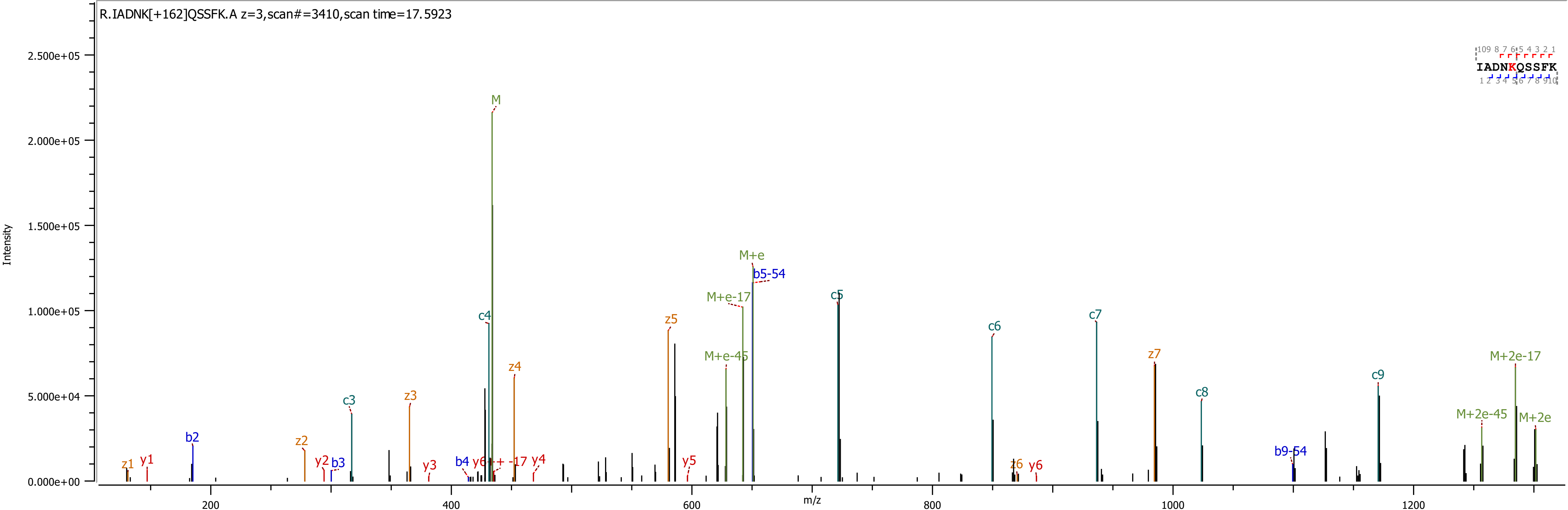

R.IADNK[+162]QSSFK.A z=3,scan#=3410,scan time=17.5923
IADNKQSSFK
Intensity
m/z
M
M+e
b5-54
M+e-17
c5
c4
z5
c6
c7
M+e-45
z4
z7
M+2e-17
c9
z3
c3
c8
M+2e-45
M+2e
b2
z2
y1
z1
y2
b3
y3
b4
y4
y5
z6
y6
b9-54

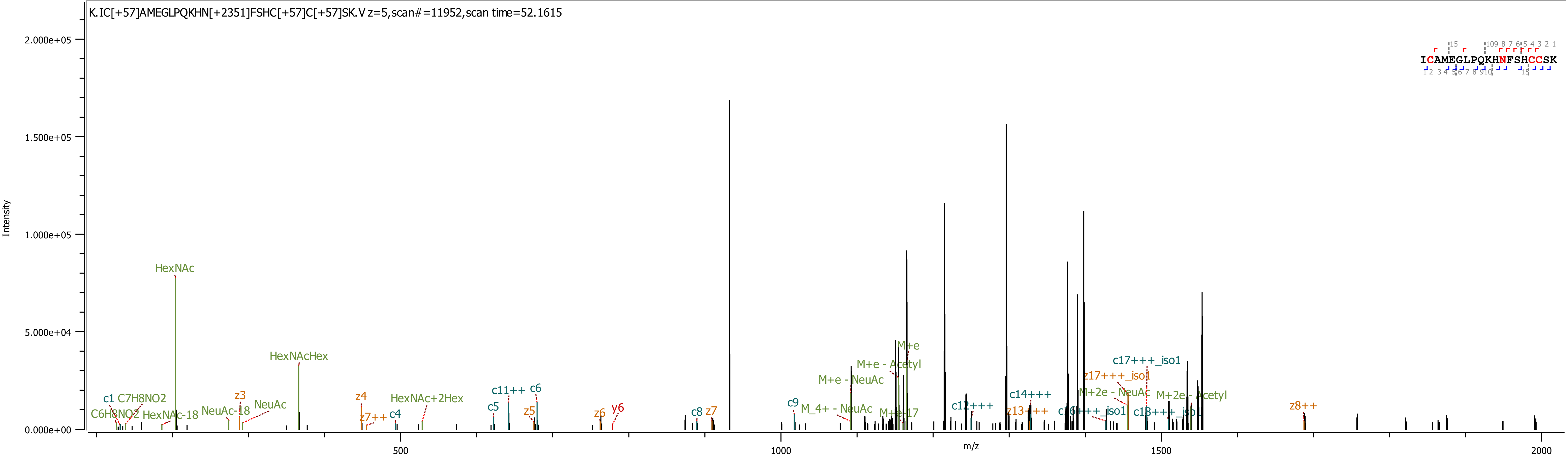

K.IC[+57]AMEGLPQKHN[+2351]FSHC[+57]C[+57]SK.V z=5,scan#=11952,scan time=52.1615
ICAMEGLPQKHNFSHCCSK
Intensity
m/z
2.000e+05
1.500e+05
1.000e+05
5.000e+04
0.000e+00
500
1000
1500
2000
HexNAc
HexNAcHex
HexNAc+2Hex
NeuAc
NeuAc-18
HexNAc-18
C6H8NO2
c1
C7H8NO2
z3
z4
z7++
c4
c5
c11++
c6
z5
z6
y6
c8
z7
c9
M_4++ - NeuAc
M+e - NeuAc
M+e - Acetyl
M+e
M+e-17
c12+++
c14+++
z13+++
c16+++_iso1
c17+++_iso1
z17+++_iso1
M+2e - NeuAc
M+2e - Acetyl
c18+++_iso1
z8++

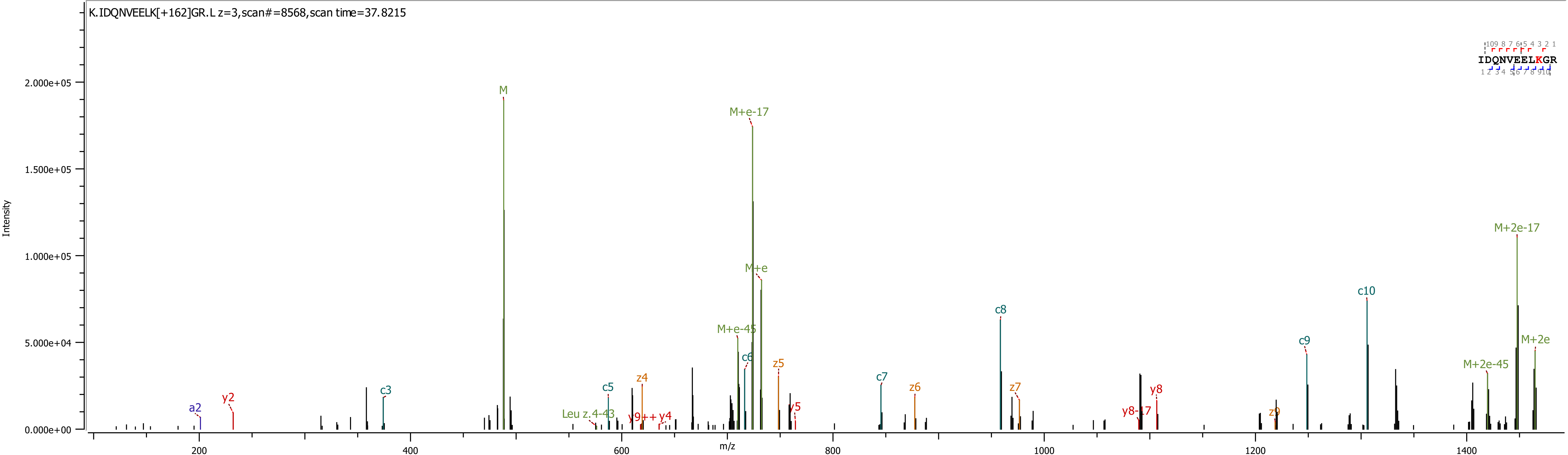

K.IDQNVEELK[+162]GR.L z=3,scan#=8568,scan time=37.8215
Intensity
m/z
0.000e+00
5.000e+04
1.000e+05
1.500e+05
2.000e+05
200
400
600
800
1000
1200
1400
IDQNVEELKGR
M
M+e-17
M+e
M+e-45
M+2e-17
M+2e
M+2e-45
a2
y2
c3
Leu z.4-43
c5
z4
y9++
y4
c6
z5
y5
c7
z6
c8
z7
y8-17
y8
z9
c9
c10

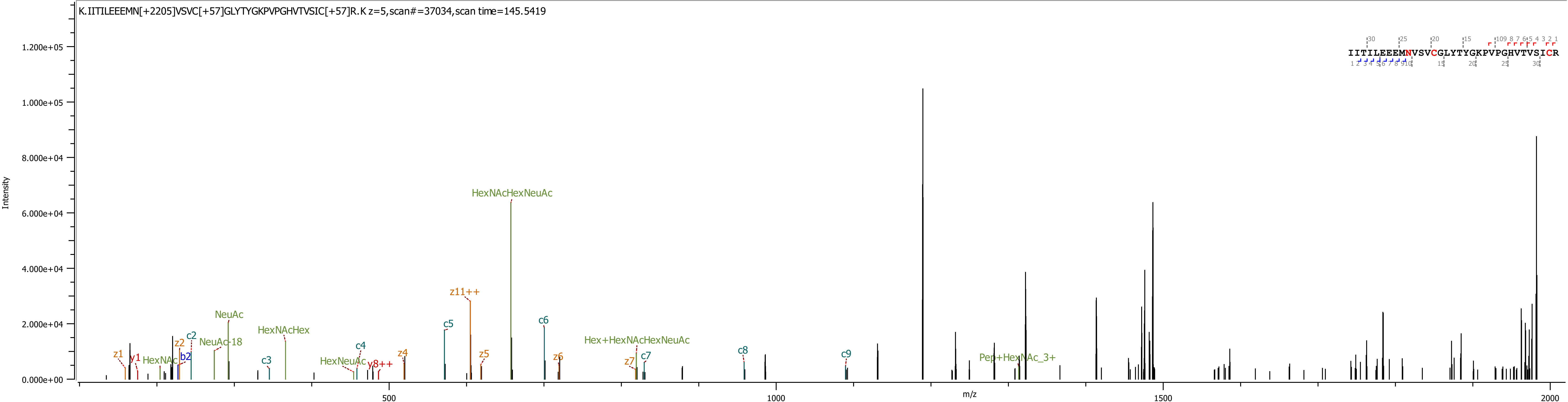

K.IITILEEEMN[+2205]VSVC[+57]GLYTYGKPVPGHVTVSIC[+57]R.K z=5,scan#=37034,scan time=145.5419
IITILEEEMNVSVCGLYTYGKPVPGHVTVSICR
Intensity
m/z
HexNAcHexNeuAc
z11++
c5
c6
NeuAc
HexNAcHex
NeuAc-18
c2
z2
b2
z1
y1
HexNAc
c3
c4
HexNeuAc
y8++
z4
z5
z6
Hex+HexNAcHexNeuAc
z7
c7
c8
c9
Pep+HexNAc_3+

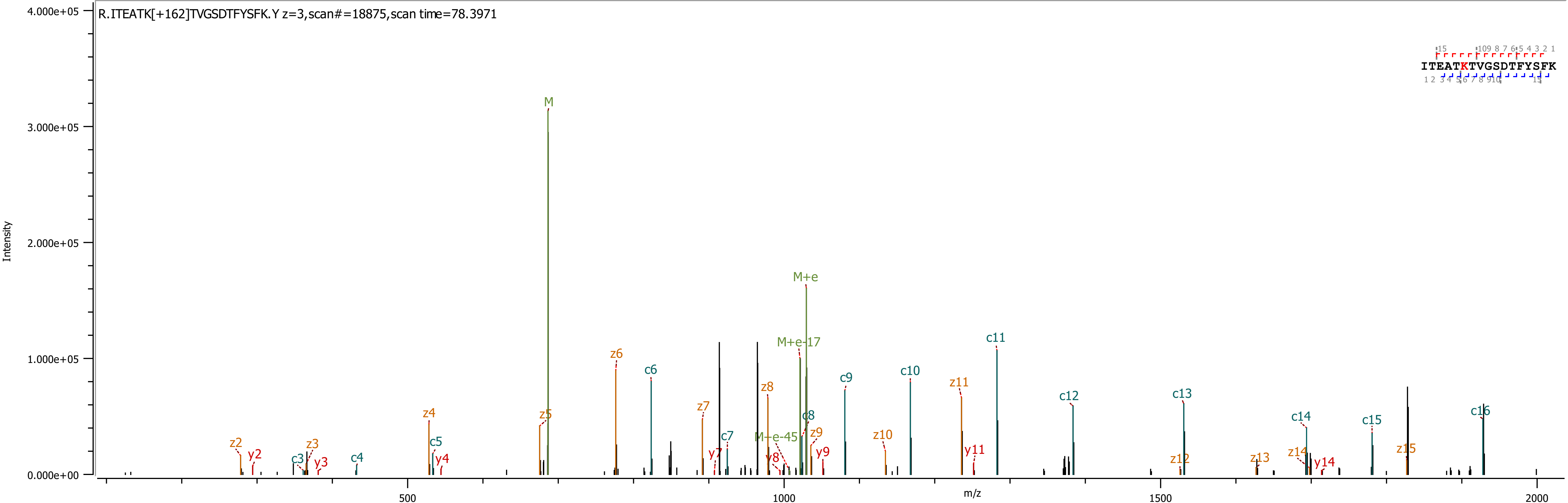

R.ITEATK[+162]TVGSDTFYSFK.Y z=3,scan#=18875,scan time=78.3971
ITEATKTVGSDTFYSFK
Intensity
m/z
4.000e+05
3.000e+05
2.000e+05
1.000e+05
0.000e+00
500
1000
1500
2000
M
M+e
M+e-17
M+e-45
z2 y2 c3 z3 y3 c4 z4 c5 y4 z5 z6 c6 z7 y7 c7 z8 y8 c8 z9 y9 c9 z10 c10 z11 y11 c11 c12 z12 c13 z13 z14 c14 y14 c15 z15 c16

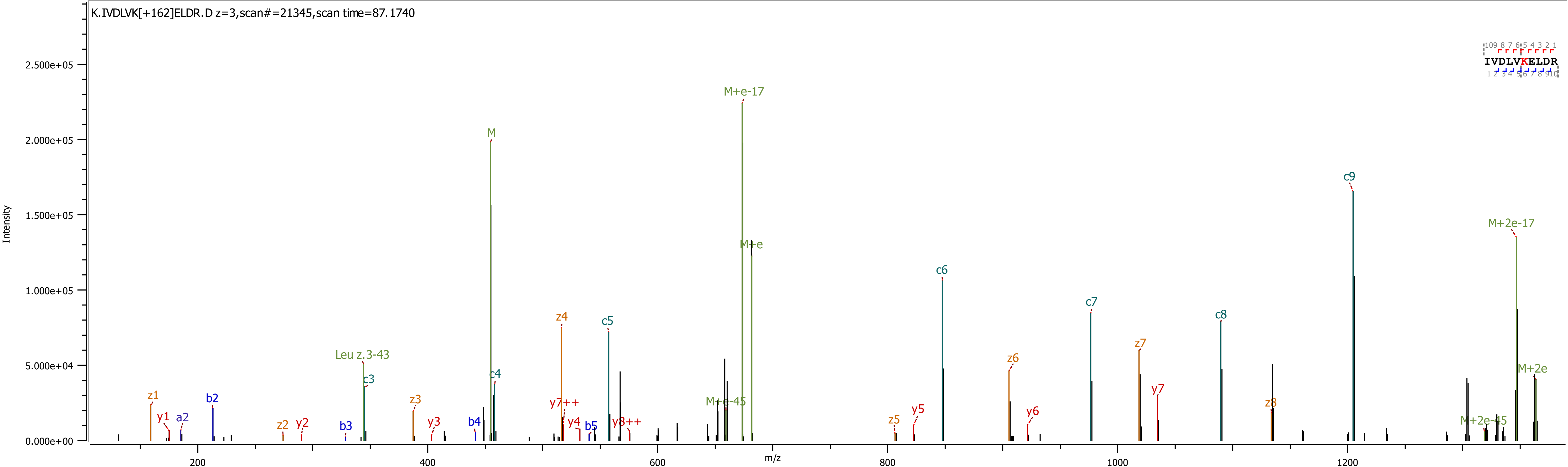
K.IVDLVK[+162]ELDR.D z=3,scan#=21345,scan time=87.1740
Intensity
m/z
IVDLVKELDR
M+e-17
M
c9
M+2e-17
M+e
c6
c7
c8
z4
c5
z7
z6
Leu z.3-43
c4
c3
M+2e
y7
z1
b2
z3
y7++
M+e-45
z8
y5
y6
y1
a2
z2
y2
b3
y3
b4
y4
b5
y3++
z5
M+2e-45

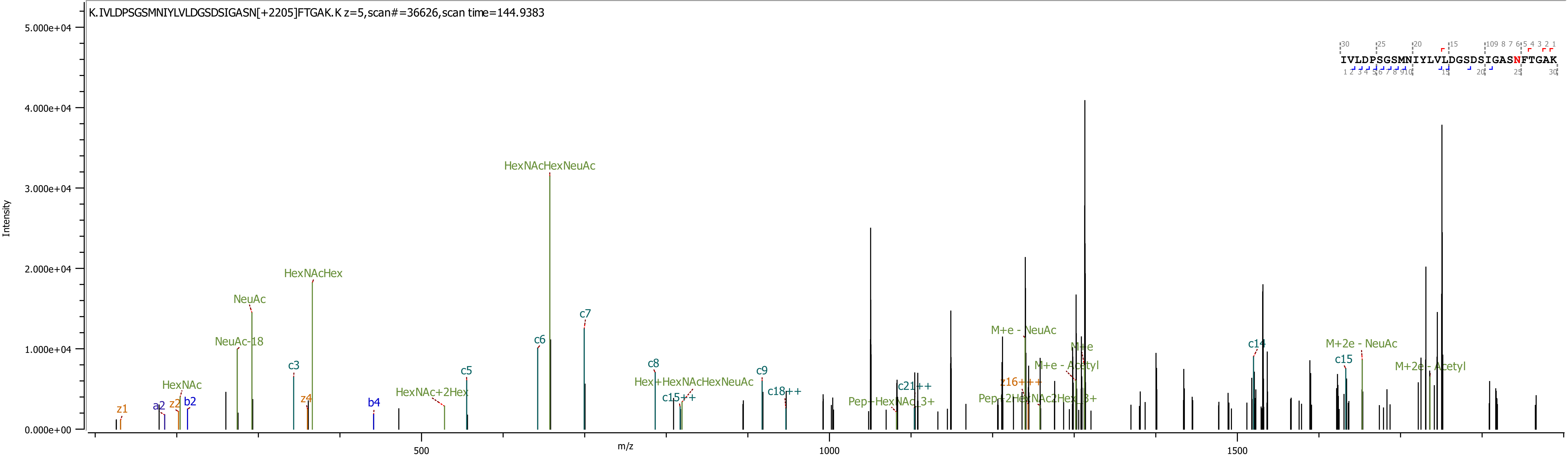
K.IVLDPSGSMNIYLVLDGSDSIGASN[+2205]FTGAK.K z=5,scan#=36626,scan time=144.9383
Intensity
m/z
IVLDPSGSMNIYLVLDGSDSIGASNFTGAK
HexNAcHexNeuAc
HexNAcHex
NeuAc
NeuAc-18
HexNAc
HexNAc+2Hex
Hex+HexNAcHexNeuAc
Pep+HexNAc_3+
Pep+2HexNAc2Hex_3+
M+e - NeuAc
M+e
M+e - Acetyl
M+2e - NeuAc
M+2e - Acetyl
z1
a2
z2
b2
c3
z4
b4
c5
c6
c7
c8
c15++
c9
c18++
c21++
z16+++
c14
c15
5.000e+04
4.000e+04
3.000e+04
2.000e+04
1.000e+04
0.000e+00
500
1000
1500

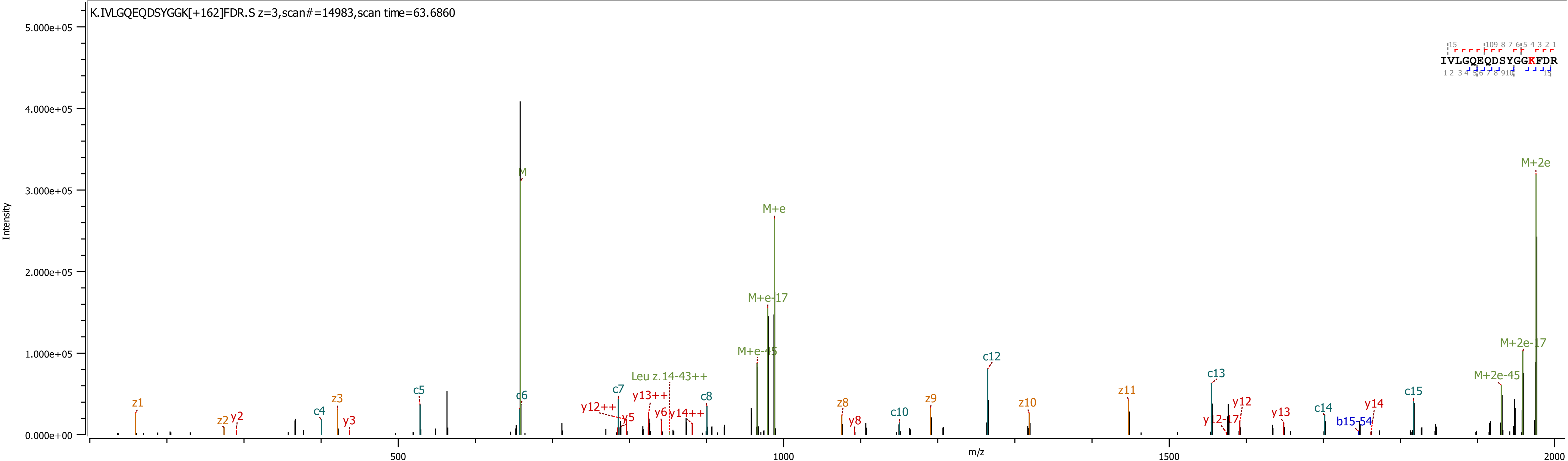
K.IVLGQEQDSYGGK[+162]FDR.S z=3,scan#=14983,scan time=63.6860
IVLGQEQDSYGGKFDR
Intensity
m/z
5.000e+05
4.000e+05
3.000e+05
2.000e+05
1.000e+05
0.000e+00
500
1000
1500
2000
M
M+e
M+e-17
M+e-45
M+2e
M+2e-17
M+2e-45
Leu z.14-43++
z1
z2
z3
z8
z9
z10
z11
y2
y3
y5
y6
y8
y12
y12-17
y13
y14
y12++
y13++
y14++
c4
c5
c6
c7
c8
c10
c12
c13
c14
c15
b15-54

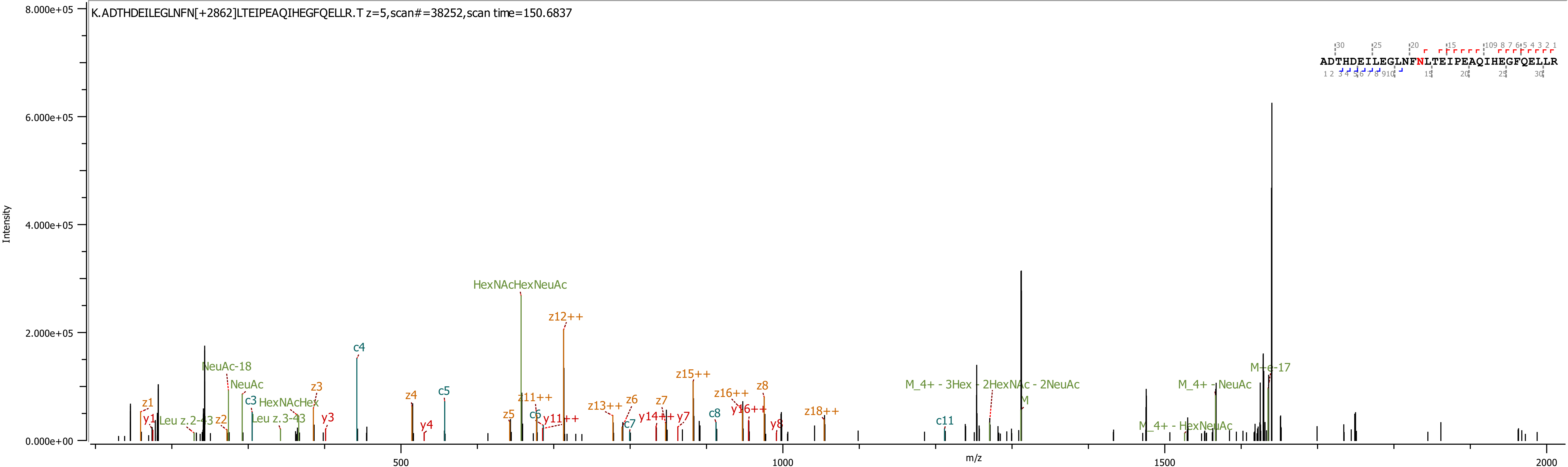

K.ADTHDEILEGLNFN[+2862]LTEIPEAQIHEGFQELLR.T z=5,scan#=38252,scan time=150.6837
ADTHDEILEGLNFNLTEIPEAQIHEGFQELLR
Intensity
m/z
8.000e+05
6.000e+05
4.000e+05
2.000e+05
0.000e+00
500
1000
1500
2000
HexNAcHexNeuAc
z12++
c4
NeuAc-18
NeuAc
c3
HexNAcHex
Leu z.2-43
Leu z.3-43
z1
y1
z2
z3
y3
z4
y4
c5
z5
c6
z11++
y11++
z13++
z6
c7
z7
y14++
y7
z15++
c8
z16++
y16++
z8
y8
z18++
c11
M_4+ - 3Hex - 2HexNAc - 2NeuAc
M
M_4+ - NeuAc
M_4+ - HexNeuAc
M+c-17

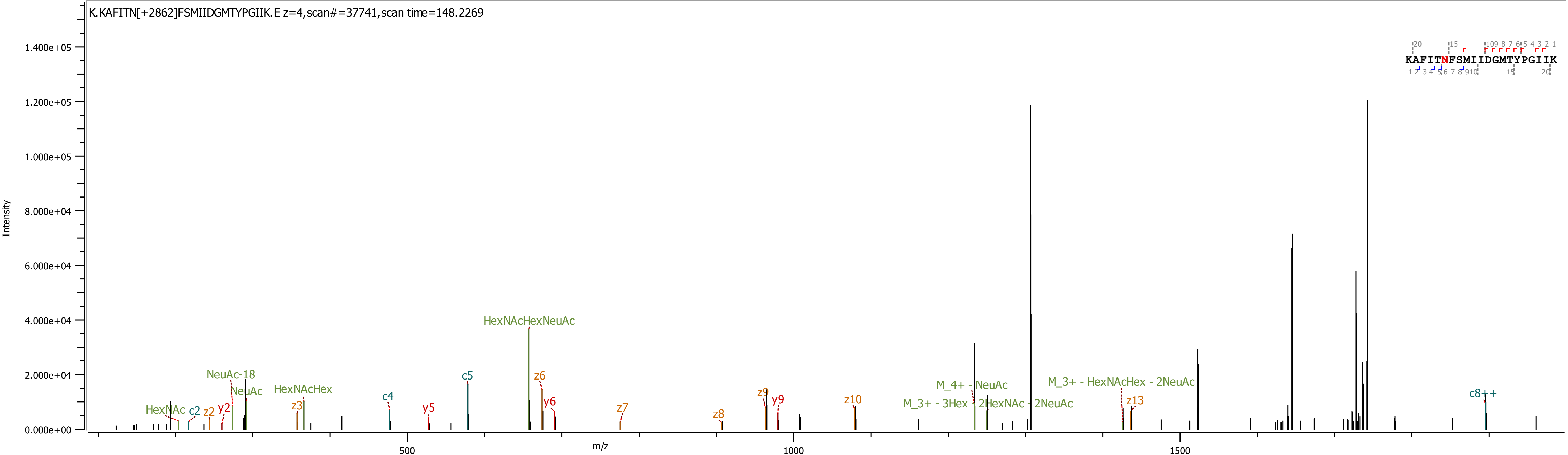
K.KAFITN[+2862]FSMIIDGMTYPGIIK.E z=4,scan#=37741,scan time=148.2269
KAFITNFSMIIDGMTYPGIIK
Intensity
m/z
1.400e+05
1.200e+05
1.000e+05
8.000e+04
6.000e+04
4.000e+04
2.000e+04
0.000e+00
500
1000
1500
HexNAc
c2
z2
y2
NeuAc-18
NeuAc
HexNAcHex
z3
c4
y5
c5
HexNAcHexNeuAc
z6
y6
z7
z8
z9
y9
z10
M_4+ - NeuAc
M_3+ - 3Hex - 2HexNAc - 2NeuAc
M_3+ - HexNAcHex - 2NeuAc
z13
c8++

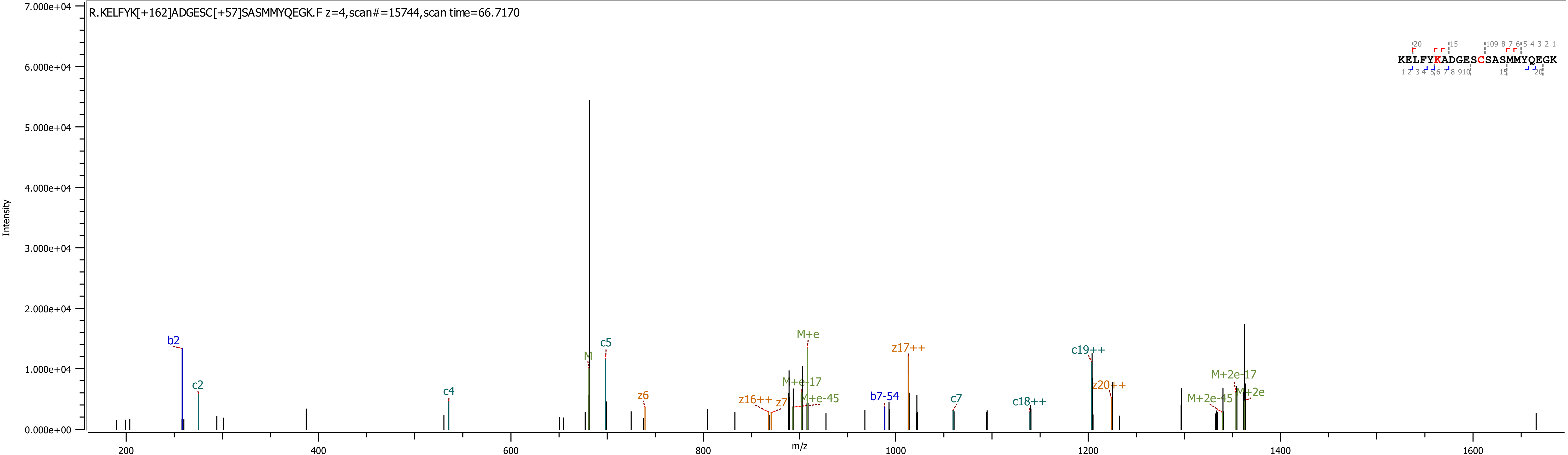
R.KELFYK[+162]ADGESC[+57]SASMMYQEGK.F z=4,scan#=15744,scan time=66.7170
KELFYKADGESCSASMMYQEGK
Intensity
m/z
b2
c2
c4
M
c5
z6
z16++
z7
M+e-17
M+e
M+e-45
b7-54
z17++
c7
c18++
c19++
z20++
M+2e-45
M+2e-17
M+2e

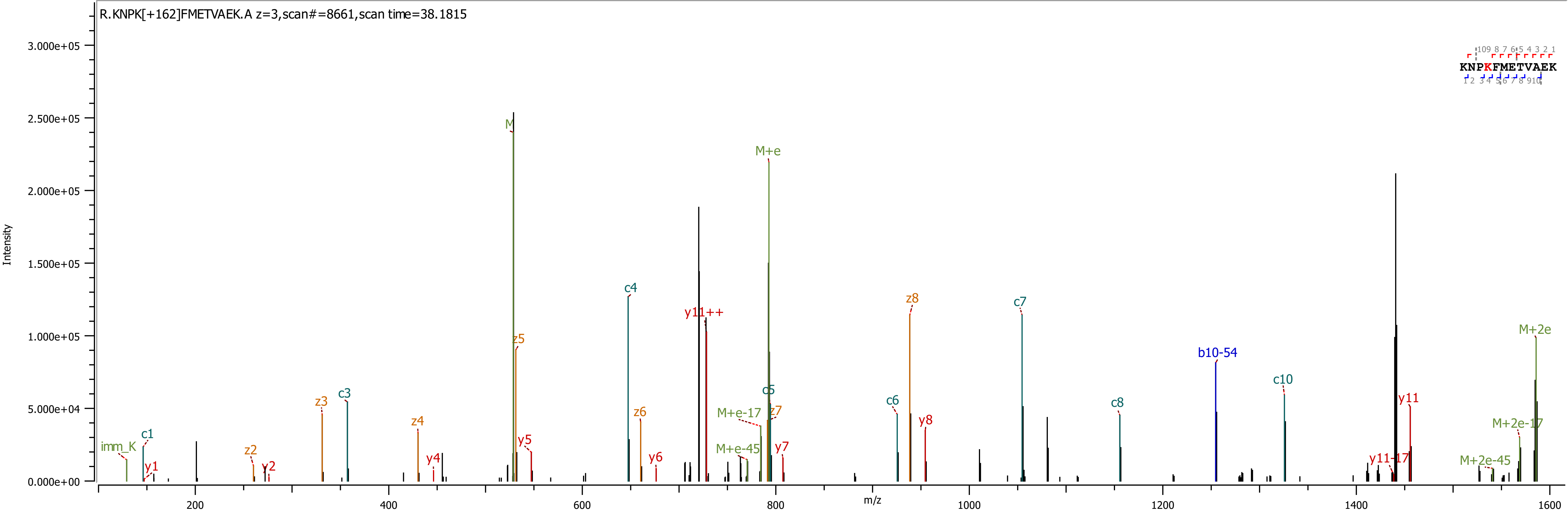

R.KNPK[+162]FMETVAEK.A z=3,scan#=8661,scan time=38.1815
KNPKFMETVAEK
Intensity
m/z
M
M+e
M+2e
y11++
z8
c7
c4
b10-54
c10
y11
M+2e-17
M+2e-45
y11-17
c8
c6
y8
c5
z7
M+e-17
M+e-45
y7
y6
z6
z5
y5
z4
y4
c3
z3
z2
y2
c1
y1
imm_K

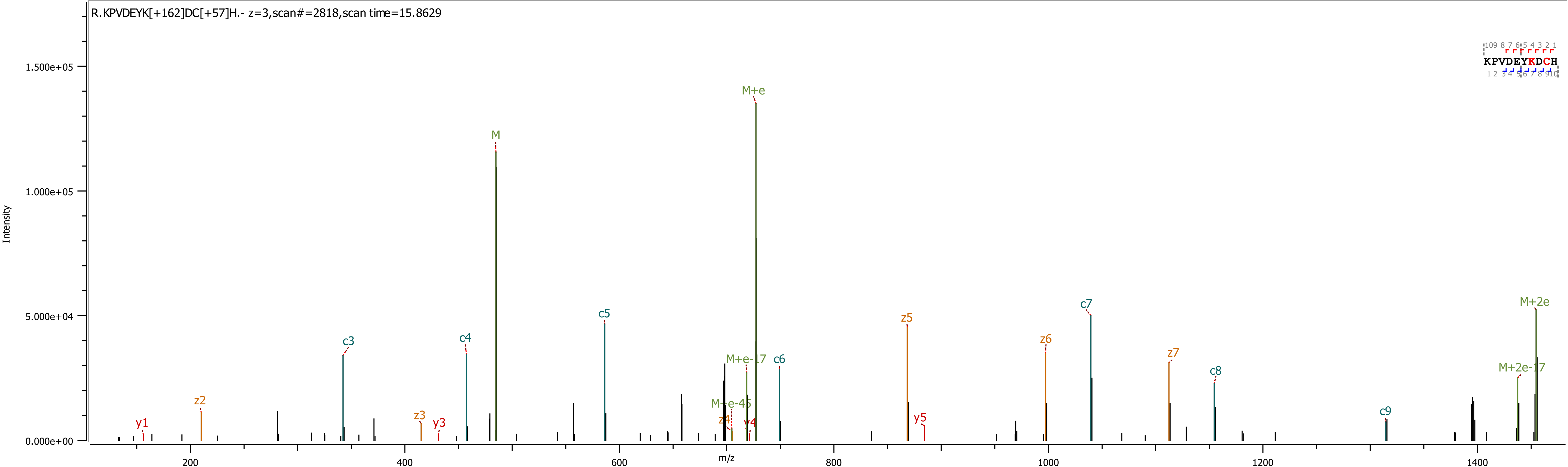
R.KPVDEYK[+162]DC[+57]H.- z=3,scan#=2818,scan time=15.8629
KPVDEYKDCH
Intensity
m/z
M
M+e
M+2e
M+e-17
M+e-45
M+2e-17
c3
c4
c5
c6
c7
c8
c9
z2
z3
z4
z5
z6
z7
y1
y3
y4
y5
0.000e+00
5.000e+04
1.000e+05
1.500e+05
200
400
600
800
1000
1200
1400

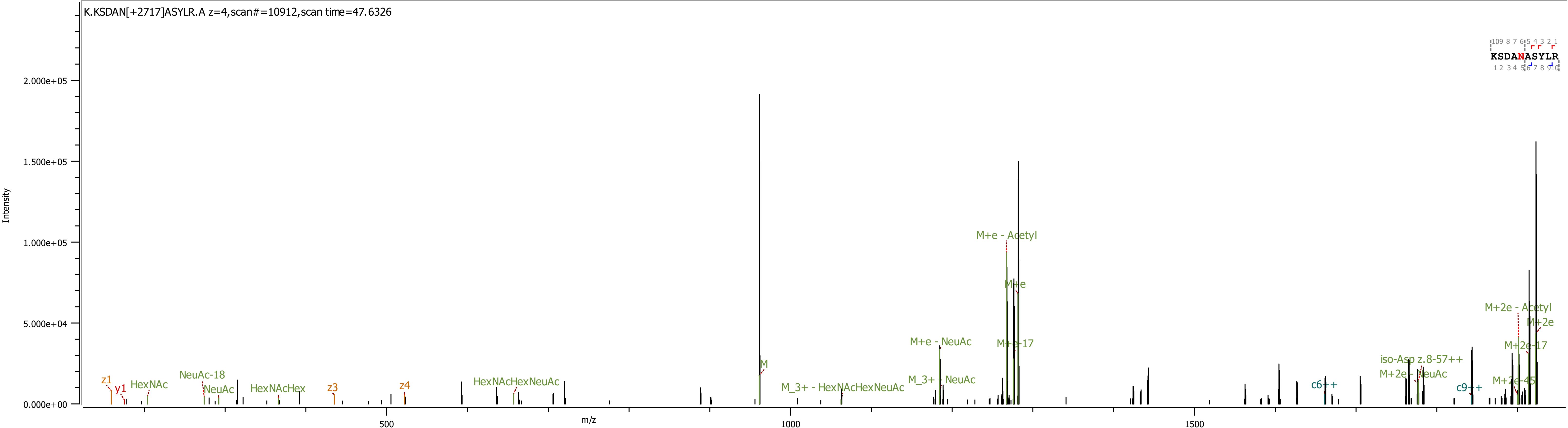
K.KSDAN[+2717]ASYLR.A z=4,scan#=10912,scan time=47.6326
Intensity
m/z
0.000e+00
5.000e+04
1.000e+05
1.500e+05
2.000e+05
500
1000
1500
KSDANASYLR
z1
y1
HexNAc
NeuAc-18
NeuAc
HexNAcHex
z3
z4
HexNAcHexNeuAc
M
M_3+ - HexNAcHexNeuAc
M+e - NeuAc
M_3+ - NeuAc
M+e - Acetyl
M+e
M+e-17
c6++
iso-Asp z.8-57++
M+2e - NeuAc
c9++
M+2e - Acetyl
M+2e
M+2e-17
M+2e-45

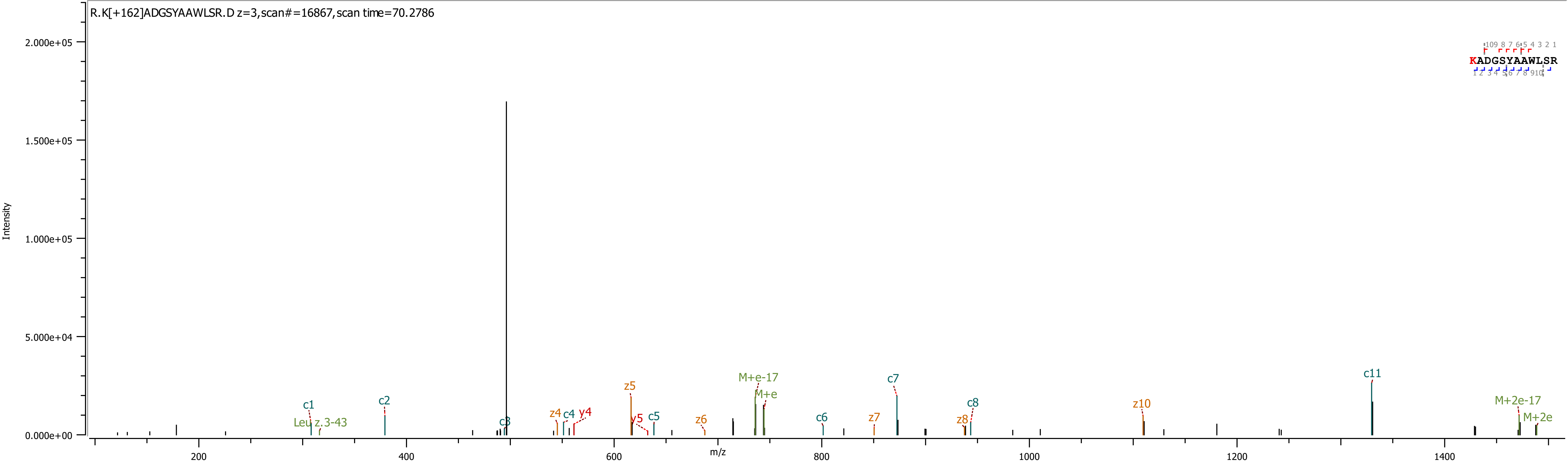

R.K[+162]ADGSYAAWLSR.D z=3,scan#=16867,scan time=70.2786
KADGSYAAWLSR
Intensity
m/z
c1
c2
c3
c4
c5
c6
c7
c8
c11
z4
z5
z6
z7
z8
z10
y4
y5
Leu z.3-43
M+e-17
M+e
M+2e-17
M+2e

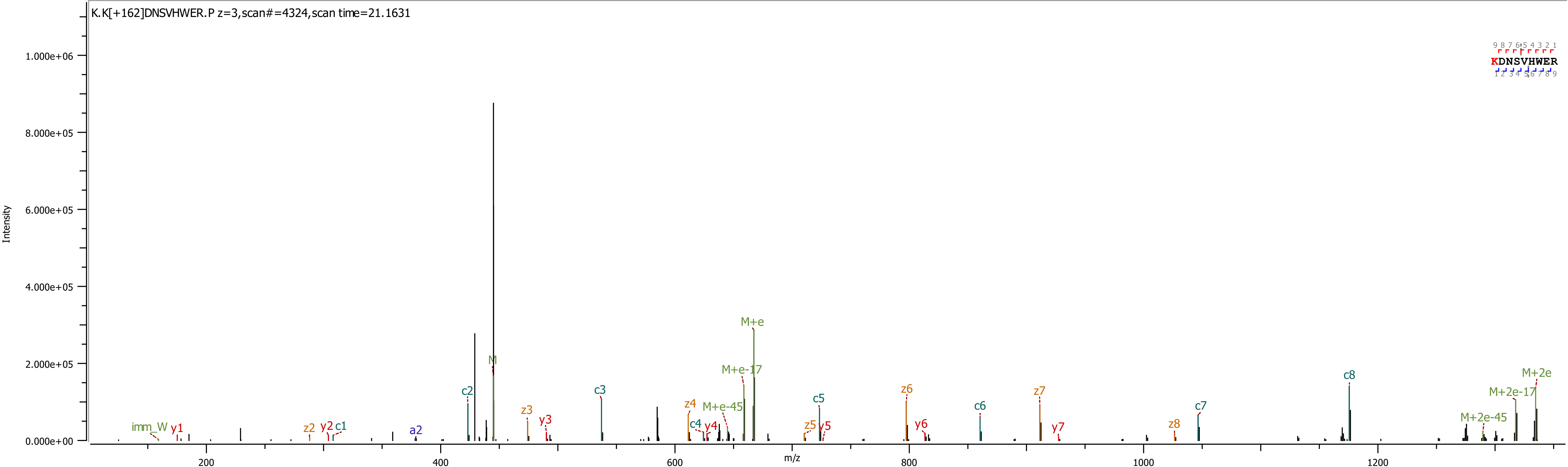
K.K[+162]DNSVHWER.P z=3,scan#=4324,scan time=21.1631
KDNSVHWER
Intensity
m/z
1.000e+06
8.000e+05
6.000e+05
4.000e+05
2.000e+05
0.000e+00
200
400
600
800
1000
1200
imm_W
y1
z2
y2
c1
a2
c2
M
z3
y3
c3
z4
c4
y4
M+e-45
M+e-17
M+e
z5
y5
c5
z6
y6
c6
z7
y7
z8
c7
c8
M+2e-45
M+2e-17
M+2e

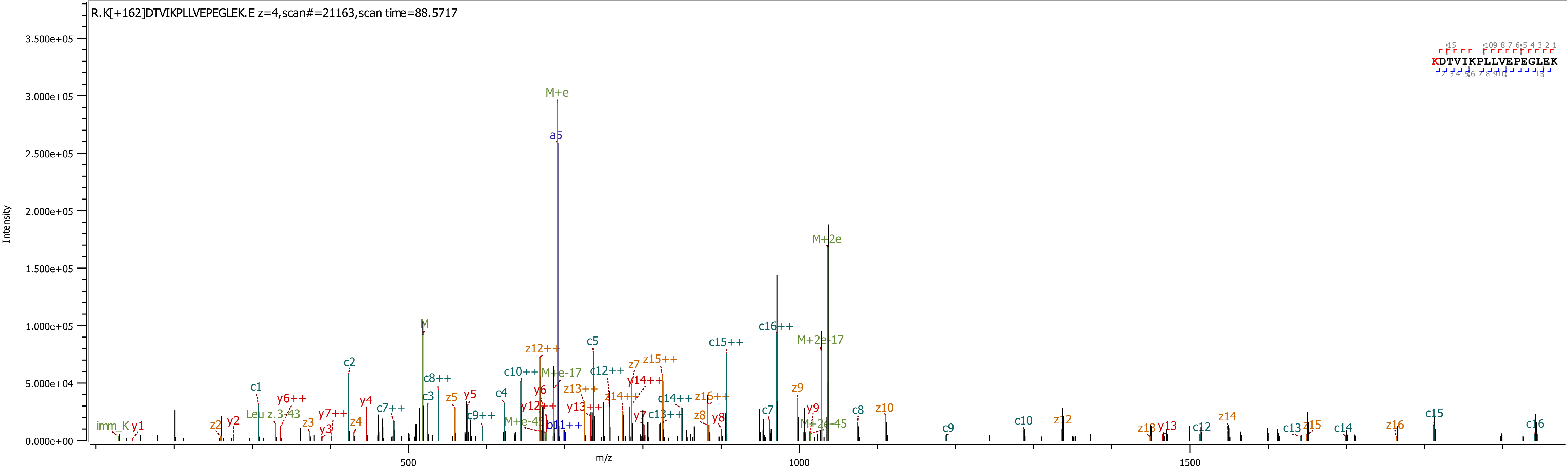

R.K[+162]DTVIKPLLVEPEGLEK.E z=4,scan#=21163,scan time=88.5717
KDTVIKPLLVEPEGLEK
Intensity
m/z
M+e
a5
M+2e
M
c16++
M+2e-17
c15++
c5
z12++
c2
c8++
c10++
M+e-17
c12++
z7
z15++
c1
y6++
y4
z9
z13++
y14++
c3
y5
c4
z5
c7++
z14++
c14++
z16++
y12++
y13++
c7
y9
c8
z10
Leu
z.3-43
imm_K
y1
z2
y2
y7++
z3
y3
z4
c9++
M+e-45
b11++
c13++
z8
y8
M+2e-45
c9
c10
z12
z13
y13
c12
z14
c13
z15
c14
z16
c15
c16
0.000e+00
5.000e+04
1.000e+05
1.500e+05
2.000e+05
2.500e+05
3.000e+05
3.500e+05
500
1000
1500

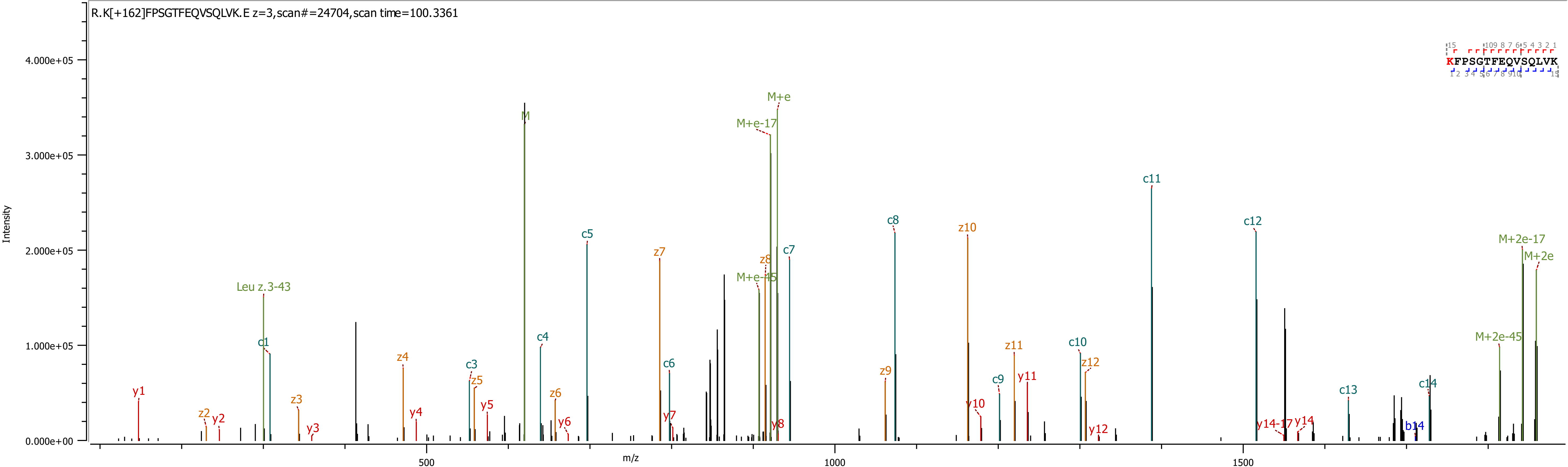

R.K[+162]FPSGTFEQVSQLVK.E z=3,scan#=24704,scan time=100.3361
KFPSGTFEQVSQLVK
Intensity
m/z
4.000e+05
3.000e+05
2.000e+05
1.000e+05
0.000e+00
500
1000
1500
M
M+e
M+e-17
M+e-45
Leu z.3-43
c1
c3
c4
c5
c6
c7
c8
c9
c10
c11
c12
c13
c14
z2
z3
z4
z5
z6
z7
z8
z9
z10
z11
z12
y1
y2
y3
y4
y5
y6
y7
y8
y10
y11
y12
y14-17
y14
b14
M+2e-17
M+2e
M+2e-45

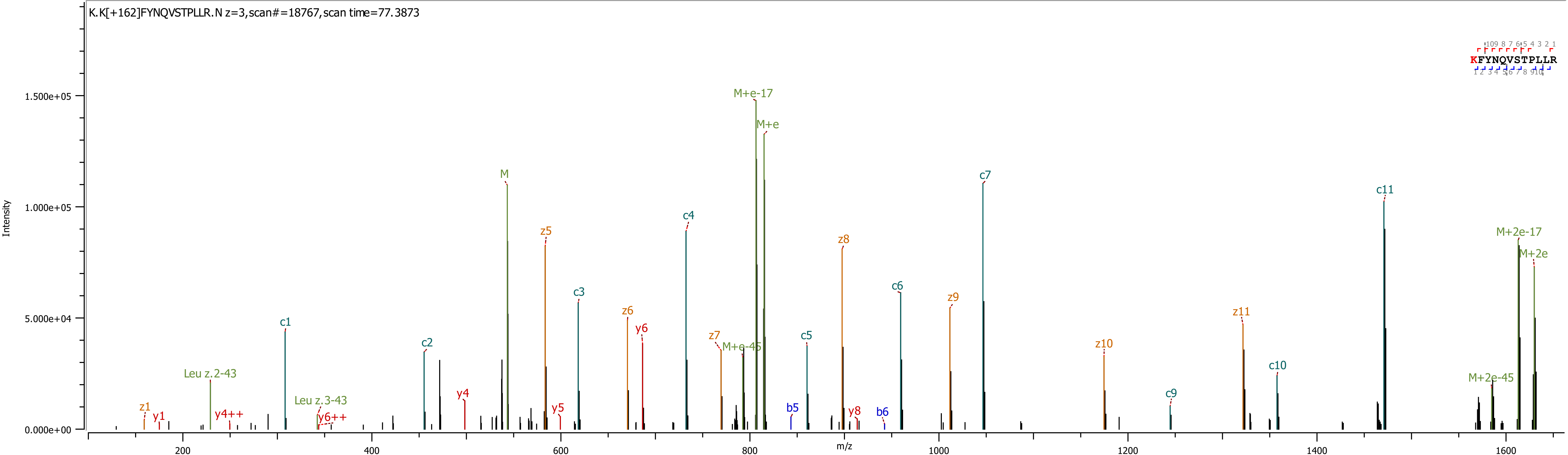

K.K[+162]FYNQVSTPLLR.N z=3,scan#=18767,scan time=77.3873
Intensity
m/z
KFYNQVSTPLLR
M+e-17
M+e
M
c7
c11
c4
z5
z8
M+2e-17
M+2e
c3
c1
c6
z9
z6
y6
z11
z7
M+e-45
c5
z10
c2
c10
Leu z.2-43
M+2e-45
y4
c9
Leu z.3-43
z1
y1
y4++
y6++
y5
b5
y8
b6
1.500e+05
1.000e+05
5.000e+04
0.000e+00
200
400
600
800
1000
1200
1400
1600

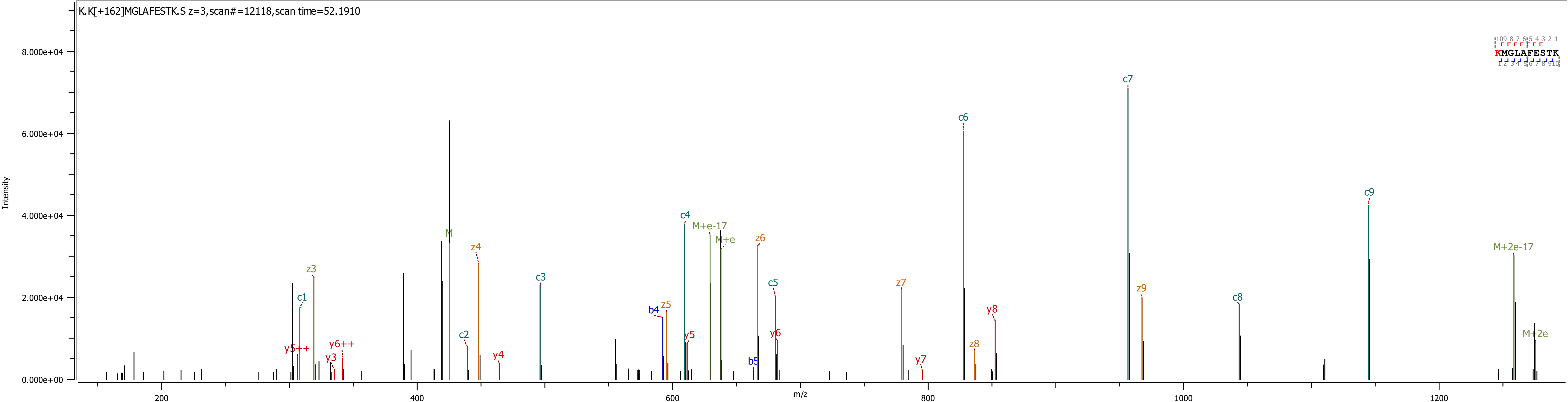
K.K[+162]MGLAFESTK.S z=3,scan#=12118,scan time=52.1910
KMGLAFESTK
Intensity
m/z
8.000e+04
6.000e+04
4.000e+04
2.000e+04
0.000e+00
200
400
600
800
1000
1200
c1
c2
c3
c4
c5
c6
c7
c8
c9
z3
z4
z5
z6
z7
z8
z9
y3
y4
y5
y6
y7
y8
y5++
y6++
b4
b5
M
M+e-17
M+e
M+2e-17
M+2e

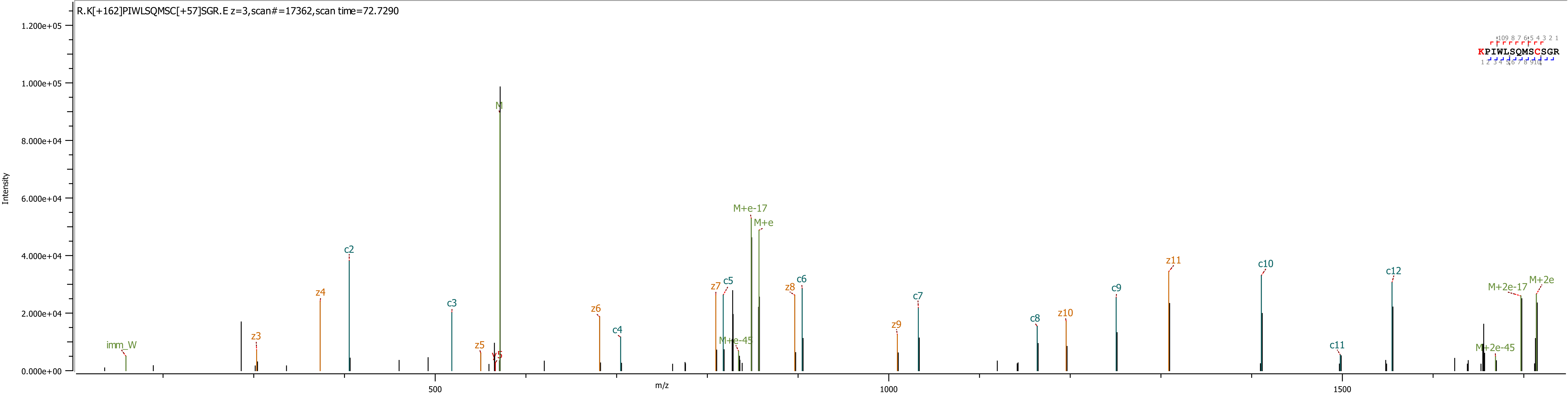

R.K[+162]PIWLSQMSC[+57]SGR.E z=3,scan#=17362,scan time=72.7290
Intensity
m/z
KPIWLSQMSCSGR
imm_W
z3
z4
c2
c3
z5
y5
M
z6
c4
z7
c5
M+e-45
M+e-17
M+e
z8
c6
z9
c7
c8
z10
c9
z11
c10
c11
c12
M+2e-45
M+2e-17
M+2e

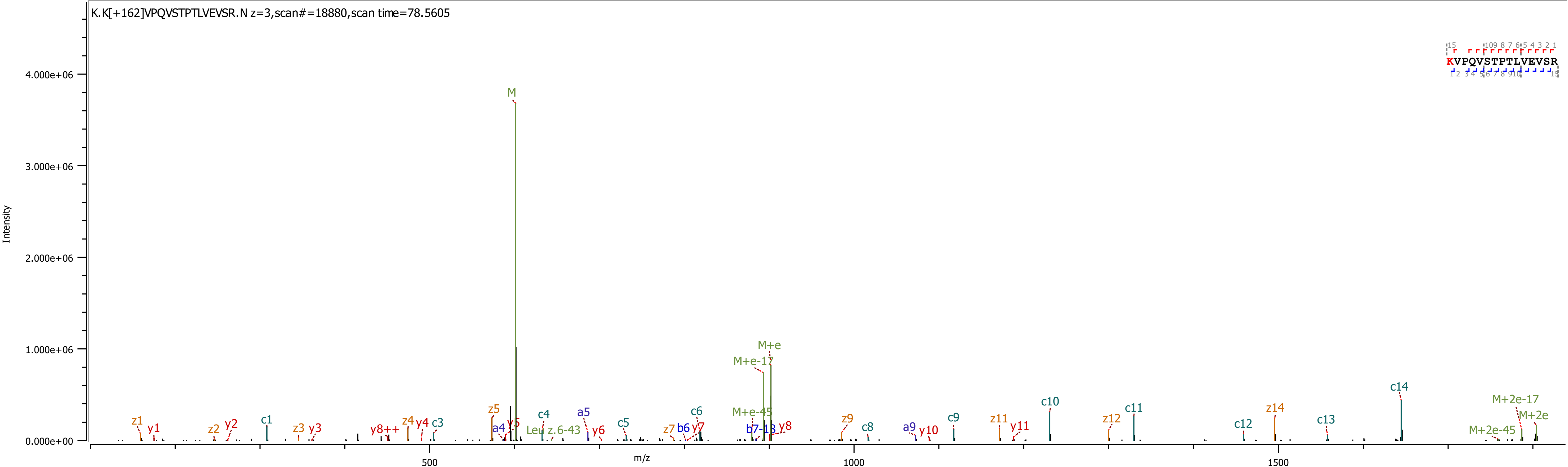

K.K[+162]VPQVSTPTLVEVSR.N z=3,scan#=18880,scan time=78.5605
KVPQVSTPTLVEVSR
Intensity
m/z
4.000e+06
3.000e+06
2.000e+06
1.000e+06
0.000e+00
500
1000
1500
M
M+e
M+e-17
M+e-45
M+2e-17
M+2e
M+2e-45
z1
y1
z2
y2
c1
z3
y3
y8++
z4
y4
c3
z5
a4
y5
c4
Leu z.6-43
a5
y6
c5
z7
b6
y7
c6
b7-18
y8
z9
c8
a9
y10
c9
z11
y11
c10
z12
c11
c12
z14
c13
c14

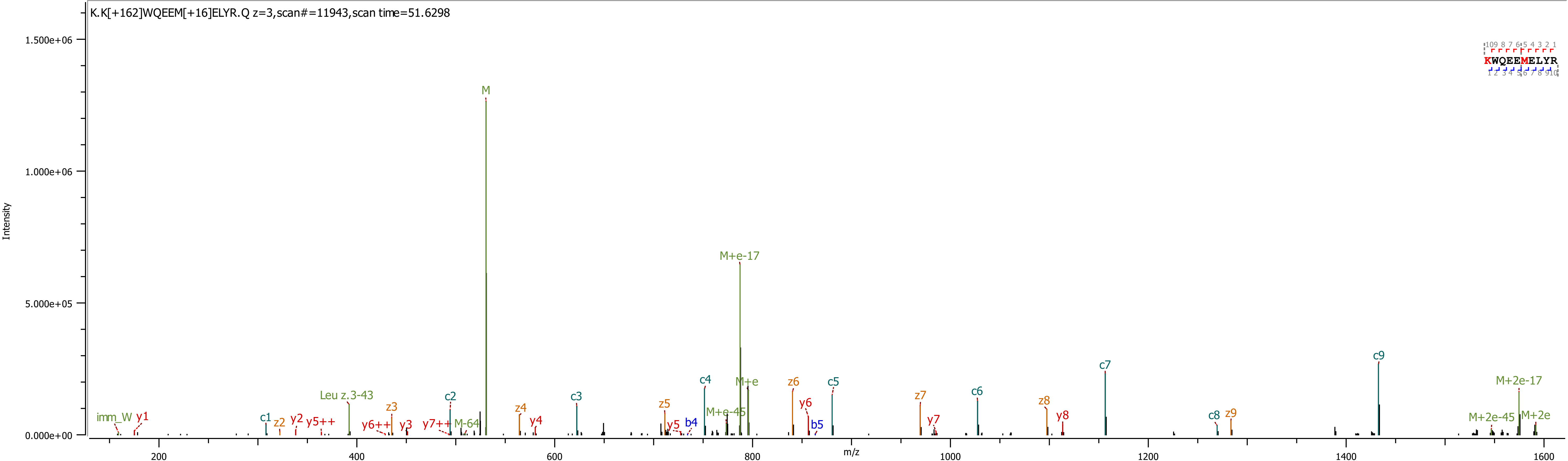
K.K[+162]WQEEM[+16]ELYR.Q z=3,scan#=11943,scan time=51.6298
Intensity
1.500e+06
1.000e+06
5.000e+05
0.000e+00
m/z
200
400
600
800
1000
1200
1400
1600
KWQEEMELYR
M
M+e-17
M+e
M+e-45
M-64
Leu z.3-43
imm_W
y1
c1
z2
y2
y5++
y6++
z3
y3
y7++
c2
z4
y4
c3
z5
y5
b4
c4
z6
c5
y6
b5
z7
y7
c6
z8
y8
c7
c8
z9
c9
M+2e-17
M+2e-45
M+2e

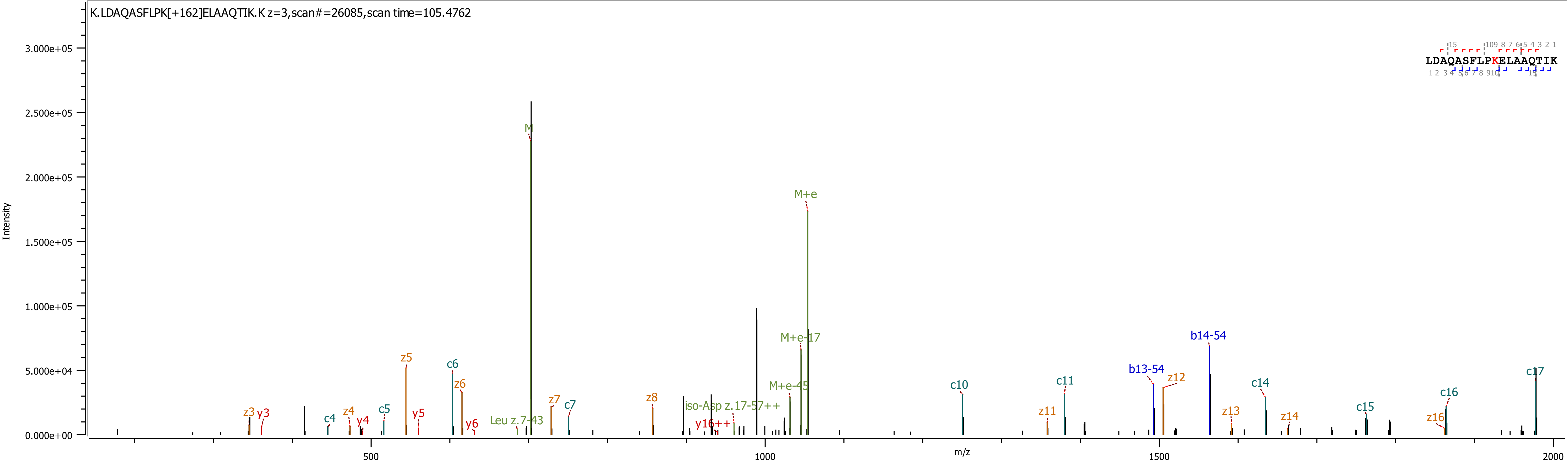
K.LDAQASFLPK[+162]ELAAQTIK.K z=3,scan#=26085,scan time=105.4762
Intensity
m/z
LDAQASFLPKELAAQTIK
M
M+e
M+e-17
M+e-45
z3
y3
c4
z4
y4
c5
z5
y5
c6
z6
y6
Leu z.7-43
z7
c7
z8
iso-Asp z.17-57++
y16++
c10
z11
c11
b13-54
z12
b14-54
z13
c14
z14
c15
c16
z16
c17

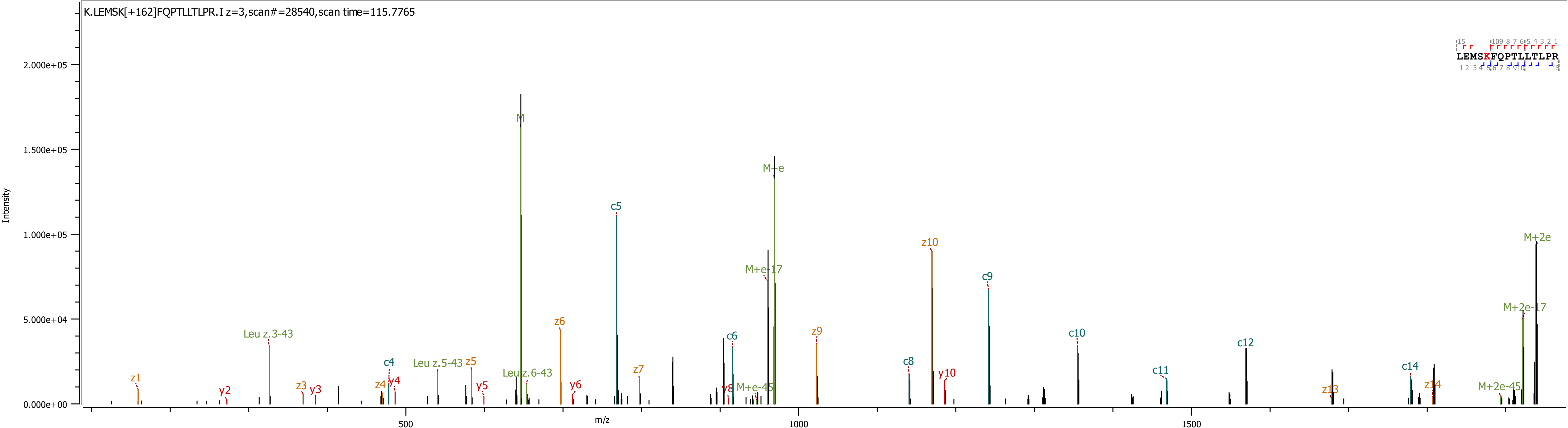
K.LEMSK[+162]FQPTLLTLPR.I z=3,scan#=28540,scan time=115.7765
Intensity
m/z
LEMSKFQPTLLTLPR
M
M+e
c5
M+e-17
z10
c9
M+2e
M+2e-17
z6
Leu z.3-43
z9
c6
c10
c12
c8
y10
c11
c14
z7
Leu z.6-43
Leu z.5-43
z5
c4
z1
y2
z3
y3
z4
y4
y5
y6
M+e-45
z13
z14
M+2e-45
0.000e+00
5.000e+04
1.000e+05
1.500e+05
2.000e+05
500
1000
1500

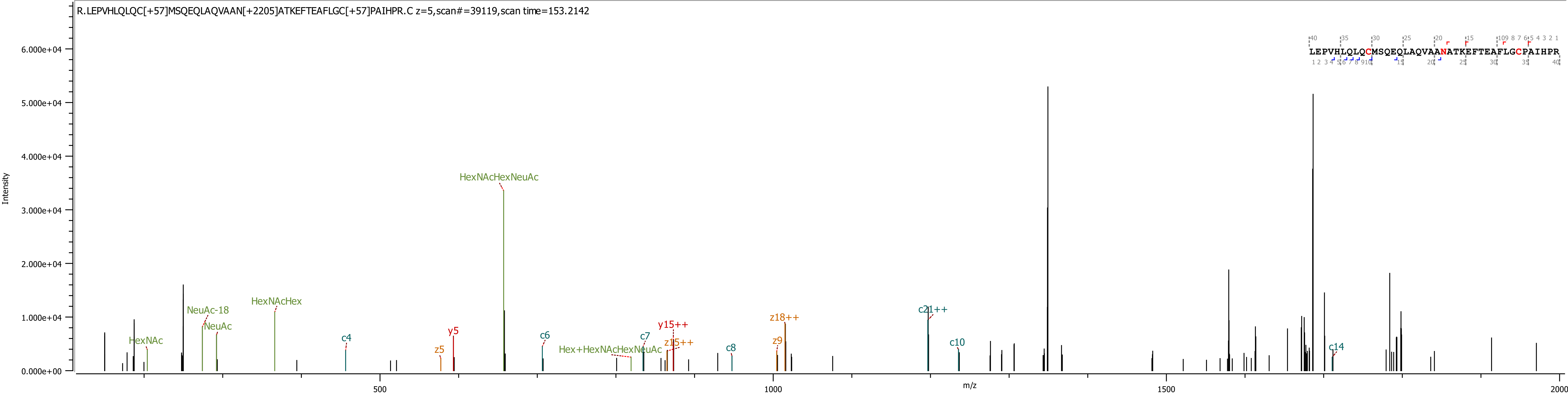

R.LEPVHLQLQC[+57]MSQEQLAQVAAN[+2205]ATKEFTEAFLGC[+57]PAIHPR.C z=5,scan#=39119,scan time=153.2142
LEPVHLQLQCMSQEQLAQVAANATKEFTEAFLGCPAIHPR
Intensity
m/z
HexNAc
NeuAc-18
NeuAc
HexNAcHex
HexNAcHexNeuAc
c4
z5
y5
c6
Hex+HexNAcHexNeuAc
c7
y15++
z15++
c8
z9
z18++
c21++
c10
c14

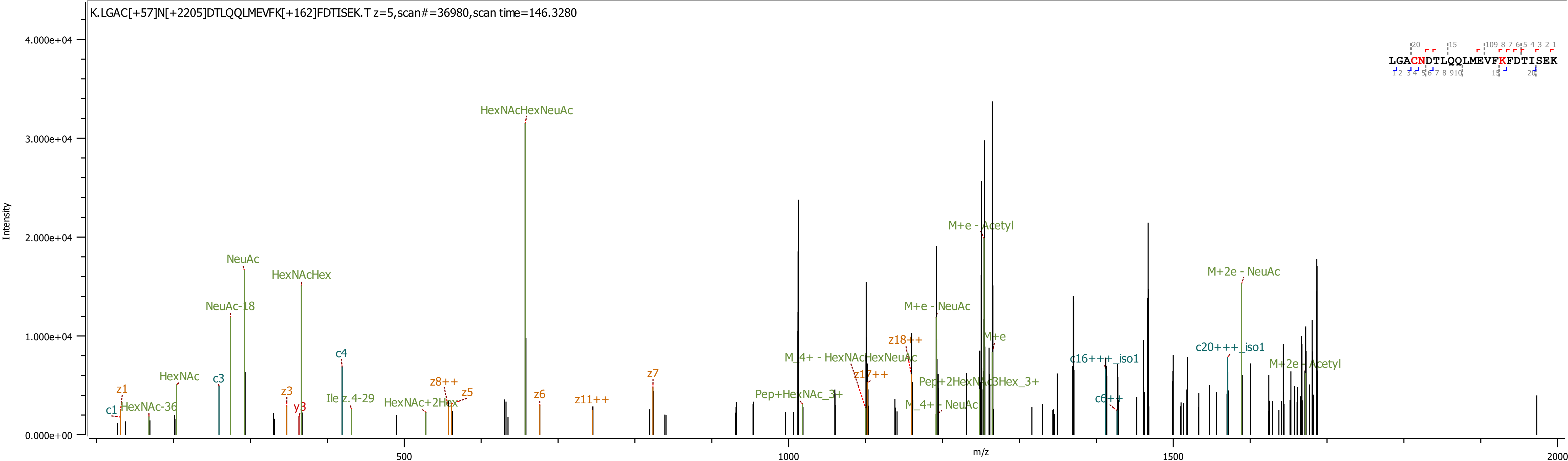
K.LGAC[+57]N[+2205]DTLQQLMEVFK[+162]FDTISEK.T z=5,scan#=36980,scan time=146.3280
Intensity
m/z
LGACNDTLQQLMEVFKFDTISEK
HexNAcHexNeuAc
NeuAc
HexNAcHex
NeuAc-18
HexNAc
HexNAc-36
c1
z1
c3
z3
y3
c4
Ile z.4-29
HexNAc+2Hex
z8++
z5
z6
z11++
z7
Pep+HexNAc_3+
M_4+ - HexNAcHexNeuAc
z17++
z18++
M+e - NeuAc
M+e - Acetyl
M+e
Pep+2HexNAc3Hex_3+
M_4+ - NeuAc
c16+++_iso1
c6++
c20+++_iso1
M+2e - NeuAc
M+2e - Acetyl

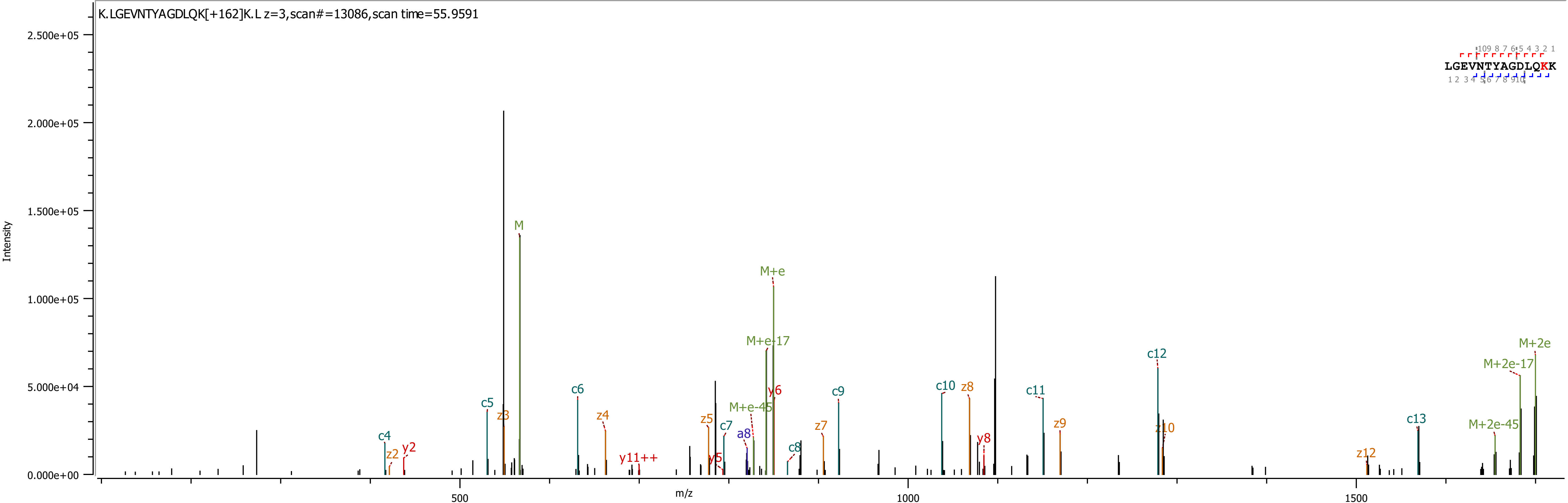

K.LGEVNTYAGDLQK[+162]K.L z=3,scan#=13086,scan time=55.9591
Intensity
m/z
LGEVNTYAGDLQKK
M
M+e
M+e-17
M+e-45
M+2e
M+2e-17
M+2e-45
c4
c5
c6
c7
c8
c9
c10
c11
c12
c13
z2
z3
z4
z5
z7
z8
z9
z10
z12
y2
y5
y6
y8
y11++
a8

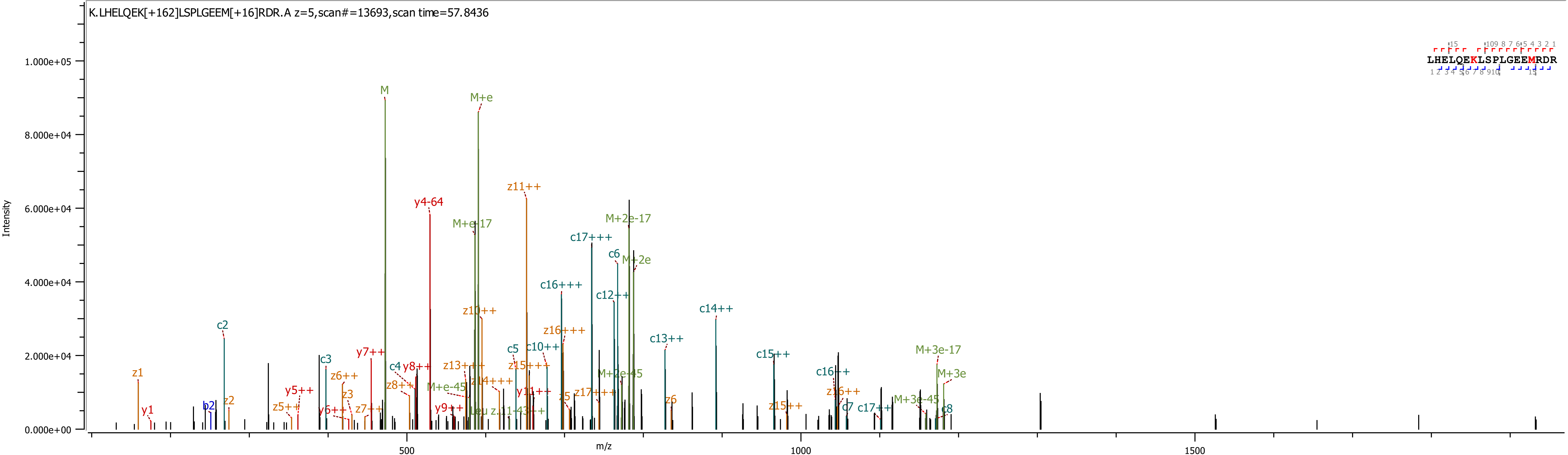
K.LHELQEK[+162]LSPLGEEM[+16]RDR.A z=5,scan#=13693,scan time=57.8436
LHELQEKLSPLGEEMRDR
Intensity
m/z
1.000e+05
8.000e+04
6.000e+04
4.000e+04
2.000e+04
0.000e+00
500
1000
1500
M
M+e
y4-64
z11++
M+e-17
M+2e-17
c17+++
c6
M+2e
c16+++
c12++
c14++
z10++
c2
z16+++
c13++
M+3e-17
c15++
M+3e
c16++
z16++

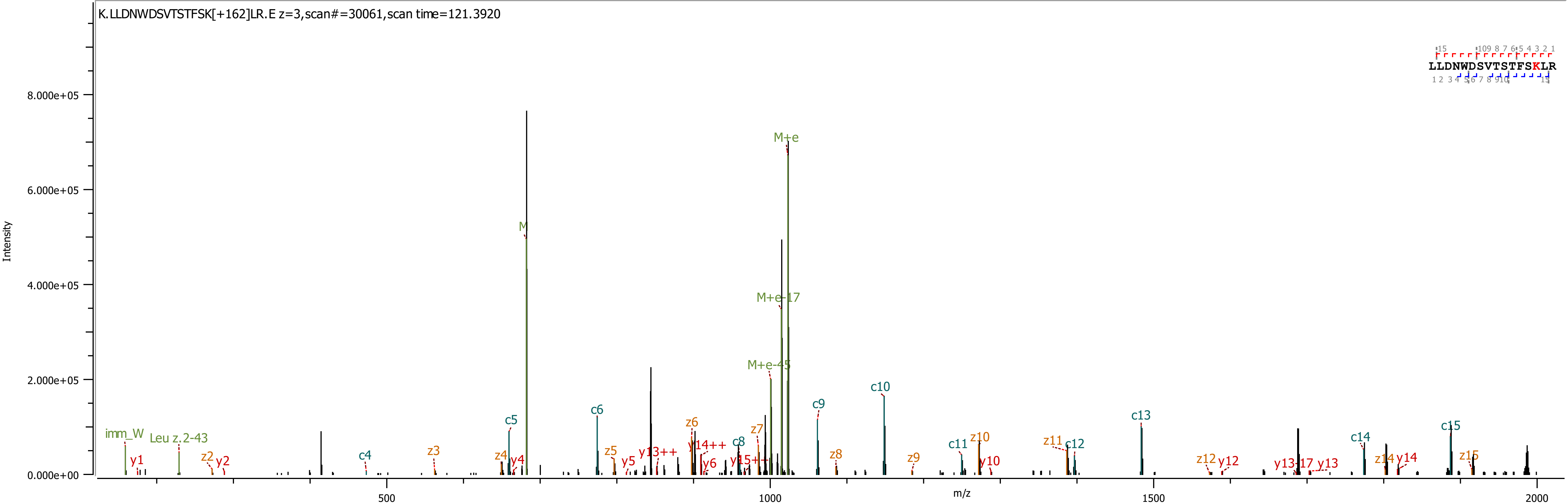
K.LLDNWDSVTSTFSK[+162]LR.E z=3,scan#=30061,scan time=121.3920
LLDNWDSVTSTFSKLR
Intensity
m/z
M+e
M
M+e-17
M+e-45
imm_W
Leu z.2-43
c4
c5
c6
c8
c9
c10
c11
c12
c13
c14
c15
z2
z3
z4
z5
z6
z7
z8
z9
z10
z11
z12
z14
z15
y1
y2
y4
y5
y6
y10
y12
y13
y14
y13++
y14++
y15++
y13-17

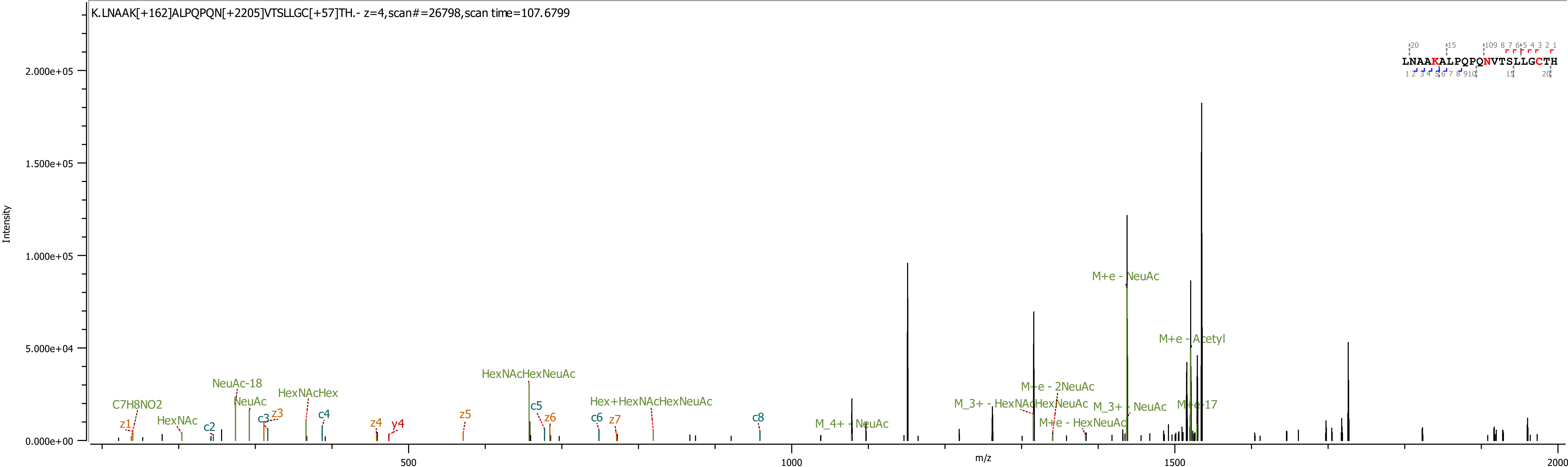

K.LNAAK[+162]ALPQPQN[+2205]VTSLLGC[+57]TH.- z=4,scan#=26798,scan time=107.6799
LNAAKALPQPQNVTSLLGCTH
Intensity
2.000e+05
1.500e+05
1.000e+05
5.000e+04
0.000e+00
m/z
500
1000
1500
2000
C7H8NO2
z1
HexNAc
c2
NeuAc-18
NeuAc
c3
z3
HexNAcHex
c4
z4
y4
z5
HexNAcHexNeuAc
c5
z6
c6
z7
Hex+HexNAcHexNeuAc
c8
M_4+ - NeuAc
M_3+ - HexNAcHexNeuAc
M+e - 2NeuAc
M+e - HexNeuAc
M_3+ - NeuAc
M+e - NeuAc
M+e - Acetyl
M+e-17

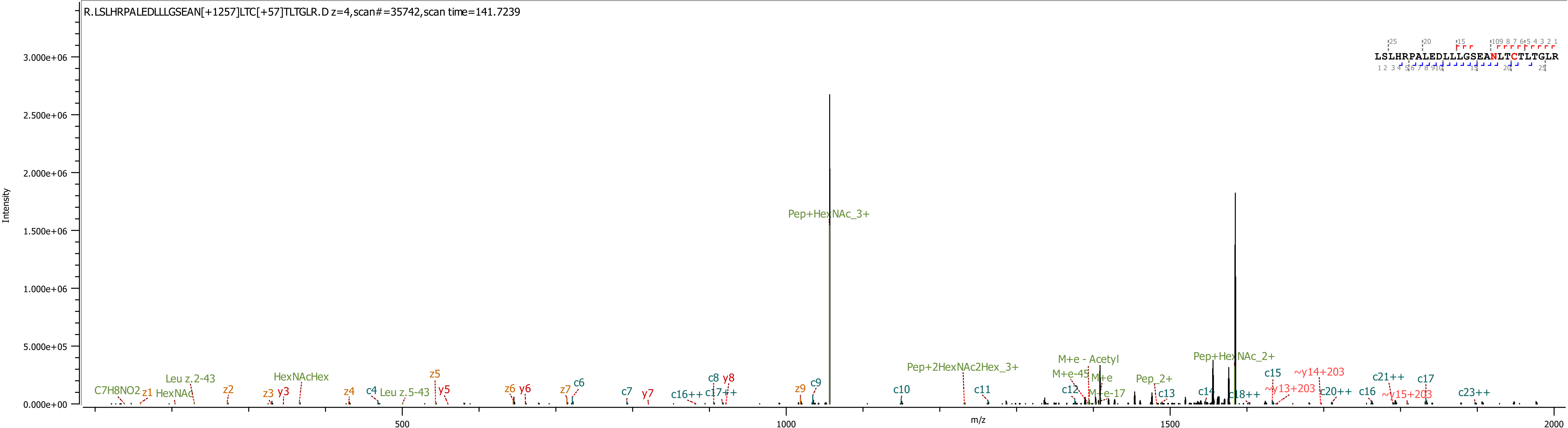
R.LSLHRPALEDLLLGSEAN[+1257]LTC[+57]TLTGLR.D z=4,scan#=35742,scan time=141.7239
LSLHRPALEDLLLGSEANLTCTLTGLR
Intensity
m/z
Pep+HexNAc_3+
Pep+HexNAc_2+
Pep+2HexNAc2Hex_3+
M+e - Acetyl
M+e-45
M+e
M+e-17
Pep_2+
C7H8NO2
HexNAc
HexNAcHex
Leu z.2-43
Leu z.5-43
~y14+203
~y13+203
~y15+203

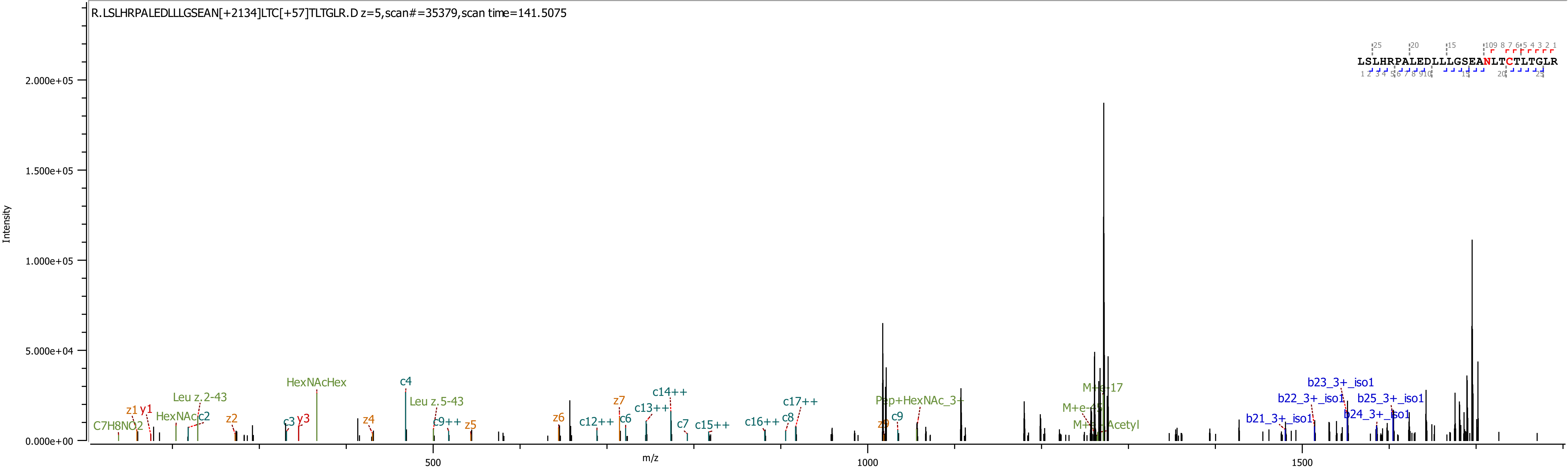
R.LSLHRPALEDLLLGSEAN[+2134]LTC[+57]TLTGLR.D z=5,scan#=35379,scan time=141.5075
LSLHRPALEDLLLGSEANLTCTLTGLR
Intensity
m/z
2.000e+05
1.500e+05
1.000e+05
5.000e+04
0.000e+00
500
1000
1500
C7H8NO2
z1
y1
HexNAc
Leu z.2-43
c2
z2
c3
y3
HexNAcHex
z4
c4
Leu z.5-43
c9++
z5
z6
c12++
z7
c6
c13++
c14++
c7
c15++
c16++
c8
c17++
z9
c9
Pep+HexNAc_3+
M+e-17
M+e-45
M+e-Acetyl
b21_3+_iso1
b22_3+_iso1
b23_3+_iso1
b24_3+_iso1
b25_3+_iso1

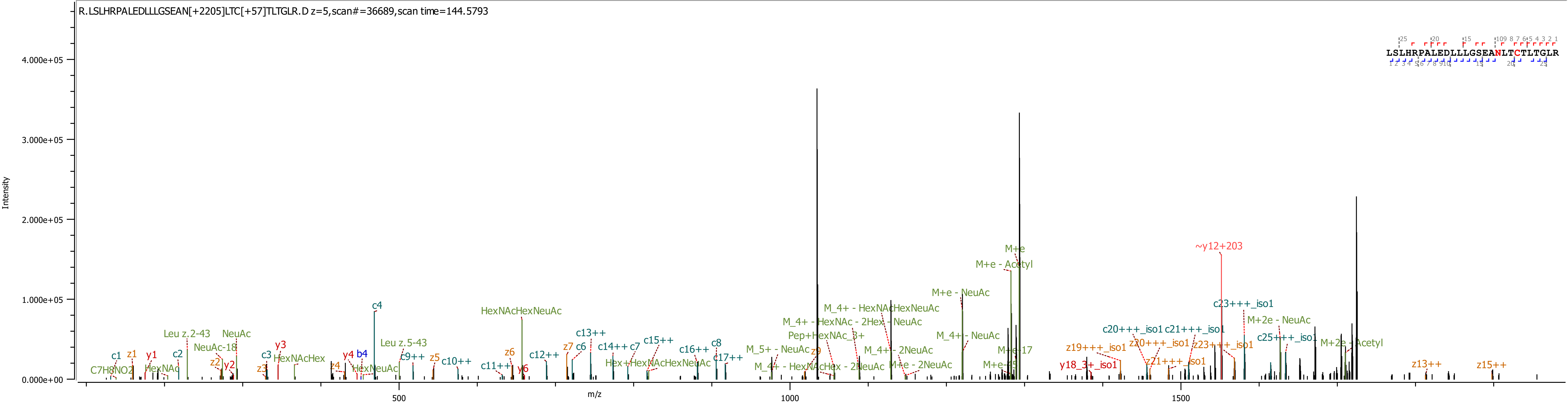
R.LSLHRPALEDLLLGSEAN[+2205]LTC[+57]TLTGLR.D z=5,scan#=36689,scan time=144.5793
LSLHRPALEDLLLGSEANLTCTLTGLR
Intensity
m/z
4.000e+05
3.000e+05
2.000e+05
1.000e+05
0.000e+00
500
1000
1500
c1
z1
y1
c2
C7H8NO2
HexNAc
Leu z.2-43
NeuAc-18
NeuAc
z2
y2
z3
c3
y3
HexNAcHex
z4
y4
b4
HexNeuAc
c4
Leu z.5-43
c9++
z5
c10++
c11++
y6
z6
HexNAcHexNeuAc
c12++
z7
c6
c13++
c14++
c7
Hex+HexNAcHexNeuAc
c15++
c16++
c8
c17++
M_5+ - NeuAc
M_4+ - HexNAcHex - 2NeuAc
z9
Pep+HexNAc_3+
M_4+ - HexNAc - 2Hex - NeuAc
M_4+ - HexNAcHexNeuAc
M_4+ - 2NeuAc
M+e - 2NeuAc
M_4+ - NeuAc
M+e - NeuAc
M+e - Acetyl
M+e
M+e-17
M+e-45
y18_3+_iso1
z19+++_iso1
c20+++_iso1
z20+++_iso1
z21+++_iso1
c21+++_iso1
~y12+203
z23+++_iso1
c23+++_iso1
M+2e - NeuAc
c25+++_iso1
M+2e - Acetyl
z13++
z15++

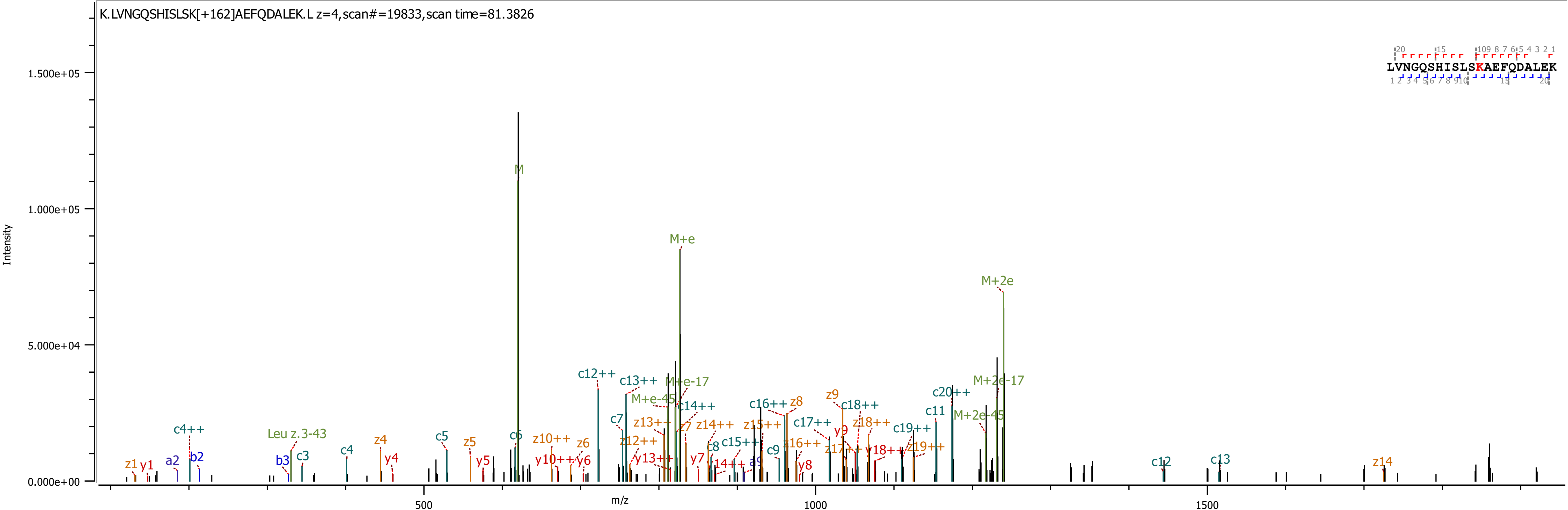

K.LVNGQSHISLSK[+162]AEFQDALEK.L z=4,scan#=19833,scan time=81.3826
LVNGQSHISLSKAEFQDALEK
Intensity
m/z
0.000e+00
5.000e+04
1.000e+05
1.500e+05
500
1000
1500
M
M+e
M+2e
M+e-17
M+e-45
M+2e-17
M+2e-45
Leu z.3-43
c4++
c12++
c13++
c14++
c16++
c18++
c20++
c17++
c19++
c15++
c11
c12
c13
z14
z8
z9
z18++
z13++
z14++
z15++
z12++
z10++
z16++
z17++
z19++
y10++
y13++
y18++
c7
c8
c9
c5
c6
c4
c3
z4
z5
z6
z7
y4
y5
y6
y7
y8
y9
z1
y1
a2
b2
b3
a9

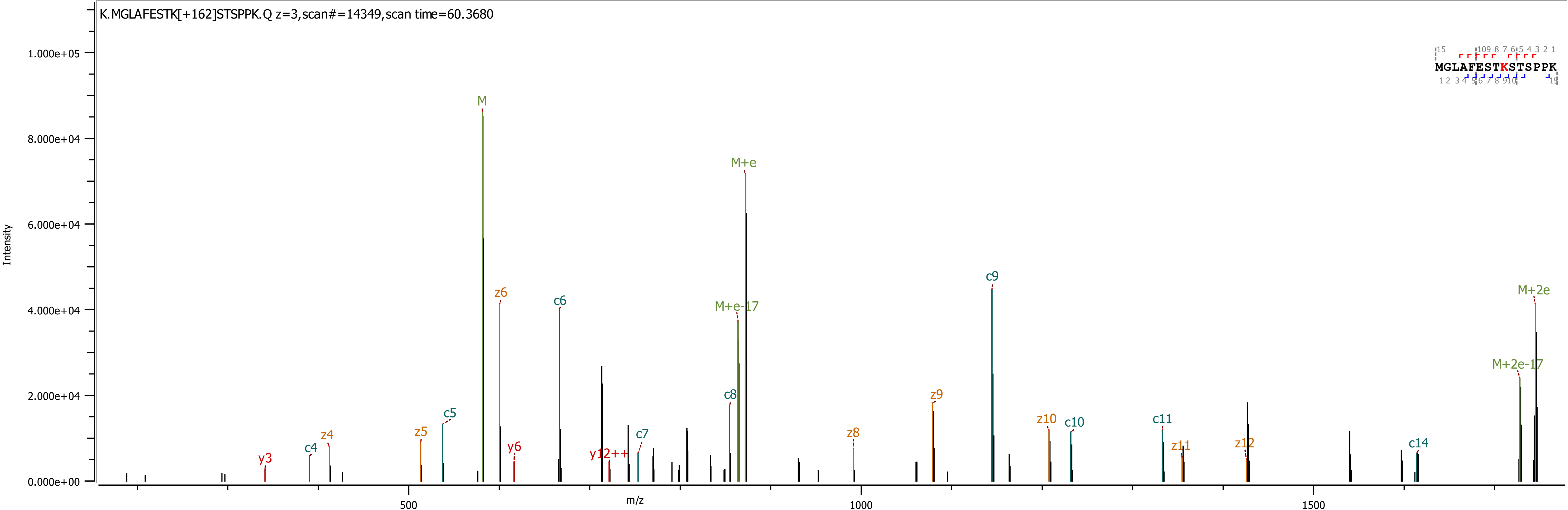
K.MGLAFESTK[+162]STSPPK.Q z=3,scan#=14349,scan time=60.3680
Intensity
m/z
MGLAFESTKSTSPPK
M
M+e
M+e-17
M+2e
M+2e-17
y3
c4
z4
z5
c5
y6
z6
c6
y12++
c7
c8
z8
z9
c9
z10
c10
c11
z11
z12
c14
0.000e+00
2.000e+04
4.000e+04
6.000e+04
8.000e+04
1.000e+05
500
1000
1500

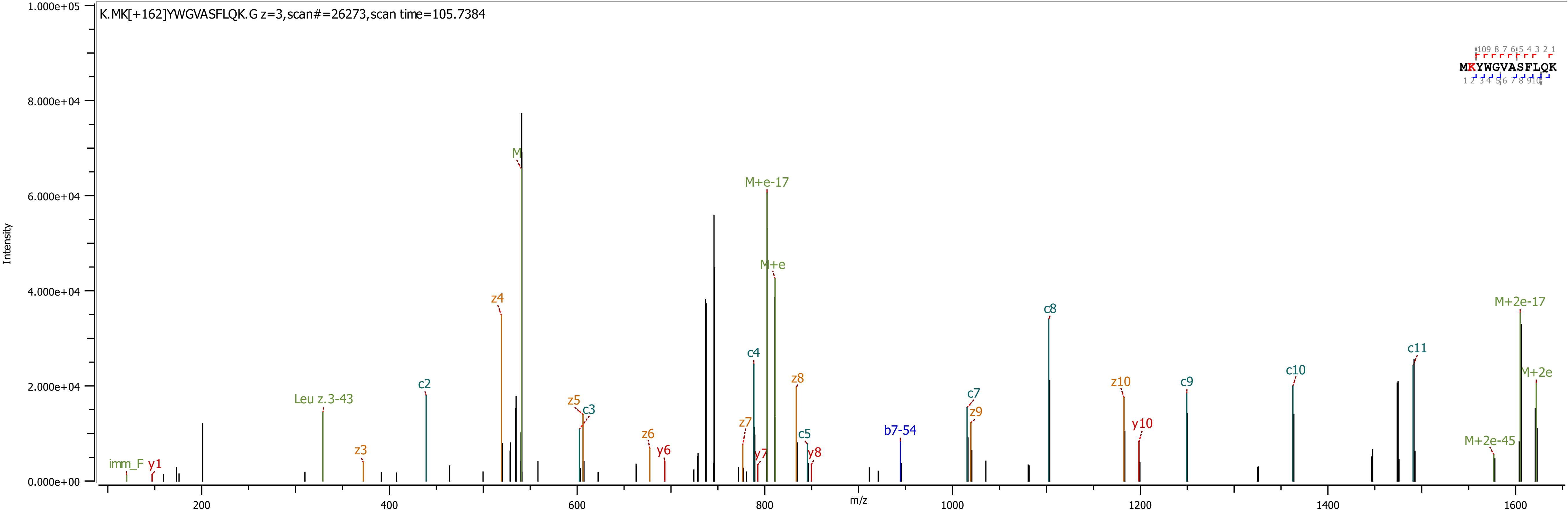
K.MK[+162]YWGVASFLQK.G z=3,scan#=26273,scan time=105.7384
MKYWGVASFLQK
Intensity
m/z
1.000e+05
8.000e+04
6.000e+04
4.000e+04
2.000e+04
0.000e+00
200
400
600
800
1000
1200
1400
1600
imm_F
y1
Leu z.3-43
z3
c2
z4
M
z5
c3
z6
y6
z7
y7
c4
M+e-17
M+e
z8
c5
y8
b7-54
c7
z9
c8
z10
y10
c9
c10
c11
M+2e-45
M+2e-17
M+2e

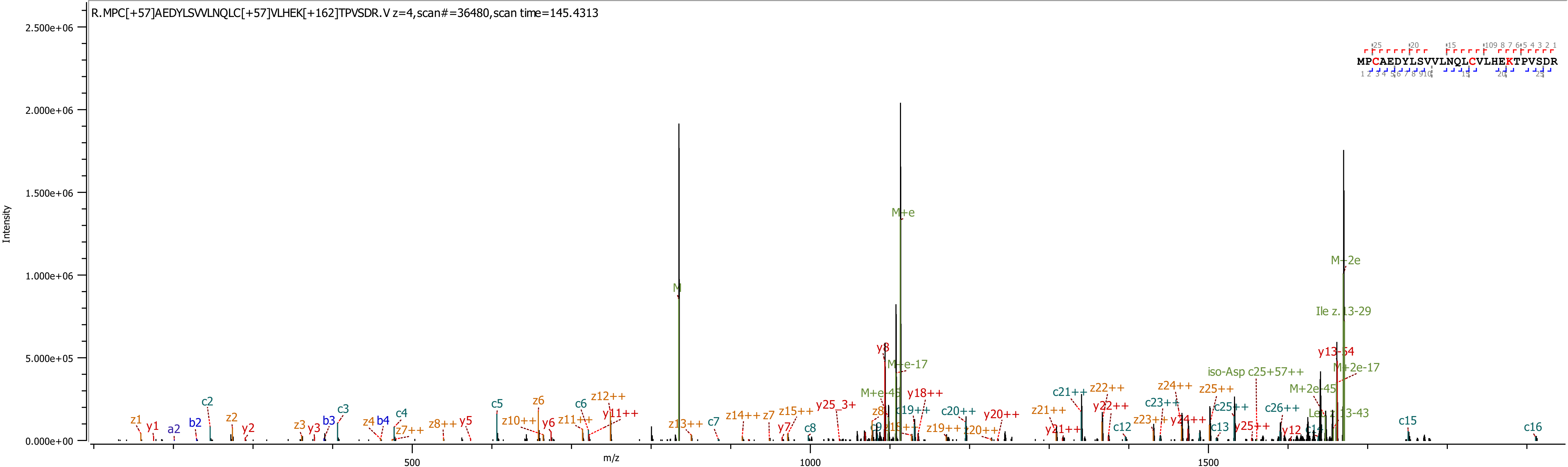
R.MPC[+57]AEDYLSVVLNQLC[+57]VLHEK[+162]TPVSDR.V z=4,scan#=36480,scan time=145.4313
MPCAEDYLSVVLNQLCVLHEKTPVSDR
Intensity
m/z
2.500e+06
2.000e+06
1.500e+06
1.000e+06
5.000e+05
0.000e+00
500
1000
1500
M
M+e
M+2e
M+e-17
M+e-45
M+2e-17
M+2e-45
Ile z13-29
iso-Asp c25+57++
y8
y13-54

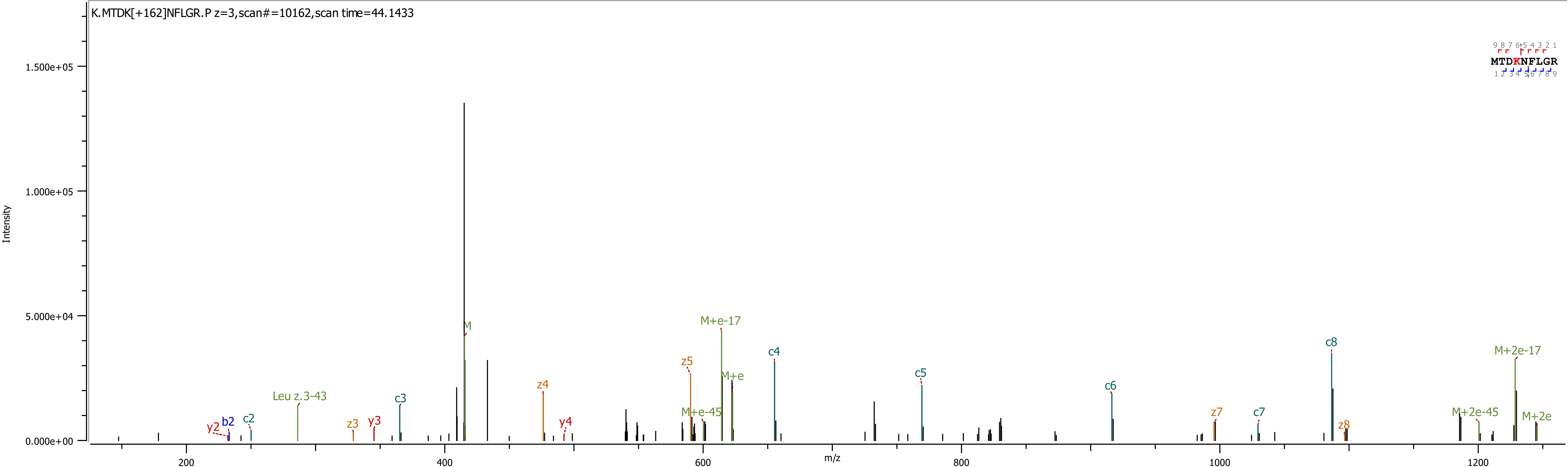
K.MTDK[+162]NFLGR.P z=3,scan#=10162,scan time=44.1433
1.500e+05
1.000e+05
5.000e+04
0.000e+00
Intensity
m/z
200
400
600
800
1000
1200
MTDKNFLGR
y2
b2
c2
Leu z.3-43
z3
y3
c3
M
z4
y4
z5
M+e-45
M+e-17
M+e
c4
c5
c6
z7
c7
c8
z8
M+2e-45
M+2e-17
M+2e

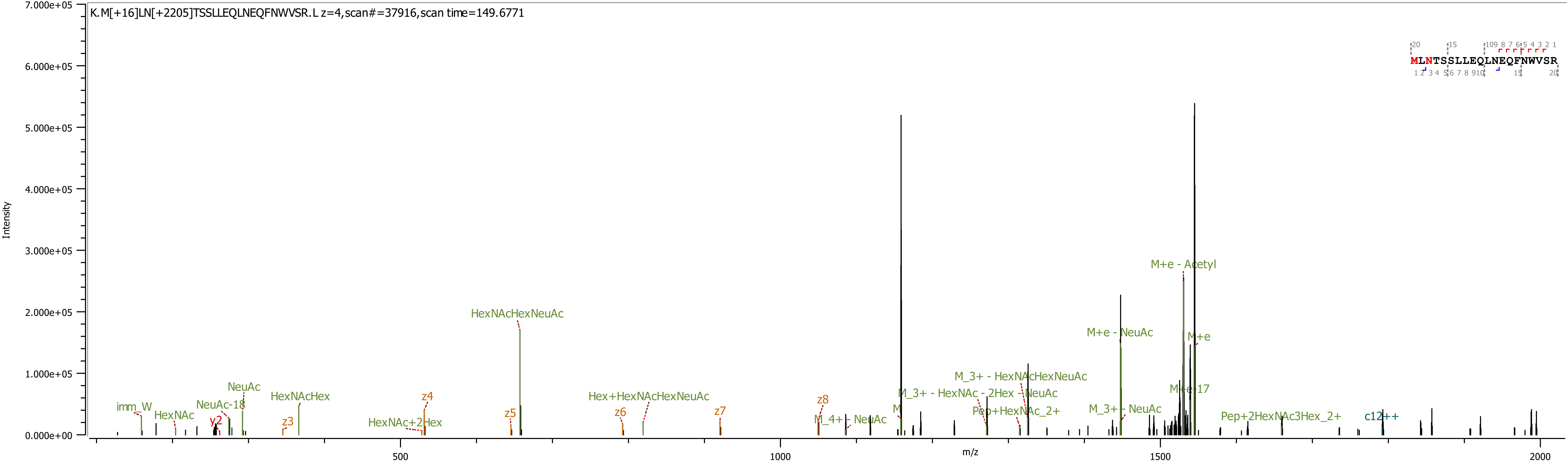
K.M[+16]LN[+2205]TSSLLEQLNEQFNWVSR.L z=4,scan#=37916,scan time=149.6771
MLNTSSLLEQLNEQFNWVSR
Intensity
m/z
7.000e+05
6.000e+05
5.000e+05
4.000e+05
3.000e+05
2.000e+05
1.000e+05
0.000e+00
500
1000
1500
2000
imm_W
HexNAc
y2
NeuAc-18
NeuAc
z3
HexNAcHex
HexNAc+2Hex
z4
z5
HexNAcHexNeuAc
z6
Hex+HexNAcHexNeuAc
z7
z8
M_4+ - NeuAc
M_3+ - HexNAc - 2Hex - NeuAc
M_3+ - HexNAcHexNeuAc
Pep+HexNAc_2+
M+e - NeuAc
M_3+ - NeuAc
M+e - Acetyl
M+e
M+e-17
Pep+2HexNAc3Hex_2+
c12++

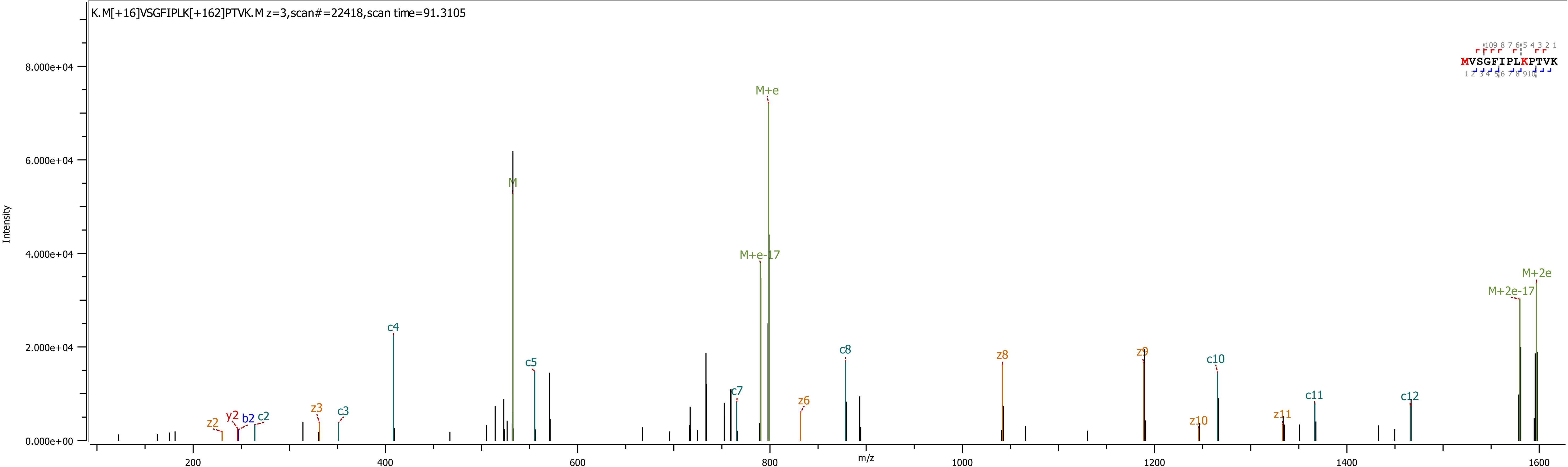
K.M[+16]VSGFIPLK[+162]PTVK.M z=3,scan#=22418,scan time=91.3105
Intensity
m/z
M+e
M
M+e-17
M+2e
M+2e-17
c2
c3
c4
c5
c7
c8
c10
c11
c12
z2
z3
z6
z8
z9
z10
z11
y2
b2
MVSGFIPLKPTVK

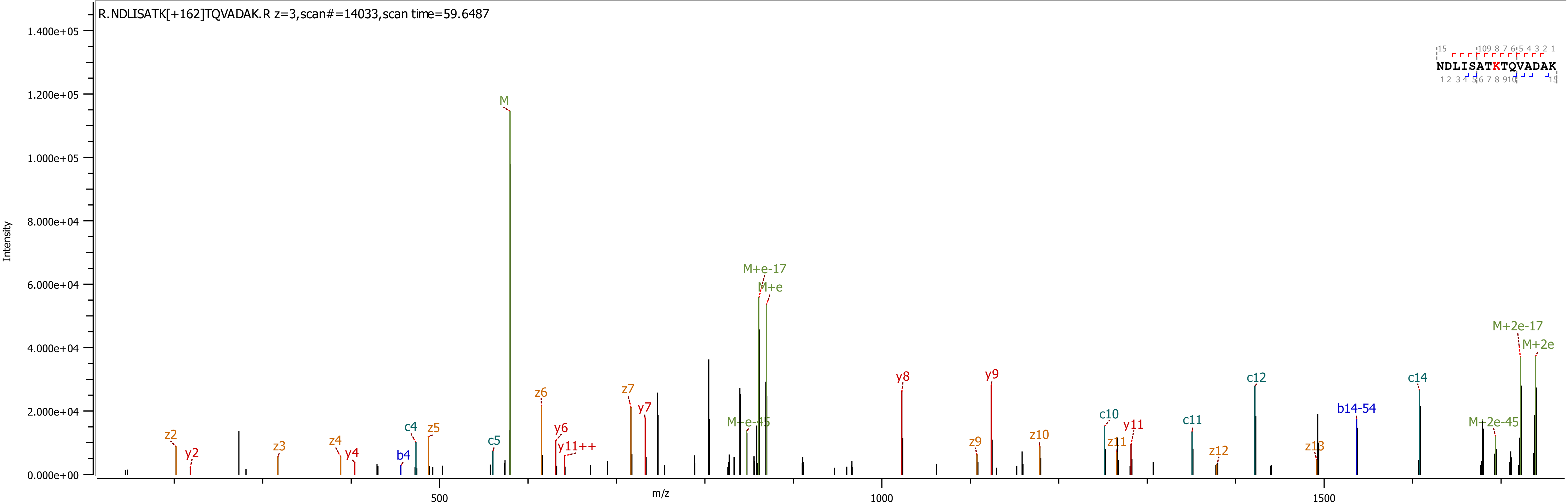
R.NDLISATK[+162]TQVADAK.R z=3,scan#=14033,scan time=59.6487
Intensity
m/z
0.000e+00
2.000e+04
4.000e+04
6.000e+04
8.000e+04
1.000e+05
1.200e+05
1.400e+05
500
1000
1500
NDLISATKTQVADAK
M
M+e-17
M+e
M+e-45
M+2e-17
M+2e
M+2e-45
z2
y2
z3
z4
y4
b4
c4
z5
c5
z6
y6
y11++
z7
y7
y8
y9
z9
z10
c10
z11
y11
c11
z12
c12
z13
b14-54
c14

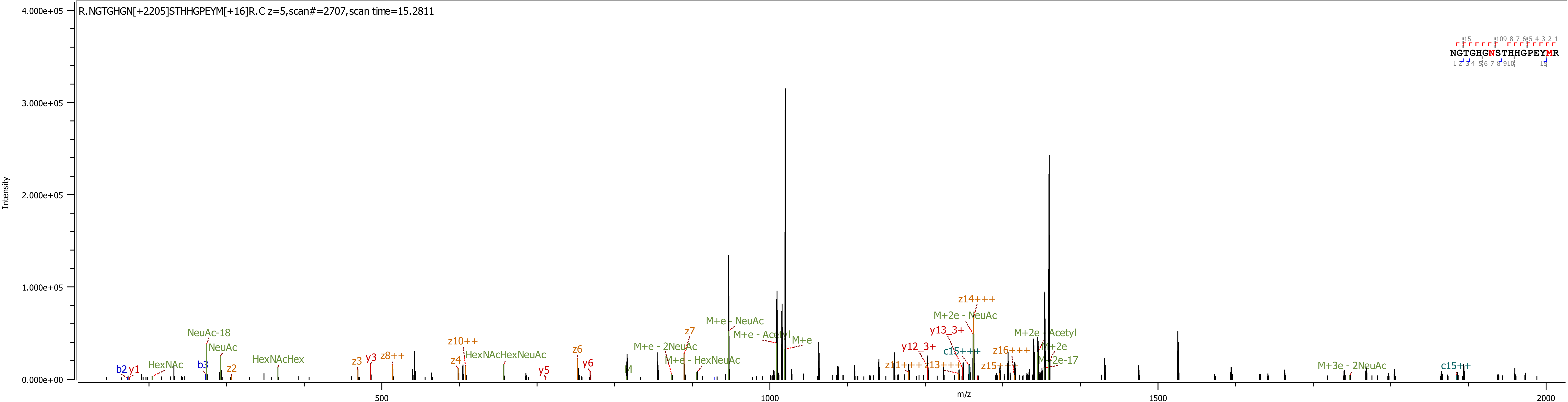
R.NGTGHGN[+2205]STHHGPEYM[+16]R.C z=5,scan#=2707,scan time=15.2811
Intensity
m/z
NGTGHGNSTHHGPEYMR
4.000e+05
3.000e+05
2.000e+05
1.000e+05
0.000e+00
500
1000
1500
2000
b2
y1
HexNAc
NeuAc-18
NeuAc
b3
z2
HexNAcHex
z3
y3
z8++
z10++
z4
HexNAcHexNeuAc
y5
z6
y6
M
M+e - 2NeuAc
z7
M+e - HexNeuAc
M+e - NeuAc
M+e - Acetyl
M+e
y12_3+
y13_3+
z11+++
z13+++
c15+++
z14+++
M+2e - NeuAc
z15+++
z16+++
M+2e - Acetyl
M+2e
M+2e-17
M+3e - 2NeuAc
c15++

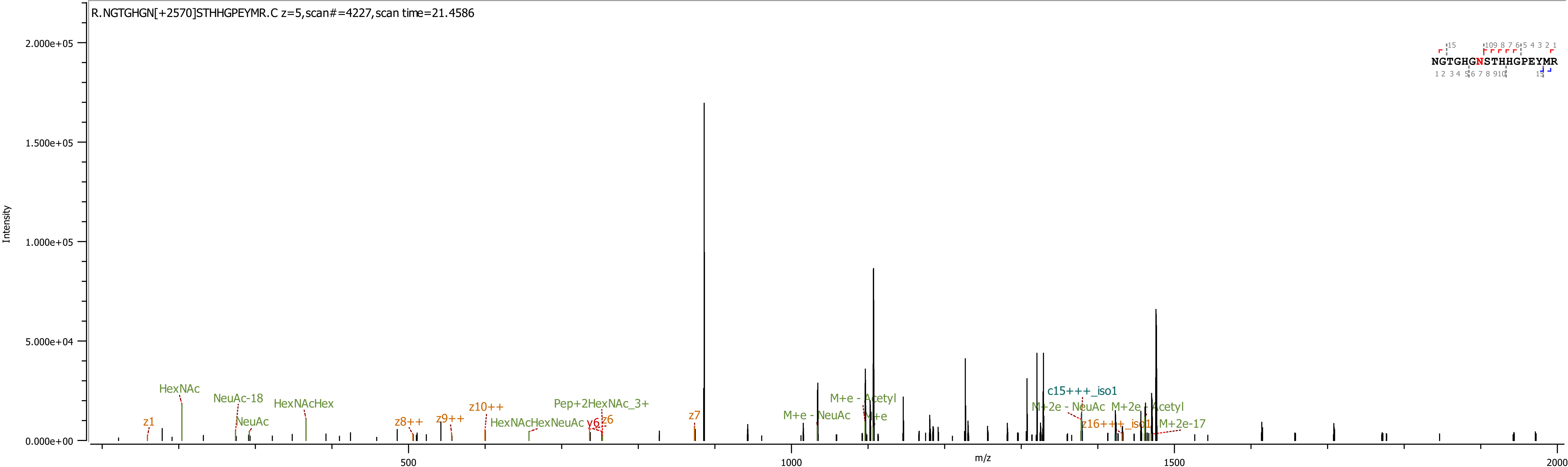
R.NGTGHGN[+2570]STHHGPEYMR.C z=5,scan#=4227,scan time=21.4586
Intensity
2.000e+05
1.500e+05
1.000e+05
5.000e+04
0.000e+00
m/z
500
1000
1500
2000
NGTGHGNSTHHGPEYMR
HexNAc
NeuAc-18
NeuAc
HexNAcHex
z1
z8++
z9++
z10++
HexNAcHexNeuAc
Pep+2HexNAc_3+
y6
z6
z7
M+e - NeuAc
M+e - Acetyl
M+e
c15+++_iso1
M+2e - NeuAc
M+2e - Acetyl
z16+++_iso1
M+2e-17

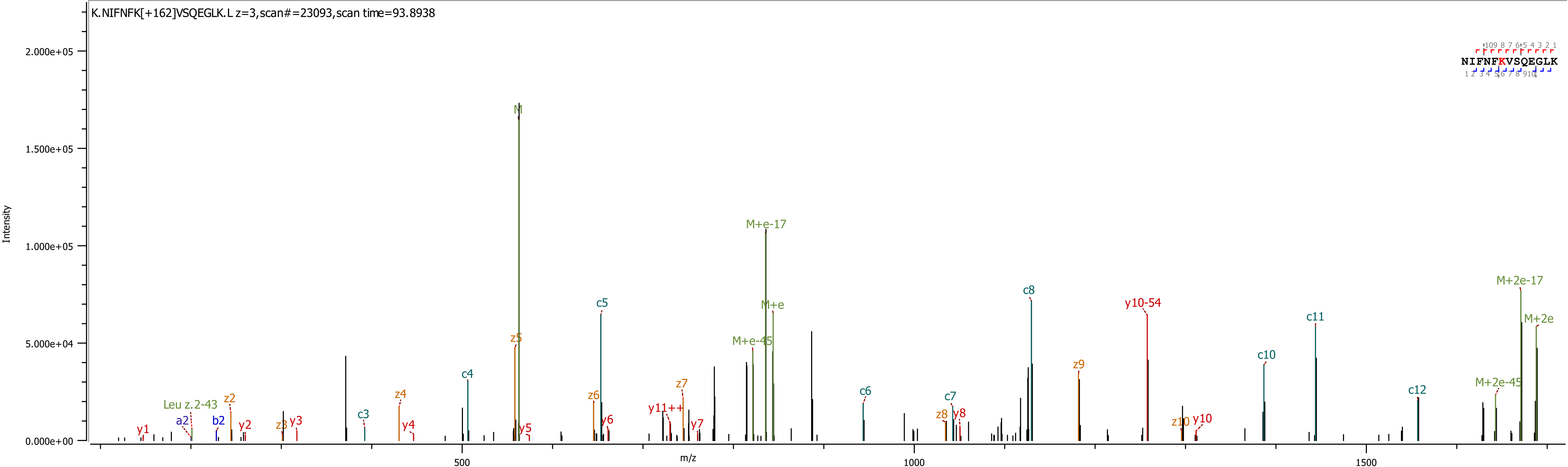

K.NIFNFK[+162]VSQEGLK.L z=3,scan#=23093,scan time=93.8938
Intensity
m/z
NIFNFKVSQEGLK
2.000e+05
1.500e+05
1.000e+05
5.000e+04
0.000e+00
500
1000
1500
M
M+e-17
M+e
M+e-45
c5
c8
y10-54
c11
c10
M+2e-17
M+2e
M+2e-45
c12
z9
z5
c4
z4
c6
c7
z8
y8
z10
y10
z6
y6
z7
y11++
y7
y5
y4
c3
z3
y3
z2
y2
b2
a2
y1
Leu z.2-43

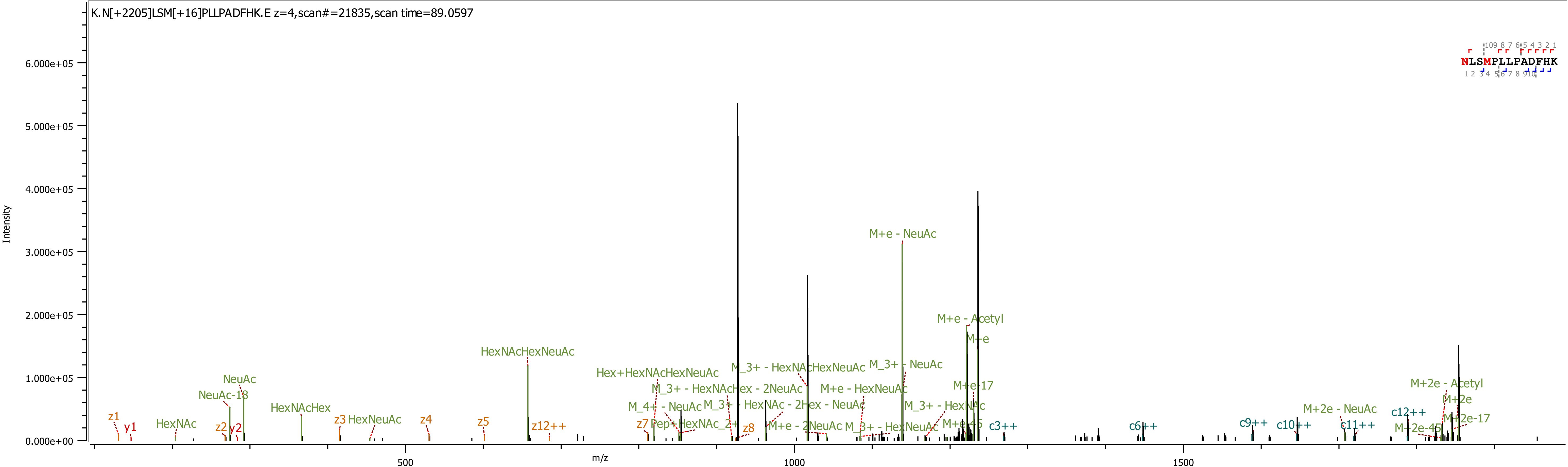
K.N[+2205]LSM[+16]PLLPADFHK.E z=4,scan#=21835,scan time=89.0597
Intensity
m/z
6.000e+05
5.000e+05
4.000e+05
3.000e+05
2.000e+05
1.000e+05
0.000e+00
500
1000
1500
NLSMPLLPADFHK
M+e - NeuAc
M+e - Acetyl
M+e
HexNAcHexNeuAc
Hex+HexNAcHexNeuAc
M_3+ - HexNAcHexNeuAc
M_3+ - NeuAc
M_3+ - HexNAcHex - 2NeuAc
M+e - HexNeuAc
M+e-17
M_4+ - NeuAc
M_3+ - HexNAc - 2Hex - NeuAc
M_3+ - HexNAc
NeuAc
NeuAc-18
HexNAcHex
HexNAc
HexNeuAc
z1
y1
z2
y2
z3
z4
z5
z12++
z7
Pep+HexNAc_2+
z8
M+e - 2NeuAc
M_3+ - HexNeuAc
c3++
c6++
c9++
c10++
c11++
c12++
M+2e - NeuAc
M+2e - Acetyl
M+2e
M+2e-17
M+2e-45

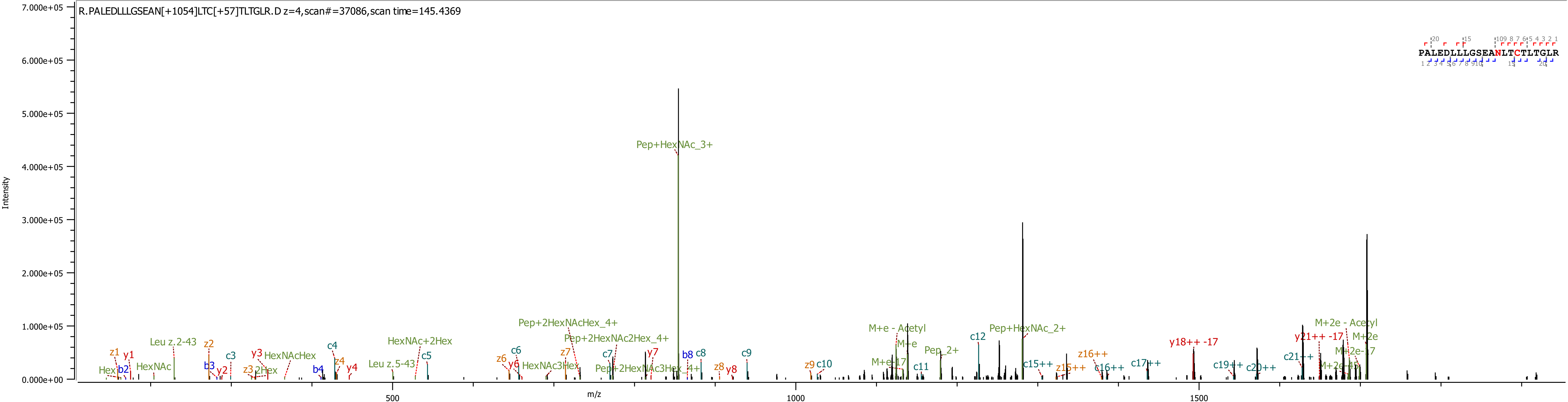
R.PALEDLLLGSEAN[+1054]LTC[+57]TLTGLR.D z=4,scan#=37086,scan time=145.4369
PALEDLLLGSEANLTCTLTGLR
Intensity
m/z
Pep+HexNAc_3+
Pep+HexNAc_2+
Pep+2HexNAcHex_4+
Pep+2HexNAc2Hex_4+
Pep+2HexNAc3Hex_4+
HexNAc3Hex
HexNAc+2Hex
HexNAcHex
HexNAc
Hex
2Hex
Leu z.2-43
Leu z.5-43
M+e - Acetyl
M+e
M+e-17
Pep_2+
M+2e - Acetyl
M+2e
M+2e-17
y18++ -17
y21++ -17
z16++
z15++
c15++
c16++
c17++
c19++
c20++
c21++

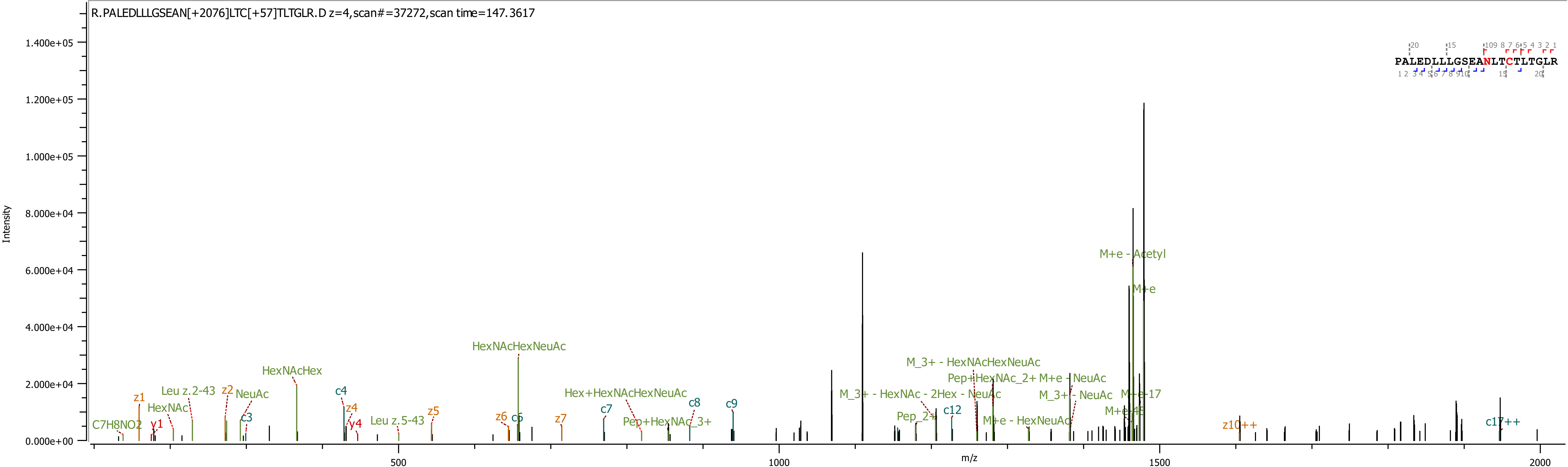
R.PALEDLLLGSEAN[+2076]LTC[+57]TLTGLR.D z=4,scan#=37272,scan time=147.3617
PALEDLLLGSEANLTCTLTGLR
Intensity
m/z
1.400e+05
1.200e+05
1.000e+05
8.000e+04
6.000e+04
4.000e+04
2.000e+04
0.000e+00
500
1000
1500
2000
M+e - Acetyl
M+e
HexNAcHexNeuAc
HexNAcHex
M_3+ - HexNAcHexNeuAc
Pep+HexNAc_2+
M+e - NeuAc
M_3+ - HexNAc - 2Hex - NeuAc
M_3+ - NeuAc
M+e-17
M+e-45
Hex+HexNAcHexNeuAc
Pep+HexNAc_3+
Pep_2+
M+e - HexNeuAc
Leu z.2-43
Leu z.5-43
NeuAc
HexNAc
C7H8NO2
z1
y1
z2
c3
c4
z4
y4
z5
z6
c6
z7
c7
c8
c9
c12
z10++
c17++

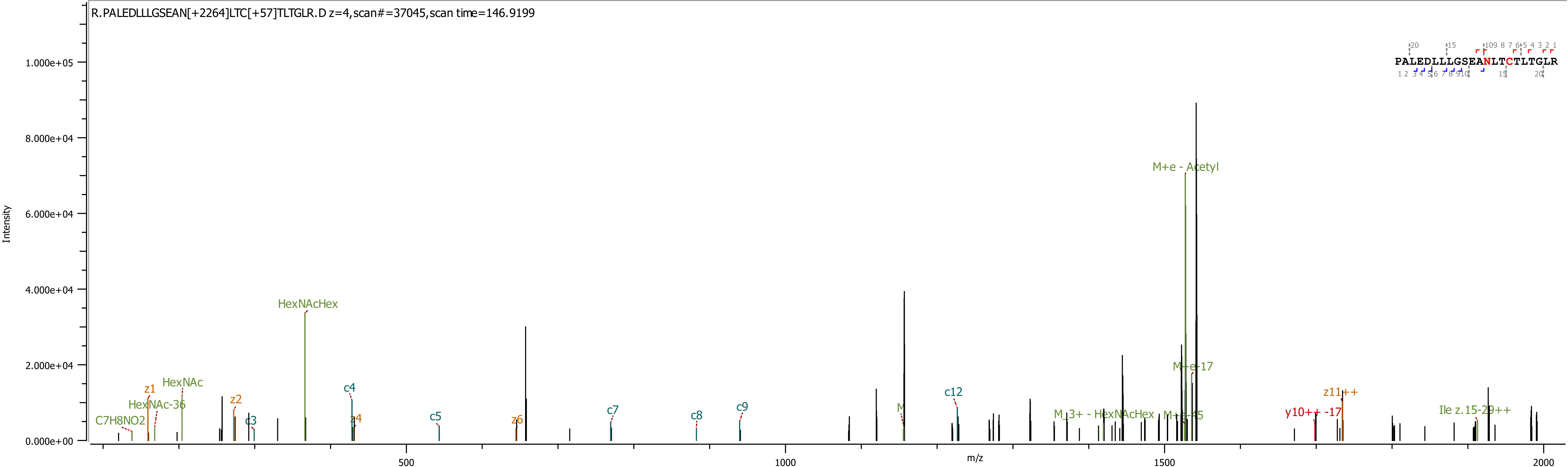
R.PALEDLLLGSEAN[+2264]LTC[+57]TLTGLR.D z=4,scan#=37045,scan time=146.9199
PALEDLLLGSEANLTCTLTGLR
Intensity
m/z
1.000e+05
8.000e+04
6.000e+04
4.000e+04
2.000e+04
0.000e+00
500
1000
1500
2000
M+e - Acetyl
HexNAcHex
M+e-17
C7H8NO2
z1
HexNAc-36
HexNAc
z2
c3
c4
z4
c5
z6
c7
c8
c9
c12
M_3+ - HexNAcHex
y10++ -17
z11++
Ile z.15-20++

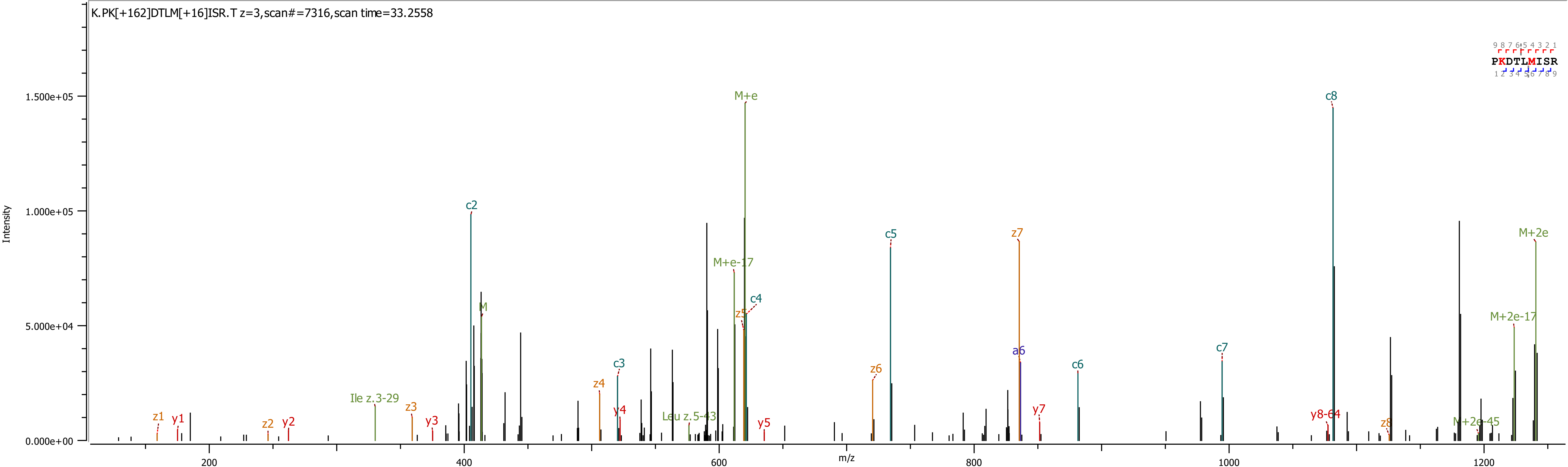
K.PK[+162]DTLM[+16]ISR.T z=3,scan#=7316,scan time=33.2558
PKDTLMISR
Intensity
m/z
1.500e+05
1.000e+05
5.000e+04
0.000e+00
200
400
600
800
1000
1200
z1
y1
z2
y2
Ile z.3-29
z3
y3
c2
M
z4
c3
y4
Leu z.5-43
M+e-17
z5
c4
M+e
y5
z6
c5
z7
a6
y7
c6
c7
y8-64
c8
z8
M+2e-45
M+2e-17
M+2e

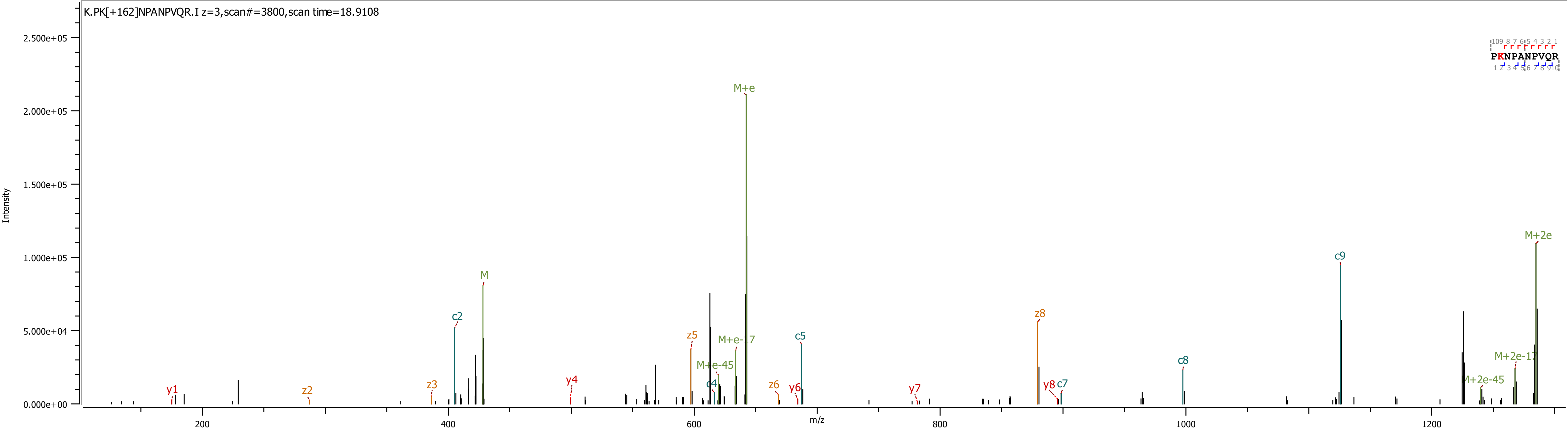

K.PK[+162]NPANPVQR.I z=3,scan#=3800,scan time=18.9108
Intensity
m/z
PKNPANPVQR
M+e
M
c2
c4
c5
c7
c8
c9
y1
y4
y6
y7
y8
z2
z3
z5
z6
z8
M+e-45
M+e-17
M+2e
M+2e-17
M+2e-45
2.500e+05
2.000e+05
1.500e+05
1.000e+05
5.000e+04
0.000e+00
200
400
600
800
1000
1200

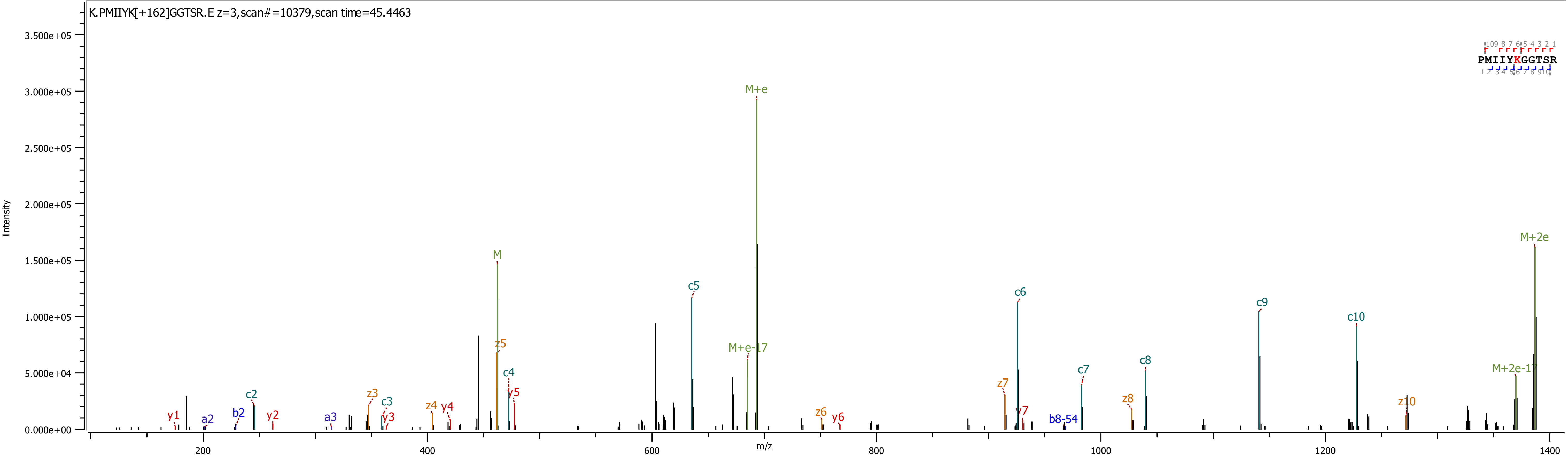

K.PMIIYK[+162]GGTSR.E z=3,scan#=10379,scan time=45.4463
Intensity
m/z
PMIIYKGGTSR
M+e
M+2e
M
c5
c6
c9
c10
M+e-17
M+2e-17
z5
c4
y5
c7
c8
z7
z8
z10
c2
z3
c3
z4
y4
y1
a2
b2
y2
a3
y3
z6
y6
y7
b8-54

K.QDSLSSQNQLGVLPLSWDIPELVNMGQWK[+162]IR.A z=4,scan#=38754,scan time=151.8909

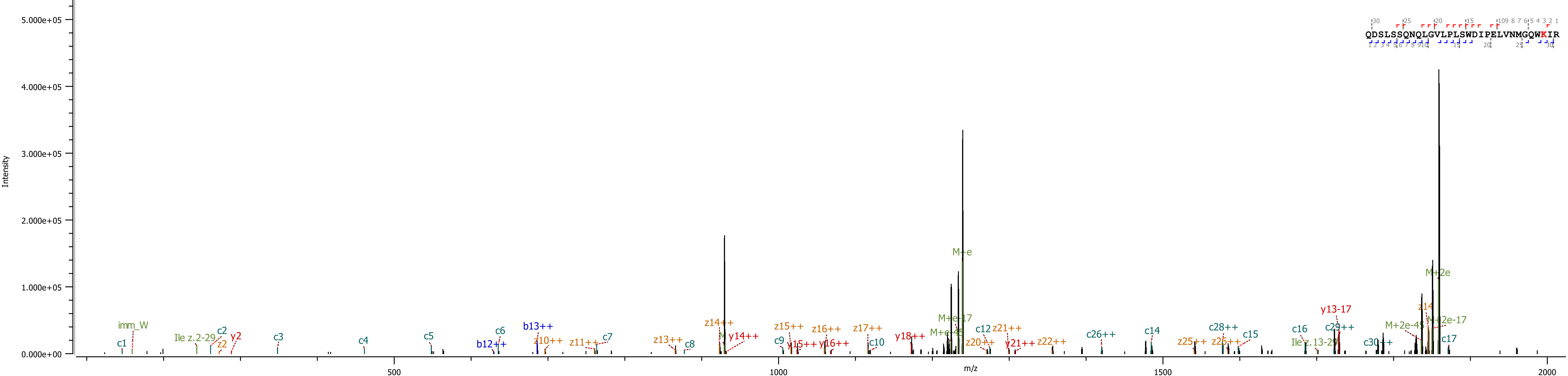

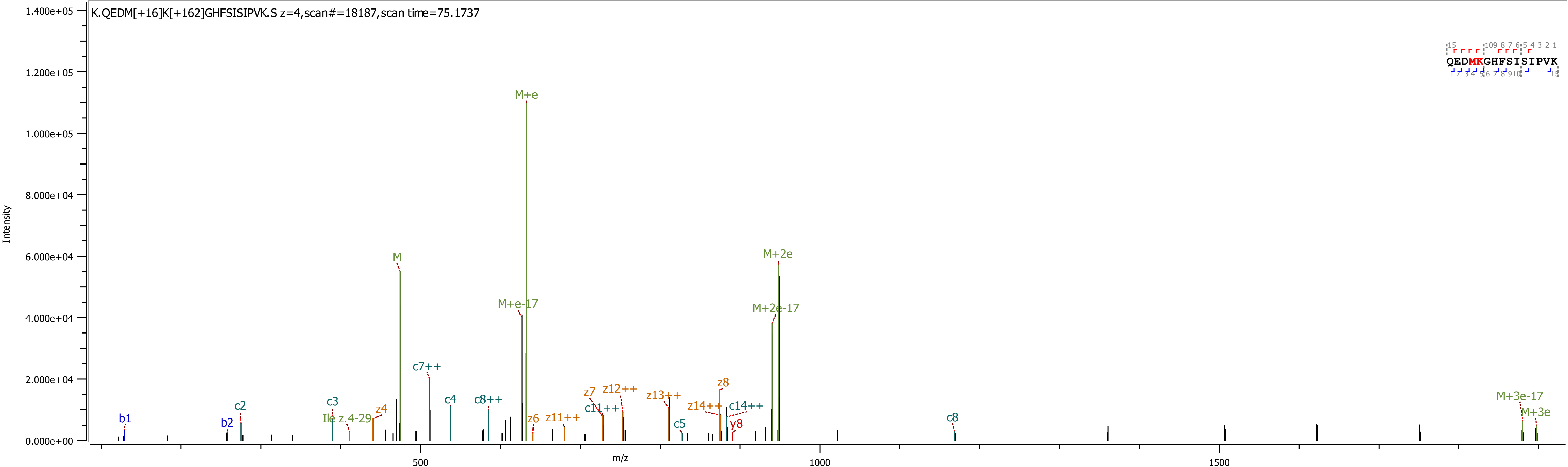
K.QEDM[+16]K[+162]GHFSISIPVK.S z=4,scan#=18187,scan time=75.1737
Intensity
m/z
QEDMKGHFSISIPVK
M+e
M
M+e-17
M+2e
M+2e-17
M+3e-17
M+3e
b1
b2
c2
c3
c4
c5
c7++
c8++
c8
c11++
c14++
Ile
z.4-29
z4
z6
z7
z8
z11++
z12++
z13++
z14++
y8

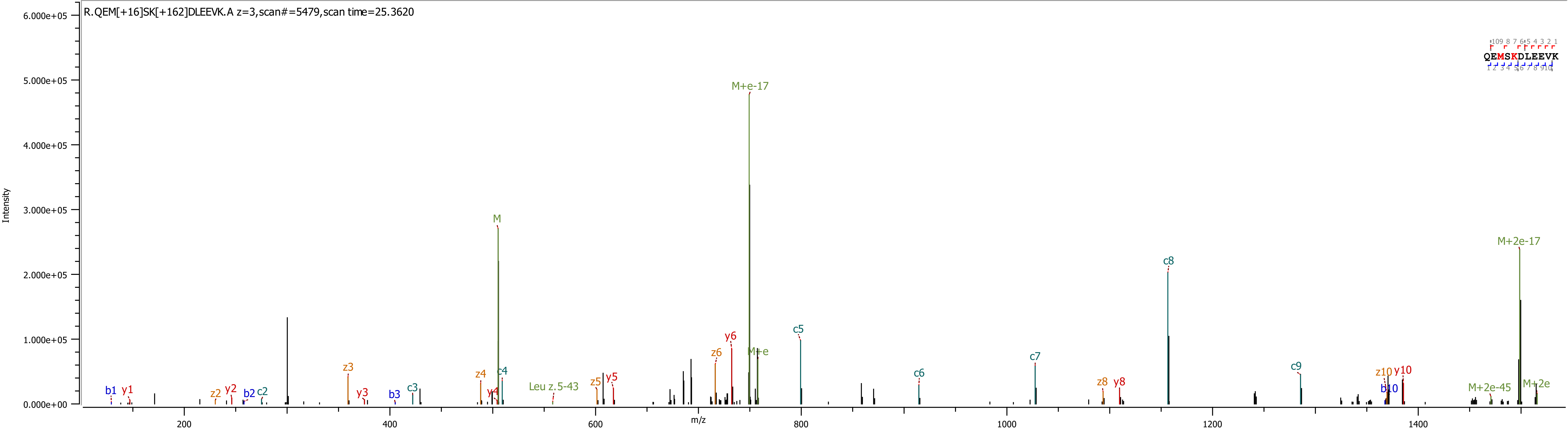

R.QEM[+16]SK[+162]DLEEVK.A z=3,scan#=5479,scan time=25.3620
Intensity
m/z
6.000e+05
5.000e+05
4.000e+05
3.000e+05
2.000e+05
1.000e+05
0.000e+00
200
400
600
800
1000
1200
1400
QEMSKDLEEVK
M+e-17
M
M+2e-17
c8
c5
y6
z6
M+e
c7
c6
c9
z10
y10
b10
z8
y8
M+2e-45
M+2e
z3
z4
c4
y4
Leu z.5-43
z5
y5
b1
y1
z2
y2
b2
c2
y3
b3
c3

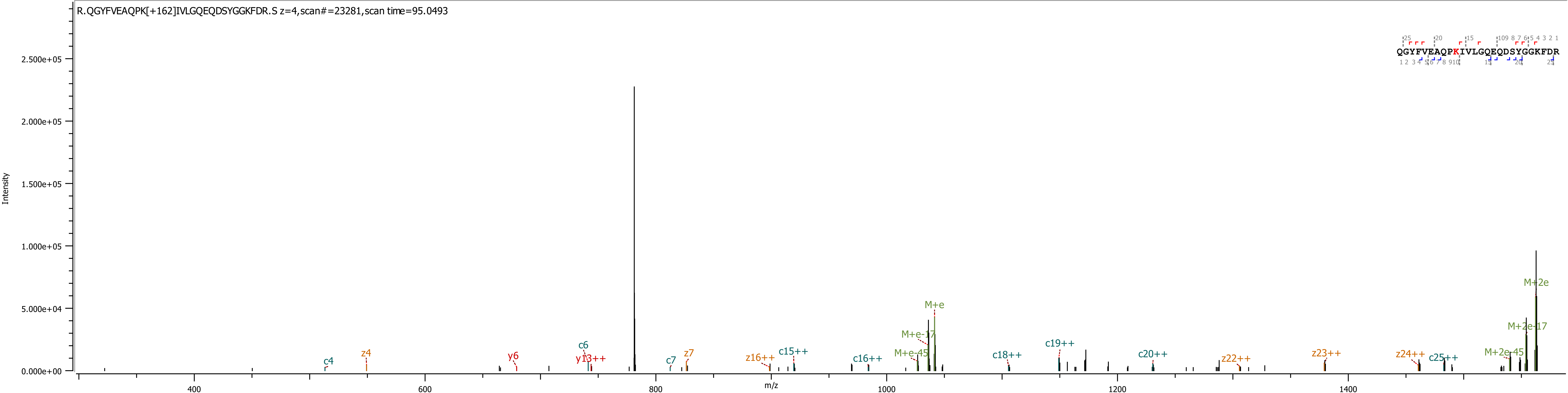
R.QGYFVEAQPK[+162]IVLGQEQDSYGGKFDR.S z=4,scan#=23281,scan time=95.0493
QGYFVEAQPKIVLGQEQDSYGGKFDR
Intensity
m/z
M+2e
M+2e-17
M+2e-45
M+e
M+e-17
M+e-45
c4
z4
y6
c6
y13++
c7
z7
z16++
c15++
c16++
c18++
c19++
c20++
z22++
z23++
z24++
c25++

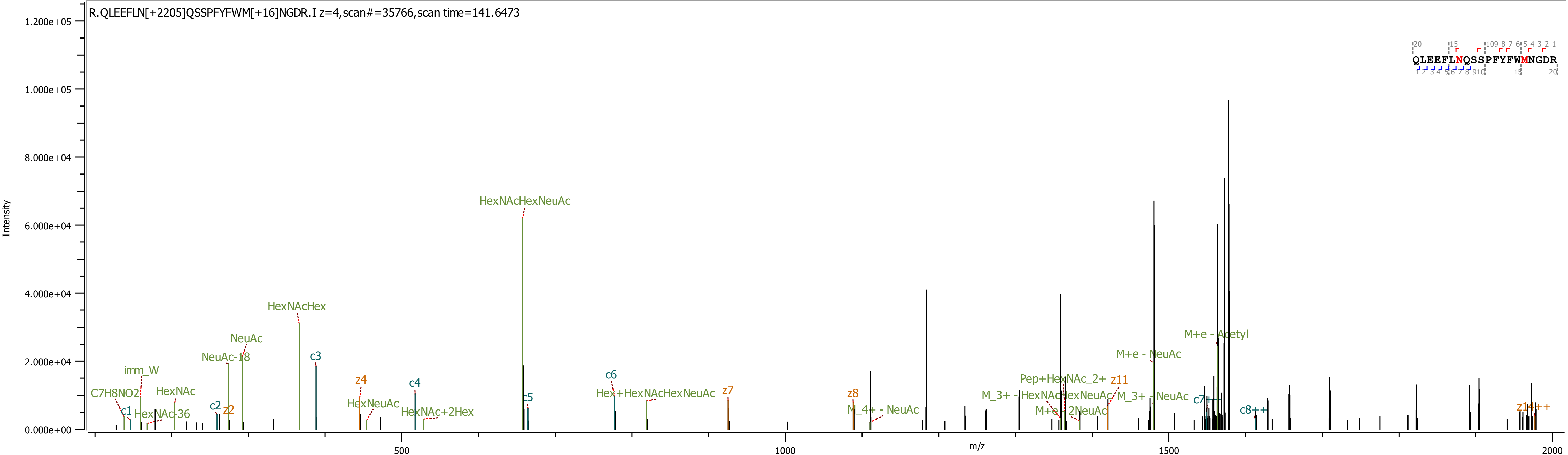

R.QLEEFLN[+2205]QSSPFYFWM[+16]NGDR.I z=4,scan#=35766,scan time=141.6473
QLEEFLNQSSPFYFWMNGDR
Intensity
m/z
1.200e+05
1.000e+05
8.000e+04
6.000e+04
4.000e+04
2.000e+04
0.000e+00
500
1000
1500
2000
HexNAcHexNeuAc
HexNAcHex
NeuAc
NeuAc-18
c3
imm_W
C7H8NO2
HexNAc
c1
HexNAc-36
c2
z2
z4
c4
HexNeuAc
HexNAc+2Hex
c5
c6
Hex+HexNAcHexNeuAc
z7
z8
M_4+ - NeuAc
M+e - Acetyl
M+e - NeuAc
Pep+HexNAc_2+
z11
M_3+ - HexNAcHexNeuAc
M_3+ - NeuAc
M+e - 2NeuAc
c7++
c8++
z14++

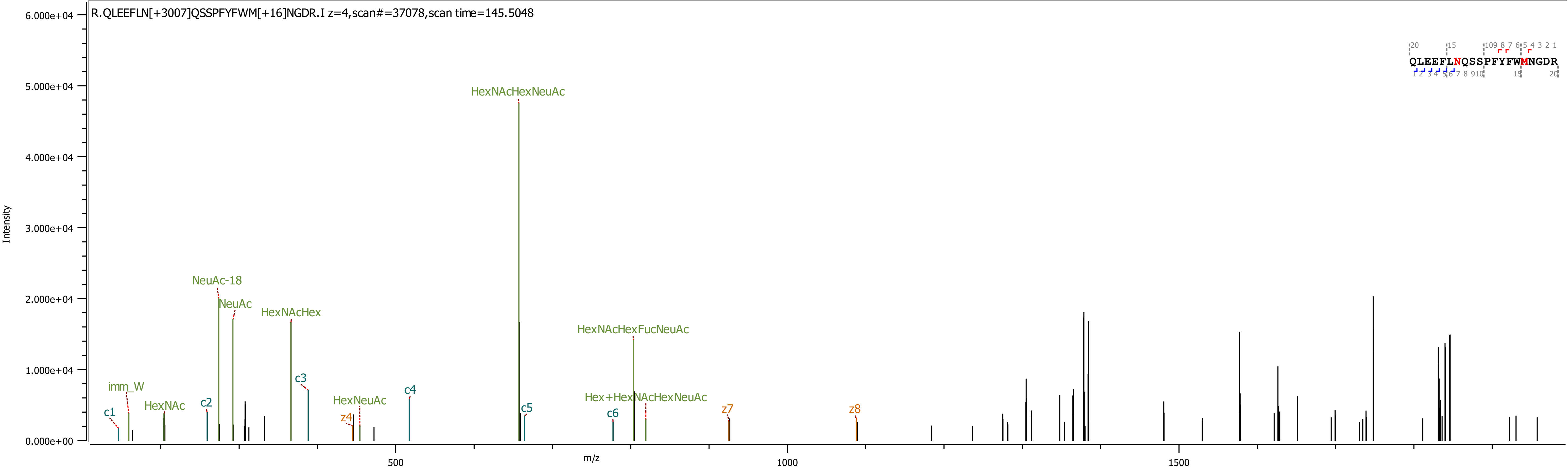
R.QLEEFLN[+3007]QSSPFYFWM[+16]NGDR.I z=4,scan#=37078,scan time=145.5048
Intensity
m/z
QLEEFLNQSSPFYFWMNGDR
HexNAcHexNeuAc
NeuAc-18
NeuAc
HexNAcHex
HexNAcHexFucNeuAc
Hex+HexNAcHexNeuAc
imm_W
HexNAc
HexNeuAc
c1
c2
c3
c4
c5
c6
z4
z7
z8
6.000e+04
5.000e+04
4.000e+04
3.000e+04
2.000e+04
1.000e+04
0.000e+00
500
1000
1500

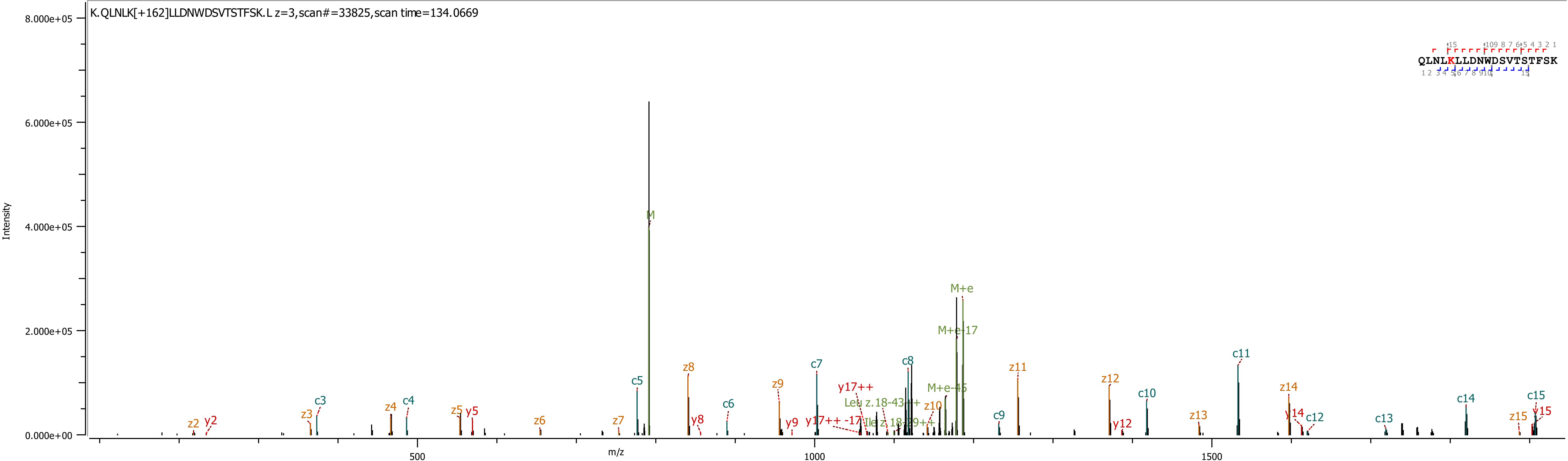
K.QLNLK[+162]LLDNWDSVTSTFSK.L z=3,scan#=33825,scan time=134.0669
Intensity
m/z
8.000e+05
6.000e+05
4.000e+05
2.000e+05
0.000e+00
500
1000
1500
QLNLKLLDNWDSVTSTFSK
M
M+e
M+e-17
M+e-45
c3
c4
c5
c6
c7
c8
c9
c10
c11
c12
c13
c14
c15
z2
z3
z4
z5
z6
z7
z8
z9
z10
z11
z12
z13
z14
z15
y2
y5
y8
y9
y12
y14
y15
y17++
y17++ -17
Leu z.18-43++
Ile z.18-29++

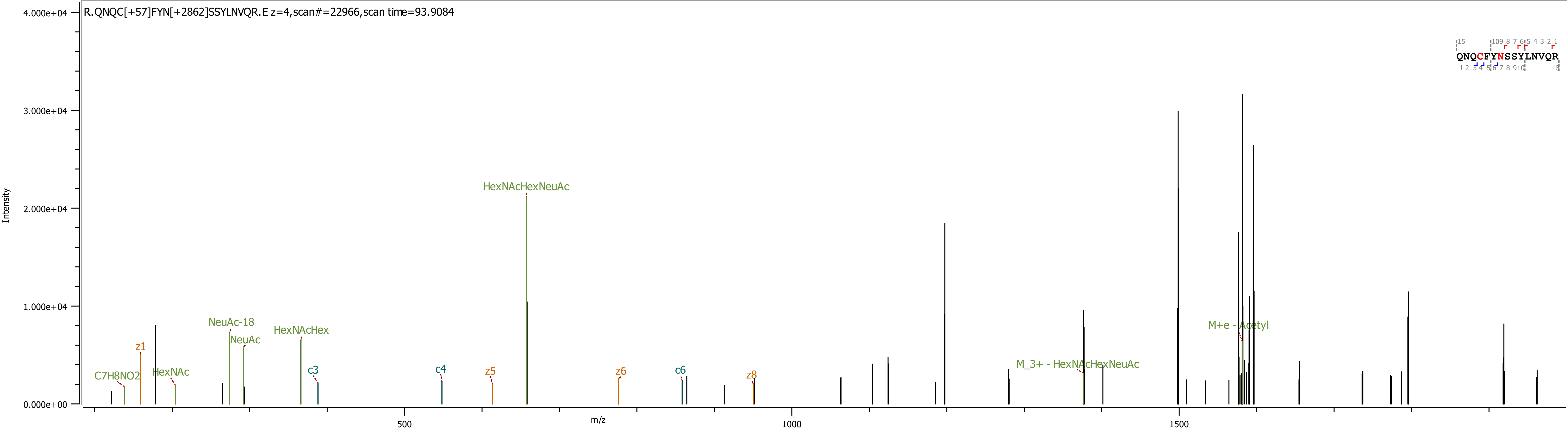
R.QNQC[+57]FYN[+2862]SSYLNVQR.E z=4,scan#=22966,scan time=93.9084
QNQCFYNSSYLNVQR
Intensity
m/z
C7H8NO2
z1
HexNAc
NeuAc-18
NeuAc
HexNAcHex
c3
c4
z5
HexNAcHexNeuAc
z6
c6
z8
M_3+ - HexNAcHexNeuAc
M+e - Acetyl

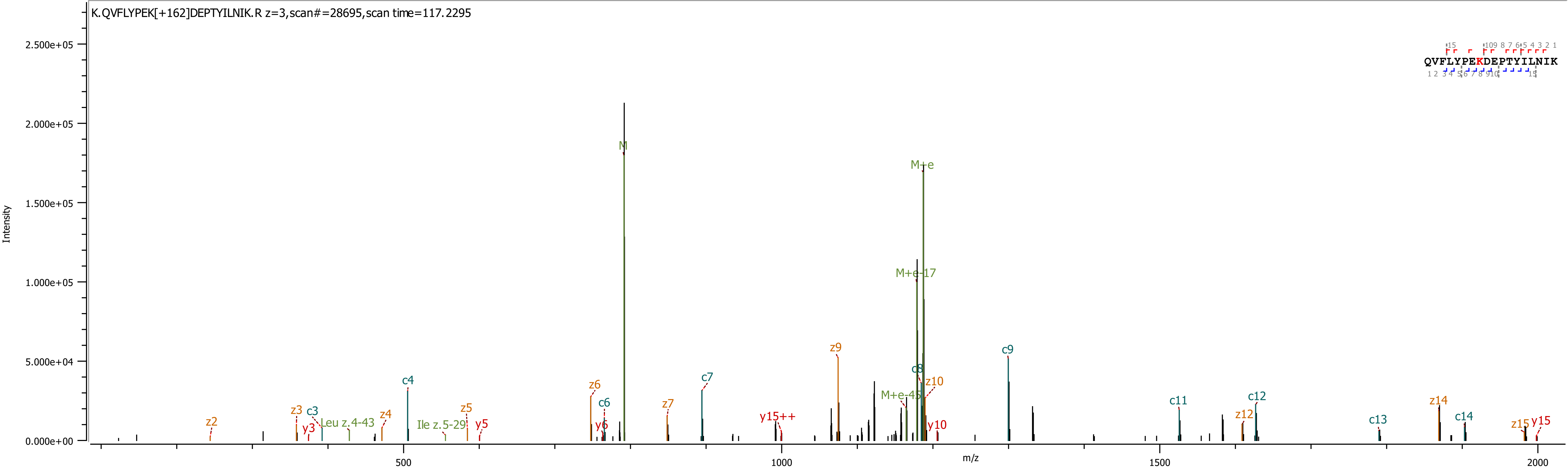
K.QVFLYPEK[+162]DEPTYILNIK.R z=3,scan#=28695,scan time=117.2295
Intensity
2.500e+05
2.000e+05
1.500e+05
1.000e+05
5.000e+04
0.000e+00
m/z
500
1000
1500
2000
QVFLYPEKDEPTYILNIK
M
M+e
M+e-17
M+e-45
z2
z3
c3
y3
Leu z.4-43
z4
c4
Ile z.5-29
z5
y5
z6
c6
y6
z7
c7
y15++
z9
c8
z10
y10
c9
c11
z12
c12
c13
z14
c14
z15
y15

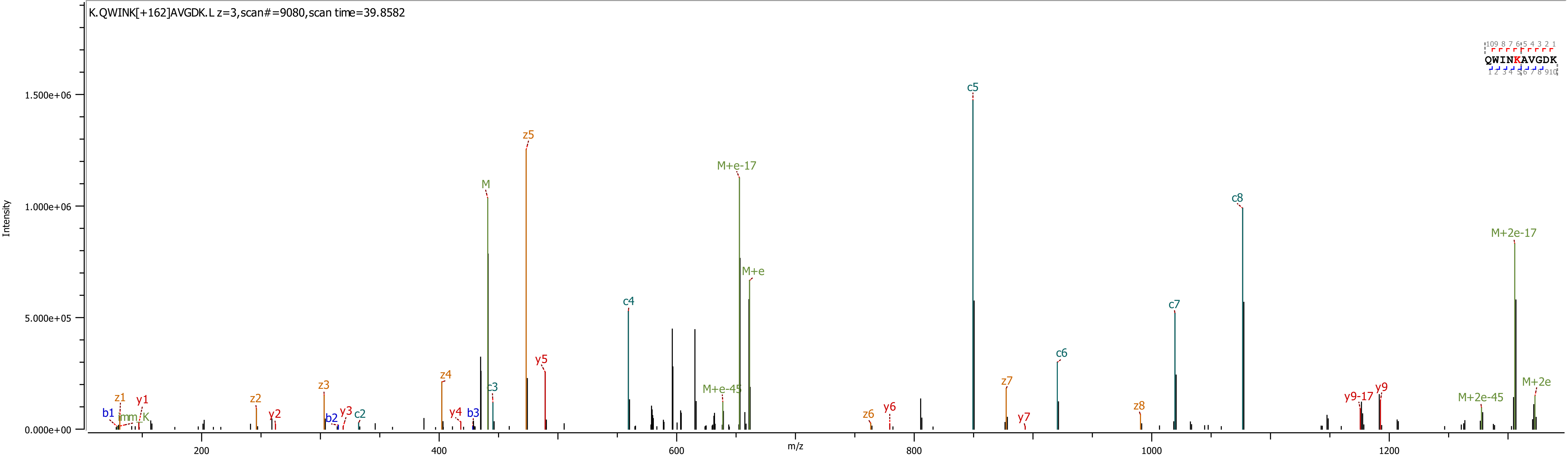

K.QWINK[+162]AVGDK.L z=3,scan#=9080,scan time=39.8582
QWINKAVGDK
Intensity
m/z
c5
z5
M+e-17
M
c8
M+2e-17
M+e
c4
c7
c6
y5
z4
c3
z7
M+e-45
y9
M+2e
z3
z1
y1
z2
y9-17
M+2e-45
z8
y6
b1
b2
y2
y3
c2
y4
b3
z6
y7

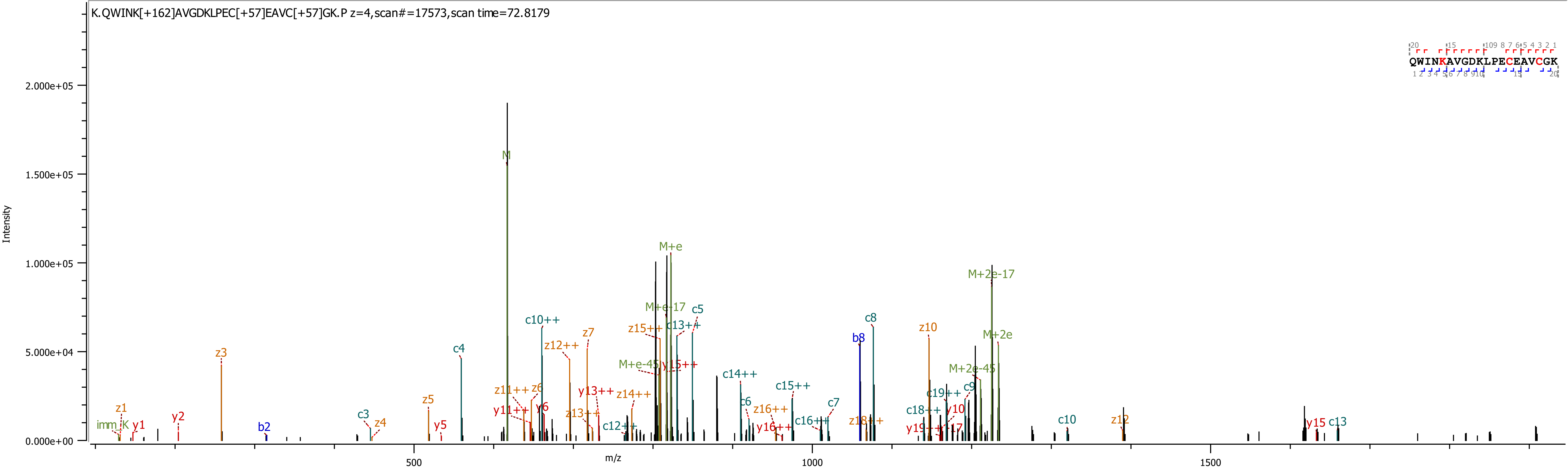
K.QWINK[+162]AVGDKLPEC[+57]EAVC[+57]GK.P z=4,scan#=17573,scan time=72.8179
QWINKAVGDKLPECEAVCGK
Intensity
2.000e+05
1.500e+05
1.000e+05
5.000e+04
0.000e+00
m/z
500
1000
1500
M
M+e
M+e-17
M+e-45
M+2e-17
M+2e
M+2e-45
imm_K
z1
y1
y2
z3
b2
c3
z4
z5
y5
c4
c10++
z11++
z6
y11++
y6
z12++
z7
y13++
z13++
z14++
c12++
z15++
y15++
c13++
c5
c14++
c6
z16++
y16++
c15++
c16++
c7
z18++
b8
c8
z10
c18++
c19++
y19++
y10
y17
c9
c10
z12
y15
c13

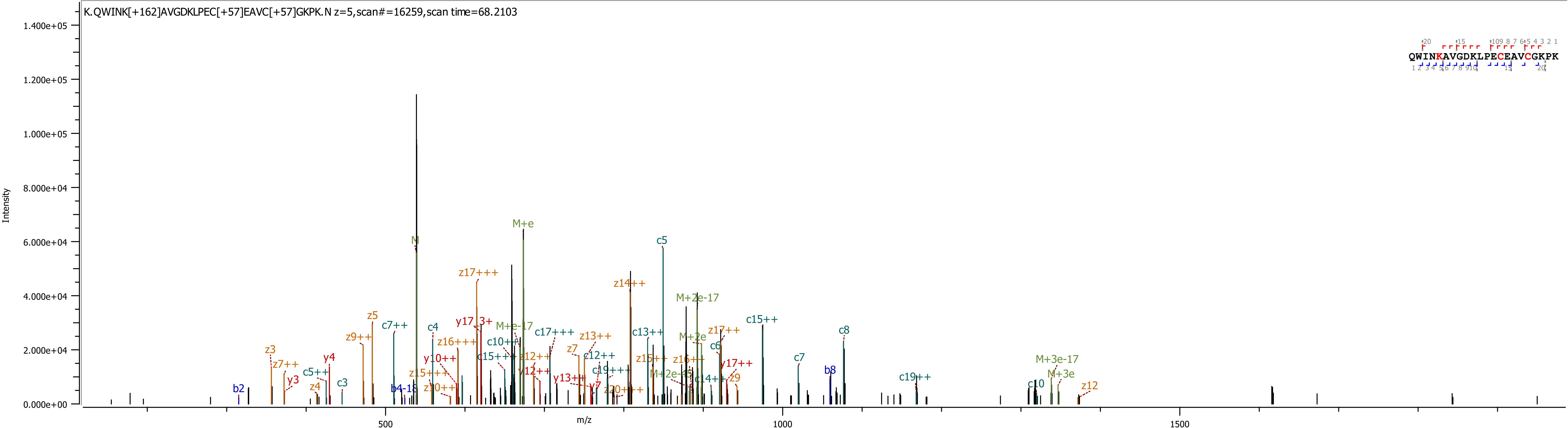
K.QWINK[+162]AVGDKLPEC[+57]EAVC[+57]GKPK.N z=5,scan#=16259,scan time=68.2103
QWINKAVGDKLPECEAVCGKPK
Intensity
m/z
1.400e+05
1.200e+05
1.000e+05
8.000e+04
6.000e+04
4.000e+04
2.000e+04
0.000e+00
500
1000
1500

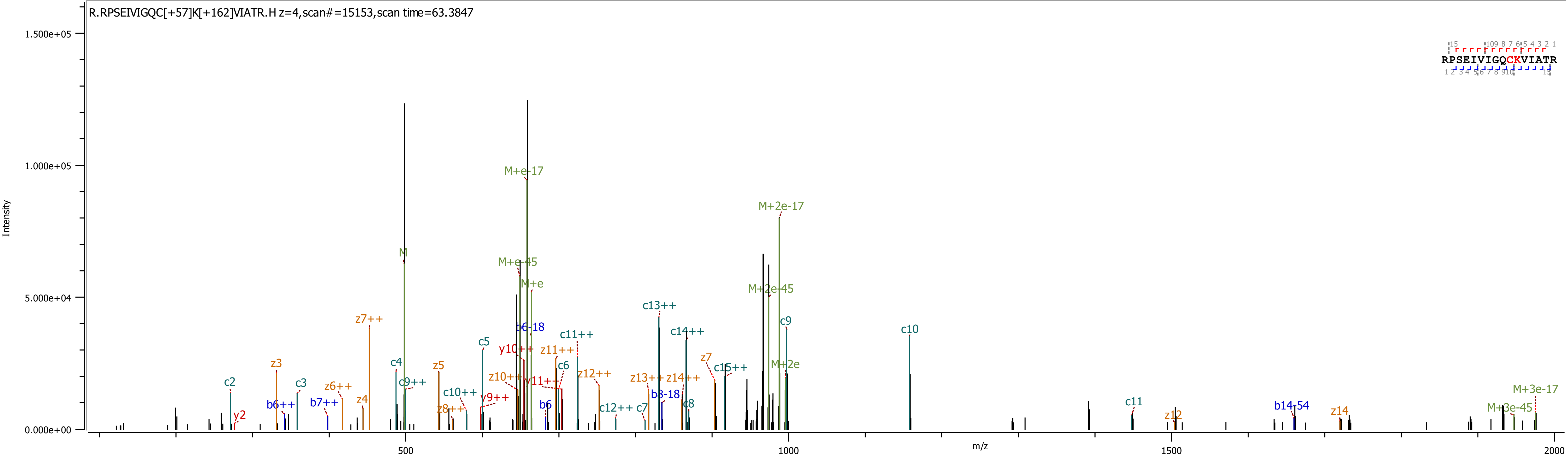

R.RPSEIVIGQC[+57]K[+162]VIATR.H z=4,scan#=15153,scan time=63.3847
RPSEIVIGQCKVIATR
Intensity
m/z
1.500e+05
1.000e+05
5.000e+04
0.000e+00
500
1000
1500
2000
M+e-17
M+2e-17
M
M+e-45
M+e
M+2e-45
c13++
c9
c10
c14++
b6-18
c11++
z7++
c5
y10++
z11++
z7
c15++
M+2e
z3
c4
z5
c6
c2
c3
z6++
c9++
c10++
z10++
y11++
z12++
z13++
z14++
b8-18
M+3e-17
z4
y9++
b6++
b7++
z8++
c12++
c7
c8
b6
c11
b14-54
M+3e-45
y2
z12
z14

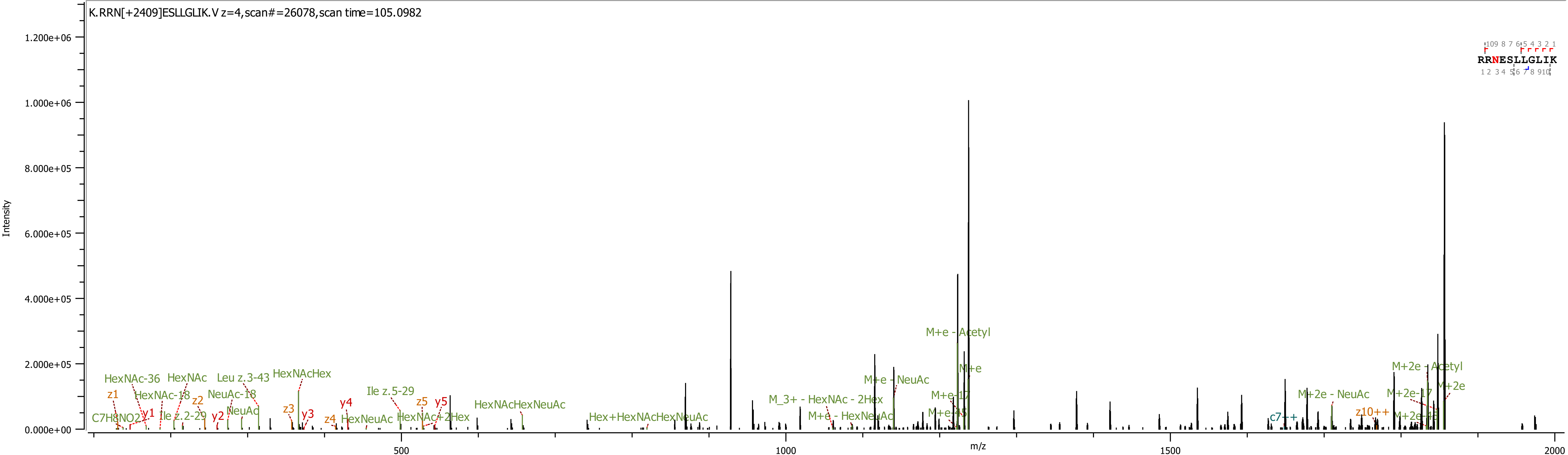
K.RRN[+2409]ESLLGLIK.V z=4,scan#=26078,scan time=105.0982
Intensity
m/z
RRNESLLGLIK
M+e - Acetyl
M+e
M+e - NeuAc
M+e-17
M+e - HexNeuAc
M_3+ - HexNAc - 2Hex
M+2e - Acetyl
M+2e
M+2e - NeuAc
M+2e-17
z10++
c7++
HexNAc-36
HexNAc
HexNAc-18
Leu z.3-43
HexNAcHex
NeuAc-18
NeuAc
Ile z.2-29
Ile z.5-29
C7H8NO2
HexNeuAc
HexNAc+2Hex
HexNAcHexNeuAc
Hex+HexNAcHexNeuAc
z1
y1
z2
y2
z3
y3
z4
y4
z5
y5

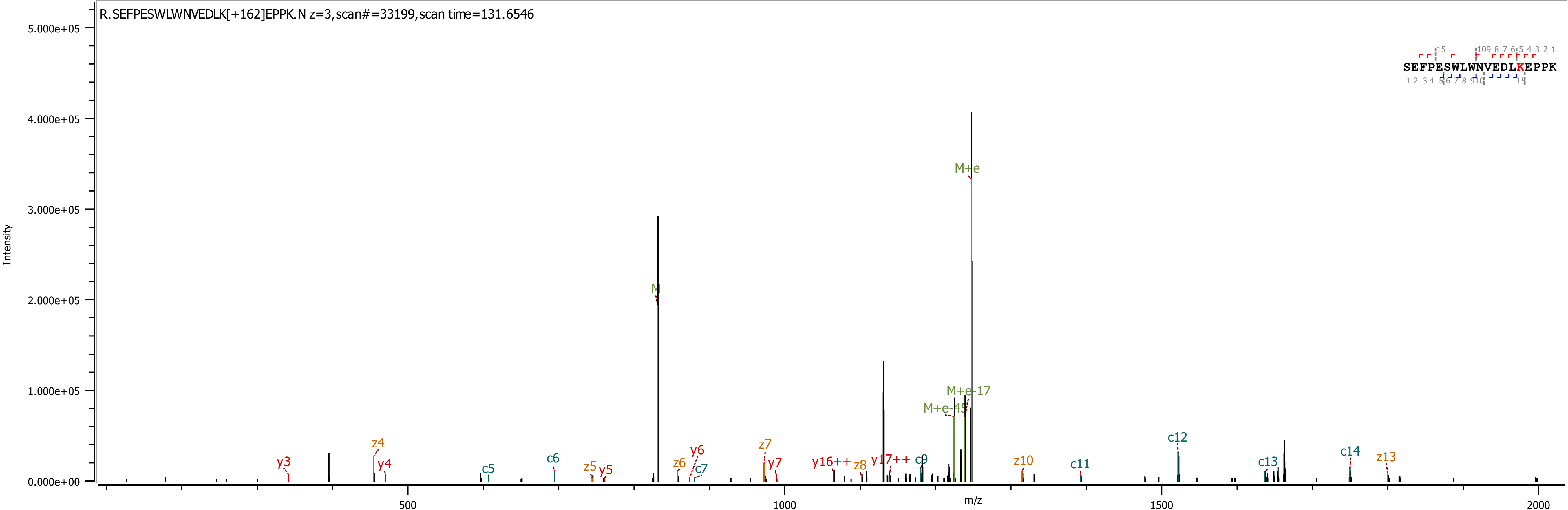
R.SEFPESWLWNVEDLK[+162]EPPK.N z=3,scan#=33199,scan time=131.6546
SEFPESWLWNVEDLKEPPK
Intensity
m/z
M+e
M
M+e-17
M+e-45
y3
z4
y4
c5
c6
z5
y5
z6
y6
c7
z7
y7
y16++
z8
y17++
c9
z10
c11
c12
c13
c14
z13

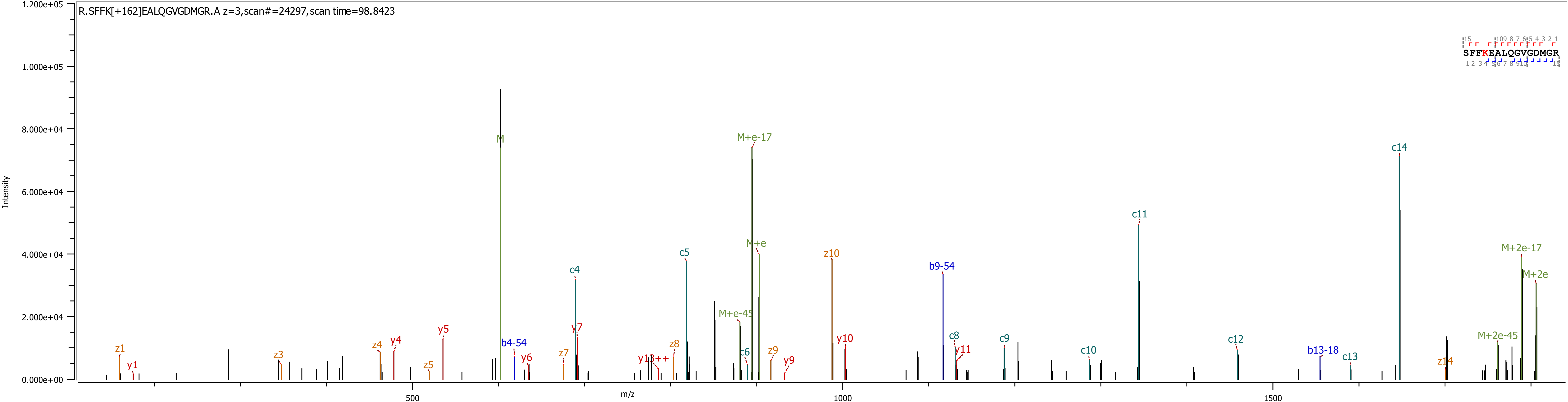
R.SFFK[+162]EALQGVGDMGR.A z=3,scan#=24297,scan time=98.8423
Intensity
m/z
SFFKEALQGVGDMGR
M
M+e-17
M+e
M+e-45
M+2e-17
M+2e
M+2e-45
c4
c5
c6
c8
c9
c10
c11
c12
c13
c14
z1
z3
z4
z5
z7
z8
z9
z10
z14
y1
y4
y5
y6
y7
y9
y10
y11
y13++
b4-54
b9-54
b13-18

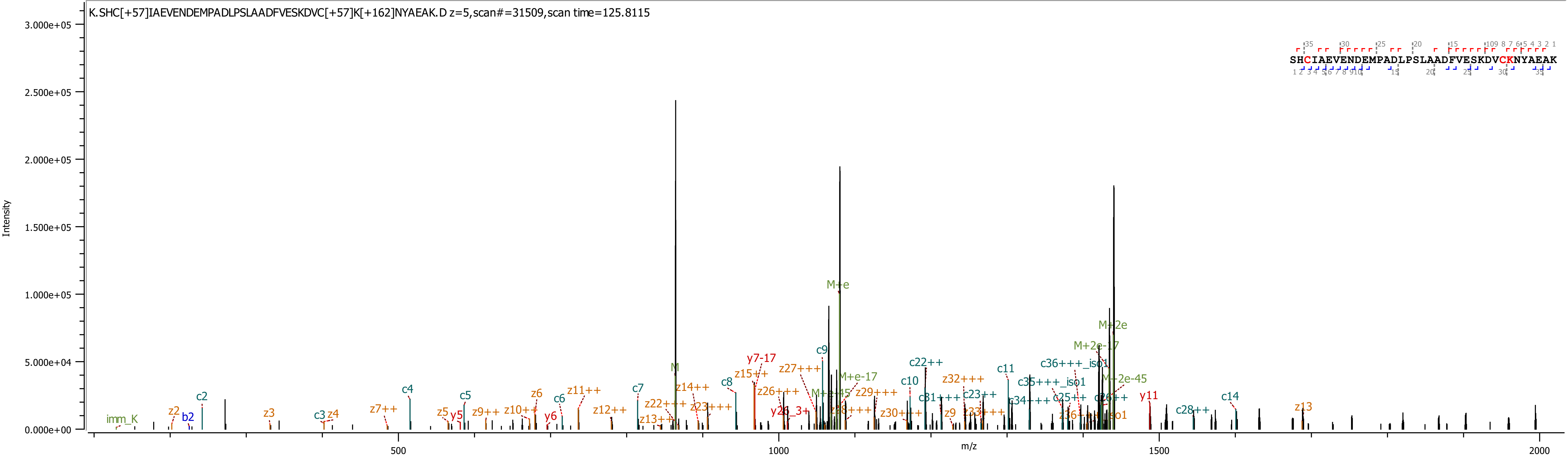
K.SHC[+57]IAEVENDEMPADLPSLAADFVESKDVC[+57]K[+162]NYAEAK.D z=5,scan#=31509,scan time=125.8115
SHCIAEVENDEMPADLPSLAADFVESKDVCKNYAEAK
Intensity
m/z
3.000e+05
2.500e+05
2.000e+05
1.500e+05
1.000e+05
5.000e+04
0.000e+00
500
1000
1500
2000

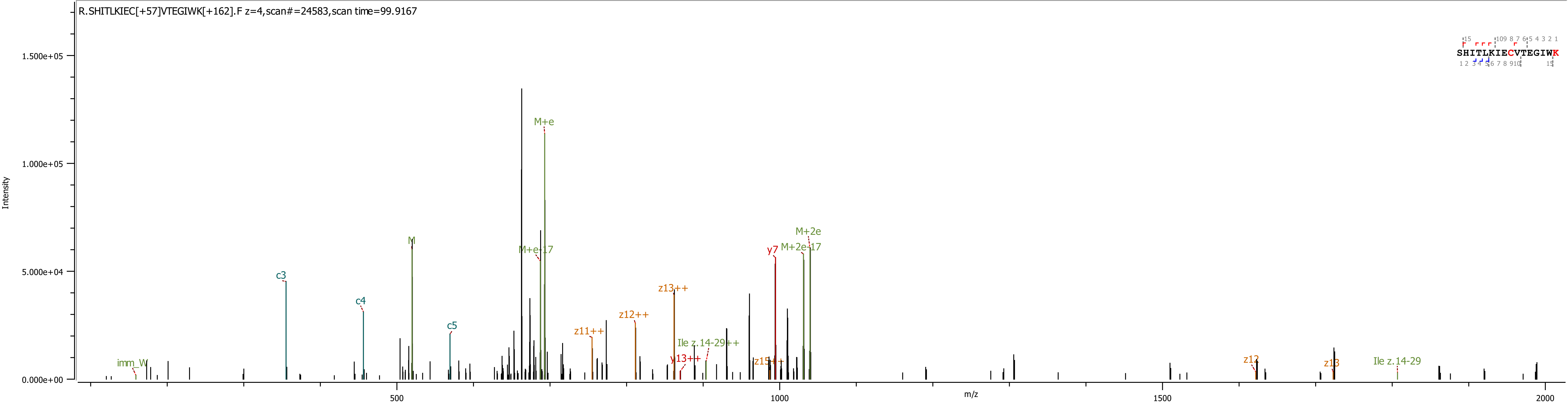
R.SHITLKIEC[+57]VTEGIWK[+162].F z=4,scan#=24583,scan time=99.9167
SHITLKIECVTEGIWK
Intensity
1.500e+05
1.000e+05
5.000e+04
0.000e+00
m/z
500
1000
1500
2000
M+e
M
M+e-17
M+2e
M+2e-17
y7
c3
c4
c5
z13++
z12++
z11++
Ile z.14-29++
y13++
z15++
z12
z13
Ile z.14-29
imm_W

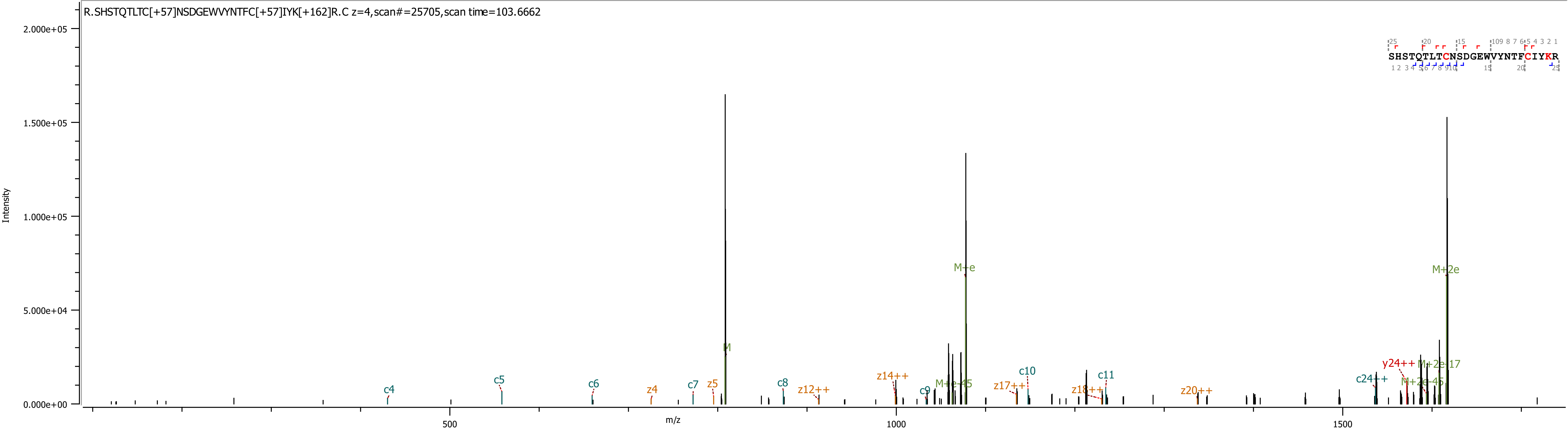

R.SHSTQTLTC[+57]NSDGEWVYNTFC[+57]IYK[+162]R.C z=4,scan#=25705,scan time=103.6662
Intensity
m/z
SHSTQTLTCNSDGEWVYNTFCIYKR
M
M+e
M+2e
M+2e-17
M+2e-45
M+e-45
c4
c5
c6
c7
c8
c9
c10
c11
c24++
z4
z5
z12++
z14++
z17++
z18++
z20++
y24++

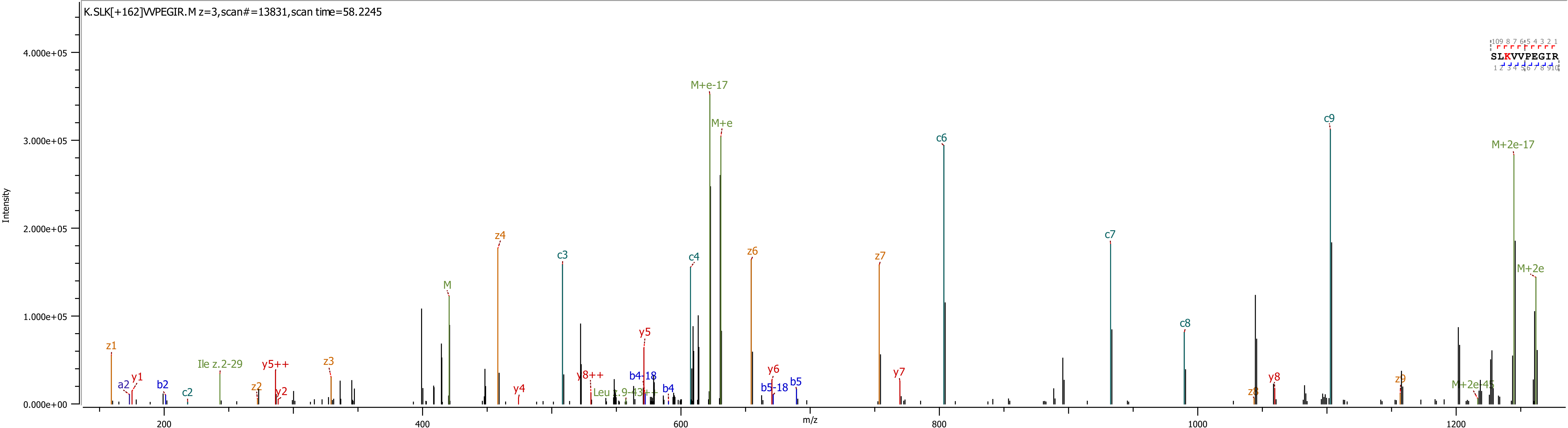
K.SLK[+162]VVPEGIR.M z=3,scan#=13831,scan time=58.2245
Intensity
m/z
4.000e+05
3.000e+05
2.000e+05
1.000e+05
0.000e+00
200
400
600
800
1000
1200
SLKVVPEGIR
M+e-17
M+e
c9
c6
M+2e-17
M+2e
c7
c3
c4
z4
z6
z7
M
c8
y5
z1
a2
y1
b2
c2
Ile z.2-29
y5++
z2
y2
z3
y4
y8++
Leu z.9-43++
b4-18
b4
b5-18
b5
y6
y7
z8
y8
z9
M+2e-45

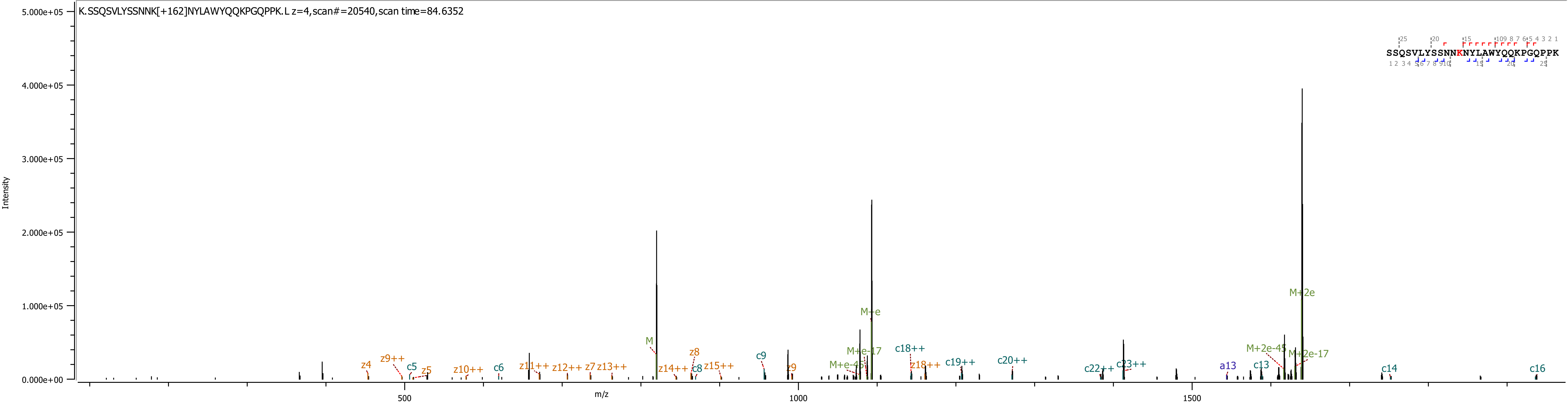
K.SSQSVLYSSNNK[+162]NYLAWYQQKPGQPPK.L z=4,scan#=20540,scan time=84.6352
SSQSVLYSSNNKNYLAWYQQKPGQPPK
Intensity
m/z
5.000e+05
4.000e+05
3.000e+05
2.000e+05
1.000e+05
0.000e+00
500
1000
1500
z4
z9++
c5
z5
z10++
c6
z11++
z12++
z7
z13++
M
z14++
z8
c8
z15++
c9
z9
M+e-45
M+e-17
M+e
c18++
z18++
c19++
c20++
c22++
c23++
a13
c13
M+2e-45
M+2e
M+2e-17
c14
c16

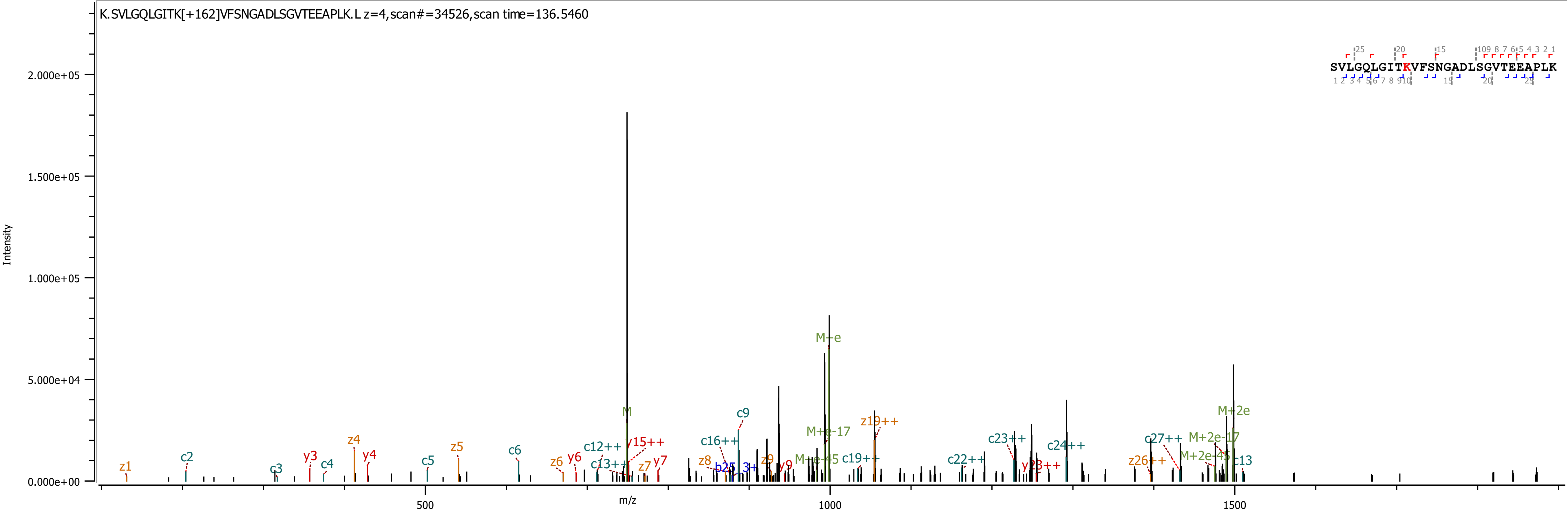
K.SVLGQLGITK[+162]VFSNGADLSGVTEEAPLK.L z=4,scan#=34526,scan time=136.5460
SVLGQLGITKVFSNGADLSGVTEEAPLK
Intensity
2.000e+05
1.500e+05
1.000e+05
5.000e+04
0.000e+00
m/z
500
1000
1500
z1
c2
c3
y3
c4
z4
y4
c5
z5
c6
z6
y6
c12++
c13++
M
y15++
z7
y7
z8
c16++
c9
z9
y9
M+e-45
M+e
M+e-17
c19++
z19++
c22++
c23++
y23++
c24++
z26++
c27++
M+2e-17
M+2e-45
M+2e
c13

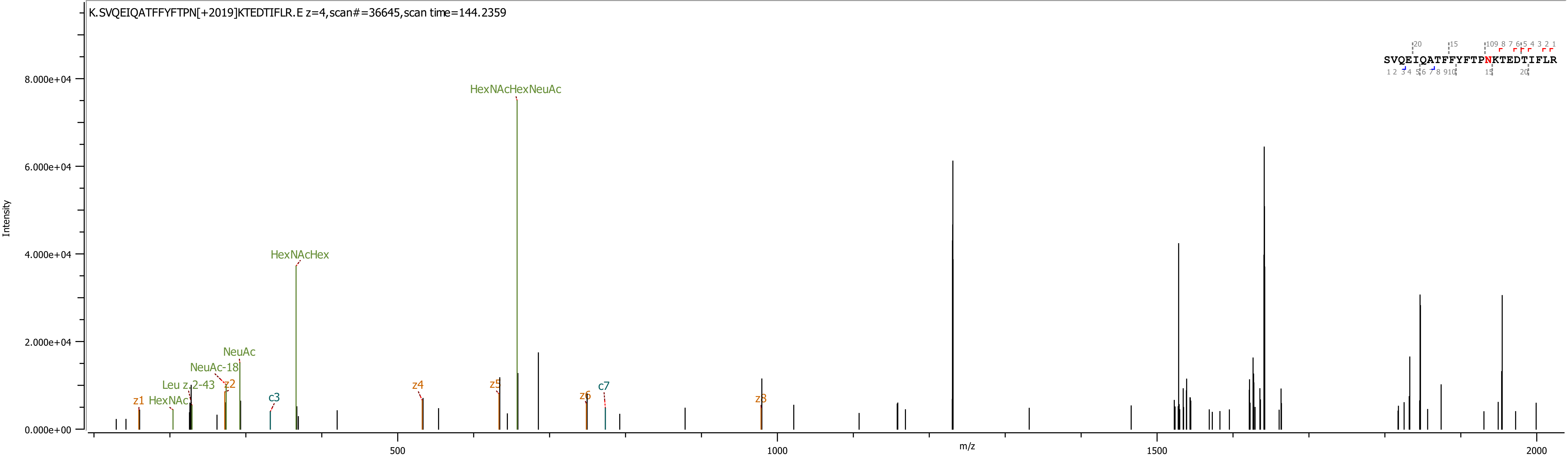
K.SVQEIQATFFYFTPN[+2019]KTEDTIFLR.E z=4,scan#=36645,scan time=144.2359
SVQEIQATFFYFTPNKTEDTIFLR
Intensity
m/z
8.000e+04
6.000e+04
4.000e+04
2.000e+04
0.000e+00
500
1000
1500
2000
HexNAcHexNeuAc
HexNAcHex
NeuAc
NeuAc-18
Leu z 2-43
z1
HexNAc
z2
c3
z4
z5
z6
c7
z8

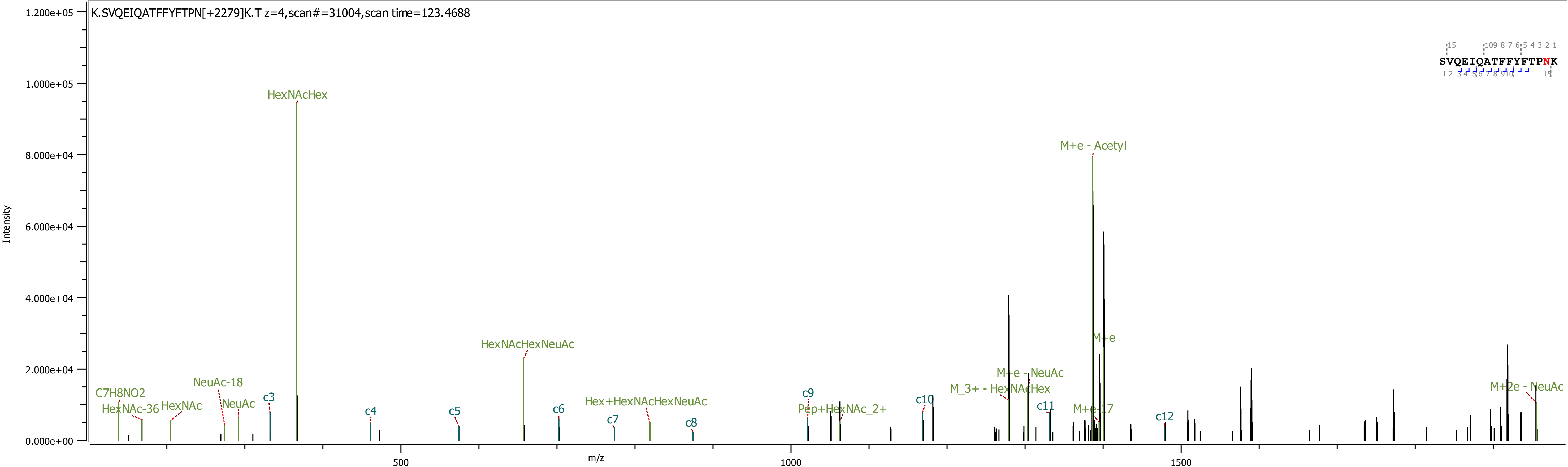

K.SVQEIQATFFYFTPN[+2279]K.T z=4,scan#=31004,scan time=123.4688
Intensity
m/z
1.200e+05
1.000e+05
8.000e+04
6.000e+04
4.000e+04
2.000e+04
0.000e+00
500
1000
1500
HexNAcHex
M+e - Acetyl
HexNAcHexNeuAc
M+e
M+e - NeuAc
M_3+ - HexNAcHex
M+2e - NeuAc
C7H8NO2
HexNAc-36
HexNAc
NeuAc-18
NeuAc
Hex+HexNAcHexNeuAc
Pep+HexNAc_2+
M+e-17
c3
c4
c5
c6
c7
c8
c9
c10
c11
c12
SVQEIQATFFYFTPNK

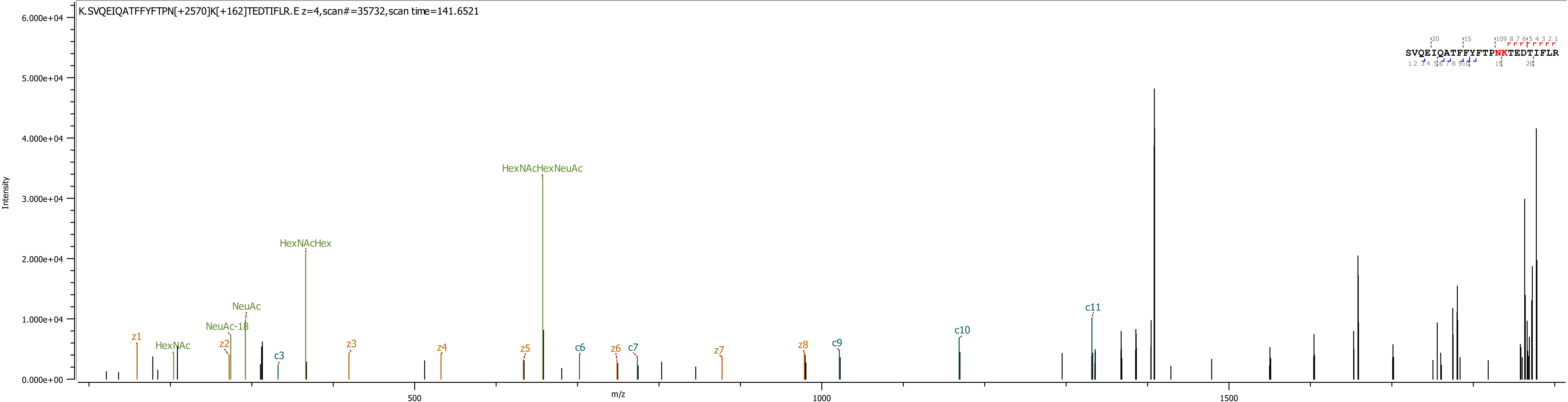
K.SVQEIQATFFYFTPN[+2570]K[+162]TEDTIFLR.E z=4,scan#=35732,scan time=141.6521
Intensity
m/z
SVQEIQATFFYFTPNKTEDTIFLR
z1
HexNAc
z2
NeuAc-18
NeuAc
c3
HexNAcHex
z3
z4
z5
HexNAcHexNeuAc
c6
z6
c7
z7
z8
c9
c10
c11
500
1000
1500

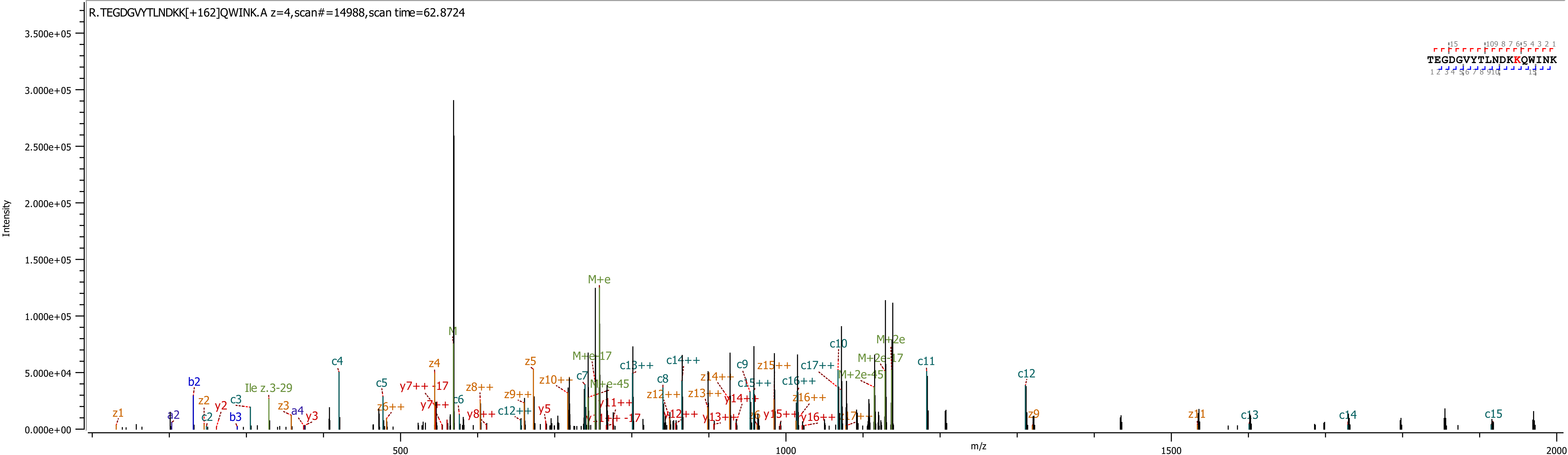
R.TEGDGVYTLNDKK[+162]QWINK.A z=4,scan#=14988,scan time=62.8724
TEGDGVYTLNDKKQWINK
Intensity
m/z
M+e
M
M+e-17
M+e-45
M+2e
M+2e-17
M+2e-45
Ile z.3-29
c4
c5
c6
c7
c8
c9
c10
c11
c12
c13
c14
c15
c12++
c13++
c14++
c15++
c16++
c17++
z1
z2
z3
z4
z5
z9
z11
z6++
z8++
z9++
z10++
z12++
z13++
z14++
z15++
z16++
y2
y3
y5
y7++
y7++ -17
y8++
y11++
y11++ -17
y12++
y13++
y14++
y15++
y16++
a2
a4
b2
b3
c2
c3

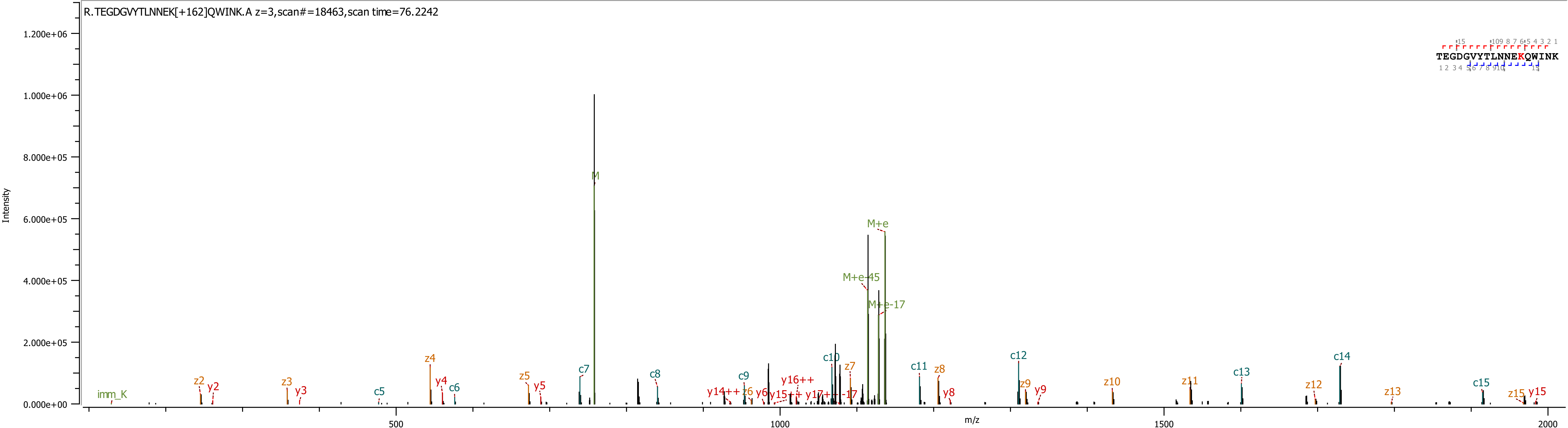

R.TEGDGVYTLNNEK[+162]QWINK.A z=3,scan#=18463,scan time=76.2242
TEGDGVYTLNNEKQWINK
Intensity
m/z
1.200e+06
1.000e+06
8.000e+05
6.000e+05
4.000e+05
2.000e+05
0.000e+00
500
1000
1500
2000
M
M+e
M+e-45
M+e-17
imm_K
z2
y2
z3
y3
c5
z4
y4
c6
z5
y5
c7
c8
c9
z6
y6
y14++
y15++
y16++
c10
z7
c11
z8
y8
c12
z9
y9
z10
z11
c13
z12
z13
c14
c15
z15
y15

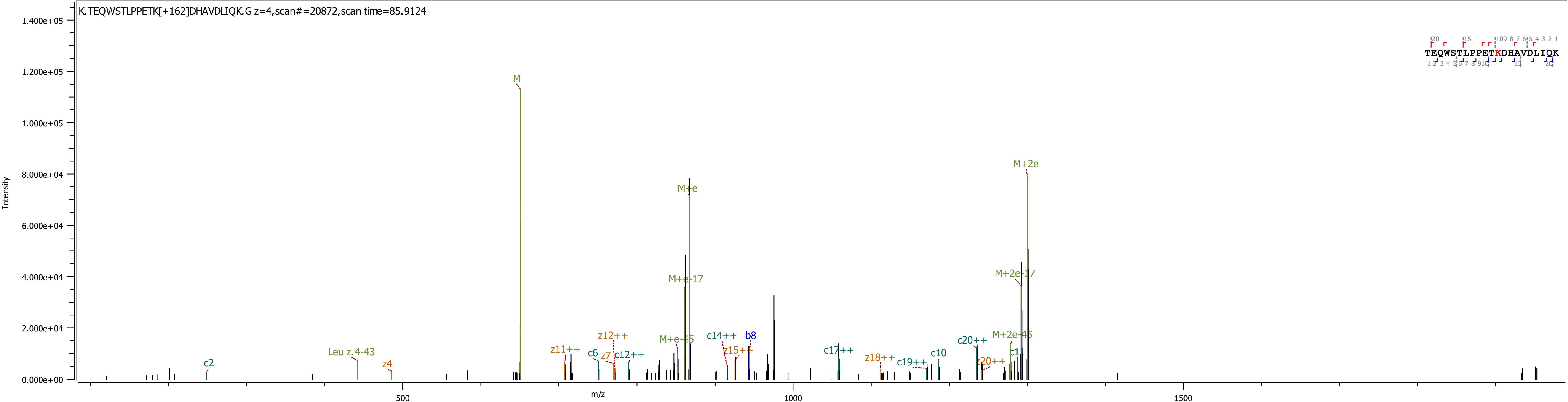
K.TEQWSTLPPETK[+162]DHAVDLIQK.G z=4,scan#=20872,scan time=85.9124
Intensity
m/z
M
M+e
M+e-17
M+e-45
M+2e
M+2e-17
M+2e-45
Leu z.4-43
c2
z4
z11++
c6
z7
z12++
c12++
c14++
b8
z15++
c17++
z18++
c19++
c10
c20++
z20++
c11
TEQWSTLPPETKDHAVDLIQK

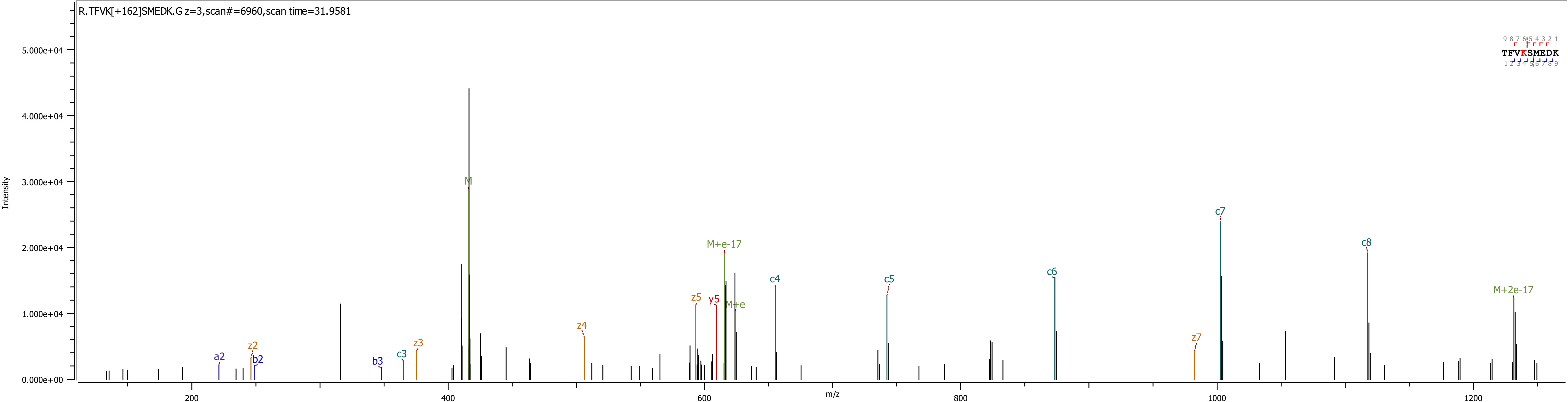
R.TFVK[+162]SMEDK.G z=3,scan#=6960,scan time=31.9581
TFVKSMEDK
Intensity
m/z
M
M+e-17
M+e
M+2e-17
a2
z2
b2
b3
c3
z3
z4
z5
y5
c4
c5
c6
z7
c7
c8

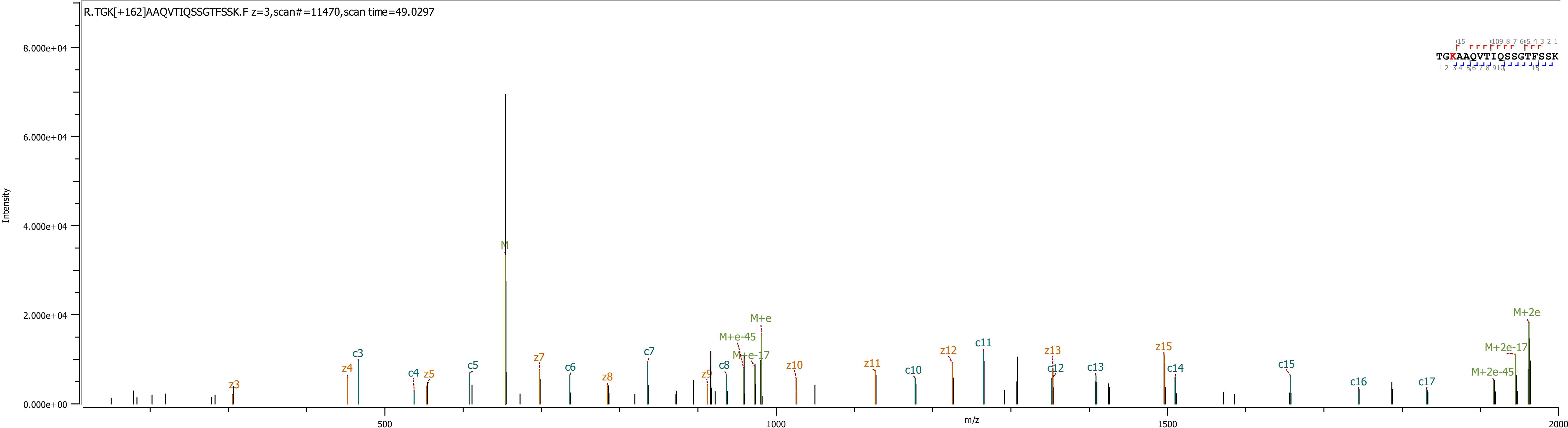
R.TGK[+162]AAQVTIQSSGTFSSK.F z=3,scan#=11470,scan time=49.0297
TGKAAQVTIQSSGTFSSK
Intensity
m/z
8.000e+04
6.000e+04
4.000e+04
2.000e+04
0.000e+00
500
1000
1500
2000
M
M+e
M+e-45
M+e-17
M+2e
M+2e-17
M+2e-45
c3
c4
c5
c6
c7
c8
c10
c11
c12
c13
c14
c15
c16
c17
z3
z4
z5
z7
z8
z9
z10
z11
z12
z13
z15

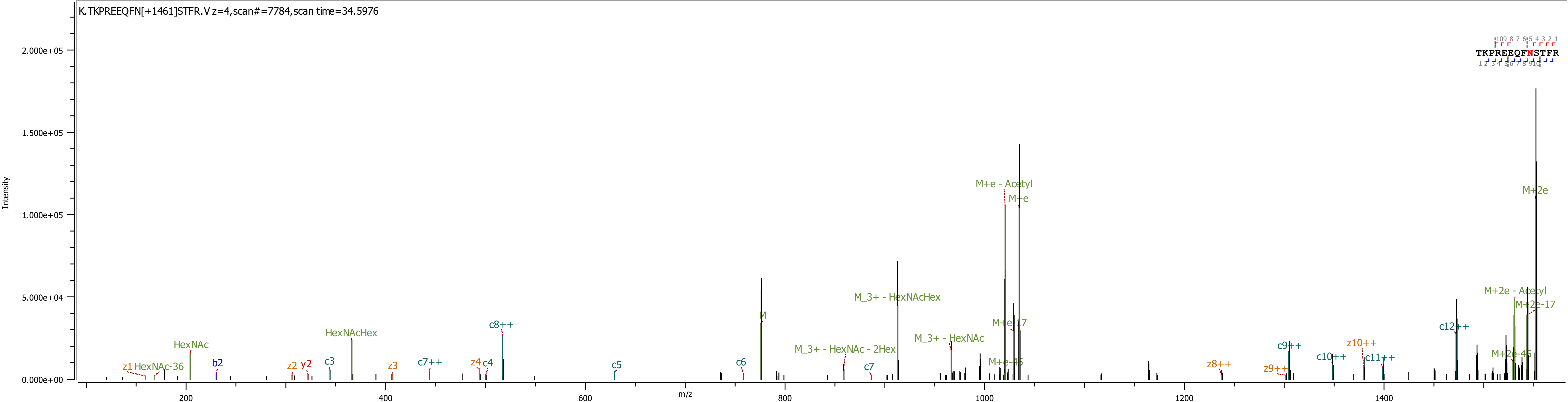
K.TKPREEQFN[+1461]STFR.V z=4,scan#=7784,scan time=34.5976
TKPREEQFNSTFR
Intensity
2.000e+05
1.500e+05
1.000e+05
5.000e+04
0.000e+00
m/z
200
400
600
800
1000
1200
1400
z1
HexNAc-36
HexNAc
b2
z2
y2
c3
HexNAcHex
z3
c7++
z4
c4
c8++
c5
c6
M
M_3+ - HexNAc - 2Hex
c7
M_3+ - HexNAcHex
M_3+ - HexNAc
M+e - Acetyl
M+e
M+e-17
M+e-45
z8++
z9++
c9++
c10++
z10++
c11++
c12++
M+2e
M+2e - Acetyl
M+2e-17
M+2e-45

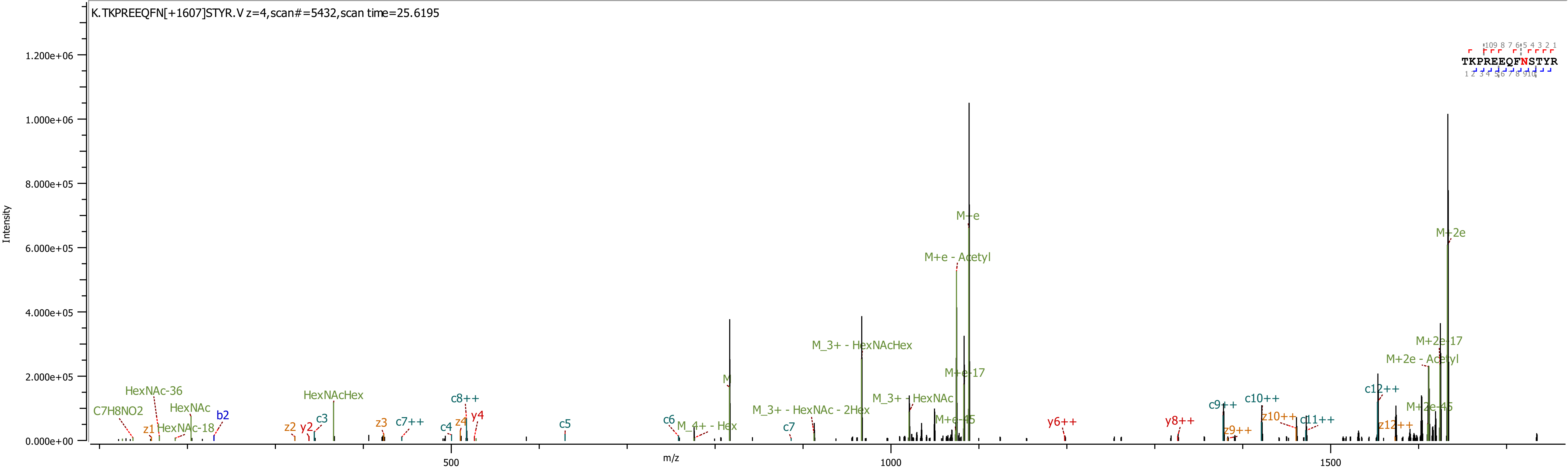

K.TKPREEQFN[+1607]STYR.V z=4,scan#=5432,scan time=25.6195
TKPREEQFNSTYR
Intensity
m/z
1.200e+06
1.000e+06
8.000e+05
6.000e+05
4.000e+05
2.000e+05
0.000e+00
500
1000
1500
M+e
M+e - Acetyl
M+2e
M_3+ - HexNAcHex
M+2e-17
M+2e - Acetyl
M+e-17
M
HexNAc-36
HexNAcHex
c8++
c12++
M_3+ - HexNAc
c9++
c10++
C7H8NO2
HexNAc
b2
y4
M_3+ - HexNAc - 2Hex
M+2e-45
z3
c7++
c3
c6
M_4+ - Hex
M+e-45
y6++
y8++
z10++
c11++
z1
HexNAc-18
z2
y2
c4
z4
c5
c7
z9++
z12++

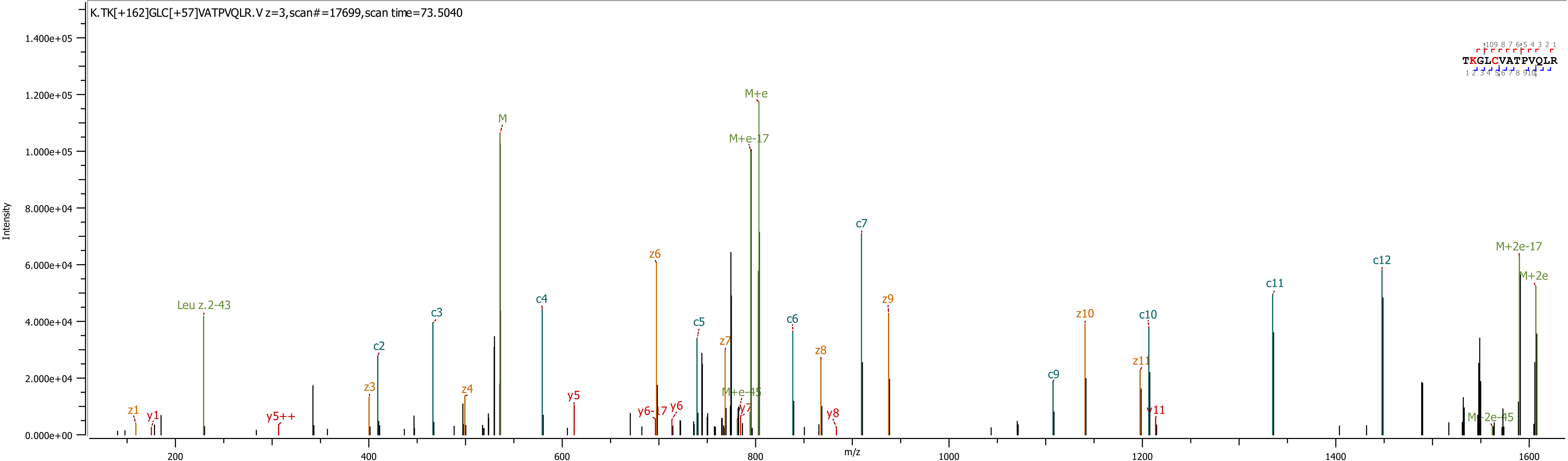

K.TK[+162]GLC[+57]VATPVQLR.V z=3,scan#=17699,scan time=73.5040
TKGLCVATPVQLR
Intensity
m/z
M+e
M
M+e-17
c7
z6
M+2e-17
c12
M+2e
c11
z9
c4
c3
z10
c10
c5
c6
Leu z.2-43
c2
z7
z8
z11
c9
z3
z4
y5
M+e-45
y6
y6-17
y7
y11
y8
z1
y1
y5++
M+2e-45

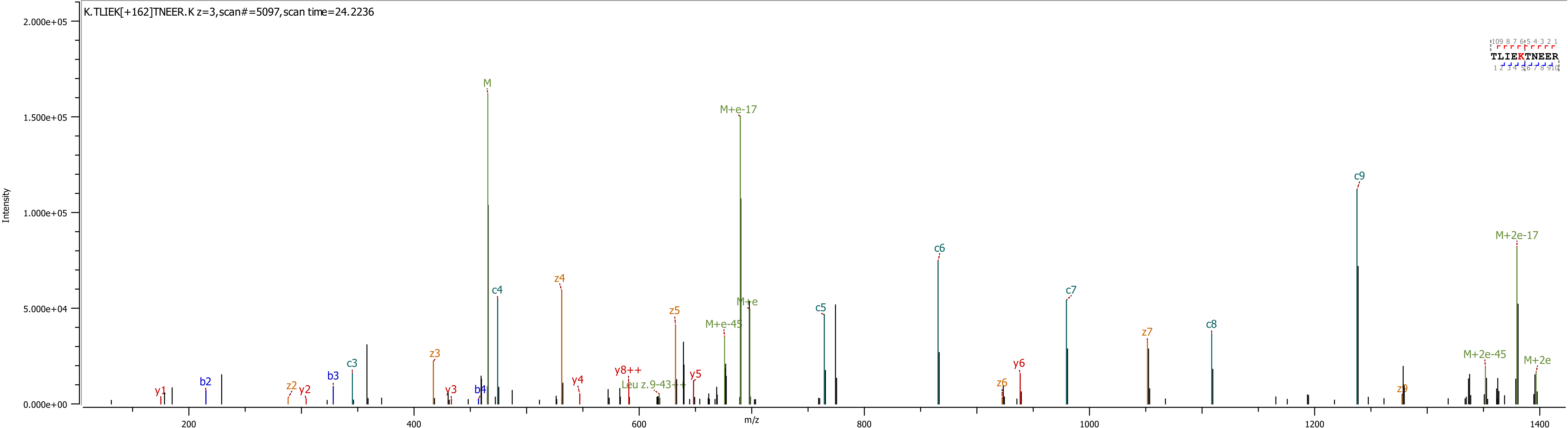

K.TLIEK[+162]TNEER.K z=3,scan#=5097,scan time=24.2236
Intensity
m/z
2.000e+05
1.500e+05
1.000e+05
5.000e+04
0.000e+00
200
400
600
800
1000
1200
1400
TLIEKTNEER
M
M+e-17
c9
M+2e-17
c6
c4
z4
M+e
c7
c5
z5
M+e-45
c8
z7
z3
M+2e-45
M+2e
c3
b3
b2
y8++
y5
y6
y4
z2
y2
y1
y3
b4
z6
z9
Leu z.9-43++

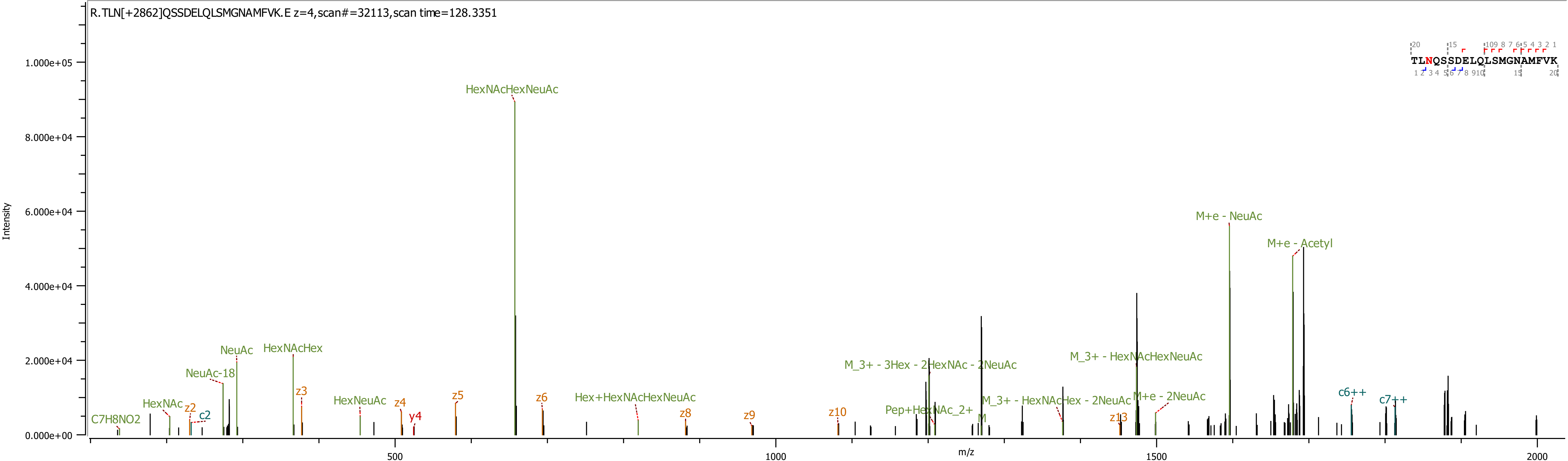

R.TLN[+2862]QSSDELQLSMGNAMFVK.E z=4,scan#=32113,scan time=128.3351
TLNQSSDELQLSMGNAMFVK
Intensity
m/z
1.000e+05
8.000e+04
6.000e+04
4.000e+04
2.000e+04
0.000e+00
500
1000
1500
2000
HexNAcHexNeuAc
M+e - NeuAc
M+e - Acetyl
M_3+ - HexNAcHexNeuAc
M_3+ - 3Hex - 2HexNAc - 2NeuAc
NeuAc
HexNAcHex
NeuAc-18
z3
HexNAc
z2
c2
C7H8NO2
HexNeuAc
z4
y4
z5
z6
Hex+HexNAcHexNeuAc
z8
z9
z10
Pep+HexNAc_2+
M
M_3+ - HexNAcHex - 2NeuAc
M+e - 2NeuAc
z13
c6++
c7++

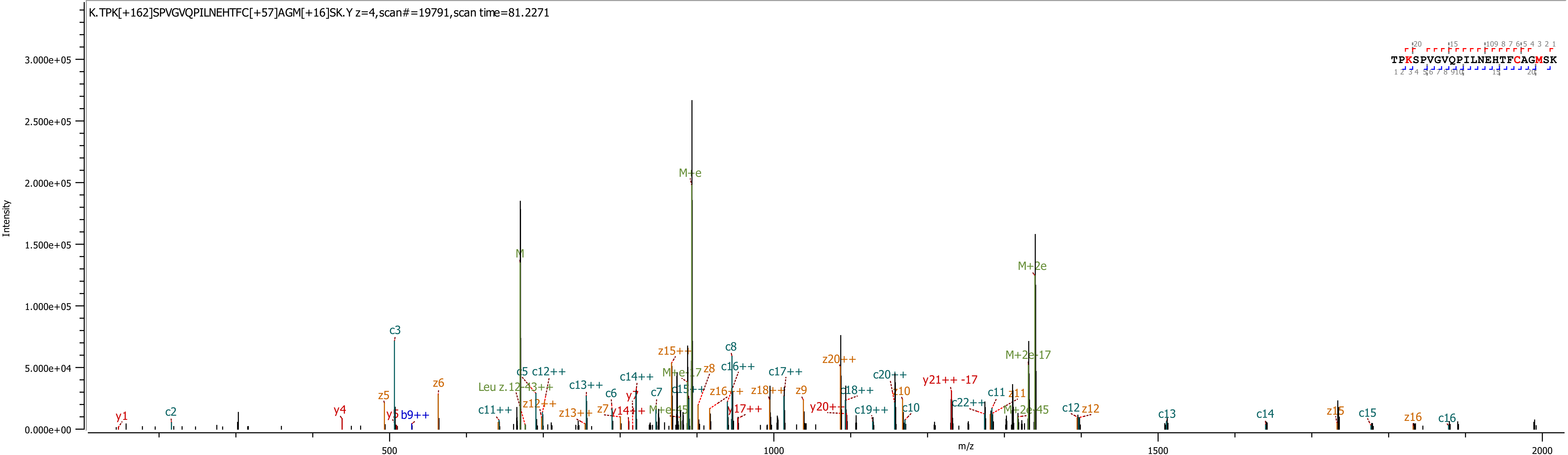

K.TPK[+162]SPVGVQPILNEHTFC[+57]AGM[+16]SK.Y z=4,scan#=19791,scan time=81.2271
Intensity
m/z
TPKSPVGVQPILNEHTFCAGMSK
M+e
M
M+2e
c3
M+2e-17
c8
z15++
z20++
y21++ -17

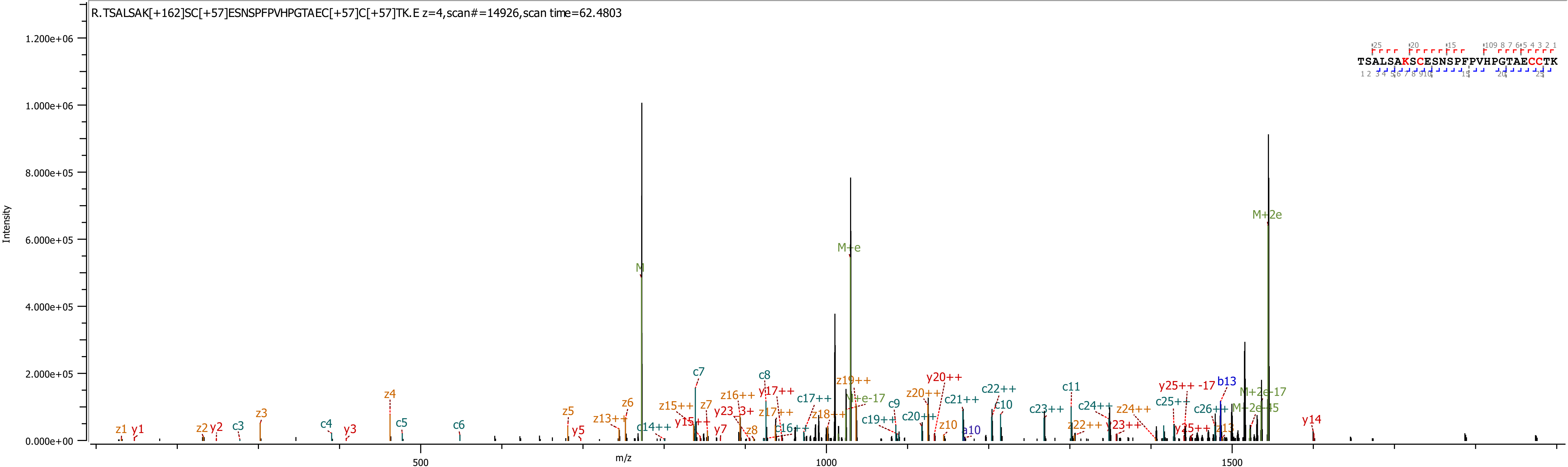
R.TSALSAK[+162]SC[+57]ESNSPFPVHPGTAEC[+57]C[+57]TK.E z=4,scan#=14926,scan time=62.4803
TSALSAKSCESNSPFPVHPGTAECCTK
Intensity
m/z
M
M+e
M+2e
M+e-17
M+2e-17
M+2e-45
b13
y14

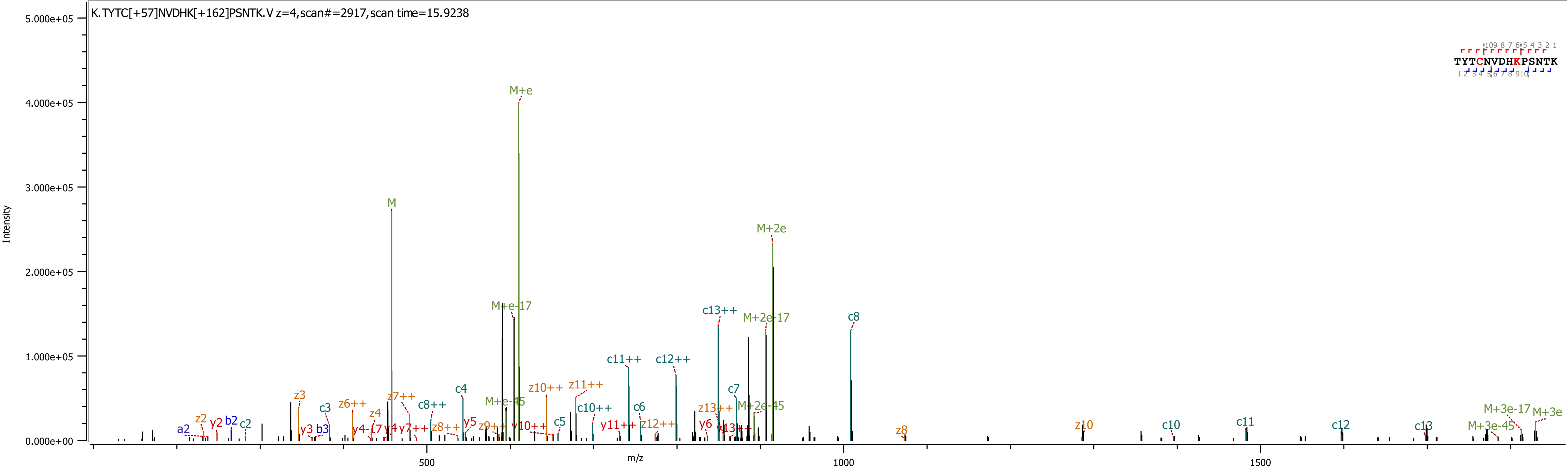
K.TYTC[+57]NVDHK[+162]PSNTK.V z=4,scan#=2917,scan time=15.9238
Intensity
m/z
5.000e+05
4.000e+05
3.000e+05
2.000e+05
1.000e+05
0.000e+00
500
1000
1500
TYTCNVDHKPSNTK
M+e
M
M+2e
M+e-17
c13++
M+2e-17
c8
c11++
c12++
c4
c7
z10++
z11++
M+e-45
M+2e-45
z3
c3
z6++
z4
z7++
c8++
c10++
c6
z13++
c5
y5
y6
a2
z2
y2
b2
c2
y3
b3
y4-17
y4
y7++
z8++
z9++
y10++
y11++
z12++
y13++
z8
z10
c10
c11
c12
c13
M+3e-17
M+3e
M+3e-45

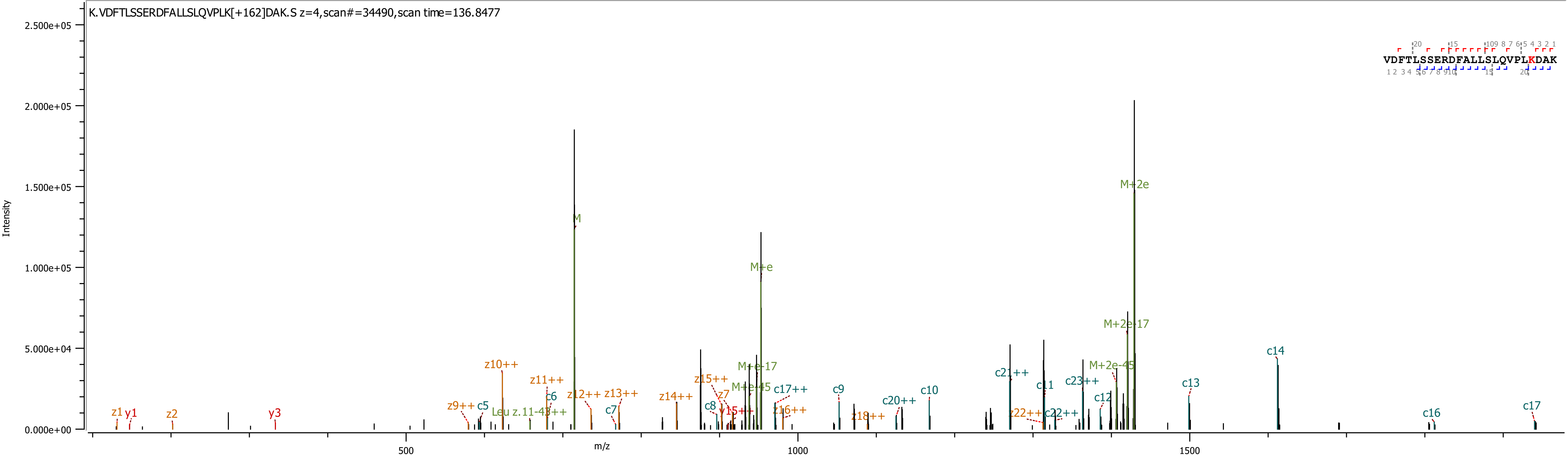

K.VDFTLSSERDFALLSLQVPLK[+162]DAK.S z=4,scan#=34490,scan time=136.8477
VDFTLSSERDFALLSLQVPLKDAK
Intensity
m/z
2.500e+05
2.000e+05
1.500e+05
1.000e+05
5.000e+04
0.000e+00
500
1000
1500
M
M+e
M+2e
M+2e-17
M+2e-45
M+e-17
M+e-45
Leu z.11-43++
z1
y1
z2
y3
z9++
c5
z10++
z11++
c6
z12++
z13++
c7
z14++
c8
z15++
z7
y15++
c17++
z16++
c9
z18++
c20++
c10
c21++
z22++
c11
c22++
c23++
c12
c13
c14
c16
c17

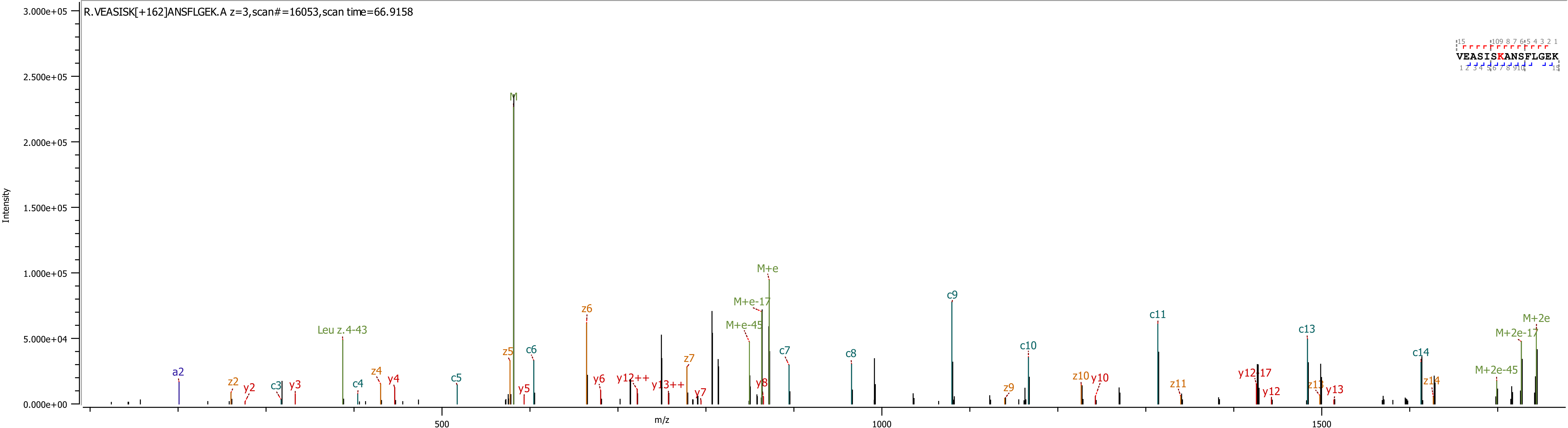

R.VEASISK[+162]ANSFLGEK.A z=3,scan#=16053,scan time=66.9158
VEASISKANSFLGEK
Intensity
m/z
3.000e+05
2.500e+05
2.000e+05
1.500e+05
1.000e+05
5.000e+04
0.000e+00
500
1000
1500
M
M+e
M+e-17
M+e-45
M+2e
M+2e-17
M+2e-45
Leu z.4-43
a2
c3
c4
c5
c6
c7
c8
c9
c10
c11
c13
c14
y2
y3
y4
y5
y6
y7
y8
y10
y12
y13
y12++
y13++
y12-17
z2
z4
z5
z6
z7
z9
z10
z11
z13
z14

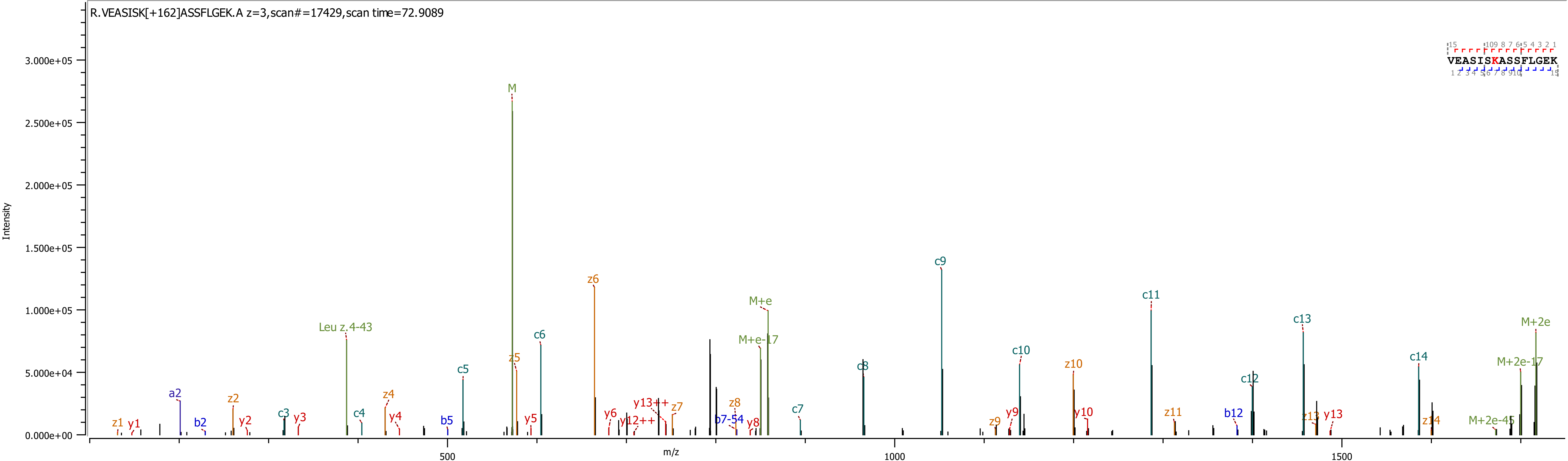
R.VEASISK[+162]ASSFLGEK.A z=3,scan#=17429,scan time=72.9089
VEASISKASSFLGEK
Intensity
m/z
M
z6
c9
M+e
c11
c13
M+2e
Leu z.4-43
c6
M+e-17
c10
z10
c14
M+2e-17
c5
z5
c8
c12
a2
z2
z4
c3
c4
y4
y3
y13++
z7
z8
y12++
b7-54
y8
c7
y6
y1
z1
b2
y2
b5
y5
z9
y9
y10
z11
b12
z13
y13
z14
M+2e-45
0.000e+00
5.000e+04
1.000e+05
1.500e+05
2.000e+05
2.500e+05
3.000e+05
500
1000
1500

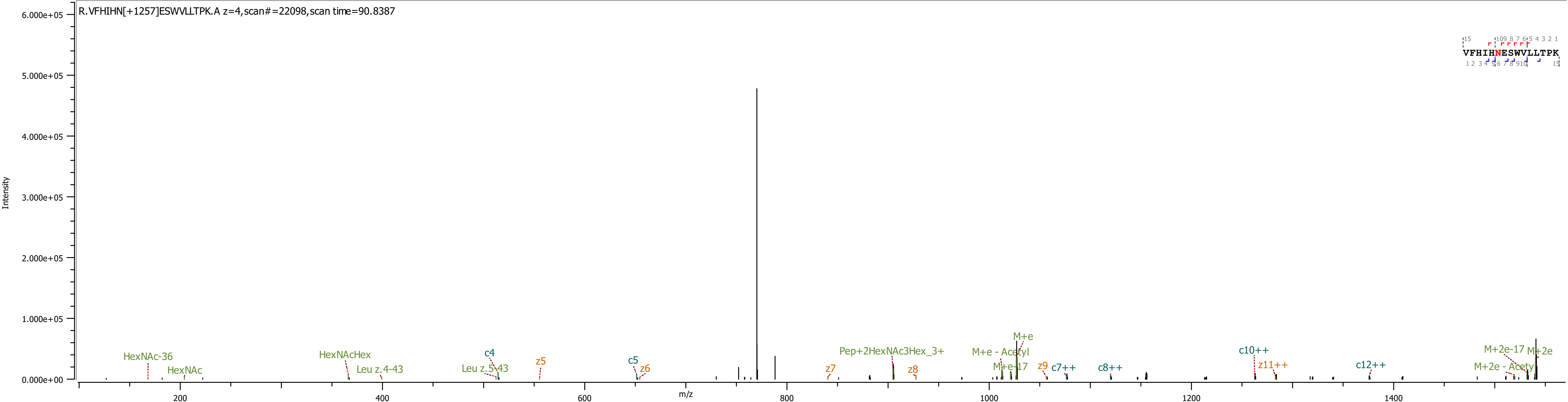

R.VFHIHN[+1257]ESWVLLTPK.A z=4,scan#=22098,scan time=90.8387
VFHIHNESWVLLTPK
Intensity
m/z
HexNAc-36
HexNAc
HexNAcHex
Leu z.4-43
c4
Leu z.5-43
z5
c5
z6
z7
Pep+2HexNAc3Hex_3+
z8
M+e - Acetyl
M+e
M+e-17
z9
c7++
c8++
c10++
z11++
c12++
M+2e-17
M+2e
M+2e - Acetyl

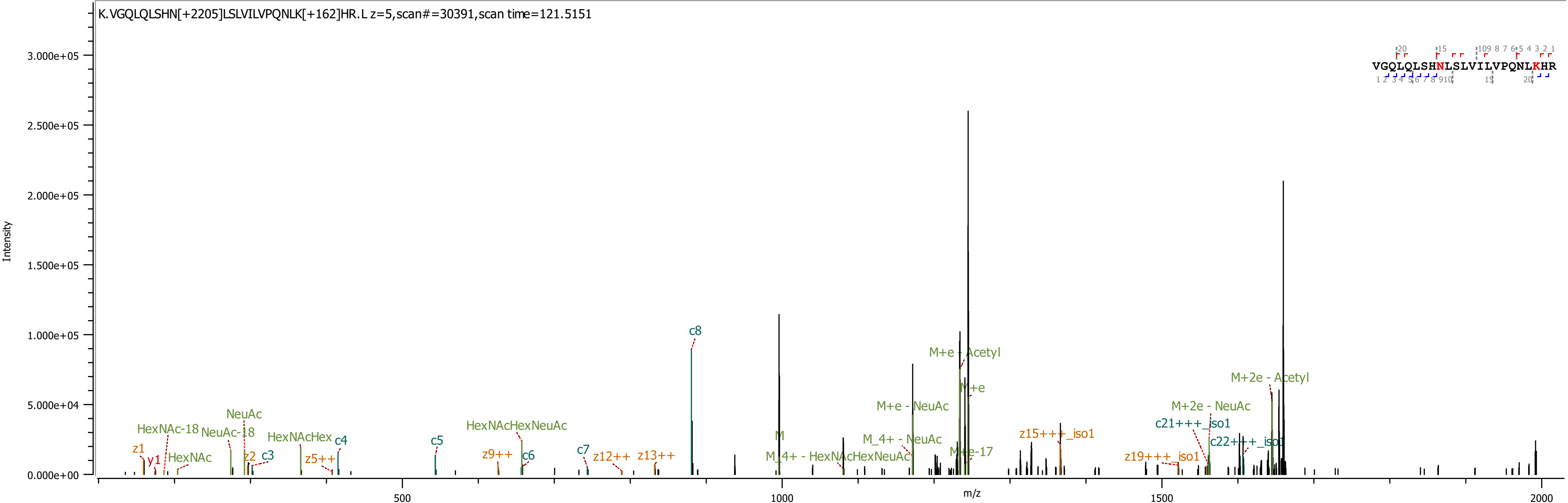
K.VGQLQLSHN[+2205]LSLVILVPQNLK[+162]HR.L z=5,scan#=30391,scan time=121.5151
VGQLQLSHNLSLVILVPQNLKHR
Intensity
3.000e+05
2.500e+05
2.000e+05
1.500e+05
1.000e+05
5.000e+04
0.000e+00
m/z
500
1000
1500
2000
z1
y1
HexNAc-18
HexNAc
NeuAc-18
NeuAc
z2
c3
HexNAcHex
z5++
c4
c5
z9++
HexNAcHexNeuAc
c6
c7
z12++
z13++
c8
M
M_4+ - HexNAcHexNeuAc
M_4+ - NeuAc
M+e - NeuAc
M+e - Acetyl
M+e
M+e-17
z15+++_iso1
z19+++_iso1
c21+++_iso1
c22+++_iso1
M+2e - NeuAc
M+2e - Acetyl

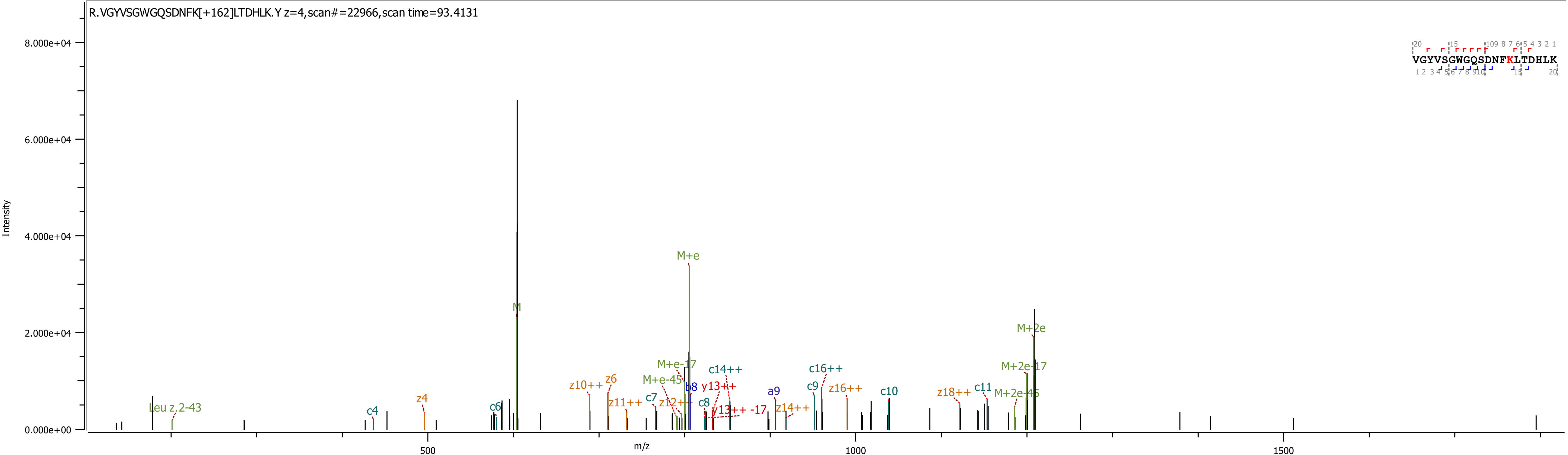

R.VGYVSGWGQSDNFK[+162]LTDHLK.Y z=4,scan#=22966,scan time=93.4131
VGYVSGWGQSDNFKLTDHLK
Intensity
m/z
8.000e+04
6.000e+04
4.000e+04
2.000e+04
0.000e+00
500
1000
1500
M
M+e
M+2e
M+e-17
M+e-45
M+2e-17
M+2e-45
Leu z.2-43
c4
z4
c6
z10++
z6
z11++
c7
z12++
b8
y13++
c8
y13++ -17
c14++
a9
z14++
c9
c16++
z16++
c10
z18++
c11

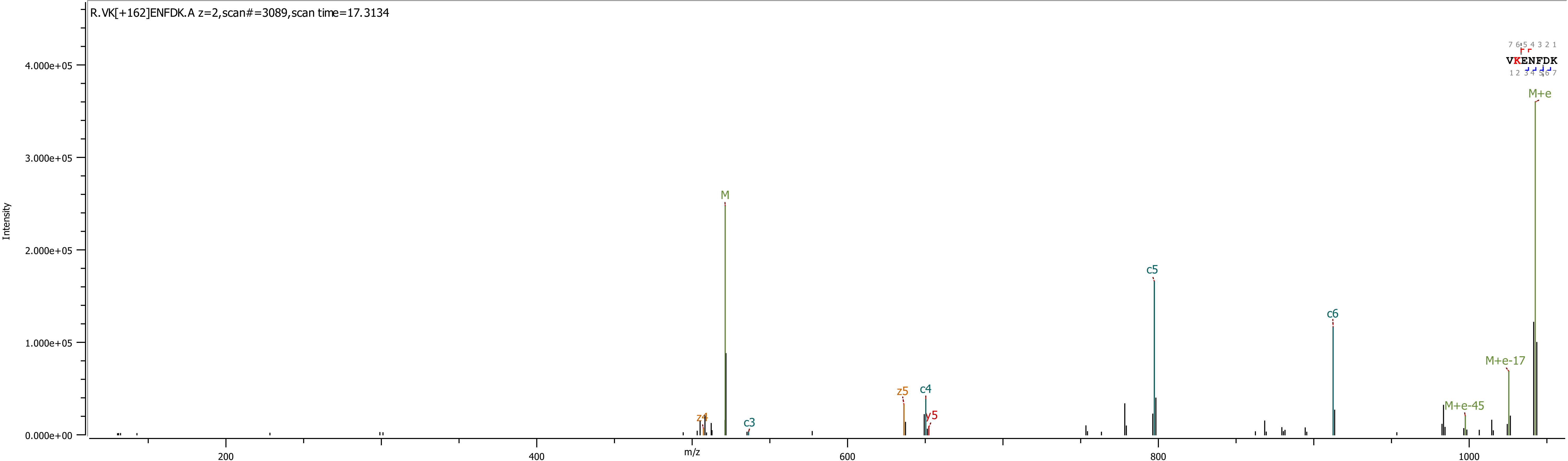
R.VK[+162]ENFDK.A z=2,scan#=3089,scan time=17.3134
Intensity
m/z
VKENFDK
M
c5
c6
M+e
M+e-17
M+e-45
z4
c3
z5
c4
y5
4.000e+05
3.000e+05
2.000e+05
1.000e+05
0.000e+00
200
400
600
800
1000

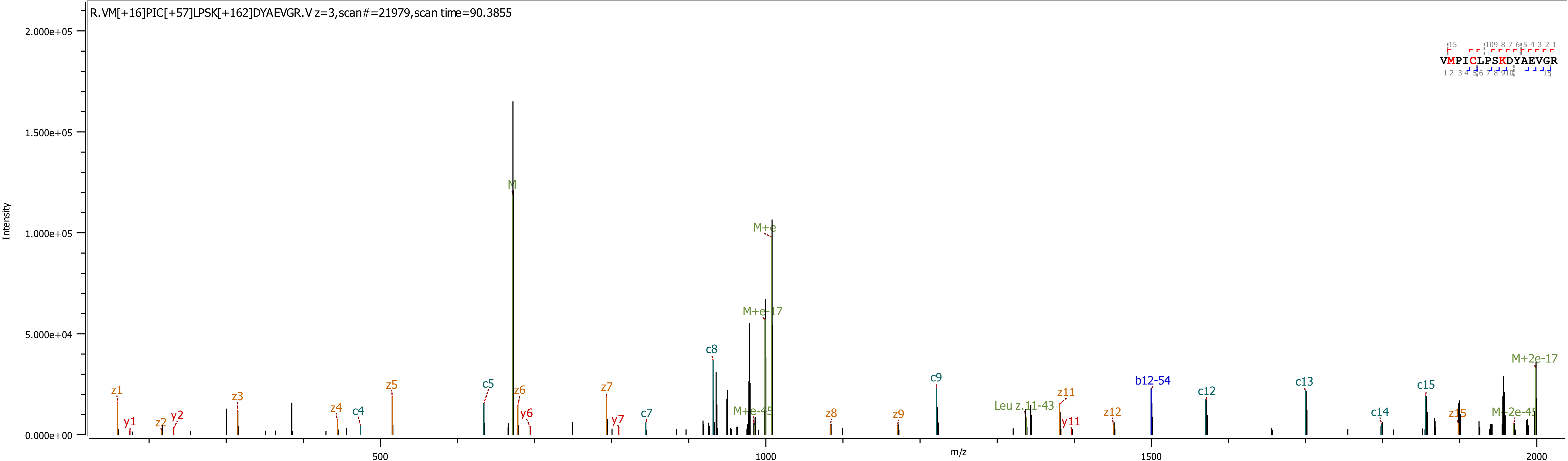

R.VM[+16]PIC[+57]LPSK[+162]DYAEVGR.V z=3,scan#=21979,scan time=90.3855
Intensity
m/z
2.000e+05
1.500e+05
1.000e+05
5.000e+04
0.000e+00
500
1000
1500
2000
VMPICLPSKDYAEVGR
M
M+e
M+e-17
M+e-45
M+2e-17
M+2e-45
Leu z.11-43
b12-54
z1
y1
z2
y2
z3
z4
c4
z5
c5
z6
y6
z7
y7
c7
c8
z8
z9
c9
z11
y11
z12
c12
c13
c14
c15
z15

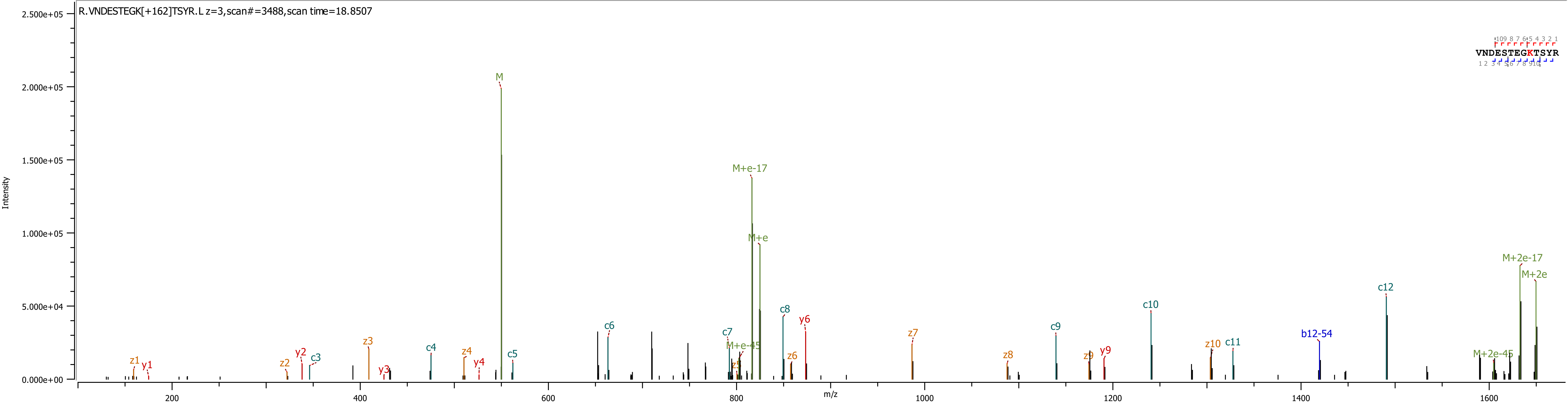
R.VNDESTEGK[+162]TSYR.L z=3,scan#=3488,scan time=18.8507
VNDESTEGKTSYR
Intensity
m/z
M
M+e-17
M+e
M+e-45
M+2e-17
M+2e
M+2e-45
c3
c4
c5
c6
c7
c8
c9
c10
c11
c12
y1
y2
y3
y4
y6
y9
z1
z2
z3
z4
z5
z6
z7
z8
z9
z10
b12-54

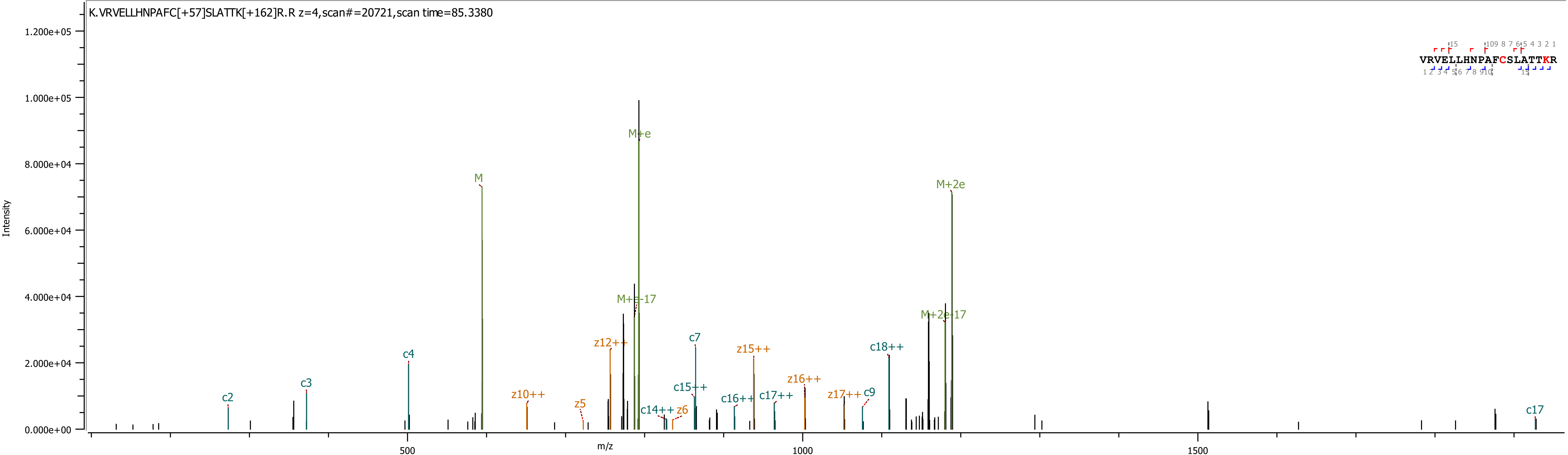
K.VRVELLHNPAFC[+57]SLATTK[+162]R.R z=4,scan#=20721,scan time=85.3380
Intensity
m/z
VRVELLHNPAFCSLATTKR
M
M+e
M+e-17
M+2e
M+2e-17
c2
c3
c4
c7
c9
c17
c14++
c15++
c16++
c17++
c18++
z5
z6
z10++
z12++
z15++
z16++
z17++

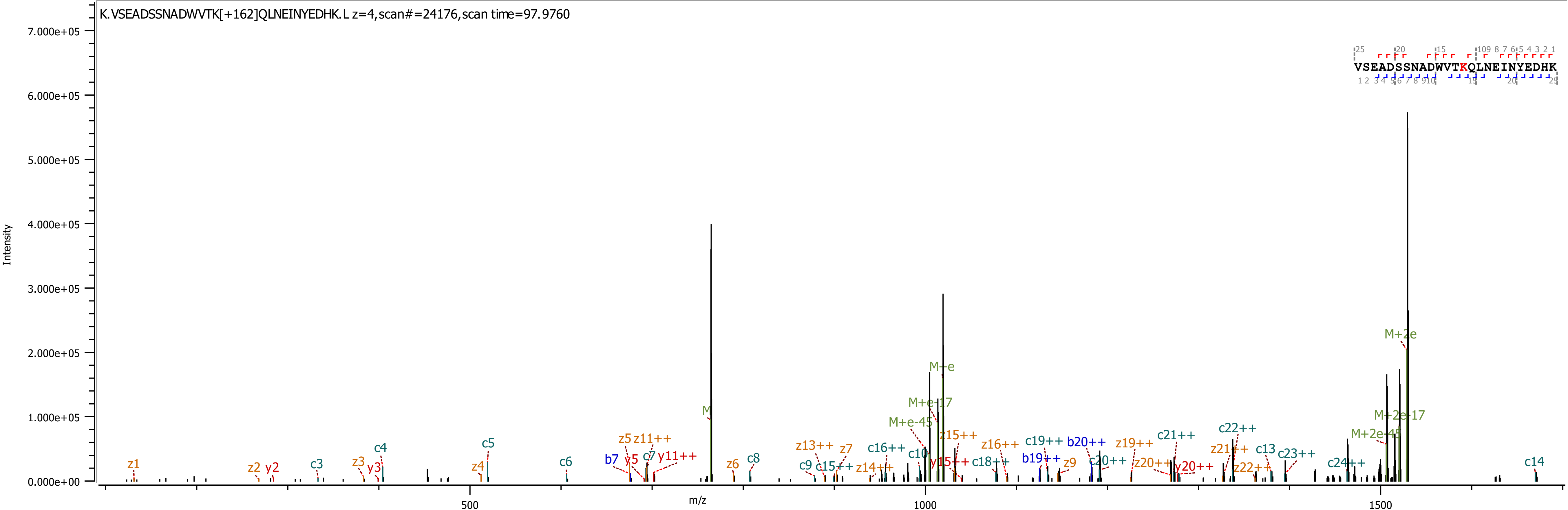
K.VSEADSSNADWVTK[+162]QLNEINYEDHK.L z=4,scan#=24176,scan time=97.9760
Intensity
m/z
VSEADSSNADWVTKQLNEINYEDHK
M+2e
M+e
M
M+2e-17
M+2e-45
M+e-17
M+e-45
c4
c5
c6
c7
c8
c9
c10
c13
c14
b7
b19++
b20++
y2
y3
y5
y11++
y15++
y20++
z1
z2
z3
z4
z5
z6
z7
z9
z11++
z13++
z14++
z15++
z16++
z19++
z20++
z21++
z22++
c3
c15++
c16++
c18++
c19++
c20++
c21++
c22++
c23++
c24++

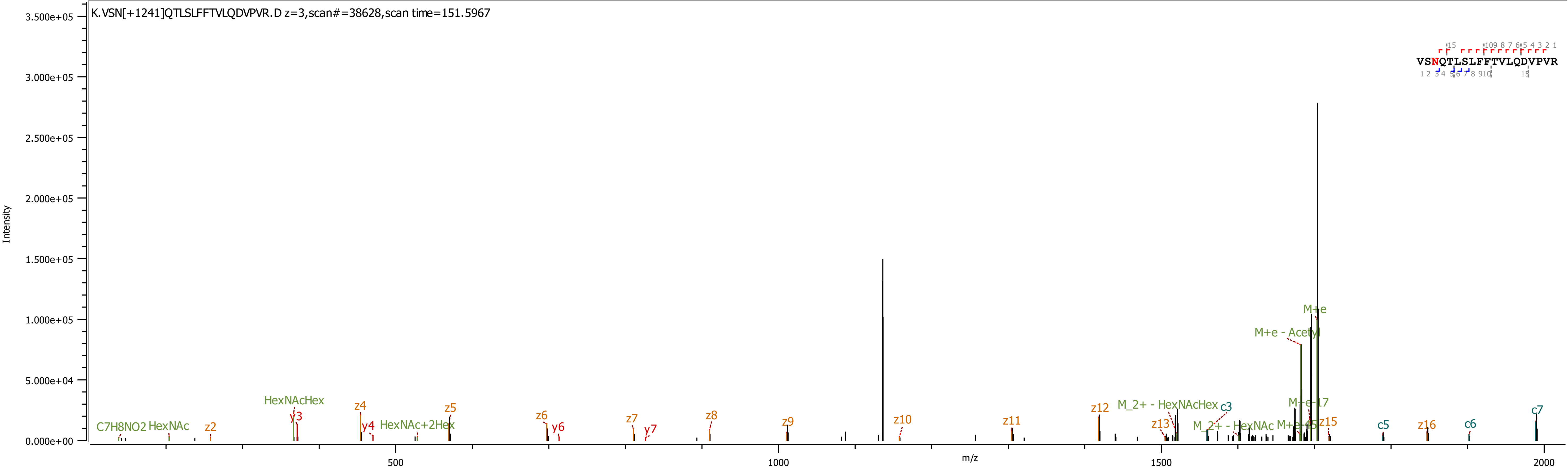
K.VSN[+1241]QTLSLFFTVLQDVPVR.D z=3,scan#=38628,scan time=151.5967
VSNQTLSLFFTVLQDVPVR
Intensity
m/z
3.500e+05
3.000e+05
2.500e+05
2.000e+05
1.500e+05
1.000e+05
5.000e+04
0.000e+00
500
1000
1500
2000
C7H8NO2
HexNAc
z2
HexNAcHex
y3
z4
y4
HexNAc+2Hex
z5
z6
y6
z7
y7
z8
z9
z10
z11
z12
z13
M_2+ - HexNAcHex
c3
M_2+ - HexNAc
M+e - Acetyl
M+e
M+e-17
z15
c5
z16
c6
c7

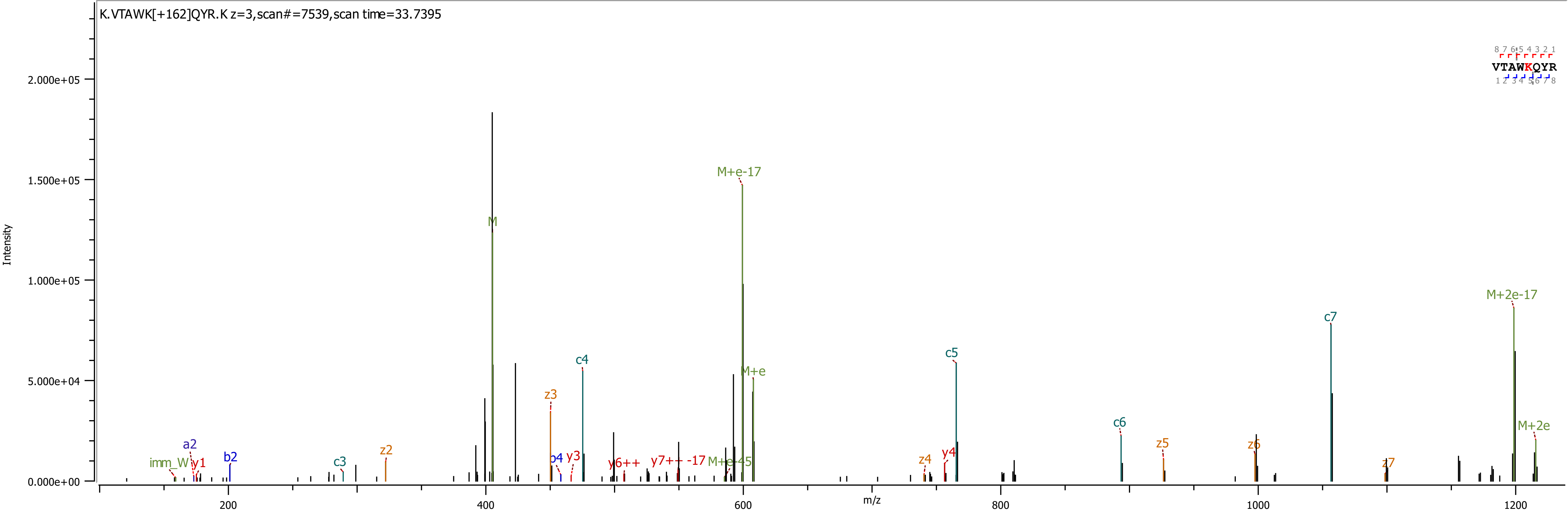

K.VTAWK[+162]QYR.K z=3,scan#=7539,scan time=33.7395
Intensity
2.000e+05
1.500e+05
1.000e+05
5.000e+04
0.000e+00
m/z
200
400
600
800
1000
1200
VTAWKQYR
M
M+e-17
M+e
M+e-45
M+2e-17
M+2e
imm_W
a2
b2
b4
y1
y3
y4
y6++
y7++ -17
c3
c4
c5
c6
c7
z2
z3
z4
z5
z6
z7

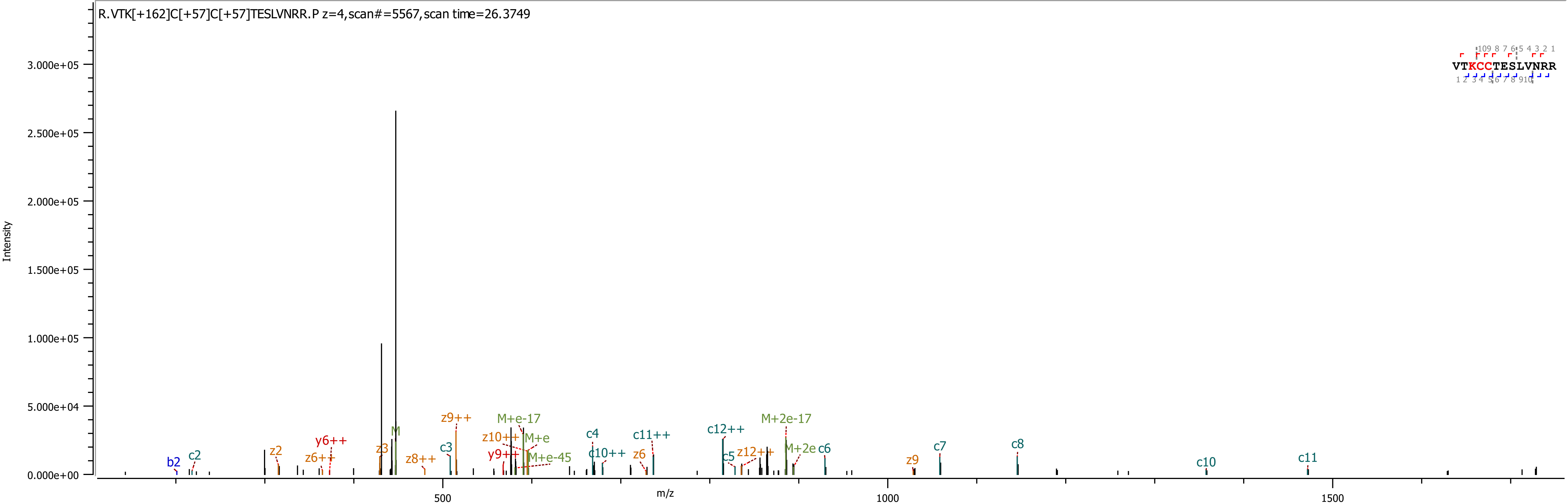
R.VTK[+162]C[+57]C[+57]TESLVNRR.P z=4,scan#=5567,scan time=26.3749
Intensity
m/z
VTKCCTESLVNRR
b2
c2
z2
y6++
z6++
z3
M
z8++
c3
z9++
z10++
y9++
M+e-17
M+e
M+e-45
c4
c10++
c11++
z6
c12++
c5
z12++
M+2e-17
M+2e
c6
z9
c7
c8
c10
c11
500
1000
1500

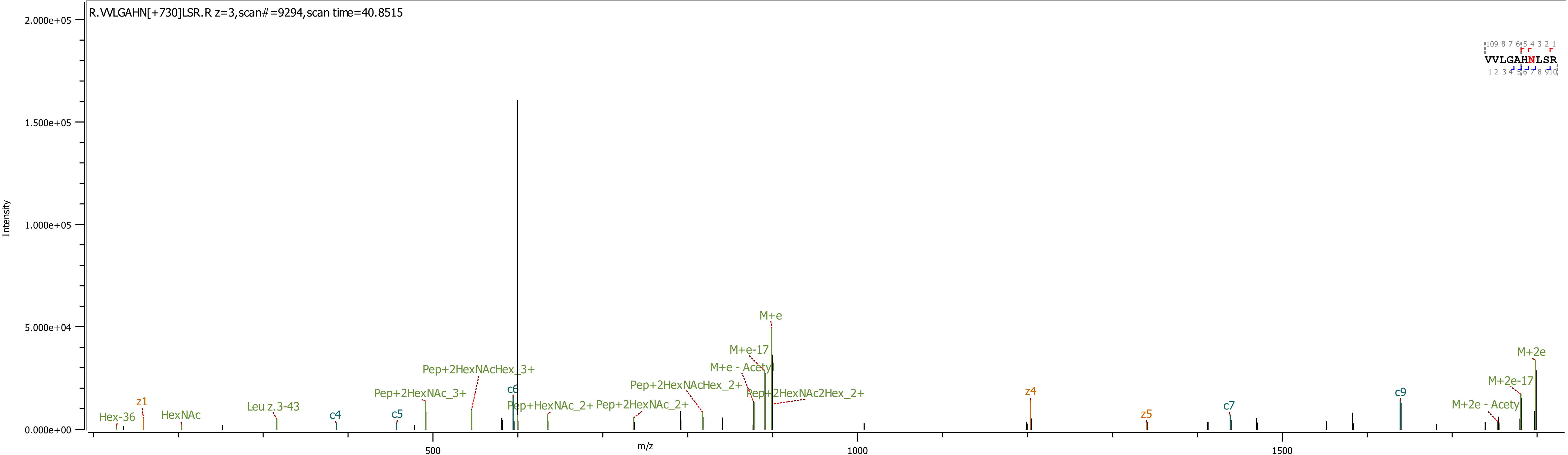
R.VVLGAHN[+730]LSR.R z=3,scan#=9294,scan time=40.8515
Intensity
m/z
2.000e+05
1.500e+05
1.000e+05
5.000e+04
0.000e+00
500
1000
1500
Hex-36
z1
HexNAc
Leu z.3-43
c4
c5
Pep+2HexNAc_3+
Pep+2HexNAcHex_3+
c6
Pep+HexNAc_2+
Pep+2HexNAc_2+
Pep+2HexNAcHex_2+
M+e - Acetyl
M+e-17
M+e
Pep+2HexNAc2Hex_2+
z4
z5
c7
c9
M+2e - Acetyl
M+2e-17
M+2e
VVLGAHNLSR

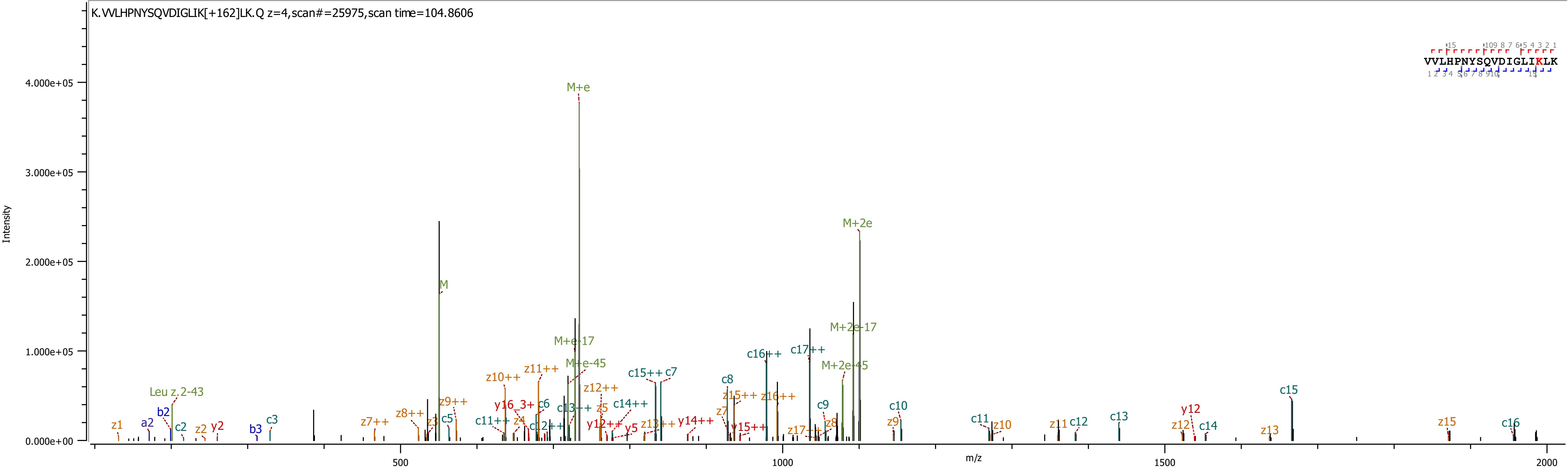

K.VVLHPNYSQVDIGLIK[+162]LK.Q z=4,scan#=25975,scan time=104.8606
VVLHPNYSQVDIGLIKLK
Intensity
m/z
M+e
M+2e
M
M+e-17
M+e-45
M+2e-17
M+2e-45
Leu z.2-43

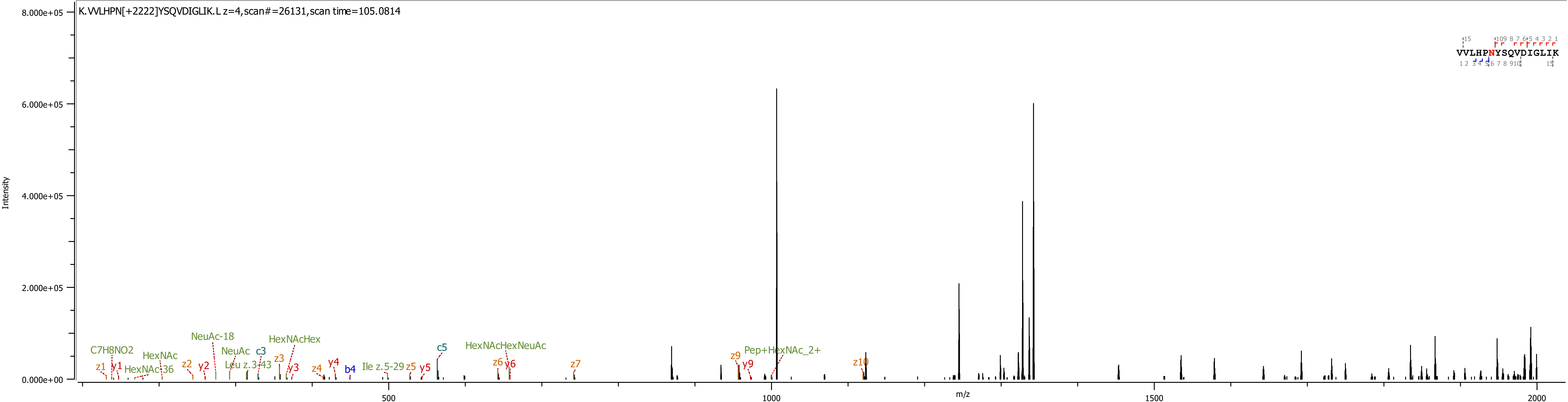

K.VVLHPN[+2222]YSQVDIGLIK.L z=4,scan#=26131,scan time=105.0814
VVLHPNYSQVDIGLIK
Intensity
m/z
8.000e+05
6.000e+05
4.000e+05
2.000e+05
0.000e+00
500
1000
1500
2000
C7H8NO2
z1
y1
HexNAc-36
HexNAc
z2
y2
NeuAc-18
NeuAc
Leu z.3-43
c3
z3
HexNAcHex
y3
z4
y4
b4
Ile z.5-29
z5
y5
c5
z6
y6
HexNAcHexNeuAc
z7
z9
y9
Pep+HexNAc_2+
z10

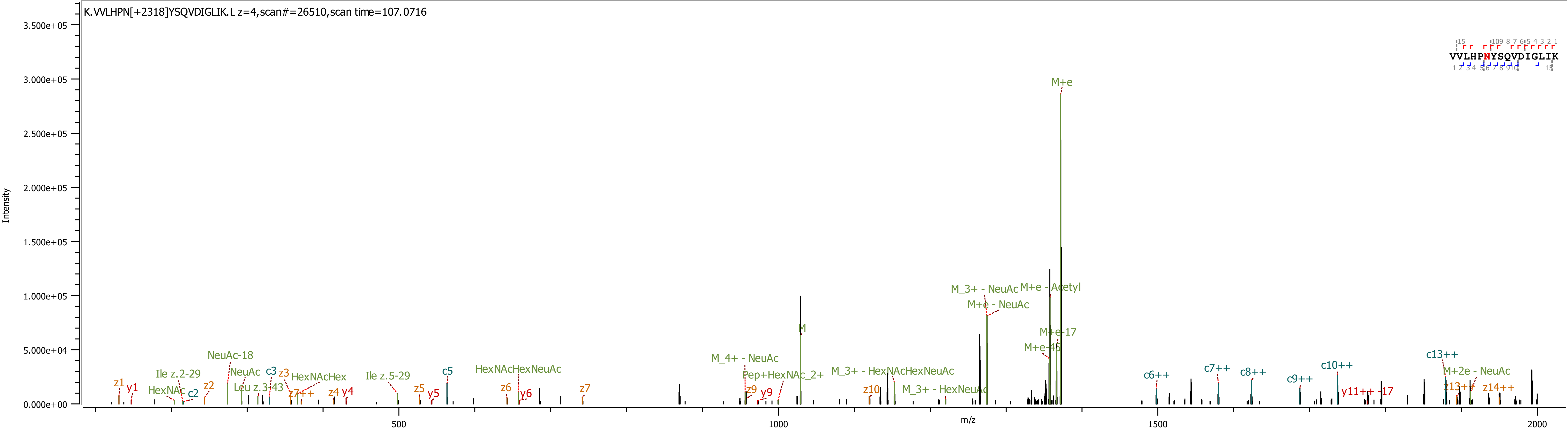

K.VVLHPN[+2318]YSQVDIGLIK.L z=4,scan#=26510,scan time=107.0716
Intensity
m/z
VVLHPNYSQVDIGLIK
M+e
M_3+ - NeuAc
M+e - Acetyl
M+e - NeuAc
M
M+e-17
M+e-46
NeuAc-18
Ile z.2-29
NeuAc
c3
z3
HexNAcHex
Ile z.5-29
c5
HexNAcHexNeuAc
M_4+ - NeuAc
Pep+HexNAc_2+
M_3+ - HexNAcHexNeuAc
M_3+ - HexNeuAc
z1
y1
HexNAc
c2
z2
Leu z.3-43
z7++
z4
y4
z5
y5
z6
y6
z7
z9
y9
z10
c6++
c7++
c8++
c9++
c10++
y11++-17
c13++
M+2e - NeuAc
z13++
z14++

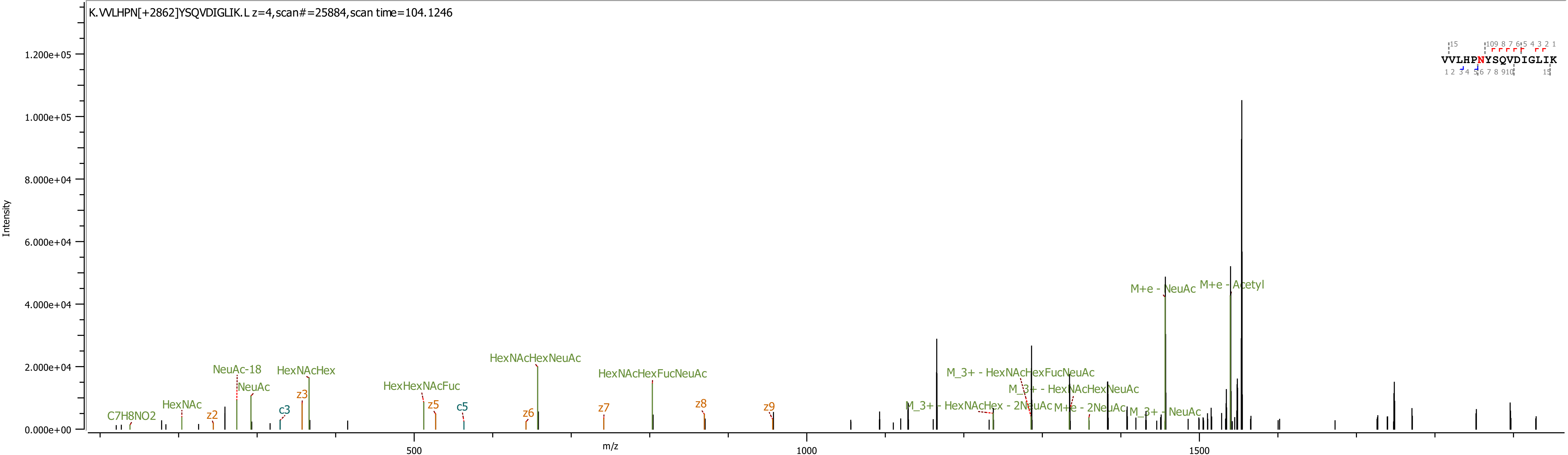
K.VVLHPN[+2862]YSQVDIGLIK.L z=4,scan#=25884,scan time=104.1246
VVLHPNYSQVDIGLIK
Intensity
m/z
C7H8NO2
HexNAc
z2
NeuAc-18
NeuAc
c3
z3
HexNAcHex
HexHexNAcFuc
z5
c5
HexNAcHexNeuAc
z6
z7
HexNAcHexFucNeuAc
z8
z9
M_3+ - HexNAcHex - 2NeuAc
M_3+ - HexNAcHexFucNeuAc
M_3+ - HexNAcHexNeuAc
M+e - 2NeuAc
M_3+ - NeuAc
M+e - NeuAc
M+e - Acetyl

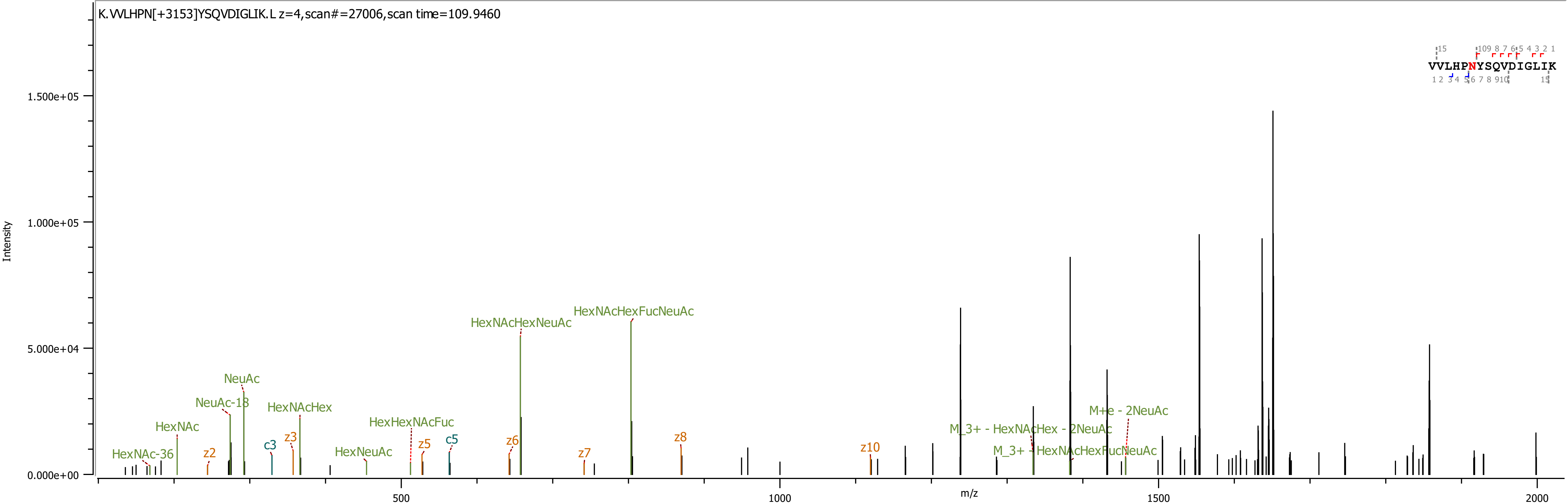

K.VVLHPN[+3153]YSQVDIGLIK.L z=4,scan#=27006,scan time=109.9460
VVLHPNYSQVDIGLIK
Intensity
1.500e+05
1.000e+05
5.000e+04
0.000e+00
m/z
500
1000
1500
2000
HexNAc-36
HexNAc
z2
NeuAc-18
NeuAc
c3
z3
HexNAcHex
HexNeuAc
HexHexNAcFuc
z5
c5
z6
HexNAcHexNeuAc
z7
HexNAcHexFucNeuAc
z8
z10
M_3+ - HexNAcHex - 2NeuAc
M_3+ - HexNAcHexFucNeuAc
M+e - 2NeuAc

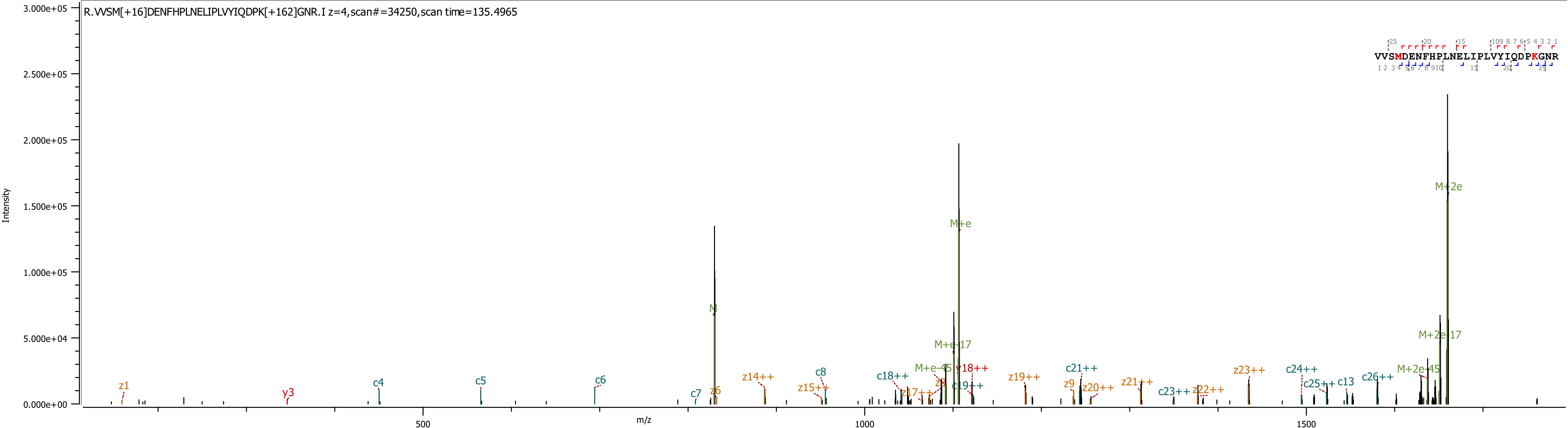
R.VVSM[+16]DENFHPLNELIPLVYIQDPK[+162]GNR.I z=4,scan#=34250,scan time=135.4965
VVSMDENFHPLNELIPLVYIQDPKGNR
Intensity
m/z
3.000e+05
2.500e+05
2.000e+05
1.500e+05
1.000e+05
5.000e+04
0.000e+00
500
1000
1500
z1
y3
c4
c5
c6
c7
z6
M
z14++
z15++
c8
c18++
z17++
z8
M+e-45
y18++
c19++
M+e-17
M+e
z19++
z9
c21++
z20++
z21++
c23++
z22++
z23++
c24++
c25++
c13
c26++
M+2e-45
M+2e-17
M+2e

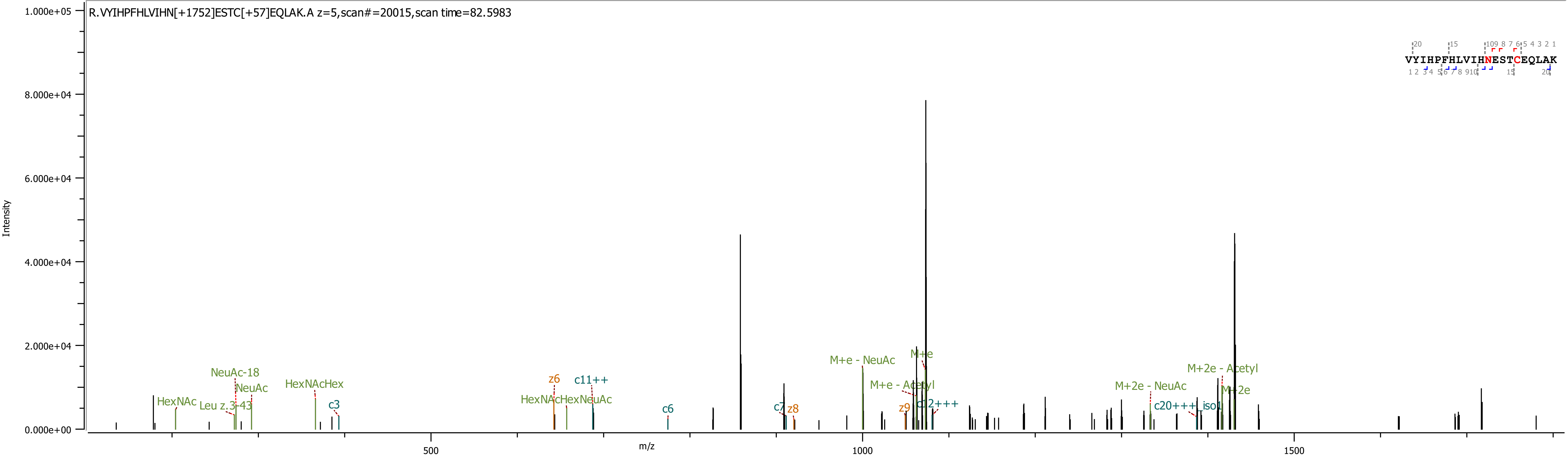
R.VYIHPFHLVIHN[+1752]ESTC[+57]EQLAK.A z=5,scan#=20015,scan time=82.5983
VYIHPFHLVIHNESTCEQLAK
Intensity
m/z
1.000e+05
8.000e+04
6.000e+04
4.000e+04
2.000e+04
0.000e+00
500
1000
1500
HexNAc
NeuAc-18
NeuAc
Leu z.3-43
HexNAcHex
c3
z6
c11++
HexNAcHexNeuAc
c6
c7
z8
M+e - NeuAc
M+e - Acetyl
M+e
z9
c12+++
M+2e - NeuAc
c20+++
isoI
M+2e - Acetyl
M+2e

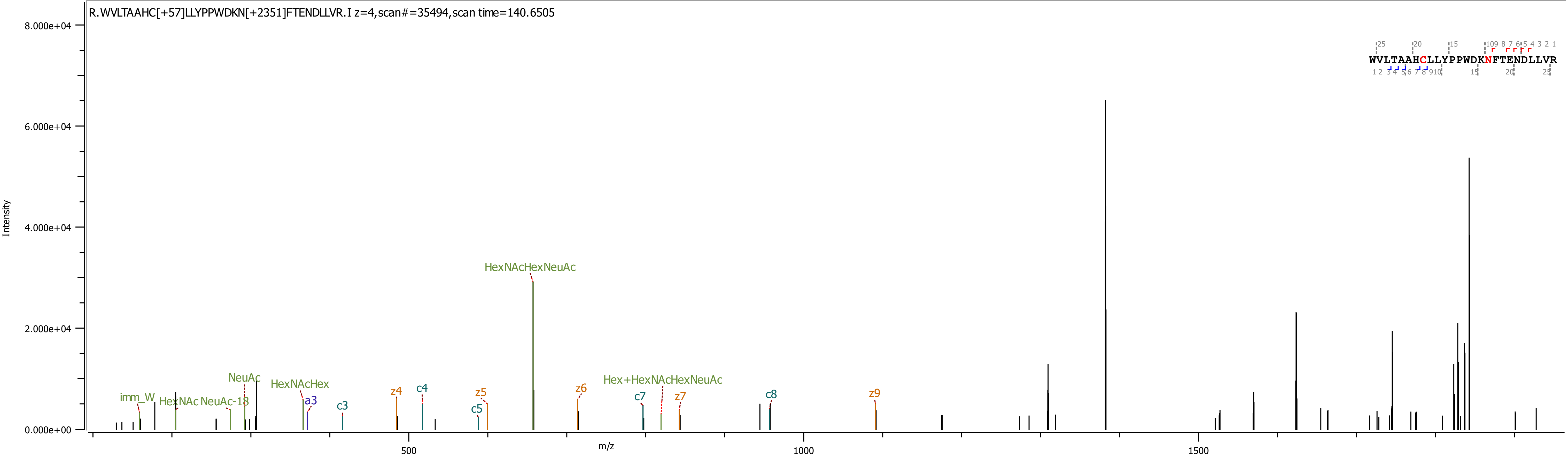

R.WVLTAAHC[+57]LLYPPWDKN[+2351]FTENDLLVR.I z=4,scan#=35494,scan time=140.6505
WVLTAAHCLLYPPWDKNFTENDLLVR
Intensity
8.000e+04
6.000e+04
4.000e+04
2.000e+04
0.000e+00
m/z
500
1000
1500
imm_W
HexNAc
NeuAc-18
NeuAc
HexNAcHex
a3
c3
z4
c4
c5
z5
HexNAcHexNeuAc
z6
c7
Hex+HexNAcHexNeuAc
z7
c8
z9

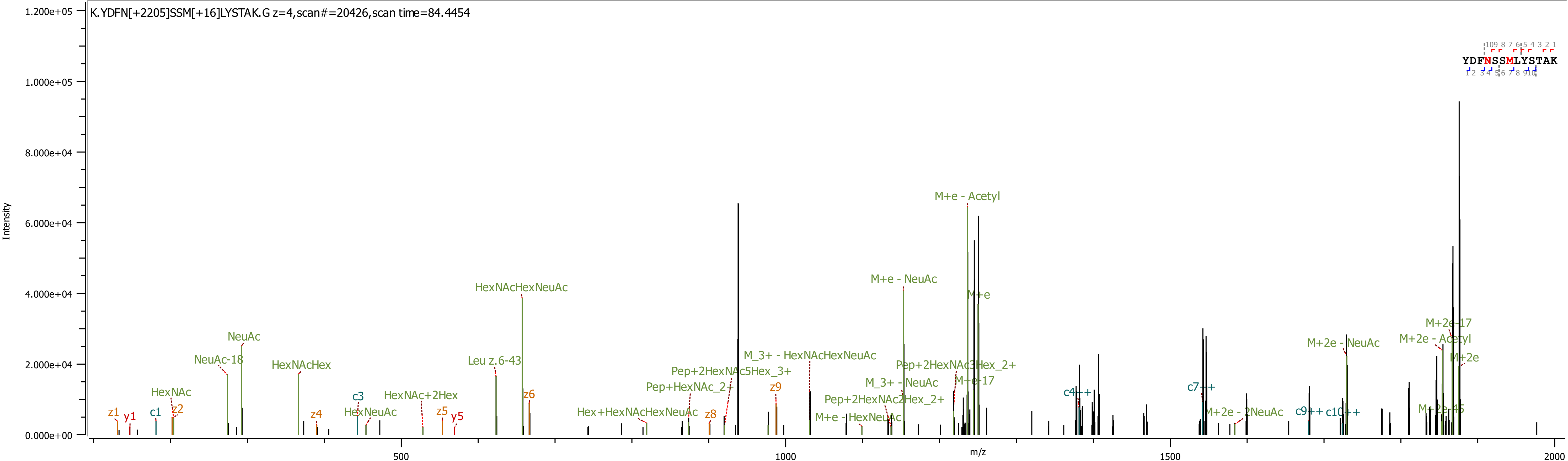
K.YDFN[+2205]SSM[+16]LYSTAK.G z=4,scan#=20426,scan time=84.4454
Intensity
m/z
YDFNSSMLYSTAK
M+e - Acetyl
M+e - NeuAc
M+e
HexNAcHexNeuAc
NeuAc
NeuAc-18
HexNAcHex
HexNAc
Leu z.6-43
HexNAc+2Hex
HexNeuAc
M_3+ - HexNAcHexNeuAc
Pep+2HexNAc5Hex_3+
Pep+HexNAc_2+
Pep+2HexNAc3Hex_2+
M_3+ - NeuAc
Pep+2HexNAc2Hex_2+
M+e - HexNeuAc
M+e-17
Hex+HexNAcHexNeuAc
M+2e-17
M+2e - Acetyl
M+2e - NeuAc
M+2e
M+2e - 2NeuAc
M+2e-45
c1
c3
c4++
c7++
c9++
c10++
z1
z2
z4
z5
z6
z8
z9
y1
y5

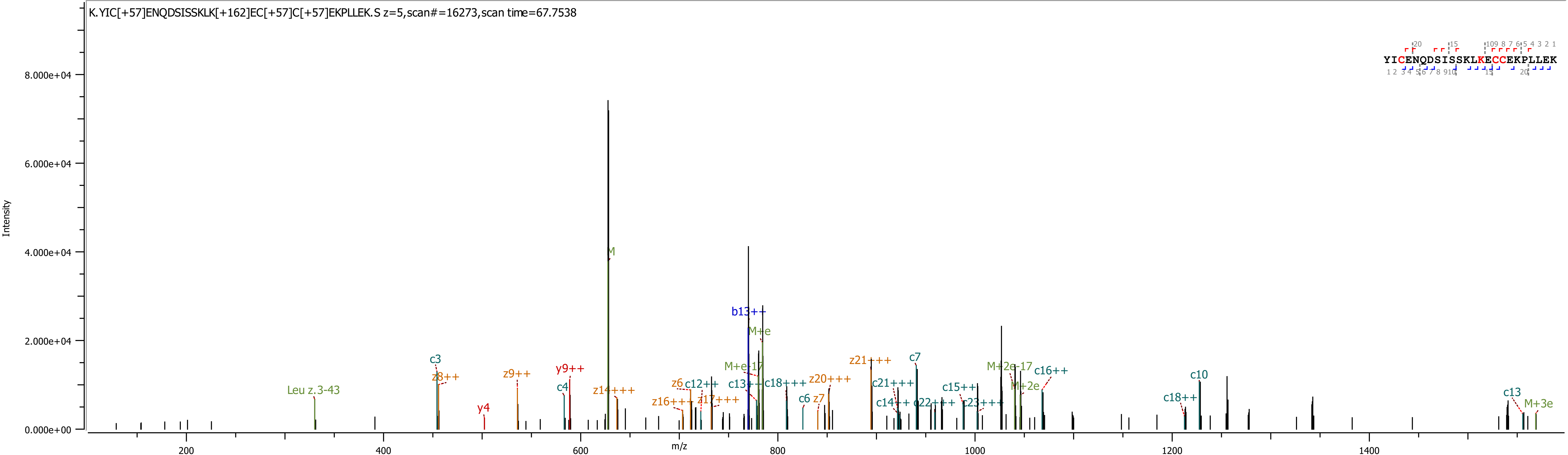
K.YIC[+57]ENQDSISSKLK[+162]EC[+57]C[+57]EKPLLEK.S z=5,scan#=16273,scan time=67.7538
Intensity
m/z
YICENQDSISSKLKECCEKPLLEK
Leu z.3-43
c3
z8++
z9++
y9++
y4
c4
M
z14+++
b13++
M+e
M+e-17
z6
c12++
c13++
c18+++
z16+++
z17+++
c6
z7
z20+++
z21+++
c21+++
c14++
c22+++
c7
c15++
c23+++
M+2e-17
c16++
M+2e
c10
c18++
c13
M+3e

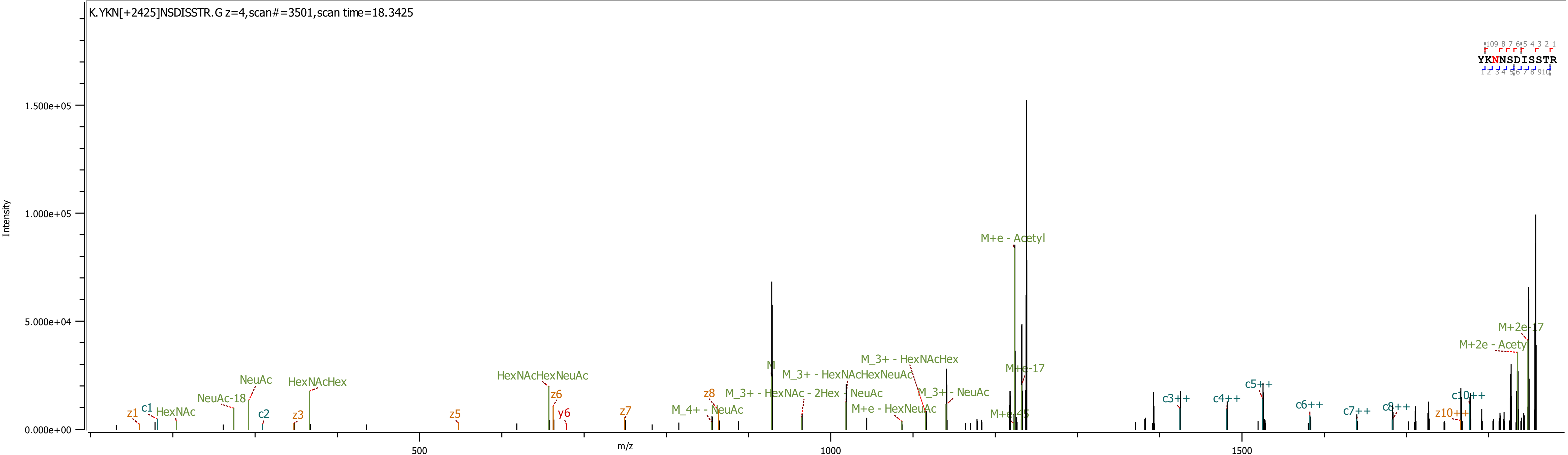

K.YKN[+2425]NSDISSTR.G z=4,scan#=3501,scan time=18.3425
Intensity
m/z
1.500e+05
1.000e+05
5.000e+04
0.000e+00
500
1000
1500
YKNNSDISSTR
M+e - Acetyl
M_3+ - HexNAcHex
M_3+ - HexNAcHexNeuAc
M_3+ - HexNAc - 2Hex - NeuAc
M_3+ - NeuAc
M+e - HexNeuAc
M_4+ - NeuAc
M+e-17
M+e-45
M+2e-17
M+2e - Acetyl
HexNAcHexNeuAc
HexNAcHex
NeuAc
NeuAc-18
HexNAc
M
z1
c1
c2
z3
z5
z6
y6
z7
z8
c3++
c4++
c5++
c6++
c7++
c8++
c10++
z10++

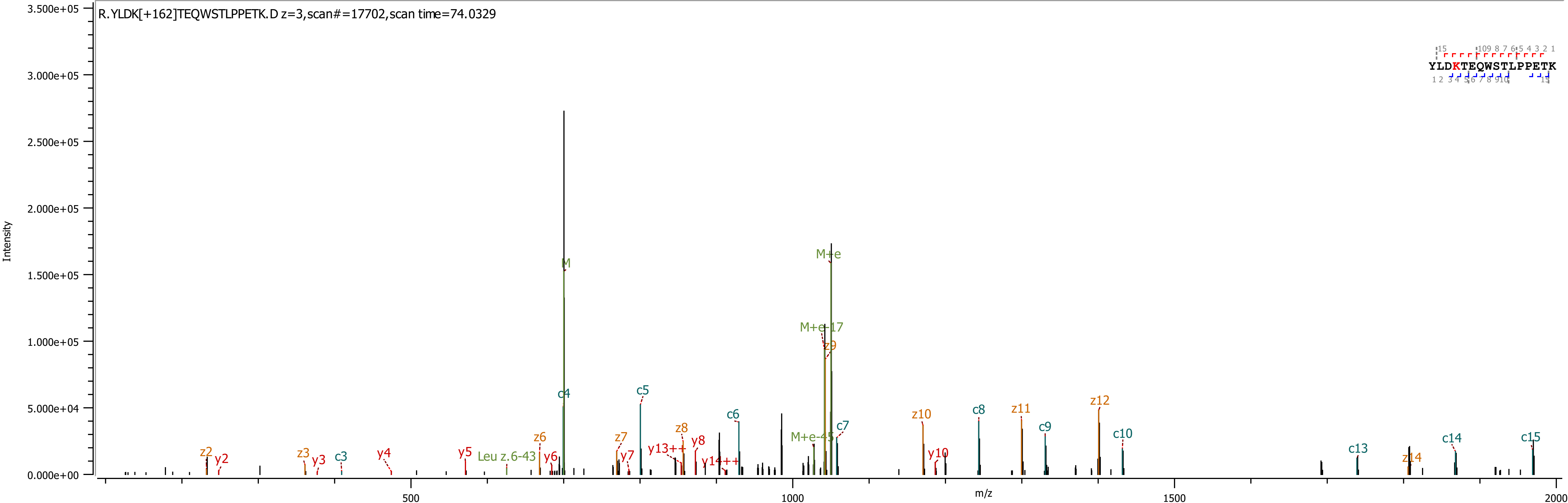
R.YLDK[+162]TEQWSTLPPETK.D z=3,scan#=17702,scan time=74.0329
Intensity
m/z
3.500e+05
3.000e+05
2.500e+05
2.000e+05
1.500e+05
1.000e+05
5.000e+04
0.000e+00
500
1000
1500
2000
YLDKTEQWSTLPPETK
M
M+e
M+e-17
M+e-45
Leu z.6-43
c3
c4
c5
c6
c7
c8
c9
c10
c13
c14
c15
z2
z3
z6
z7
z8
z9
z10
z11
z12
z14
y2
y3
y4
y5
y6
y7
y8
y10
y13++
y14++

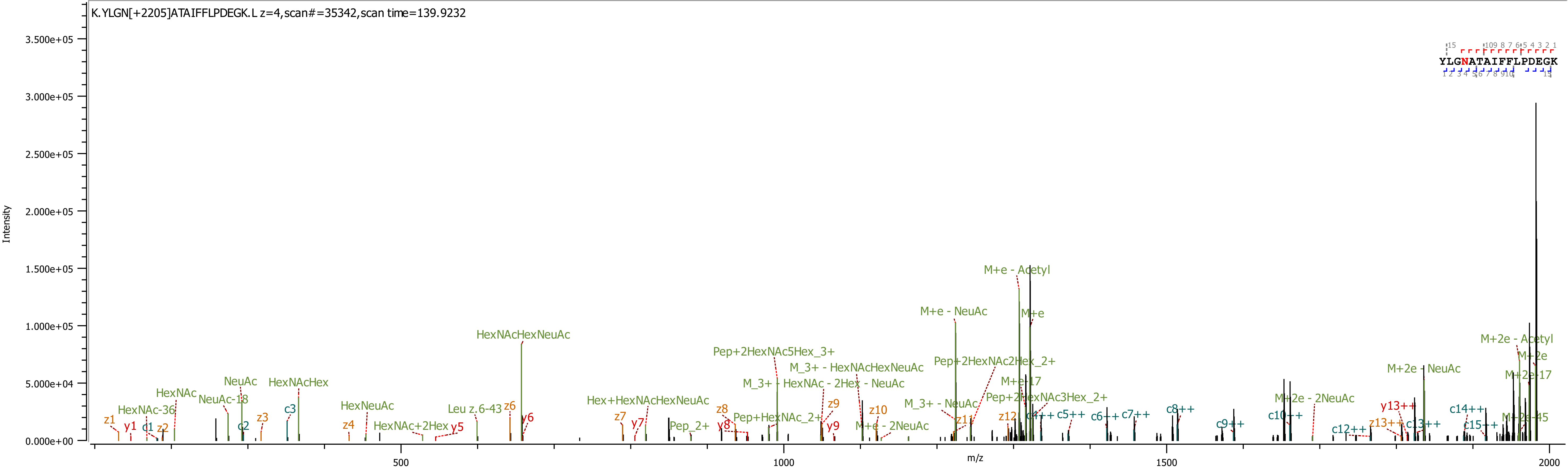

K.YLGN[+2205]ATAIFFLPDEGK.L z=4,scan#=35342,scan time=139.9232
YLGNATAIFFLPDEGK
Intensity
m/z
3.500e+05
3.000e+05
2.500e+05
2.000e+05
1.500e+05
1.000e+05
5.000e+04
0.000e+00
500
1000
1500
2000
HexNAcHexNeuAc
M+e - Acetyl
M+e - NeuAc
M+e
Pep+2HexNAc5Hex_3+
M_3+ - HexNAcHexNeuAc
M_3+ - HexNAc - 2Hex - NeuAc
Pep+2HexNAc2Hex_2+
M+e-17
Pep+2HexNAc3Hex_2+
M_3+ - NeuAc
M+2e - Acetyl
M+2e
M+2e-17
M+2e - NeuAc
M+2e - 2NeuAc
M+2e-45
HexNAc
HexNAc-36
NeuAc
NeuAc-18
HexNAcHex
HexNeuAc
HexNAc+2Hex
Leu z.6-43
Hex+HexNAcHexNeuAc
Pep_2+
Pep+HexNAc_2+
M+e - 2NeuAc
z1
y1
c1
z2
c2
z3
c3
z4
y5
z6
y6
z7
y7
z8
y8
z9
y9
z10
z11
z12
c4++
c5++
c6++
c7++
c8++
c9++
c10++
c12++
y13++
z13++
c13++
c14++
c15++

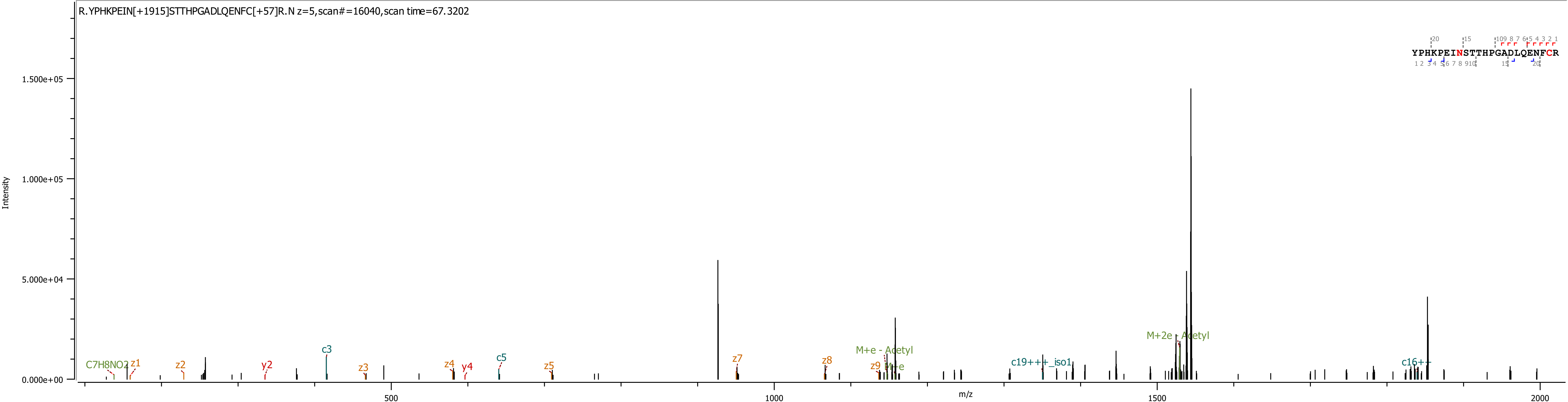
R.YPHKPEIN[+1915]STTHPGADLQENFC[+57]R.N z=5,scan#=16040,scan time=67.3202
Intensity
m/z
YPHKPEINSTTHPGADLQENFCR
C7H8NO2
z1
z2
y2
c3
z3
z4
y4
c5
z5
z7
z8
z9
M+e - Acetyl
M+e
c19+++_iso1
M+2e - Acetyl
c16++
0.000e+00
5.000e+04
1.000e+05
1.500e+05
500
1000
1500
2000

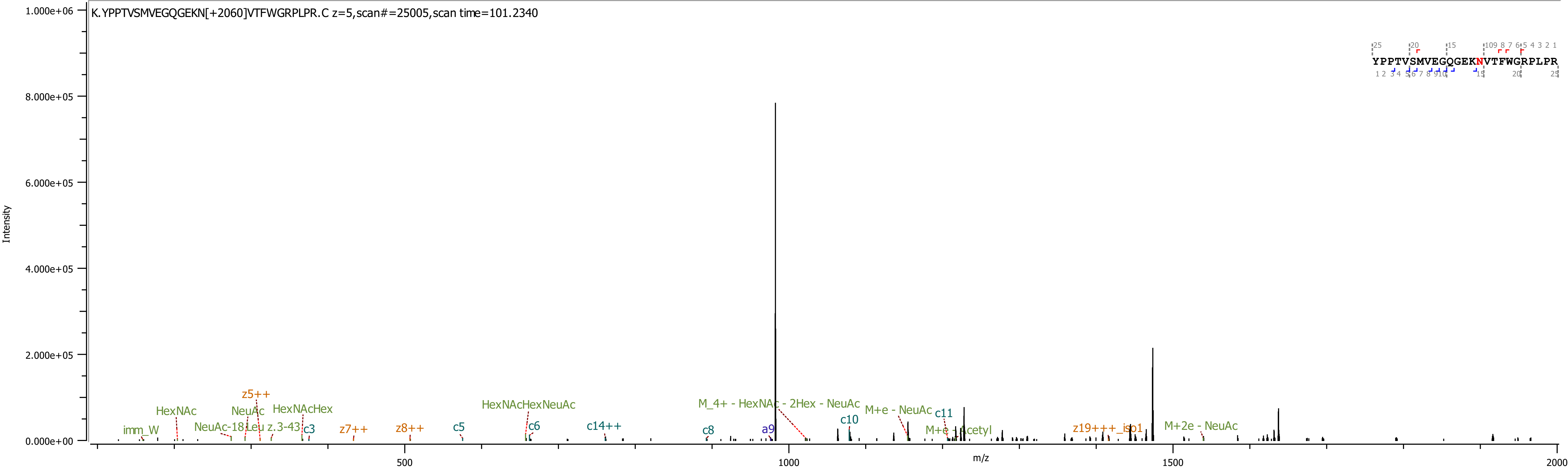
K.YPPTVSMVEGQGEKN[+2060]VTFWGRPLPR.C z=5,scan#=25005,scan time=101.2340
Intensity
m/z
YPPTVSMVEGQGEKNVTFWGRPLPR
imm_W
HexNAc
NeuAc-18
Leu
NeuAc
z5++
z.3-43
HexNAcHex
c3
z7++
z8++
c5
HexNAcHexNeuAc
c6
c14++
c8
a9
M_4+ - HexNAc - 2Hex - NeuAc
c10
M+e - NeuAc
c11
M+e - Acetyl
z19+++_iso1
M+2e - NeuAc

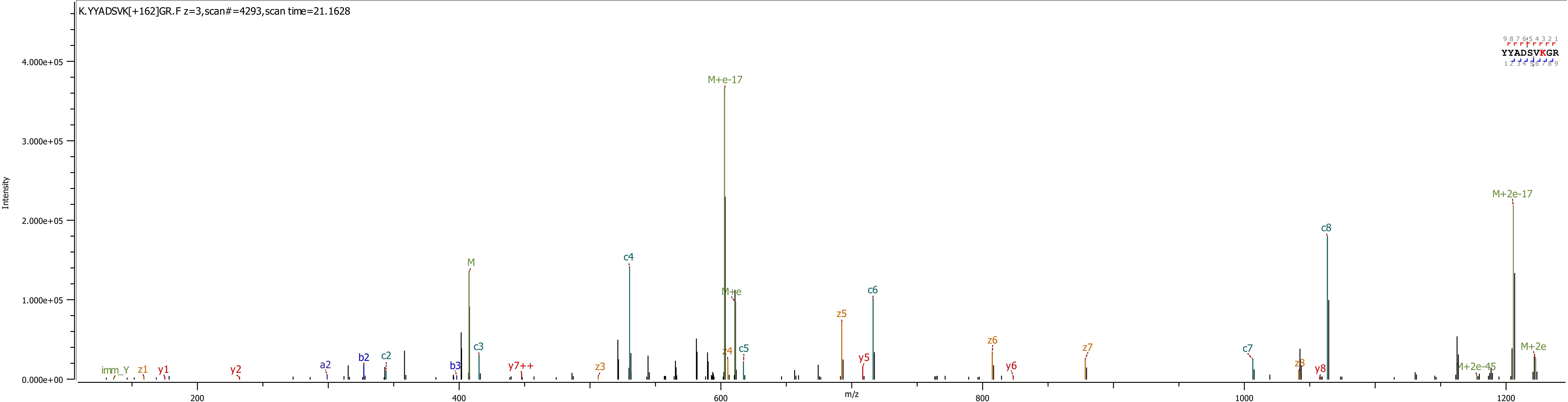
K.YYADSVK[+162]GR.F z=3,scan#=4293,scan time=21.1628
YYADSVKGR
Intensity
m/z
4.000e+05
3.000e+05
2.000e+05
1.000e+05
0.000e+00
200
400
600
800
1000
1200
M+e-17
M+2e-17
c8
M
c4
M+e
c6
z5
z6
z7
c7
c3
c5
z4
y5
c2
b2
a2
b3
y7++
z3
y6
z8
y8
M+2e
M+2e-45
imm_Y
z1
y1
y2
