# Supplementary material for: Cognitive decline in older adults with type 2 diabetes: Unraveling site-specific glycoproteomic alterations
Source: PLoS One. 2025 May 8;20(5):e0318916. doi: 10.1371/journal.pone.0318916 (PMC12061096; doi:10.1371/journal.pone.0318916)
Supplement: S11 File — (PDF) [file pone.0318916.s011.pdf]

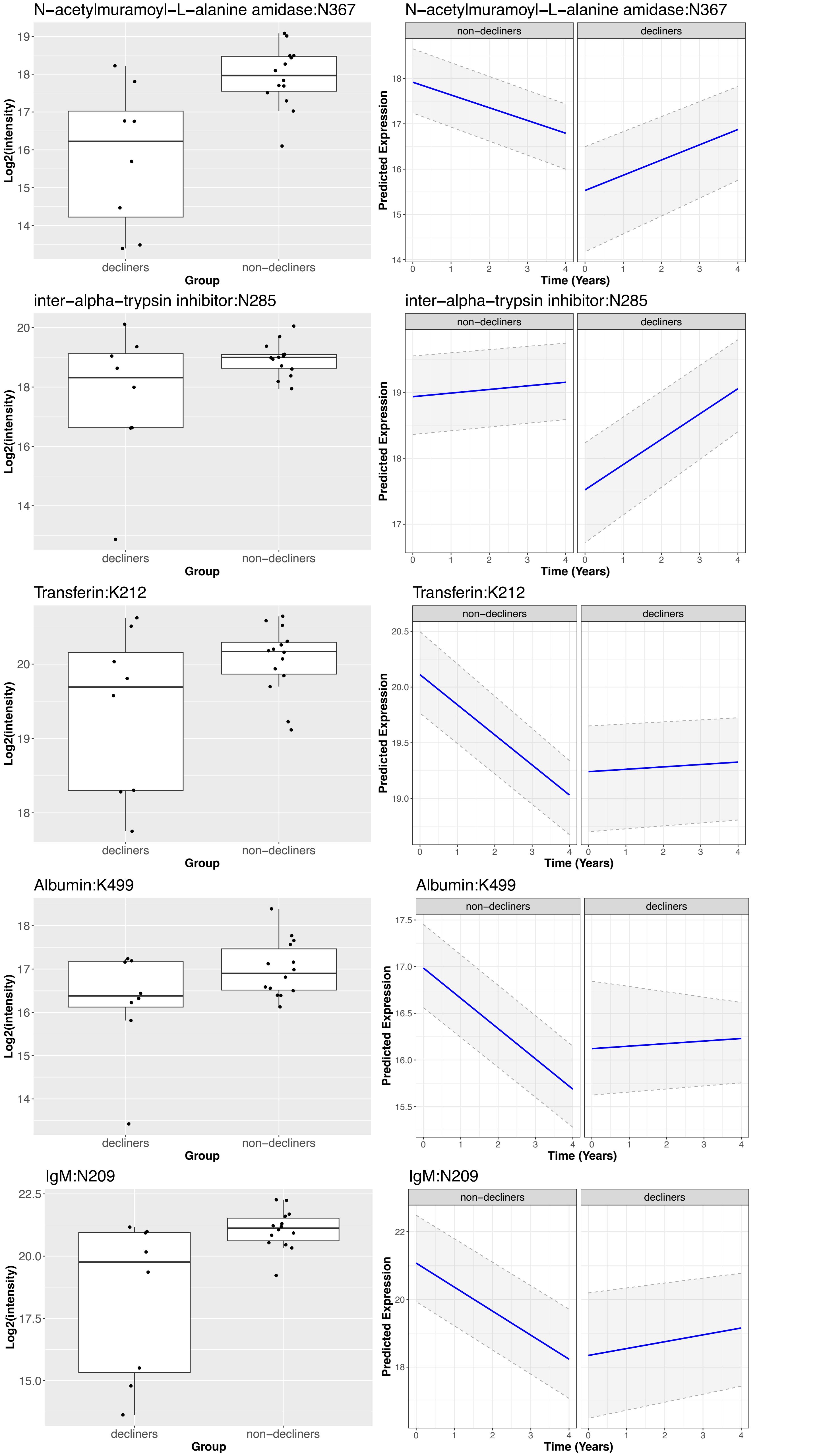
N−acetylmuramoyl−L−alanine amidase:N367
Log2(intensity)
19
18
17
16
15
14
decliners
non−decliners
Group
N−acetylmuramoyl−L−alanine amidase:N367
non−decliners
decliners
Predicted Expression
18
17
16
15
14
0 1 2 3 4
Time (Years)
inter−alpha−trypsin inhibitor:N285
Log2(intensity)
20
18
16
14
decliners
non−decliners
Group
inter−alpha−trypsin inhibitor:N285
non−decliners
decliners
Predicted Expression
19
18
17
0 1 2 3 4
Time (Years)
Transferin:K212
Log2(intensity)
20
19
18
decliners
non−decliners
Group
Transferin:K212
non−decliners
decliners
Predicted Expression
20.5
20.0
19.5
19.0
0 1 2 3 4
Time (Years)
Albumin:K499
Log2(intensity)
18
17
16
15
14
decliners
non−decliners
Group
Albumin:K499
non−decliners
decliners
Predicted Expression
17.5
17.0
16.5
16.0
15.5
0 1 2 3 4
Time (Years)
IgM:N209
Log2(intensity)
22.5
20.0
17.5
15.0
decliners
non−decliners
Group
IgM:N209
non−decliners
decliners
Predicted Expression
22
20
18
0 1 2 3 4
Time (Years)

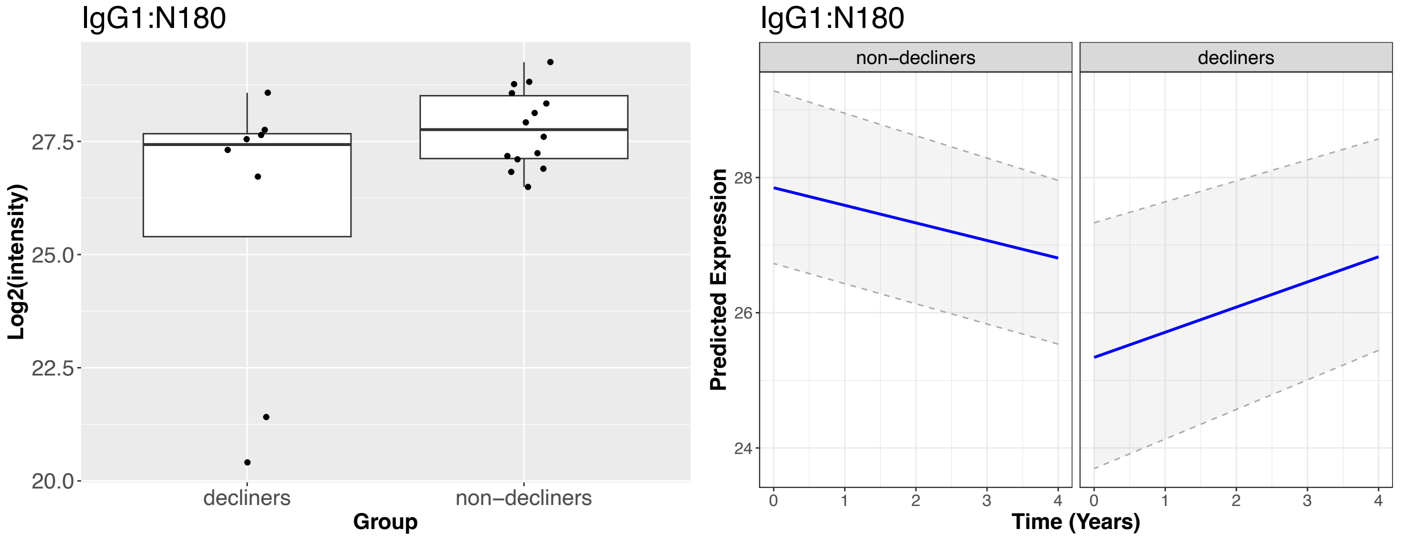
IgG1:N180
Log2(intensity)
Group
decliners
non−decliners
IgG1:N180
non−decliners
decliners
Predicted Expression
Time (Years)

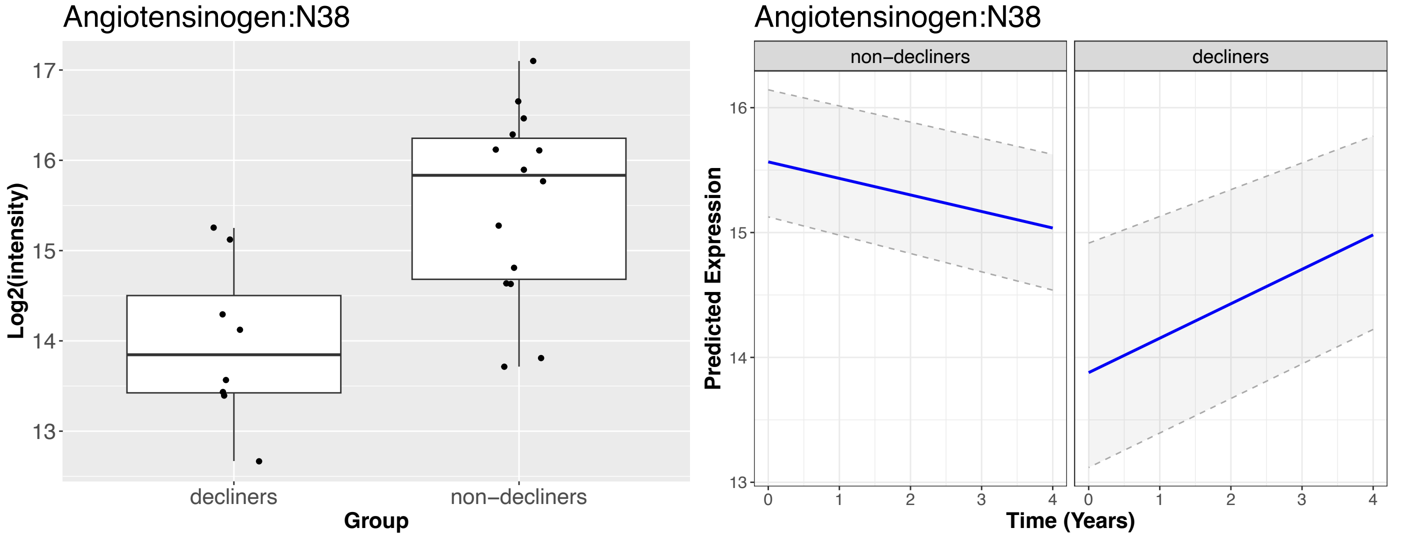
Angiotensinogen:N38
Log2(intensity)
Group
decliners
non−decliners
Angiotensinogen:N38
non−decliners
decliners
Predicted Expression
Time (Years)

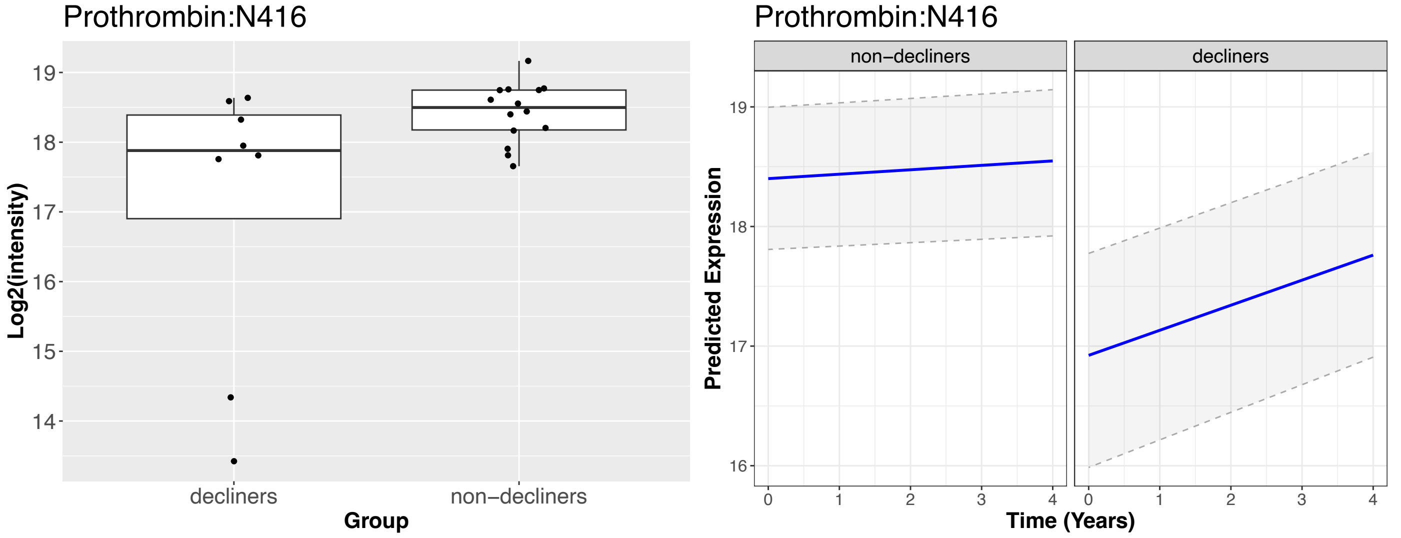
Prothrombin:N416
Log2(intensity)
Group
decliners
non−decliners
Prothrombin:N416
non−decliners
decliners
Predicted Expression
Time (Years)

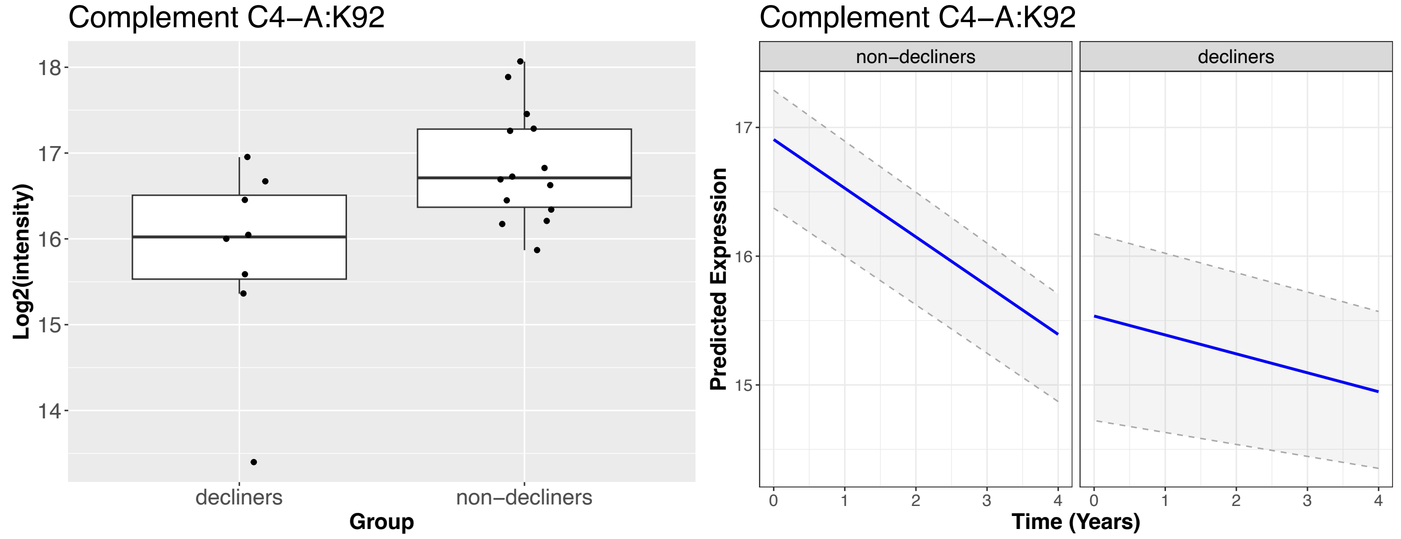
Complement C4−A:K92
Log2(intensity)
Group
decliners
non−decliners
Complement C4−A:K92
non−decliners
decliners
Predicted Expression
Time (Years)

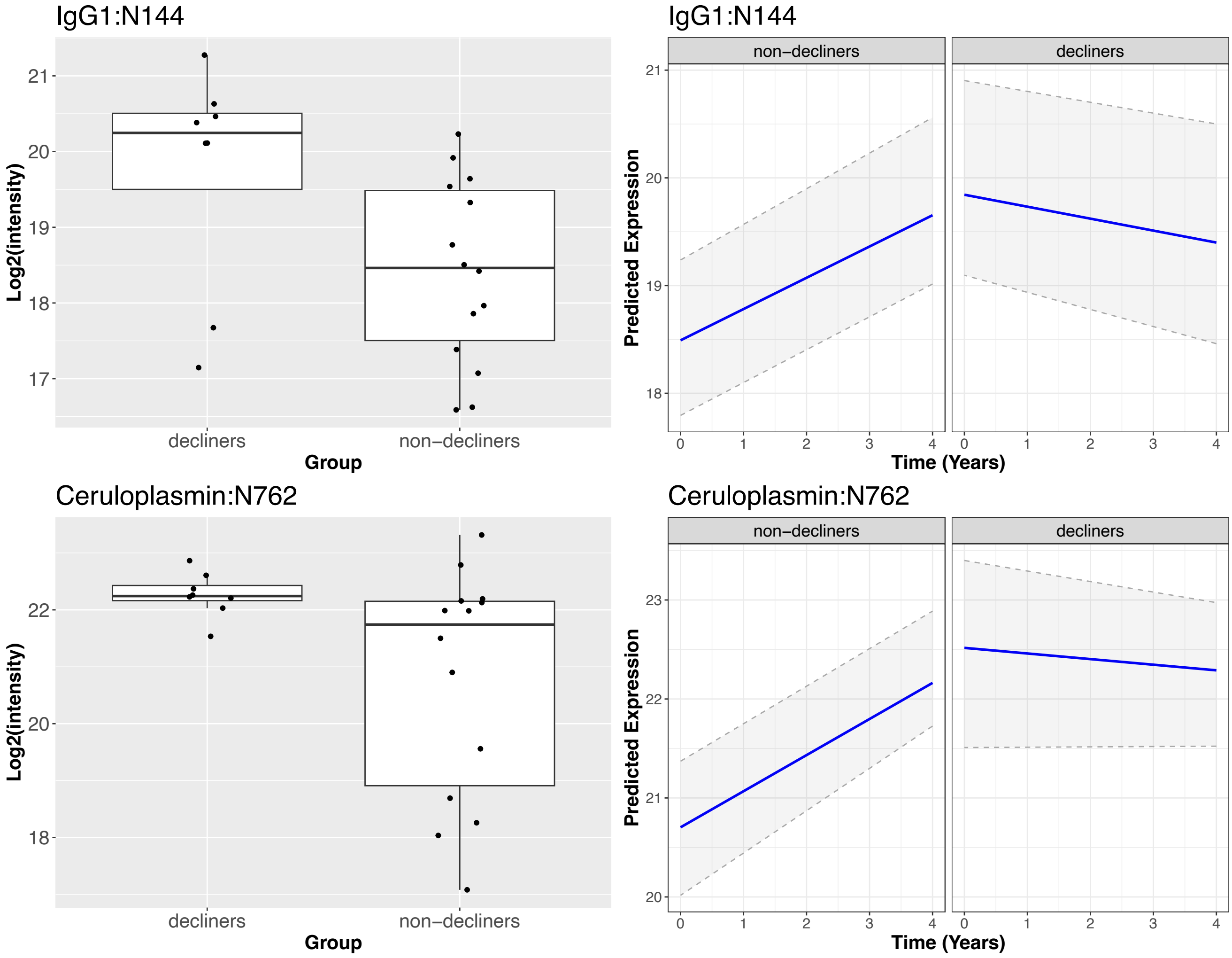
IgG1:N144
Log2(intensity)
21
20
19
18
17
decliners
non−decliners
Group
IgG1:N144
non−decliners
decliners
Predicted Expression
21
20
19
18
0
1
2
3
4
Time (Years)
Ceruloplasmin:N762
Log2(intensity)
22
20
18
decliners
non−decliners
Group
Ceruloplasmin:N762
non−decliners
decliners
Predicted Expression
23
22
21
20
Time (Years)

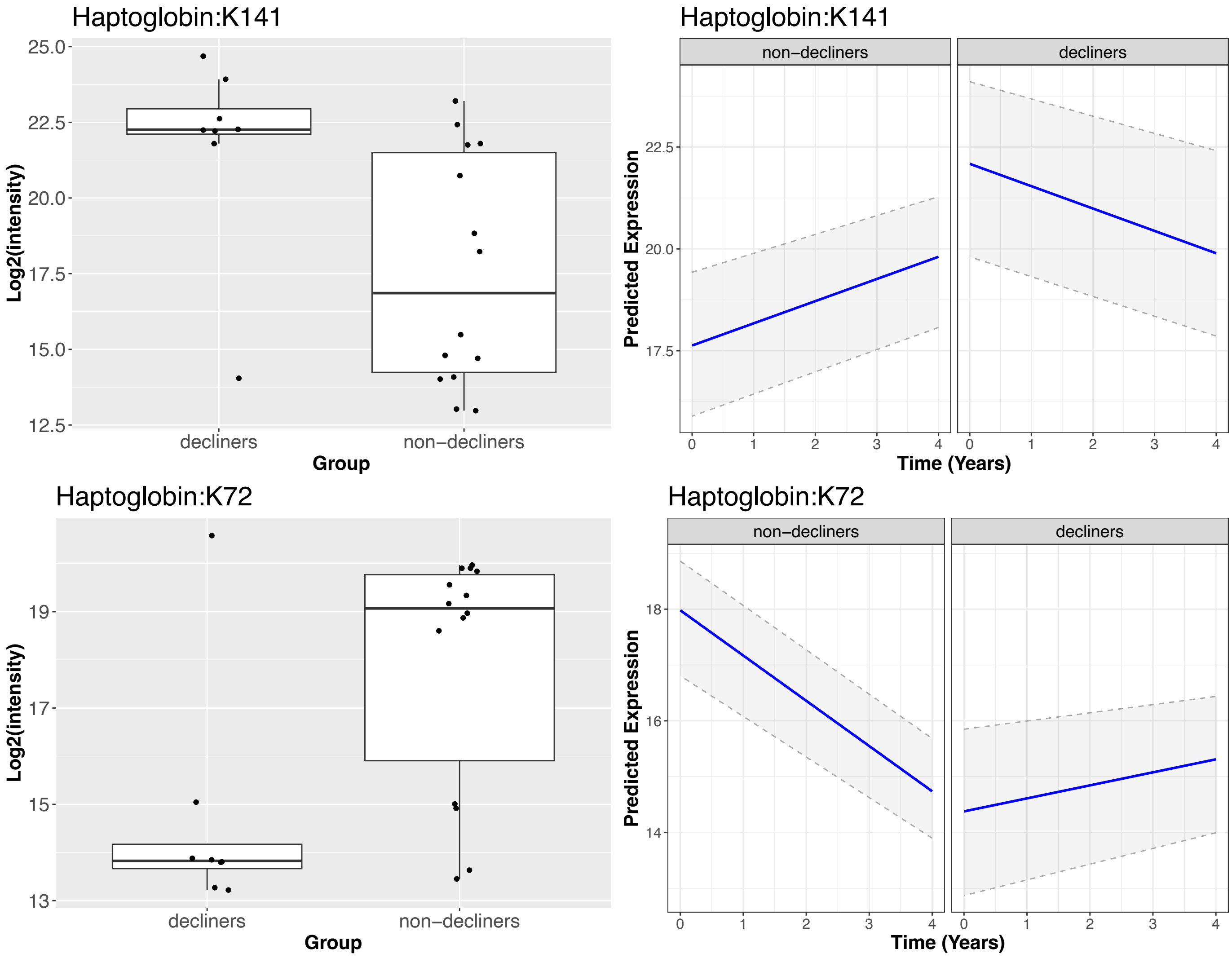
Haptoglobin:K141
Log2(intensity)
25.0
22.5
20.0
17.5
15.0
12.5
decliners
non−decliners
Group
Haptoglobin:K141
non−decliners
decliners
Predicted Expression
22.5
20.0
17.5
0
1
2
3
4
Time (Years)
Haptoglobin:K72
Log2(intensity)
19
17
15
13
decliners
non−decliners
Group
Haptoglobin:K72
non−decliners
decliners
Predicted Expression
18
16
14
Time (Years)
