# Supplementary material for: Cognitive decline in older adults with type 2 diabetes: Unraveling site-specific glycoproteomic alterations
Source: PLoS One. 2025 May 8;20(5):e0318916. doi: 10.1371/journal.pone.0318916 (PMC12061096; doi:10.1371/journal.pone.0318916)

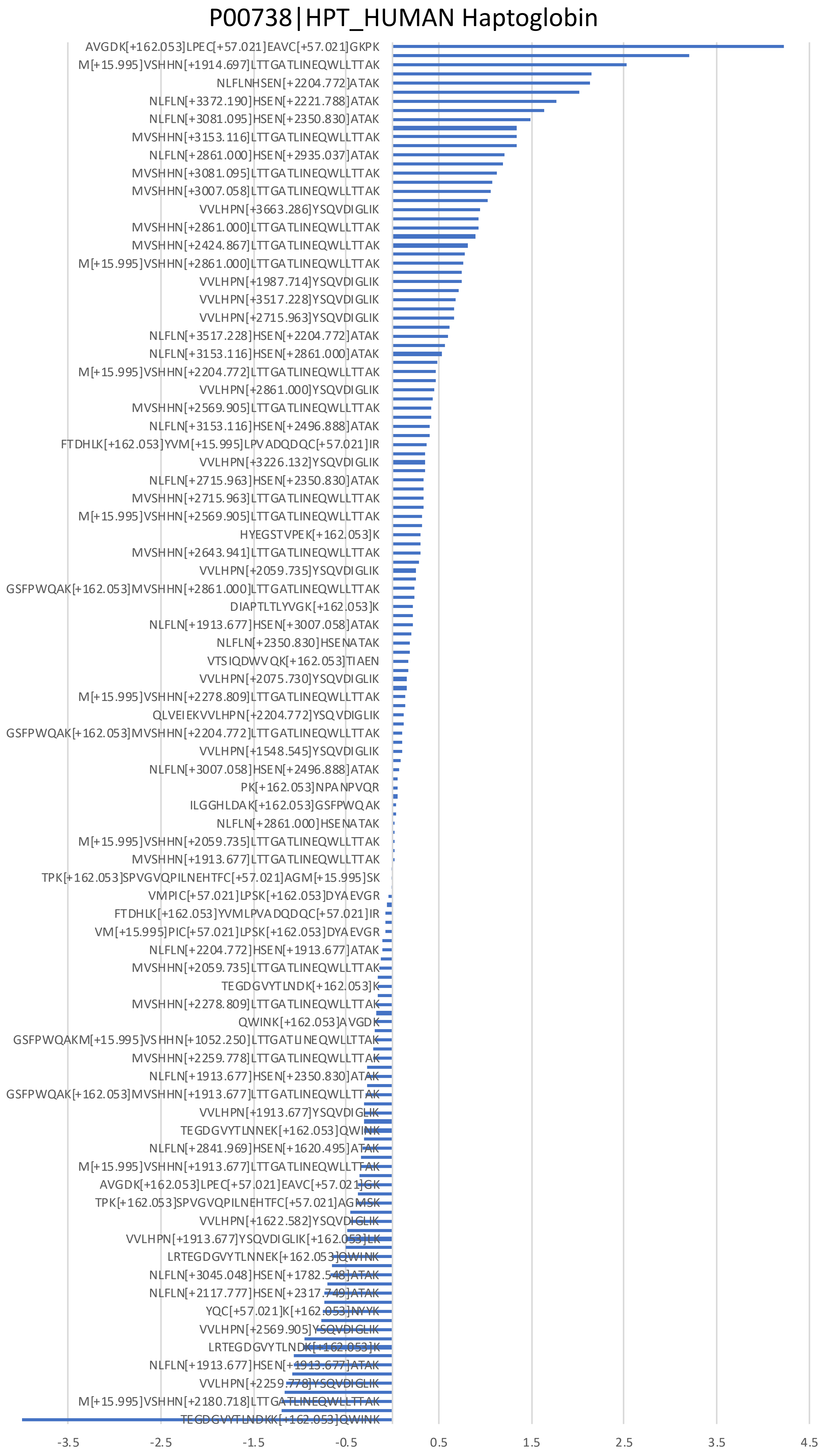
P00738|HPT_HUMAN Haptoglobin
AVGDK[+162.053]LPEC[+57.021]EAVC[+57.021]GKPK
M[+15.995]VSHHN[+1914.697]LTTGATLINEQWLLTTAK
NLFLNHSEN[+2204.772]ATAK
NLFLN[+3372.190]HSEN[+2221.788]ATAK
NLFLN[+3081.095]HSEN[+2350.830]ATAK
MVSHHN[+3153.116]LTTGATLINEQWLLTTAK
NLFLN[+2861.000]HSEN[+2935.037]ATAK
MVSHHN[+3081.095]LTTGATLINEQWLLTTAK
MVSHHN[+3007.058]LTTGATLINEQWLLTTAK
VVLHPN[+3663.286]YSQVDIGLIK
MVSHHN[+2861.000]LTTGATLINEQWLLTTAK
MVSHHN[+2424.867]LTTGATLINEQWLLTTAK
M[+15.995]VSHHN[+2861.000]LTTGATLINEQWLLTTAK
VVLHPN[+1987.714]YSQVDIGLIK
VVLHPN[+3517.228]YSQVDIGLIK
VVLHPN[+2715.963]YSQVDIGLIK
NLFLN[+3517.228]HSEN[+2204.772]ATAK
NLFLN[+3153.116]HSEN[+2861.000]ATAK
M[+15.995]VSHHN[+2204.772]LTTGATLINEQWLLTTAK
VVLHPN[+2861.000]YSQVDIGLIK
MVSHHN[+2569.905]LTTGATLINEQWLLTTAK
NLFLN[+3153.116]HSEN[+2496.888]ATAK
FTDHLK[+162.053]YVM[+15.995]LPVADQDQC[+57.021]IR
VVLHPN[+3226.132]YSQVDIGLIK
NLFLN[+2715.963]HSEN[+2350.830]ATAK
MVSHHN[+2715.963]LTTGATLINEQWLLTTAK
M[+15.995]VSHHN[+2569.905]LTTGATLINEQWLLTTAK
HYEGSTVPEK[+162.053]K
MVSHHN[+2643.941]LTTGATLINEQWLLTTAK
VVLHPN[+2059.735]YSQVDIGLIK
GSFPWQAK[+162.053]MVSHHN[+2861.000]LTTGATLINEQWLLTTAK
DIAPTLTLYVGK[+162.053]K
NLFLN[+1913.677]HSEN[+3007.058]ATAK
NLFLN[+2350.830]HSENATAK
VTSIQDWVQK[+162.053]TIAEN
VVLHPN[+2075.730]YSQVDIGLIK
M[+15.995]VSHHN[+2278.809]LTTGATLINEQWLLTTAK
QLVEIEKVVLHPN[+2204.772]YSQVDIGLIK
GSFPWQAK[+162.053]MVSHHN[+2204.772]LTTGATLINEQWLLTTAK
VVLHPN[+1548.545]YSQVDIGLIK
NLFLN[+3007.058]HSEN[+2496.888]ATAK
PK[+162.053]NPANPVQR
ILGGHLDAK[+162.053]GSFPWQAK
NLFLN[+2861.000]HSENATAK
M[+15.995]VSHHN[+2059.735]LTTGATLINEQWLLTTAK
MVSHHN[+1913.677]LTTGATLINEQWLLTTAK
TPK[+162.053]SPVGVQPILNEHTFC[+57.021]AGM[+15.995]SK
VMPIC[+57.021]LPSK[+162.053]DYAEVGR
FTDHLK[+162.053]YVMLPVADQDQC[+57.021]IR
VM[+15.995]PIC[+57.021]LPSK[+162.053]DYAEVGR
NLFLN[+2204.772]HSEN[+1913.677]ATAK
MVSHHN[+2059.735]LTTGATLINEQWLLTTAK
TEGDGVYTLNDK[+162.053]K
MVSHHN[+2278.809]LTTGATLINEQWLLTTAK
QWINK[+162.053]AVGDK
GSFPWQAKM[+15.995]VSHHN[+1052.250]LTTGATLINEQWLLTTAK
MVSHHN[+2259.778]LTTGATLINEQWLLTTAK
NLFLN[+1913.677]HSEN[+2350.830]ATAK
GSFPWQAK[+162.053]MVSHHN[+1913.677]LTTGATLINEQWLLTTAK
VVLHPN[+1913.677]YSQVDIGLIK
TEGDGVYTLNNEK[+162.053]QWINK
NLFLN[+2841.969]HSEN[+1620.495]ATAK
M[+15.995]VSHHN[+1913.677]LTTGATLINEQWLLTTAK
AVGDK[+162.053]LPEC[+57.021]EAVC[+57.021]GK
TPK[+162.053]SPVGVQPILNEHTFC[+57.021]AGMSK
VVLHPN[+1622.582]YSQVDIGLIK
VVLHPN[+1913.677]YSQVDIGLIK[+162.053]LK
LRTEGDGVYTLNNEK[+162.053]QWINK
NLFLN[+3045.048]HSEN[+1782.548]ATAK
NLFLN[+2117.777]HSEN[+2317.749]ATAK
YQC[+57.021]K[+162.053]NYYK
VVLHPN[+2569.905]YSQVDIGLIK
LRTEGDGVYTLNDK[+162.053]K
NLFLN[+1913.677]HSEN[+1913.677]ATAK
VVLHPN[+2259.778]YSQVDIGLIK
M[+15.995]VSHHN[+2180.718]LTTGATLINEQWLLTTAK
TEGDGVYTLNDKK[+162.053]QWINK
-3.5
-2.5
-1.5
-0.5
0.5
1.5
2.5
3.5
4.5

# P01871|IGHM_HUMAN Immunoglobulin heavy constant mu

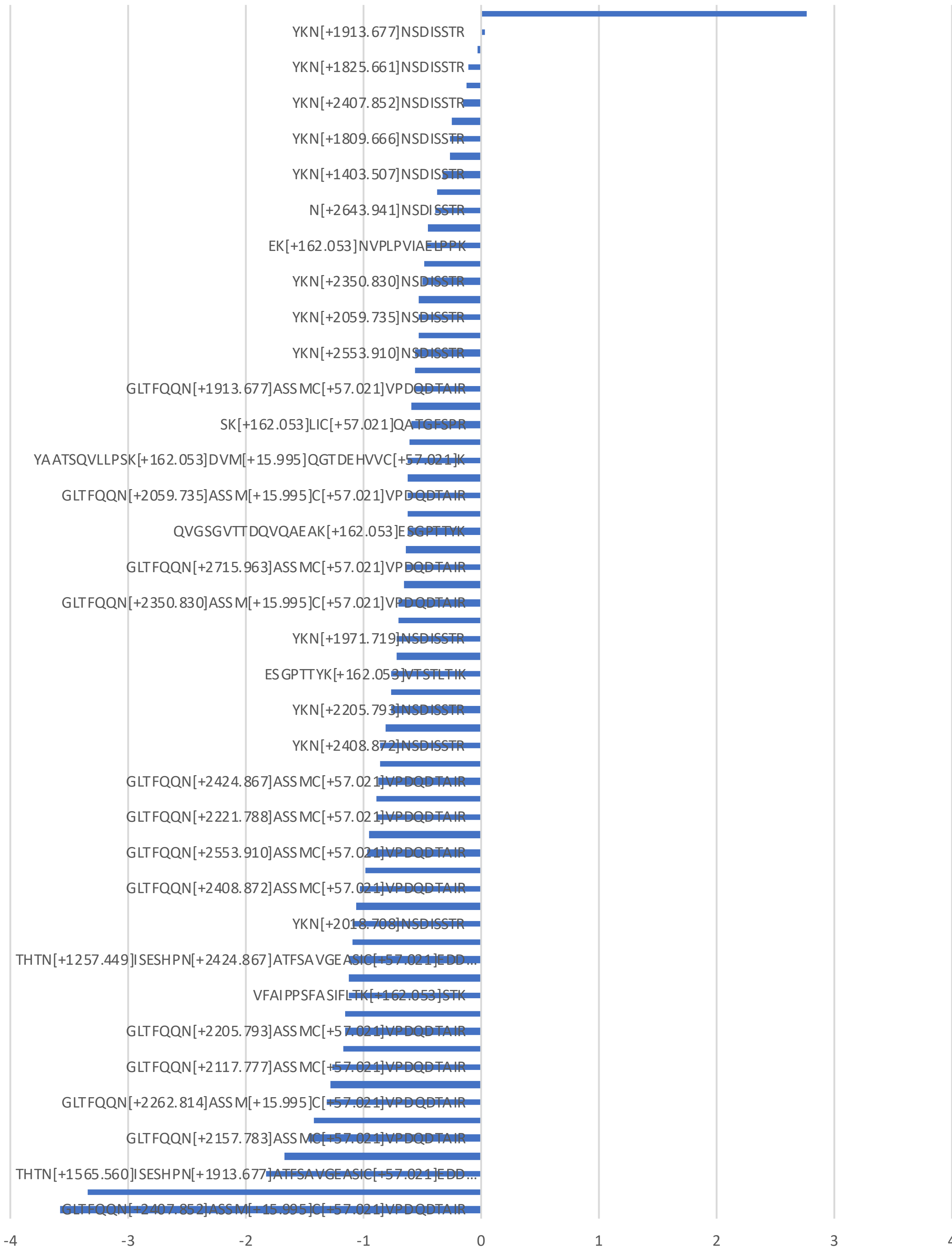

## Q96PD5|PGRP2_HUMAN N-acetylmuramoyl-L-alanine amidase

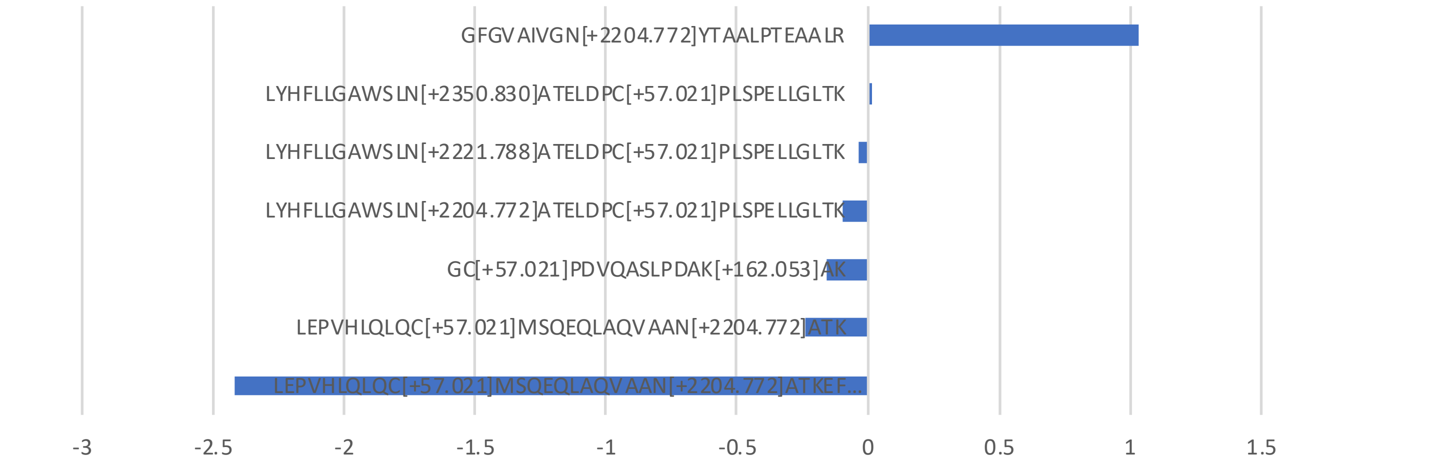


## P01876|IGHA1_HUMAN Immunoglobulin heavy constant alpha 1

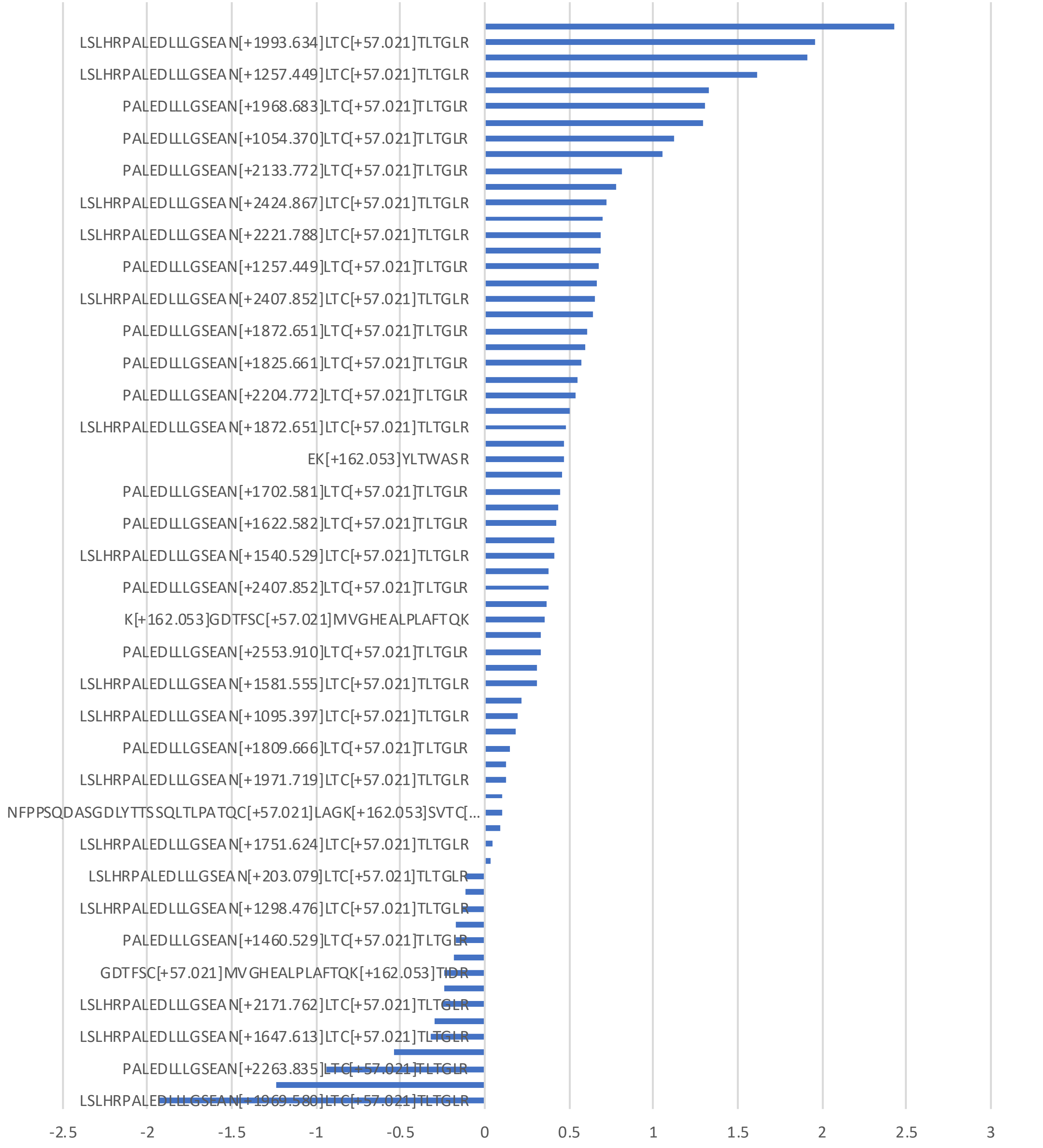

# P01019|ANGT_HUMAN Angiotensinogen

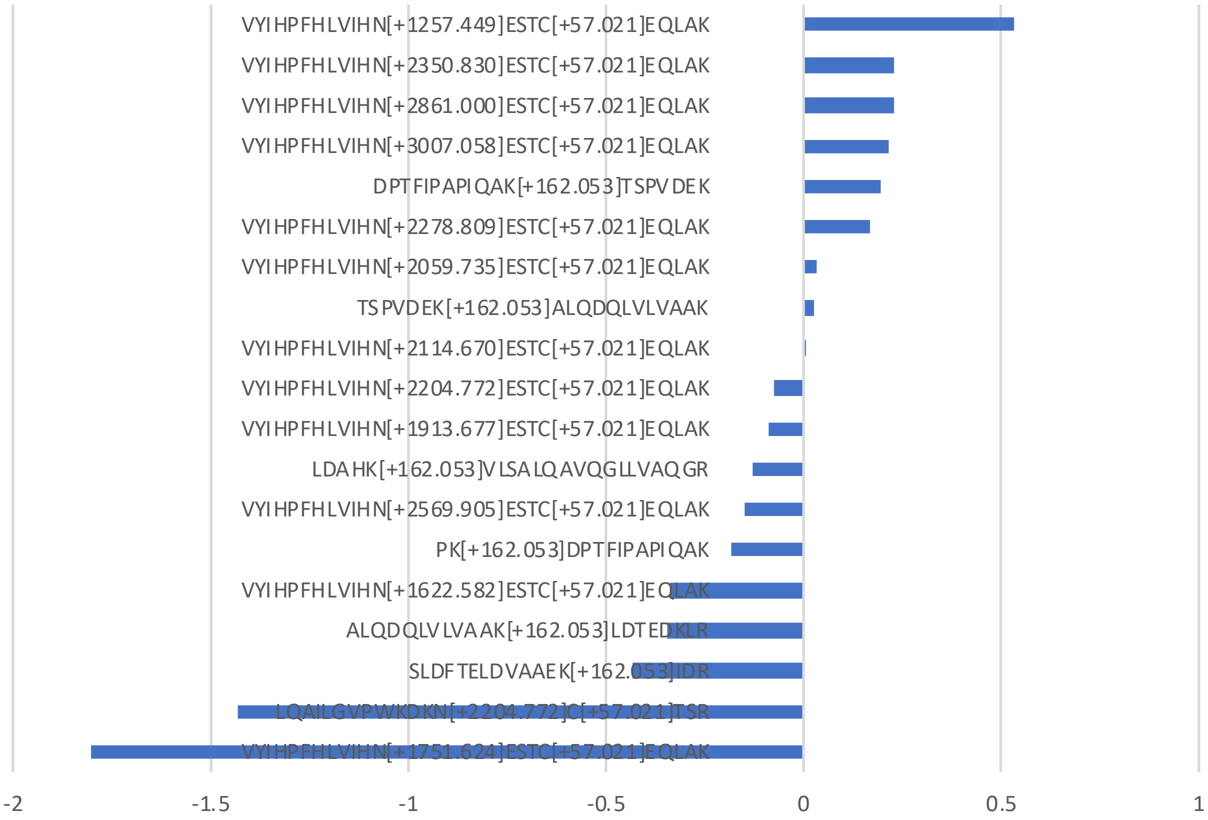


# P19827|ITIH1_HUMAN Inter-alpha-trypsin inhibitor heavy chain H1

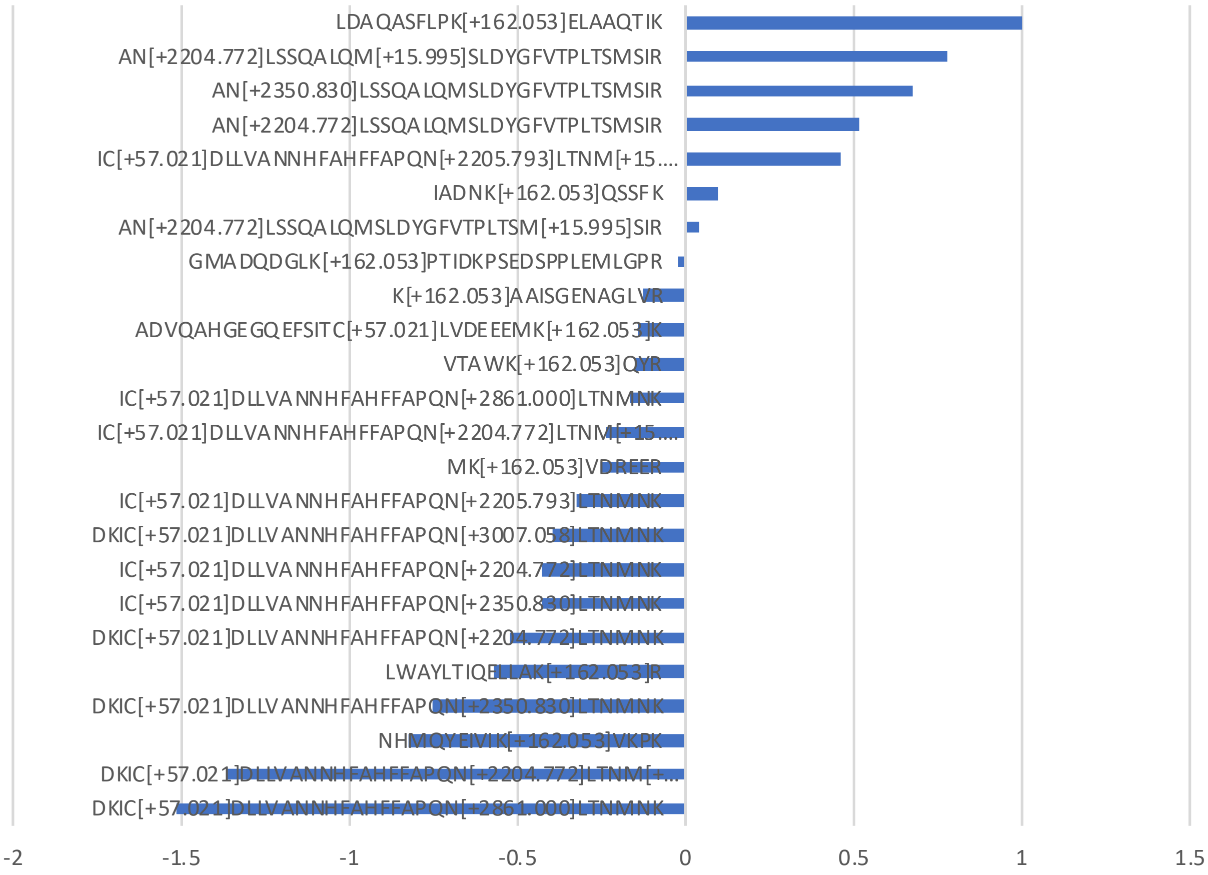

# P02787|TRFE_HUMAN Serotransferrin

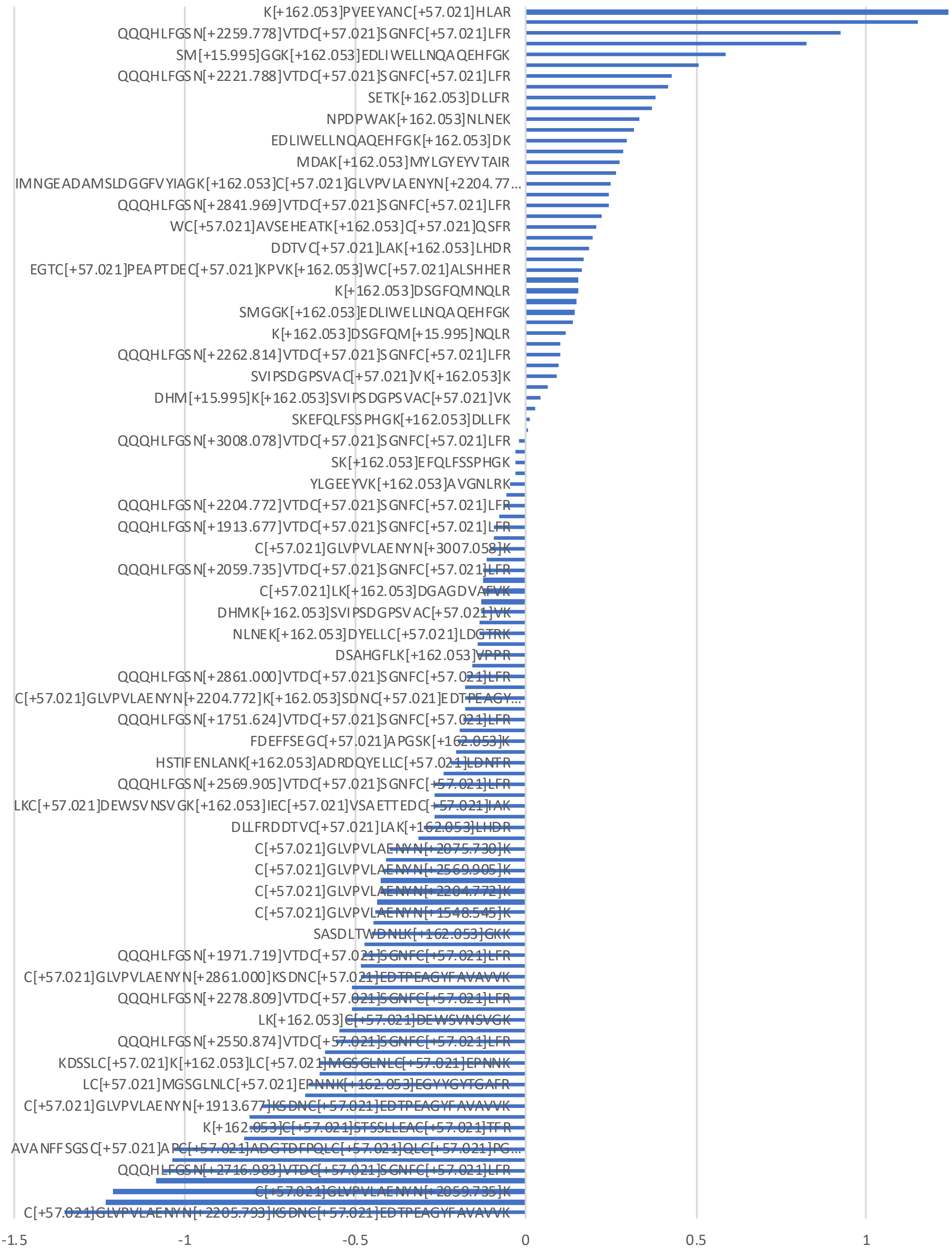

## P01857|IGHG1_HUMAN Immunoglobulin heavy constant gamma 1

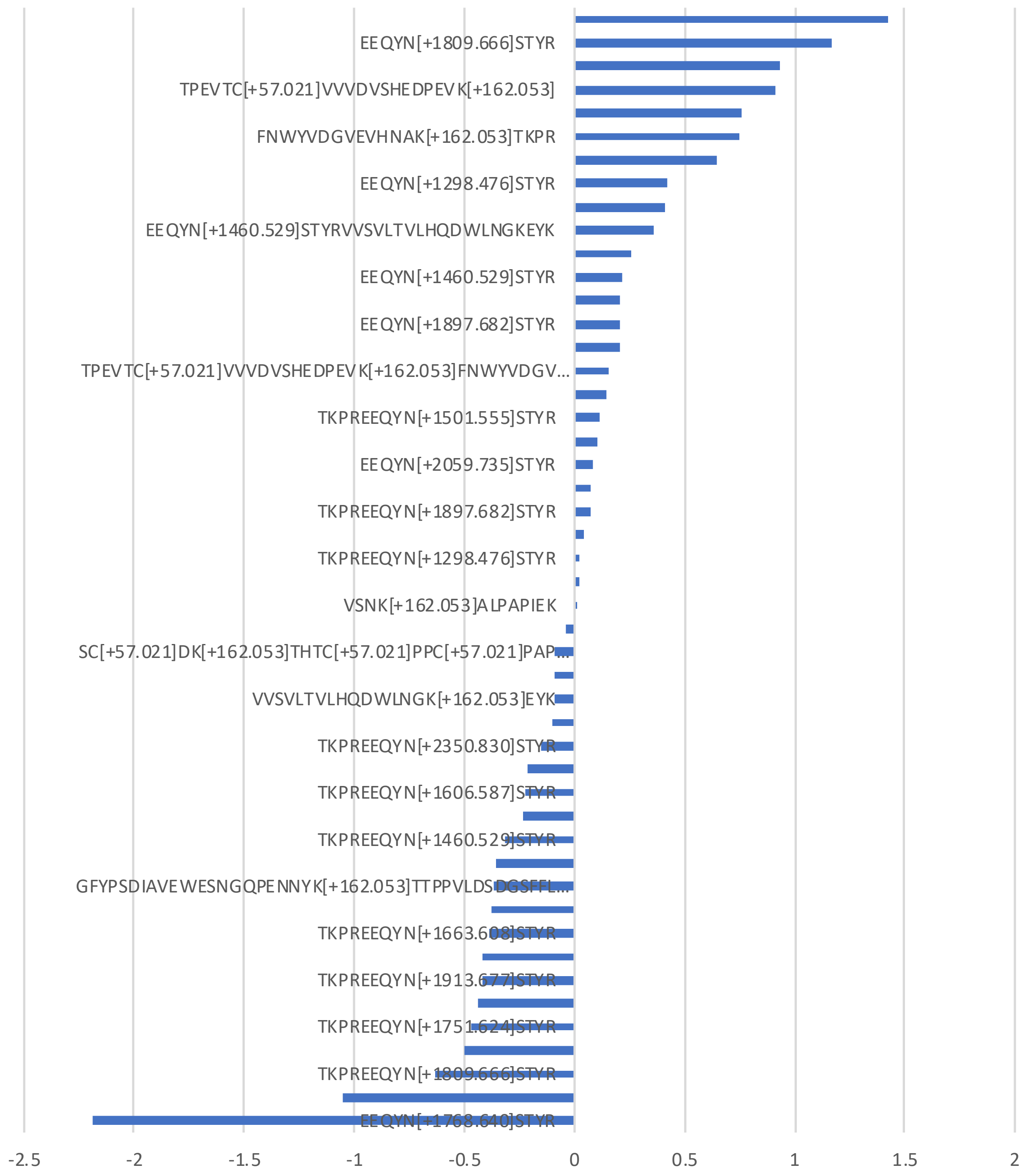


## P0C0L5|CO4B_HUMAN Complement C4-B

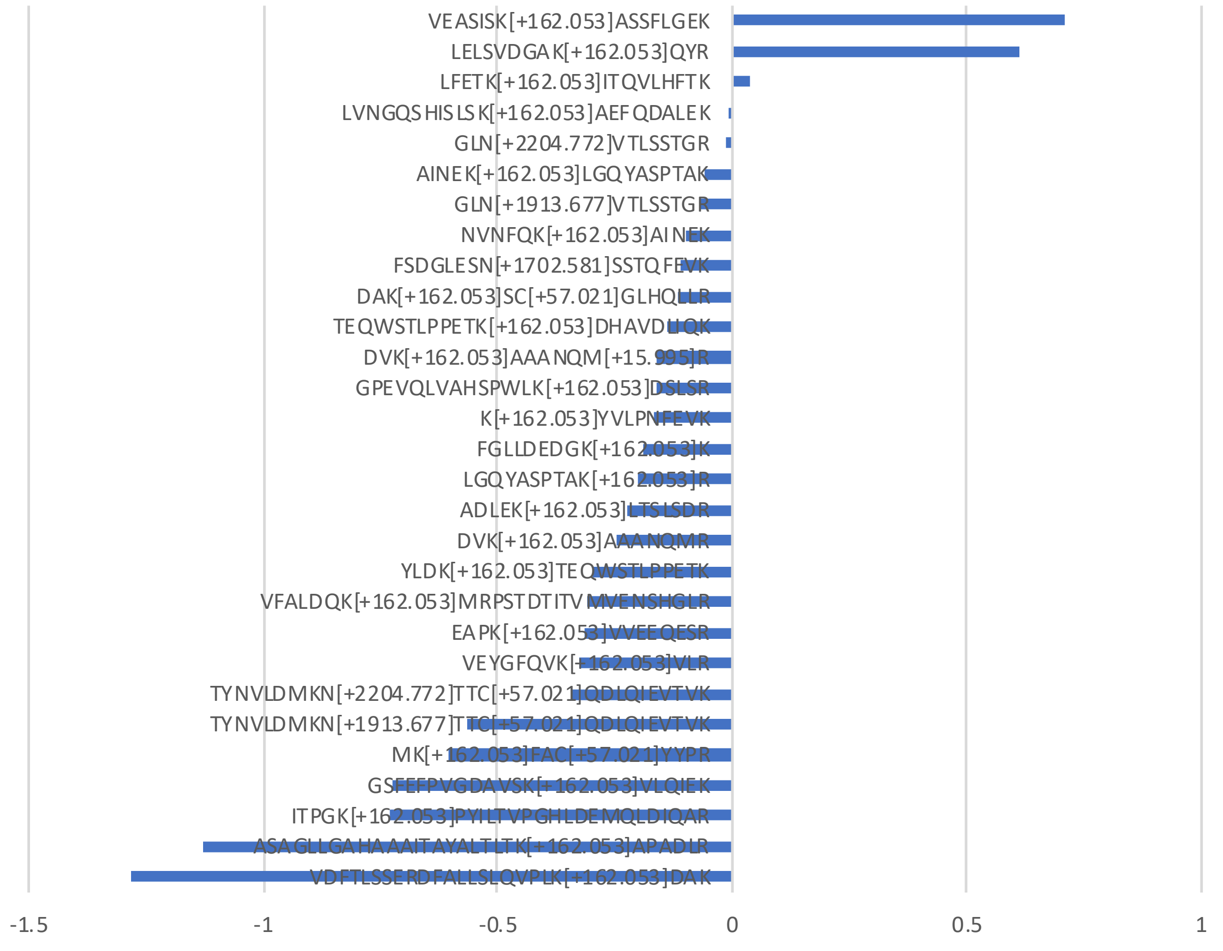

# P02768|ALBU_HUMAN Albumin

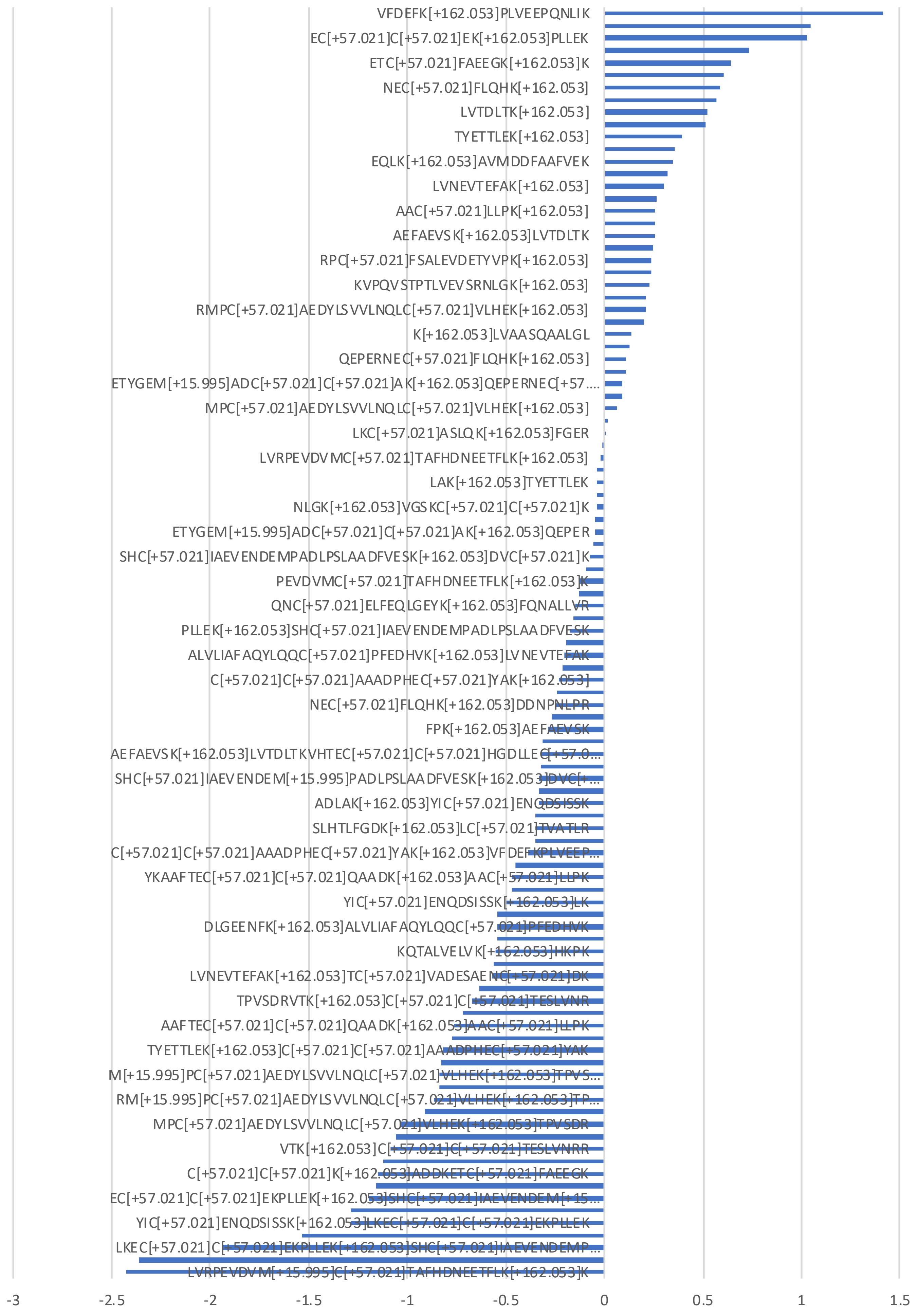

# P00734|THRB_HUMAN Prothrombin

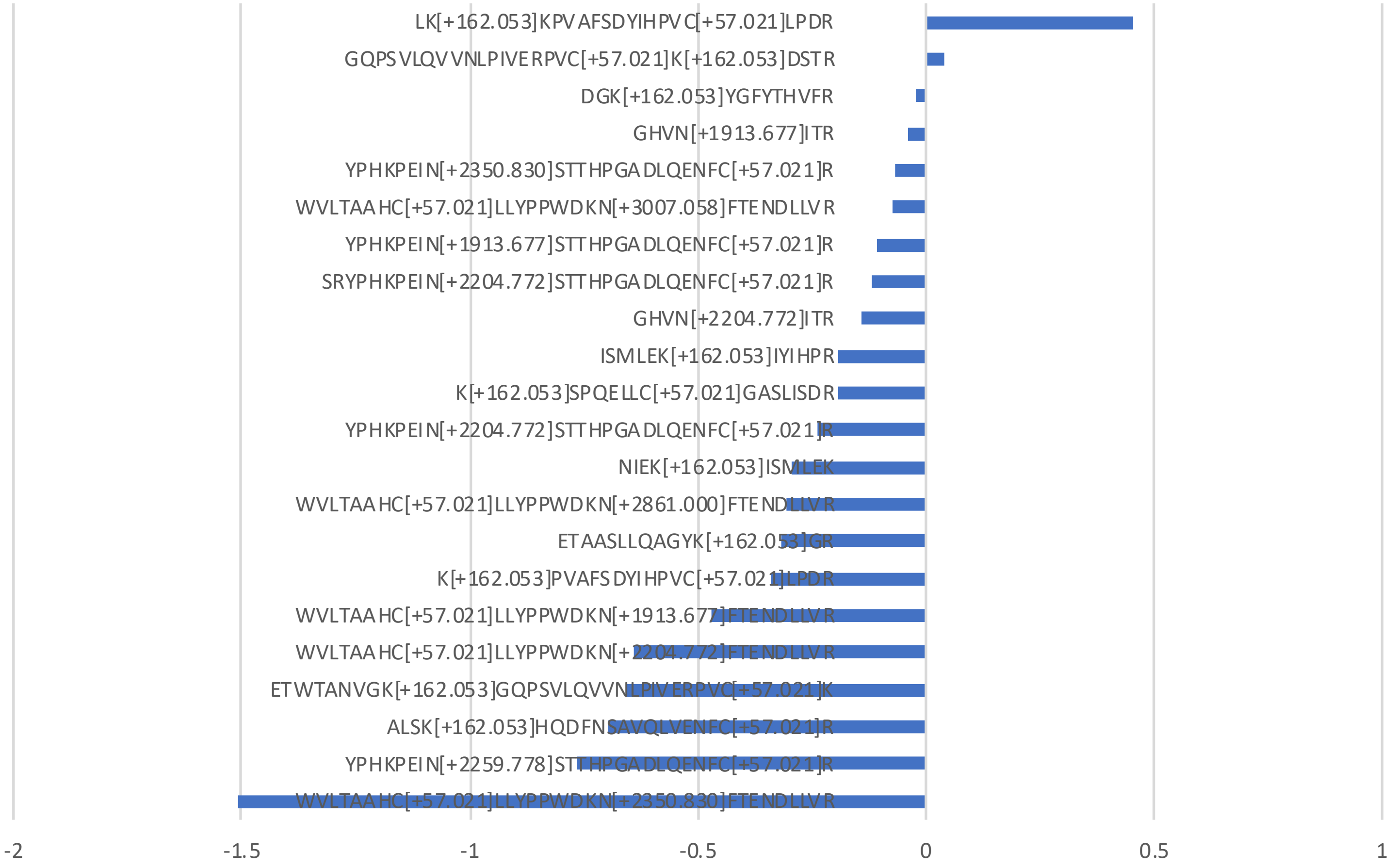


# P00450|CERU_HUMAN Ceruloplasmin

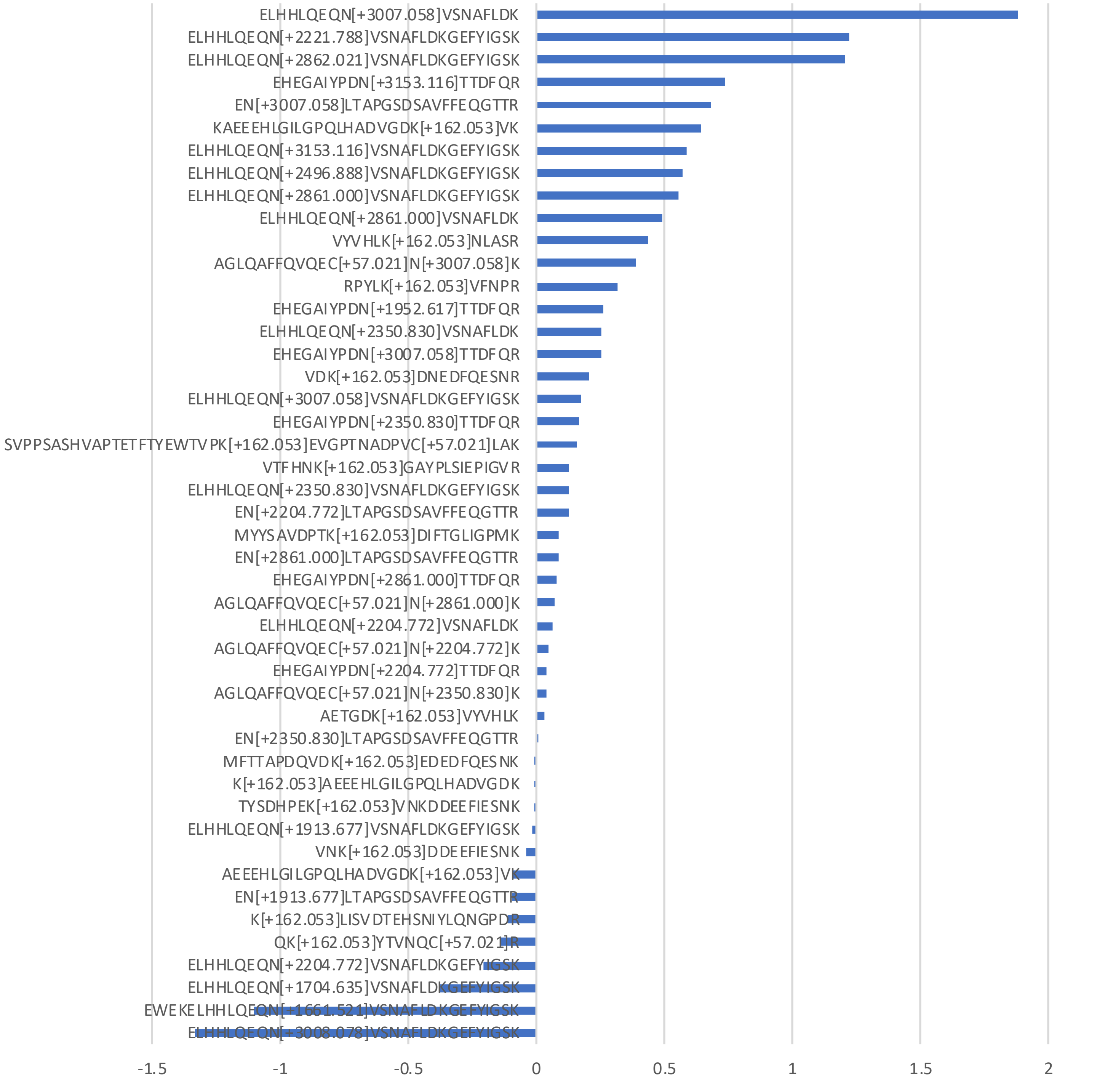

Supplement: S12 File — (PDF) [file pone.0318916.s012.pdf]
